# Small-molecule inhibitors, immune checkpoint inhibitors, and more: FDA-approved novel therapeutic drugs for solid tumors from 1991 to 2021

**DOI:** 10.1186/s13045-022-01362-9

**Published:** 2022-10-08

**Authors:** Qing Wu, Wei Qian, Xiaoli Sun, Shaojie Jiang

**Affiliations:** 1 School of Medical Imaging, Hangzhou Medical College, Hangzhou, 310053 Zhejiang China; 2 Department of Radiology, School of Medicine, The Second Affiliated Hospital, Zhejiang University, Hangzhou, 310009 Zhejiang China; 3 Department of Radiation Oncology, School of Medicine, The First Affiliated Hospital, Zhejiang University, Hangzhou, 310003 Zhejiang China

**Keywords:** The United States Food and Drug Administration, Solid tumors, Receptor tyrosine kinase inhibitors, Immune checkpoint blockades

## Abstract

The United States Food and Drug Administration (US FDA) has always been a forerunner in drug evaluation and supervision. Over the past 31 years, 1050 drugs (excluding vaccines, cell-based therapies, and gene therapy products) have been approved as new molecular entities (NMEs) or biologics license applications (BLAs). A total of 228 of these 1050 drugs were identified as cancer therapeutics or cancer-related drugs, and 120 of them were classified as therapeutic drugs for solid tumors according to their initial indications. These drugs have evolved from small molecules with broad-spectrum antitumor properties in the early stage to monoclonal antibodies (mAbs) and antibody‒drug conjugates (ADCs) with a more precise targeting effect during the most recent decade. These drugs have extended indications for other malignancies, constituting a cancer treatment system for monotherapy or combined therapy. However, the available targets are still mainly limited to receptor tyrosine kinases (RTKs), restricting the development of antitumor drugs. In this review, these 120 drugs are summarized and classified according to the initial indications, characteristics, or functions. Additionally, RTK-targeted therapies and immune checkpoint-based immunotherapies are also discussed. Our analysis of existing challenges and potential opportunities in drug development may advance solid tumor treatment in the future.

**Supplementary Information:**

The online version contains supplementary material available at 10.1186/s13045-022-01362-9.

## Background

**Fig. 1:**
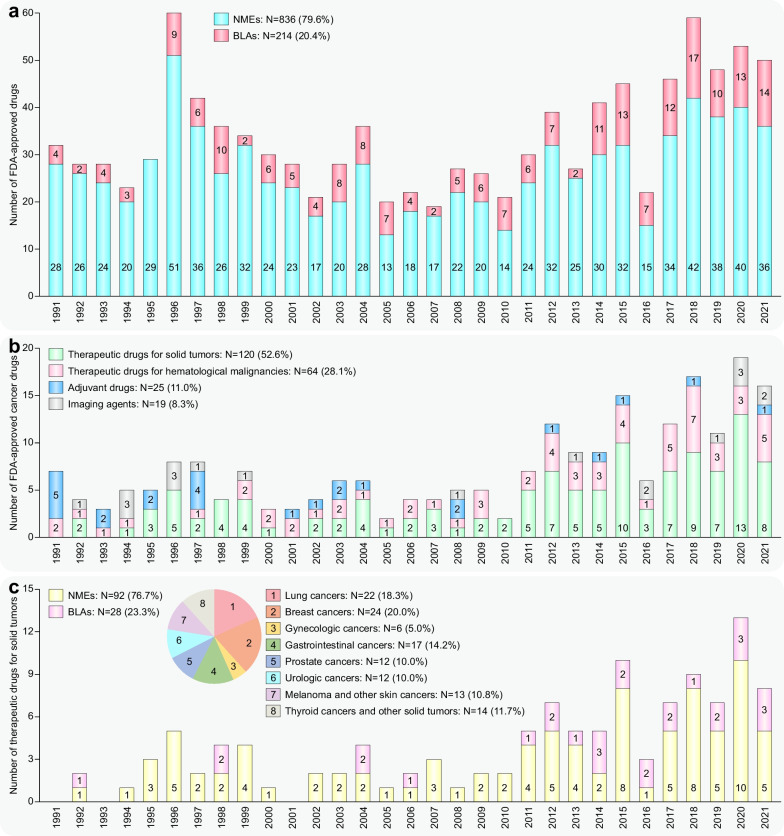
Statistics of FDA-approved drugs and cancer drugs. **a** Number of FDA-approved drugs (NMEs: New molecular entities, BLAs: Biologics license applications) over the past 31 years. **b** Number of FDA-approved cancer drugs over the past 31 years. **c** Number of FDA-approved therapeutic drugs for solid tumors during the past 31 years

Cancer is the first or second leading cause of premature death in all countries except Africa, second only to cardiovascular disease [1]. An estimated 19.3 million new cancer cases and almost 10 million cancer-related deaths occurred in 2020 worldwide [2]. Solid tumors represent more than 90% of human cancers and cancer-related mortalities [2]. For unresectable locally advanced or metastatic solid tumors, therapeutic drugs have always been the mainstream strategy. Profound changes have occurred in therapeutic drugs for solid tumors during the past 31 years. Both the number of solid tumor drugs and their proportion among all FDA-approved drugs increased in this period, especially in the most recent decade (Fig. 1a, b). More importantly, cytotoxic drugs have evolved into drugs with more precise targeting effects, including small-molecule targeted drugs, monoclonal antibodies (mAbs), and antibody–drug conjugates (ADCs), and the proportion of biological drugs has increased accordingly (Fig. 1c).

During the past three decades, the FDA granted 120 approvals for novel solid tumor therapeutic drugs (Additional file 1: Table S1–S3), and these drugs treat the most high-incidence solid tumors, including lung cancer, breast cancer, prostate cancer, gastrointestinal cancers, etc. These drugs constitute the mainstay of the modern cancer treatment system for solid tumors and hematological malignancies. Despite extraordinary achievements, the effective application of these drugs is still limited by great challenges, such as drug resistance [3], adverse effects [4], and even hyperprogressive disease with programmed death receptor-1 (PD1)/programmed death-ligand 1 (PDL1)-based immunotherapy [5].

This review describes the properties of 120 therapeutic drugs for solid tumors, summarizes the main biological mechanisms of their antitumor activity, and analyzes the target distribution of these drugs. Additionally, we elaborate on the challenges and opportunities in developing solid tumor therapeutic drugs and provide constructive suggestions and helpful solutions for the further study of solid tumor treatment.

### FDA-approved therapeutic drugs for lung cancers

Lung cancer accounted for 11.4% of cancer cases and 18.0% of cancer-related deaths worldwide in 2020. Although the incidence rate of lung cancer was surpassed by that of breast cancer in 2020, its mortality rate still far exceeded that of any other type of cancer [2]. Over the past 31 years, the FDA has granted approvals for 22 novel therapeutic drugs (including 20 small molecules and two mAbs) for lung cancer.

#### Non-small cell lung cancer

**Fig. 2:**
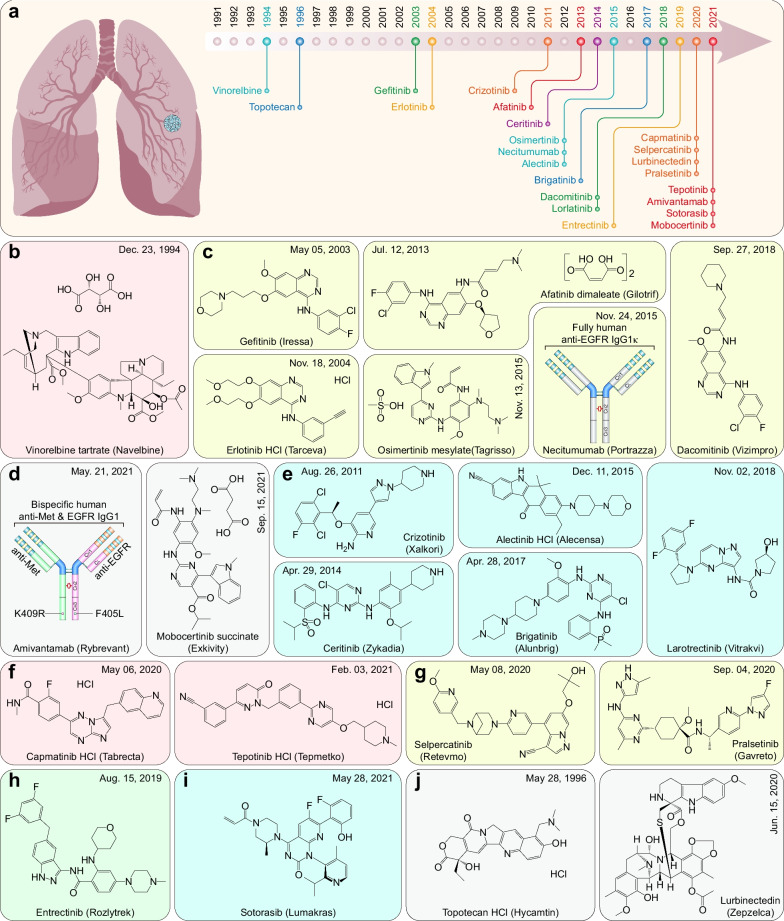
FDA-approved therapeutic drugs for lung cancers. **a** Distribution of therapeutic drugs for lung cancers during the past 31 years (adapted from [126]). **b** Microtubule inhibitor. **c** EGFR inhibitors and EGFR-directed mAb. **d** EGFR- and MET-bispecific antibody and EGFR inhibitor for NSCLC with *EGFR*^ex20ins^ mutations. **e** ALK inhibitors. **f** MET inhibitors. **g** RET inhibitors. **h** Multitarget TKI. **i** KRAS^G12C^-targeted small-molecule inhibitor. **j** DNA topoisomerase inhibitor and DNA alkylating agent for SCLC

Non-small cell lung cancer (NSCLC) includes adenocarcinoma, squamous cell carcinoma (SCC), and large-cell carcinoma (LCC) and accounts for approximately 85% of all lung cancer cases [6]. The majority of diagnosed NSCLC cases present as locally advanced or metastatic diseases [7]. Twenty of the 22 therapeutic drugs are approved for NSCLC as the initial indication, and most of them are classified as epidermal growth factor receptor (EGFR) and anaplastic lymphoma kinase (ALK) inhibitors. Therefore, *EGFR* mutation and *ALK* rearrangement tests are recommended for NSCLC before EGFR- or ALK-directed therapies [8, 9] (Fig. 2a and Table 1).

**Table 1:** FDA-approved therapeutic drugs for lung cancers

Drug (brand name)	Sponsor	Properties	Indication	Approval date	Review
Vinorelbine tartrate (Navelbine)	Pierre Fabre	Microtubule-destabilizing agent	NSCLC	12/23/1994	P
Gefitinib (Iressa)	AstraZeneca	EGFR inhibitor	NSCLC	05/05/2003	P
Erlotinib HCl (Tarceva)	OSI Pharmas	EGFR inhibitor	NSCLC	11/18/2004	P
Afatinib dimaleate (Gilotrif)	Boehringer Ingelheim	EGFR, HER2, and HER4 inhibitor	Metastatic NSCLC with *EGFR* exon 19 deletion or exon 21 (L858R) mutation	07/12/2013	P, O
Osimertinib mesylate (Tagrisso)	AstraZeneca	EGFR inhibitor	NSCLC with *EGFR*^T790M^ mutations	11/13/2015	P, O
Dacomitinib (Vizimpro)	Pfizer	EGFR inhibitor	*EGFR*-mutated NSCLC	09/27/2018	P, O
Necitumumab (Portrazza)	Eli Lilly	EGFR-directed mAb	NSCLC	11/24/2015	S, O
Amivantamab (Rybrevant)	Janssen Biotech	EGFR- and MET-bispecific antibody	*EGFR* exon 20-mutated NSCLC	05/21/2021	P
Mobocertinib succinate (Exkivity)	Takeda	EGFR inhibitor	*EGFR* exon 20-mutated NSCLC	09/15/2021	P, O
Crizotinib (Xalkori)	Merck & Co	Multitarget TKI (ALK, ROS1, and MET)	ALK-positive advanced or metastatic NSCLC	08/26/2011	P, O
Ceritinib (Zykadia)	Novartis	Multitarget TKI (ALK, IGF1R, INSR, and ROS1)	ALK-positive metastatic NSCLC	04/29/2014	P, O
Alectinib HCl (Alecensa)	Roche	ALK inhibitor	NSCLC	12/11/2015	P, O
Brigatinib (Alunbrig)	Takeda	Multitarget TKI (ALK, EGFR, IGF1R, FLT3, and ROS1)	ALK-positive NSCLC	04/28/2017	P, O
Lorlatinib (Lorbrena)	Pfizer	Multitarget TKI (ALK and ROS1)	ALK-positive NSCLC	11/02/2018	P, O
Capmatinib HCl (Tabrecta)	Novartis	MET inhibitor	NSCLC	05/06/2020	P, O
Tepotinib HCl (Tepmetko)	Emd Serono	MET inhibitor	NSCLC	02/03/2021	P, O
Selpercatinib (Retevmo)	Loxo Oncology	RET inhibitor	*RET* fusion-positive NSCLC and thyroid cancer	05/08/2020	P, O
Pralsetinib (Gavreto)	Genentech	RET inhibitor	*RET* fusion-positive NSCLC	09/04/2020	P, O
Entrectinib (Rozlytrek)	Genentech	Multitarget TKI (TRKs, ROS1, and ALK)	*NTRK* fusion-positive solid tumors and ROS1-positive NSCLC	08/15/2019	P, O
Sotorasib (Lumakras)	Amgen	KRAS^G12C^ inhibitor	*KRAS*^G12C^-mutated NSCLC	05/28/2021	P, O
Topotecan HCl (Hycamtin)	Novartis	DNA topoisomerases inhibitor	Relapsed SCLC	05/28/1996	P
Lurbinectedin (Zepzelca)	Jazz	DNA alkylating drug	SCLC	06/15/2020	P, O

*ALK* Anaplastic lymphoma kinase; *EGFR* Epidermal growth factor receptor; *HER2/4* Human epidermal growth factor receptor 2/4; *IGF1R* Insulin-like growth factor-1 receptor; *INSR* Insulin receptor; *NSCLC* Non-small-cell lung cancer; *NTRK* Neurotrophic tyrosine receptor kinase; *O* Orphan; *P* Priority; *RET* Rearranged during transfection; *ROS1* ROS proto-oncogene 1; *S* Standard; *SCLC* Small-cell lung cancer; *TKI* Tyrosine kinase inhibitor; *TRKs* Tropomyosin receptor kinases

Vinorelbine is recommended as an ingredient of systemic therapy regimens for neoadjuvant and adjuvant therapy of NSCLC. As a derivative of *vinca* alkaloid, it binds to tubulin in a complex with the RB3 protein stathmin-like domain (RB3-SLD), heavily overlapping the binding site of vinblastine [10, 11], thereby destabilizing α/β-tubulin heterodimers and leading to mitotic arrest and cell death [12] (Fig. 2b).

*EGFR* mutations occur in approximately 50% of Asian patients and 11 ~ 16% of patients in European countries with NSCLC [13–15]. Exon 19 deletion and exon 21 L858R point mutation make up the majority (> 90%) of all *EGFR* mutation-positive NSCLC [16, 17], which frequently leads to lung tumorigenesis and sensitivity to EGFR-targeted therapies [18]. The FDA has approved six EGFR tyrosine kinase inhibitors (TKIs) which have been the first-line standard of care for patients with NSCLC harboring *EGFR* mutations [19]. These TKIs include the first-generation reversible EGFR inhibitors (gefitinib [20] and erlotinib [21]), the second-generation irreversible EGFR inhibitors (afatinib [22] and dacomitinib [23]), and the third-generation irreversible EGFR inhibitor (osimertinib [24]). First-generation EGFR inhibitors exert their clinical efficacy by targeting the ATP-binding pocket of the kinase domain [20, 21]. However, despite the initial response, patients almost invariably develop primary resistance to gefitinib and erlotinib and relapse after several months [25, 26]. The most common resistance mechanism is associated with the T790M ‘gatekeeper’ mutation at exon 20 of *EGFR* [27], which blocks reversible ATP competitive inhibitors from binding and, in turn, increases ATP binding [28]. Second-generation irreversible EGFR TKIs are highly active against the T790M point mutation of *EGFR* [26, 29] and exert their effect by irreversibly alkylating Cys797 and forming a covalent bond with Cys797 at the ATP-binding pocket [30], thus avoiding the increased ATP affinity conferred by the T790M gatekeeper mutation. However, EGFR T790M shares a similar ATP affinity with wild-type (WT)-EGFR, which limits the ability to achieve plasma concentrations sufficient to inhibit EGFR^T790M^ and results in skin rash and diarrhea in patients, thereby failing to overcome T790M-mediated resistance [31]. The third-generation irreversible EGFR inhibitor osimertinib shares a similar binding mechanism with second-generation irreversible EGFR inhibitors but exhibits lower activity against EGFR^WT^, thereby overcoming the T790M-mediated TKI resistance [32]. As expected, osimertinib significantly prolongs median progression-free survival (PFS) by almost nine months compared with first-generation EGFR inhibitors [24]. However, acquired *EGFR*^C797S^ point mutation-induced impairment in the covalent binding between EGFR^Cys797^ and osimertinib and acquired *MET* amplification induced activation of the bypass pathway [33] lead to resistance to osimertinib [34]. Additionally, necitumumab is a fully human anti-EGFR IgG1κ that binds specifically to EGFR domain III, which overlaps with the EGF binding site, thereby preventing EGF ligands from binding to EGFR [35]. Thus, necitumumab was approved for first-line treatment (in combination with gemcitabine and cisplatin) for patients with metastatic squamous NSCLC [36] (Fig. 2c). Notably, necitumumab binds to most cetuximab- and panitumumab-resistant EGFR variants, such as EGFR^S440L^ and EGFR^S468R^ [37].

*EGFR* exon 20 insertion (*EGFR*^ex20ins^) is clustered between codons 762–775, such as A767_V769dup (V769_D770insASV) and S768_D770dup (D770_N771insSVD) [38, 39]; it represents approximately 6 ~ 12% of *EGFR* mutations in NSCLC cases [40–43] and frequently leads to the constitutive activation of EGFR [38]. Most *EGFR*^ex20ins^ driver mutations in NSCLC are insensitive to first- and second-generation EGFR inhibitors [44–46], except osimertinib, which exhibits partial activity against some *EGFR*^ex20ins^ driver mutations in preclinical studies [39, 45, 47]. However, the clinical trials of osimertinib are inadequate and yield contradictory results [48, 49].

Amivantamab is a bispecific IgG1 that targets both EGFR and MET produced from the two purified bivalent parental antibodies by controlled Fab-arm exchange, each containing single matched point mutations in the CH3 domains (K409R and F405L) [50, 51]. The amivantamab EGFR H-arm shares an epitope identical to that of zalutumumab and binds to EGFR domain III, which overlaps with the EGF binding site, while the MET arm of amivantamab binds to the MET Sema region, which overlaps with the hepatocyte growth factor (HGF) binding site [52]. Amivantamab exhibits antitumor efficiency through the Fc-dependent antibody-dependent cellular cytotoxicity (ADCC) mechanism, Fc-independent EGFR/MET inactivation/degradation and blockade of downstream signaling transduction, and increased interferon-γ (IFNγ) secretion [44, 53, 54]. It yielded robust and durable responses with tolerable safety in patients with *EGFR*^ex20ins^ mutations who progressed on or after platinum-based chemotherapy [55].

Designing a novel EGFR inhibitor is another strategy to address *EGFR*^ex20ins^ mutations. However, the conformation of *EGFR*^ex20ins^ mutants largely resembles that of *EGFR*^WT^ proteins because there are no amino acid substitutions in the binding site [39, 56]. Mobocertinib is an irreversible EGFR inhibitor that is structurally similar to osimertinib. It targets potential structural nuances between the *EGFR*^ex20ins^ and *EGFR*^WT^ proteins in the vicinity of the α C-helix to gain selectivity by binding to the portions of the binding site that are not exploited by osimertinib [39]. Mobocertinib demonstrates greater activity against *EGFR*^ex20ins^ mutants than EGFR^WT^ and more potent efficacy than erlotinib, gefitinib, afatinib, or osimertinib against *EGFR*^ex20ins^ mutants, except *EGFR*^C797S^-containing triple mutants [39, 57]. In subsequent clinical trials, mobocertinib exhibited potent activity with manageable toxicity in patients with advanced previously treated *EGFR*^ex20ins^ NSCLC [58, 59] (Fig. 2d).

Both aberrant ALK expression caused by *ALK* rearrangements [60] and *ALK* amplification are oncogenic driving factors of NSCLC [61]; for example, gene fusion of EMAP-like protein 4 (EML4) and *ALK* induced by *ALK* rearrangements encodes a cytoplasmic chimeric protein with constitutive kinase activity, which accounts for 3 ~ 13% of NSCLC [62, 63]. The FDA has approved five ALK inhibitors, which have been the first-line standard of care for patients with NSCLC harboring *ALK* rearrangements [17], including the first-generation ALK inhibitor (crizotinib [64, 65]), the second-generation ALK inhibitors (ceritinib [66], alectinib [67], and brigatinib [68]), and the third-generation ALK inhibitor (lorlatinib [69]). As with EGFR inhibitors, acquired drug resistance inevitably occurs in most patients after treatment with ALK inhibitors [70, 71]. The mechanisms of ALK inhibitor resistance also involve on-target mechanisms (*e.g.*, *ALK* mutations and amplification) and off-target mechanisms and are even more complicated [72]. Approximately 20 ~ 36% of crizotinib-resistant NSCLCs harbor *ALK* mutations, including 1151Tins, L1152R, C1156Y, I1171T/N/S, L1196M, G1202R, S1206C/Y, E1210K, and G1269A mutations [17, 70–74]. Regarding second-generation ALK inhibitors, *ALK* mutations account for more than half of the instances of resistance [72]. Specifically, 1151Tins, L1152P, C1156Y, F1174C/L/V, and G1202R mutations confer resistance to ceritinib [17, 72, 74], while I1171T/N/S, V1180L, L1196M, and G1202R mutations confer resistance to alectinib [72]. In addition, G1202R, D1203N, S1206Y/C, and E1210K mutations are associated with resistance to brigatinib [17, 72]. Thus, the G1202R mutation is the most common mechanism of first- and second-generation ALK inhibitor resistance. Fortunately, G1202R mutation-induced resistance can be overcome by the third-generation ALK inhibitor lorlatinib [75], which is active against the *EML4*-*ALK*^G1202R^ mutation [76]. Intriguingly, acquired C1156Y and L1198F mutations after lorlatinib treatment resensitize the tumor to crizotinib [69]. However, the off-target mechanisms of ALK inhibitor resistance are still under exploration [77] (Fig. 2e).

Mesenchymal–epithelial transition gene (*MET*) exon 14 skipping mutations and *MET* amplification occur in approximately 3 ~ 4% [78–80] and 1 ~ 6% [81–83] of patients with NSCLC, respectively [84]. *MET* exon 14 skipping mutations produce a truncated MET with a missing regulatory domain that disrupts ubiquitin-mediated degradation, resulting in increased MET levels, sustained MET activation, and oncogenesis [85]. Thus, *MET* exon 14 skipping mutations and *MET* amplification act as oncogenic-driven factors and confer EGFR inhibitor resistance to various cancers, including NSCLC, making it a promising therapeutic target [86]. Capmatinib is a highly selective, reversible type Ib MET inhibitor that targets MET and its mutants (M1250T and Y1235D) [87, 88]. It is more potent than other MET inhibitors (approximately 30 and five times more potent than crizotinib and tepotinib in vitro, respectively) [89]. Capmatinib directly binds to the phenol moiety of the MET^Y1230^ residue, while MET^D1228^ forms a salt bridge with MET^K1110^ to support the Y1230–capmatinib interaction, similar to crizotinib (Type Ia MET inhibitor) [88]. Capmatinib occupies the ATP-binding site of MET, blocks MET phosphorylation, and inhibits MET-mediated downstream signaling activation [88]. Capmatinib exhibits substantial antitumor activity in patients with advanced NSCLC harboring *MET* exon 14 skipping mutations and *MET* amplification [84, 85]. Additionally, capmatinib reverses MET-dependent EGFR inhibitor resistance and blocks the signaling pathway activation mediated by EGFR and HER3 [87]. Significant resistance was observed in cells and clinical NSCLC cases bearing *MET*^D1228^ and *MET*^Y1230^ mutations due to the structural model of the MET–capmatinib interaction [88, 90]. Tepotinib is another selective, reversible type Ib MET inhibitor for a similar clinical setting to capmatinib. Tepotinib shares a similar mechanism with capmatinib in blocking MET [91]. Thus, they achieved equivalent clinical outcomes and adverse events [84, 92, 93]. In vitro, tepotinib overlaps the most *MET* mutation-induced resistance with capmatinib, especially *MET*^Y1230^ mutations, suggesting that tepotinib may not overcome capmatinib resistance [90]. Compared with standard chemotherapy, tepotinib plus gefitinib exhibits improved antitumor activity in patients with *EGFR*-mutant NSCLC with MET overexpression or *MET* amplification [94] (Fig. 2f).

The rearranged during transfection (*RET*) gene rearrangements occur in approximately 1 ~ 2% of patients with NSCLC [95], which is frequently associated with brain metastases [96]. Two selective RET inhibitors (selpercatinib and pralsetinib) were approved as first-line treatments for patients with NSCLC harboring *RET* rearrangements [97, 98]. Selpercatinib and pralsetinib are designed to penetrate the central nervous system (CNS), thereby achieving poor CNS concentrations sufficient to maintain antitumor activity [99]. Both selpercatinib and pralsetinib exhibit activity against acquired *RET*^V804M/L^ gatekeeper resistance mutations [100, 101]. However, *RET*^G810C/S^ solvent front mutations (on-target) and *MET* amplification (off-target) were observed in selpercatinib- and pralsetinib-resistant cases [102–105]. Selpercatinib and pralsetinib bind to the RET kinase in a similar mode that occupies both front and back pockets in the active site clefts without passing through the gate between V804 and K758 into the BP-I pocket [106]. This novel binding mode avoids gatekeeper V804M/L mutation-induced resistance but fails to overcome *RET* mutations in G810 and V738 [106]. New-generation RET inhibitors are needed for this clinical dilemma. Fortunately, selpercatinib plus crizotinib therapy may be an available strategy to overcome selpercatinib resistance in *RET* fusion-positive NSCLC with *MET* amplification [104] (Fig. 2g).

ROS proto-oncogene 1 (*ROS1*) rearrangements occur in approximately 1% of patients with NSCLC [107, 108]. Crizotinib has been the first-line therapy for patients with metastatic *ROS1* fusion-positive NSCLC since 2016 [108, 109]. However, 47% of patients with ROS1-positive NSCLC develop brain metastases upon crizotinib treatment because of crizotinib’s poor CNS penetration due to P-glycoprotein-mediated efflux [110–112]. In addition, the *ROS1*^G2032R^ mutation is frequently observed in NSCLC with acquired resistance to crizotinib [113]. Entrectinib is a multitarget TKI that targets ROS1, tropomyosin receptor kinases (TRKs) (encoded by neurotrophic tyrosine receptor kinase (*NTRK*) genes), and ALK [114]. Compared with crizotinib, entrectinib is a weak substrate of P-glycoprotein that is 30 times more potent against ROS1, thereby overcoming P-glycoprotein-mediated efflux and achieving high CNS concentrations [112, 114, 115]. However, *ROS1*^G2032R^ and *ROS1*^F2004C/I^ mutations are also found in NSCLC with acquired resistance to entrectinib [116]. In addition, more mechanisms of entrectinib resistance are being identified; these include *NTRK1*^G595R^ and *NTRK1*^G667C^ mutations in colorectal cancer [117], *NTRK3*^G623R^ mutation in mammary analog secretory carcinoma (MASC) [118], and insulin-like growth factor-1 receptor (IGF1R) activation and increased P75 expression in neuroblastoma [119]. These findings present new clinical challenges (Fig. 2h).

KRAS, one of the most frequently mutated oncogenes in various cancers, was once considered an undruggable protein due to its small size, relatively smooth surface, and rapid and tight binding properties to GTP in its active state [120]. *KRAS*^G12C^ is an oncogenic driver mutation that occurs in approximately 13% of patients with NSCLC [121]. Sotorasib is the first and only KRAS^G12C^ inhibitor that binds to KRAS^G12C^ via the cysteine residue mutated from the glycine residue, locking KRAS in an inactive state [120, 122, 123]. Sotorasib provides durable clinical benefits in previously treated patients with NSCLC, making it a milestone in cancer therapy [121]. Nevertheless, acquired resistance to sotorasib inevitably occurs via both on- and off-target mechanisms in most patients [124, 125]. G12C/R68S and G12C/Y96C/A double mutants and the G12D mutant of KRAS confer on-target resistance to sotorasib [124]. *MET* amplification is detected in sotorasib-resistant subclonal NSCLC cells with *KRAS*^G12C^ mutation in vitro. Thus, sotorasib plus crizotinib therapy may be a potential strategy to combat off-target resistance [125] (Fig. 2i).

#### Small-cell lung cancer

Small-cell lung cancer (SCLC) is a high-grade neuroendocrine carcinoma with an abysmal prognosis that accounts for approximately 15% of all lung cancer cases [126]. However, only two therapeutic drugs for SCLC have been approved by the FDA over the past 31 years. Both topotecan and lurbinectedin are approved as second-line treatments for patients with recurrent metastatic SCLC. Topotecan and irinotecan are topoisomerase I (TOP1) inhibitors and belong to alkaloid camptothecin derivatives [127]. Topotecan targets TOP1 cleavage complexes (TOP1CCs) by forming a network of hydrogen bonds with Asn722, Arg364, and Asp533 residues of TOP1 at the interface of TOP1CCs [128], thereby forming a physical impediment and blocking transcription elongation [129]. Lurbinectedin is a DNA minor groove covalent binder that binds to selected DNA triplets harboring central guanine (*e.g.*, AGC, CGG, AGG, and TGG), resulting in the formation of a covalent adduct and inhibition of oncogenic transcription [130, 131] (Fig. 2j).

In general, genetic alterations that predict response to treatment account for approximately 30% of patients with NSCLC, including the mutations and/or rearrangements of *EGFR*, *MET*, *BRAF*^V600E^, *ALK*, *ROS1*, *RET*, and *NTRK* [109]. These approved therapeutic drugs, especially the various TKIs, provide significant clinical benefits for patients with lung cancer and other malignancies. However, overcoming the multiple mutations that induced TKI resistance and the off-target effects that induced disease progression remains challenging. As to SCLC, although the comprehensive genomic profiles have been elucidated, the majority of potential targets are undruggable. Seeking efficacious therapeutic targets and novel therapeutic strategies are still the focus of current research on this most deadly human cancer.

### FDA-approved therapeutic drugs for breast cancers

**Fig. 3:**
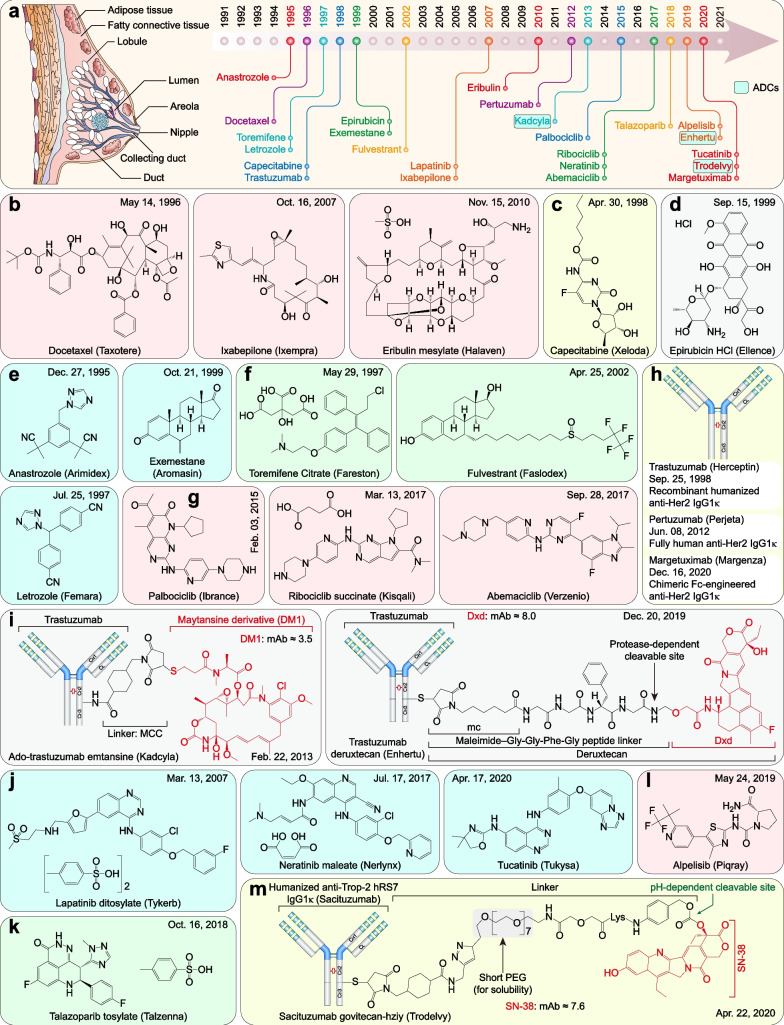
FDA-approved therapeutic drugs for breast cancers. **a** Distribution of therapeutic drugs for breast cancers during the past 31 years (adapted from [863]). **b** Microtubule inhibitors. **c** Antimetabolite. **d** DNA topoisomerase inhibitor. **e** Aromatase inhibitors. **f** ER inhibitors. **g** CDK4/6 inhibitors. **h** HER2-directed mAbs. **i** HER2-directed ADCs. **j** HER2 inhibitors. **k** PARP inhibitor. **l** PI3Kα inhibitor. **m** Trop-2-directed ADC

Breast cancer is common in females (males only account for approximately 1% of breast cancer patients [132]). Breast cancer alone accounted for 24.5% of cancer cases and 15.5% of cancer-related deaths in women and surpassed lung cancer as one of the most commonly diagnosed cancers in 2020 [2]. Over the past 31 years, the FDA granted approvals for 24 new therapeutic drugs (including 18 small molecules, three mAbs, and three ADCs) for breast cancer, more than any other type of solid tumor [133–135] (Fig. 3a and Table 2).

**Table 2:** FDA-approved therapeutic drugs for breast cancers

Drug (brand name)	Sponsor	Properties	Indication	Approval date	Review
Docetaxel (Taxotere)	Sanofi	Microtubule-stabilizing agent	Locally advanced or metastatic breast cancer	05/14/1996	P
Ixabepilone (Ixempra)	R-Pharm US	Microtubule-stabilizing agent	Locally advanced or metastatic breast cancer	10/16/2007	P
Eribulin mesylate (Halaven)	Eisai	Microtubule-destabilizing agent	Locally advanced or metastatic breast cancer	11/15/2010	P
Capecitabine (Xeloda)	Roche	A prodrug of 5-FU	Metastatic breast cancer	04/30/1998	P
Epirubicin HCl (Ellence)	Pfizer	DNA topoisomerase II inhibitor	Primary breast cancer with axillary node tumor involvement	09/15/1999	P, O
Anastrozole (Arimidex)	Ani pharms	Aromatase inhibitor	Advanced breast cancer in postmenopausal women	12/27/1995	S
Letrozole (Femara)	Novartis	Aromatase inhibitor	Advanced breast cancer	07/25/1997	S
Exemestane (Aromasin)	Pfizer	Aromatase inhibitor	ER-positive early breast cancer	10/21/1999	S, O
Toremifene citrate (Fareston)	Kyowa Kirin	ER inhibitor	ER-positive metastatic breast cancer	05/29/1997	S, O
Fulvestrant (Faslodex)	AstraZeneca	ER antagonist	HR-positive metastatic breast cancer	04/25/2002	S
Palbociclib (Ibrance)	Pfizer	CDK4/6 inhibitor	HER2-negative and HR-positive advanced or metastatic breast cancer	02/03/2015	P
Ribociclib succinate (Kisqali)	Novartis	CDK4/6 inhibitor	HR-positive, HER2-negative breast cancer	03/13/2017	P
Abemaciclib (Verzenio)	Eli Lilly	CDK4/6 inhibitor	HR-positive, HER2-negative breast cancer	09/28/2017	P
Trastuzumab (Herceptin)	Genentech	HER2-directed mAb	HER2-positive breast cancer	09/25/1998	P
Pertuzumab (Perjeta)	Genentech	HER2‑directed mAb	HER2‑positive metastatic breast cancer	06/08/2012	P
Margetuximab (Margenza)	MacroGenics	HER2-directed mAb	HER2-positive breast cancer	12/16/2020	S
Ado-trastuzumab emtansine (Kadcyla)	Genentech	HER2‑directed ADC	HER2‑positive metastatic breast cancer	02/22/2013	P
Trastuzumab deruxtecan (Enhertu)	Daiichi Sankyo	HER2-directed ADC	HER2-positive breast cancer	12/20/2019	P
Lapatinib ditosylate (Tykerb)	Novartis	HER2 and EGFR inhibitor	Advanced or metastatic breast cancer	03/13/2007	P
Neratinib maleate (Nerlynx)	Puma Biotech	EGFR, HER2, and HER4 inhibitor	HER2-overexpressed breast cancer	07/17/2017	S
Tucatinib (Tukysa)	Seagen	HER2 inhibitor	HER2-positive breast cancer	04/17/2020	P, O
Talazoparib tosylate (Talzenna)	Pfizer	PARP inhibitor	*BRCA*-mutated HER2-negative breast cancer	10/16/2018	P
Alpelisib (Piqray)	Novartis	PI3Kα inhibitor	*PIK3CA*-altered, HR-positive, and HER2-negative breast cancer	05/24/2019	P
Sacituzumab govitecan (Trodelvy)	Immunomedics	Trop-2-directed ADC	Triple-negative breast cancer (TNBC)	04/22/2020	P

*BRCA*: Breast cancer susceptibility gene; *CDK4/6* Cyclin-dependent kinases 4/6; *ER* Estrogen receptor; *HER2/4* Human epidermal growth factor receptor 2/4; *HR* Hormone receptor; *O* Orphan; *P* Priority; *PARP* Poly (ADP-ribose) polymerase; *PI3Kα* Phosphatidylinositol 3-kinase α; *S* Standard; *TKI* Tyrosine kinase inhibitor

#### Cytotoxic drugs for breast cancer

Cytotoxic drugs are still widely used in clinical practice, especially in systemic chemotherapy for recurrent unresectable (local or regional) human epidermal growth factor receptor 2 (HER2)-negative breast cancer and other malignancies [133]. Among these cytotoxic drugs, docetaxel represents one of the most notable microtubule-stabilizing agents. Docetaxel shares the same taxane binding site of β-tubulin with its analog paclitaxel [136] but shows more potent antitumor activity [137]. It exerts its activity by binding to free β-tubulin and inducing microtubule polymerization, resulting in cell cycle arrest and death [138, 139]. Ixabepilone is a β-lactam analog of epothilone B and is also classified as a microtubule-stabilizing agent. It binds tubulin in a similar but not identical manner to that of paclitaxel and exhibits potent cytotoxic activity in paclitaxel-resistant cells harboring P-glycoprotein expression or mutant tubulin [140]. In contrast, eribulin is a microtubule-destabilizing agent that terminates protofilament elongation by binding predominantly to the vinca domain on β-tubulin, resulting in microtubule catastrophes [141] (Fig. 3b). Capecitabine, a prodrug of 5-fluorouracil (5-FU), is first metabolized to 5′-deoxy-5-fluorouridine (5′DFUR) by carboxylesterase and cytidine deaminase in the liver. 5′DFUR is converted to 5-FU by thymidine phosphorylase (TP) and/or uridine phosphorylase (UP). Given the significantly higher concentrations of both TP and UP in tumor tissues than in normal tissues [142–144], the formation of 5-FU and the subsequent production of active metabolites, including fluorodeoxyuridine monophosphate (FdUMP), fluorouridine triphosphate (FUTP), and fluorodeoxyuridine triphosphate (FdUTP), preferentially occur in tumor tissues [145]. These metabolites finally lead to cell injury by attenuating thymidylate synthase activity (by FdUMP) and incorporating fraudulent bases into RNA (via FUTP) and DNA (via FdUTP) [146] (Fig. 3c). Epirubicin is a 4′-epimer of anthracycline antibiotic doxorubicin that exhibits at least equipotent cytotoxicity but is less myelotoxic than doxorubicin [147]. It binds to topoisomerase IIα (TOP2A), which interferes with helicase activity and TOP2A-DNA cleavable complex formation, resulting in irreversible DNA double-stranded breaks (DSBs) and gene transcription inhibition [148, 149] (Fig. 3d).

#### ER- or HR-positive breast cancer

Hormone receptor (HR)-positive breast cancers, including estrogen receptor (ER)- and/or progesterone receptor (PR)-positive breast cancers, account for more than 70% of all breast cancer cases [150, 151] and lead to approximately 50% of breast cancer-induced deaths [152]. Selective ER modulators (SERMs), such as tamoxifen (brand name: Nolvadex, approved on Nov. 30, 1977, by the FDA), have been the standard of care for patients with ER-positive breast cancer for over 40 years. At present, aromatase inhibitors/inactivators, SERMs, selective ER degrader/down-regulator (SERD), and cyclin-dependent kinases 4/6 (CDK4/6) inhibitors are the first-line standard of care for patients with HR-positive and HER2-negative breast cancers [153].

In premenopausal women, estrogens are mainly synthesized in the ovaries. In postmenopausal women, however, estrogens are synthesized in adipose tissue, breast, and skin, and this process is mediated by aromatase [154]. As a member of the P450 superfamily, aromatase (encoded by *CYP19*) is expressed at extragonadal sites, such as adipose tissue, breast, vascular tissue, bone, brain, and skin, in postmenopausal women [154, 155]. It converts androstenedione and testosterone released from ovaries and adrenal glands to estrone (E1) and E2, respectively [156]. Based on this principle, three third-generation aromatase inhibitors have been developed and approved for postmenopausal women with ER-positive breast cancer [153]. The reversible nonsteroidal aromatase inhibitors anastrozole and letrozole are triazole derivatives that exert clinical efficacy by binding to the heme prosthetic group of aromatase [157, 158]. In contrast, the irreversible aromatase inactivator exemestane binds to the substrate-binding pocket of aromatase, leading to its degradation [159–161]. Among the third-generation aromatase inhibitors, letrozole exhibits the most potent inhibitory effect on aromatase enzyme activity in vivo [159, 162, 163]. It is consistently 10 ~ 30 times more potent than anastrozole in inhibiting intracellular aromatase [164]. Nevertheless, conflicting results exist in various independent studies on clinical efficacy [165–167]. These contradictory results are potentially correlated with the mutation status of GATA binding protein 3 (*GATA3*) [168] or the saturation effect (all third-generation aromatase inhibitors reproducibly cause ~ 98% aromatase inhibition in humans) [161] (Fig. 3e).

Toremifene is a SERM structurally similar to tamoxifen, differing only by a single chlorine atom [169]. Like tamoxifen, toremifene exerts pharmacological activity by competitively inhibiting estradiol (E2) binding to the ER. It thus cannot be used as second-line therapy after tamoxifen failure due to similar pharmacological mechanisms [170]. In contrast, fulvestrant is a full ER antagonist approved as a SERD that overcomes the agonistic effects of tamoxifen and toremifene [171, 172]. However, because of its poor physicochemical features, fulvestrant must be administered monthly intramuscular injections, limiting its clinical application [173]. Mechanistically, it has recently been proven to exert its properties by markedly impairing the intranuclear mobility of the ER [152] (Fig. 3f).

The formation of the cyclin D–cyclin-dependent kinases 4/6 (CDK4/6) complex (also known as G1-CDK) and CDK4/6-induced retinoblastoma (RB) phosphorylation are core events of the G1-S transition in the cell cycle [174]. Inhibition of CDK4/6 induces RB hypophosphorylation and reactivation, resulting in stable cell cycle arrest in the G1 phase [175]. Three CDK4/6 inhibitors (palbociclib, ribociclib, and abemaciclib) have been approved for the first-line therapy of patients with HR-positive and HER2-negative breast cancers in combination with nonsteroidal aromatase inhibitors [176–179] or SERD (fulvestrant) [180, 181] (Fig. 3g), thereby delaying or overcoming endocrine resistance [182]. Although three CDK4/6 inhibitors share multiple similarities, unique characteristics exist in each of them [182]. Palbociclib primarily targets CDK4 monomers instead of endogenous CDK4 trimer complexes or CDK6 but promotes the formation of inactive CDK2 complexes [183]. Palbociclib and ribociclib are more selective for CDK4/6 than abemaciclib, probably due to the greater lipophilicity and larger binding site side chains than abemaciclib, which may reduce the probability of interaction with off-target kinase ATP-binding pockets [184, 185]. Ribociclib is less potent than palbociclib and abemaciclib in inhibiting RB phosphorylation [184]. In contrast, abemaciclib binds to the ATP cleft more readily and forms a hydrogen bond with the conserved catalytic residue (Lys43) of CDKs, which decreases its selectivity [184, 185].

#### HER2-positive breast cancer

HER2-positive breast cancer (including some luminal B subtype cancers) accounts for 13 ~ 15% of all breast cancer cases [186] and is associated with aggressive and metastatic behavior [187]. As the first mAb to be approved to treat solid tumors, trastuzumab is a landmark in tailored therapies [133]. Trastuzumab binds to the extracellular region of HER2 on the C-terminal portion of domain IV and exerts its function via several mechanisms, including ADCC, inhibition of HER2 shedding, and disruption of ligand-independent downstream cascades [188–191]. However, trastuzumab is insufficient to block ligand-induced HER2/HER3 dimerization [191]. In contrast, pertuzumab binds to the extracellular domain II of HER2 and blocks both ligand-dependent and ligand-independent HER2/HER3 dimerization and activation [191–193]. The addition of pertuzumab to the combination of trastuzumab plus docetaxel significantly improves median PFS, and overall survival (OS) compared to that with a pertuzumab-free regimen [194, 195]. Margetuximab, as the latest approved HER2 mAb, improves the ADCC effect in HER2-low tumors with enhanced targeting activity and overcomes trastuzumab resistance [196] (Fig. 3h). Compared with trastuzumab plus chemotherapy, margetuximab plus chemotherapy significantly improves PFS in HER2-positive patients who have received two or more prior anti-HER2 therapies [197].

Trastuzumab significantly improves the clinical outcomes of patients with HER2-positive breast cancer [198]. In the metastatic setting, however, resistance to trastuzumab and disease progression occurs in most patients treated with trastuzumab within one year [199, 200]. The general mechanisms of trastuzumab resistance refer to obstacles for trastuzumab–HER2 interaction, reactivation of HER2 downstream signaling pathways, initiation of bypass signaling pathways, and failure to trigger immune-mediated mechanisms [201]. For this reason, two HER2-based ADCs (ado-trastuzumab emtansine and trastuzumab deruxtecan) were introduced (Fig. 3i). Ado-trastuzumab emtansine is composed of trastuzumab and DM1, linked with a non-cleavable thioether linker, N-succinimidyl-4-(N-maleimidomethyl) cyclohexane-1-carboxylate (SMCC, designated MCC after conjugation) [202, 203]. DM1 is a derivative of maytansine isolated from various *Maytenus* species [204] that exerts antitumor activity by destabilizing microtubules [205]. Ado-trastuzumab emtansine retains all the antitumor efficiency of trastuzumab and is active against lapatinib-resistant breast cancer cells and lapatinib-insensitive tumors [203]. Ado-trastuzumab emtansine shows significant clinical advantages over lapatinib plus capecitabine [206] and trastuzumab plus docetaxel [207]. Despite these therapeutic advances, most patients treated with ado-trastuzumab emtansine eventually experience disease progression [208, 209]. The resistance mechanisms of ado-trastuzumab emtansine partially overlap with those of trastuzumab but also include P-glycoprotein overexpression and receptor-mediated endocytosis defects [210, 211]. Trastuzumab deruxtecan is composed of trastuzumab and TOP1 inhibitor payload (Dxd, an exatecan derivative) linked with a protease-cleavable maleimide tetrapeptide linker [212]. Trastuzumab deruxtecan exhibits durable antitumor activity in patients previously treated with ado-trastuzumab emtansine [213]. Recently, the phase 3 DESTINY-Breast03 trial (NCT03529110) demonstrated that trastuzumab deruxtecan exhibits superiority over trastuzumab emtansine in patients previously treated with the trastuzumab plus taxane regimen [214].

Moreover, three HER2 inhibitors (lapatinib, neratinib, and tucatinib) were approved as third-line regimens for the treatment of HER2-positive breast cancer in combination with trastuzumab and/or capecitabine [215] (Fig. 3j). In contrast to HER2-directed mAbs, HER2 inhibitors bind to the cytoplasmic tyrosine kinase domain instead of the extracellular region of HER2. Lapatinib is a potent dual inhibitor of both EGFR and HER2 [216, 217] that exerts antitumor activity by reversibly binding to the cytoplasmic ATP-binding sites of EGFR and HER2, leading to the impediment of tyrosine kinase phosphorylation, which dampens or abrogates the activation of HER2-mediated downstream pathways [218]. Intriguingly, lapatinib also reverses P-glycoprotein- and ABCG2-mediated multidrug resistance (MDR) by directly attenuating their transport activity [219]. However, *HER2*^T798M/I^ gatekeeper mutations and bypass signaling pathway initiation inevitably confer resistance to lapatinib [220–222]. Neratinib is an irreversible inhibitor of EGFR, HER2, and HER4 that binds to the conserved Cys773 of EGFR and Cys805 of HER2, which forms a covalent bond with the HER family at the cleft of the ATP-binding site [223, 224]. Neratinib exhibited substantial clinical activity in patients with and without prior trastuzumab treatment [225], while the neratinib plus paclitaxel regimen yielded higher complete pathological response rates than the trastuzumab plus paclitaxel regimen in patients with HER2-positive, HR-negative breast cancer [226]. However, the *HER2*^T798I^ gatekeeper mutation also confers resistance to neratinib [227], suggesting that neratinib may not overcome lapatinib resistance, although it displays nanomolar antiproliferative activity against this mutant in vitro [228]. Tucatinib is another reversible HER2 inhibitor that shares a similar binding mechanism with lapatinib but exhibits the highest selectivity to HER2 among these HER2 inhibitors [229]. Compared to placebo, tucatinib’s addition to the trastuzumab plus capecitabine regimen exhibited acceptable toxicity [230], improved survival outcomes, improved objective response rate (ORR), and reduced the risk of death [231, 232]. Among these HER2 inhibitors, neratinib exhibits the most potent activity against HER2 kinase, followed by tucatinib and lapatinib [228].

#### BRCA-mutated breast cancer

Germline mutations of *BRCA1* and/or *BRCA2* are observed in more than 5% of all breast cancer cases and approximately 13% of basal-like breast cancer (BLBC) cases [233]. *BRCA1/2* mutations frequently indicate a deficiency in repairing DNA DSBs by homologous recombination [234] and predispose patients to breast, ovarian, and other cancers [235–237]. Poly (ADP-ribose) polymerases (PARPs) are essential for DNA single-strand break (SSB) repair by base excision repair (BER) [238]. The N-terminal zinc finger motifs of PARPs bind to damaged DNA, which activates its catalytic C-terminal to hydrolyze nicotinamide adenine dinucleotide (NAD^+^) and produce ADP-ribose units, thereby yielding linear and branched poly (ADP-ribose) (PAR) for the resealing of DNA SSBs during BER [239, 240]. PARP inhibitors are designed to inhibit auto-PARylation by competitively binding to PARPs at the NAD^+^ binding site [241, 242], leading to cell death in *BRCA1/2*-mutated cancer cells through a synthetic lethality mechanism [243]. Breast cancers harboring germline mutations in either *BRCA1* or *BRCA2* are highly sensitive to PARP inhibitors [244, 245], and thus, inhibiting PARPs has become a therapeutic strategy for targeting *BRCA1/2*-mutated cancer cells [246]. Talazoparib is the fourth (also the latest) PARP inhibitor approved by the FDA (after olaparib, rucaparib, and niraparib) [247]. Through hydrogen-bonding and π-stacking interactions, including those mediated by active site water molecules, talazoparib is anchored to the nicotinamide-binding pocket [248], leading to a noticeable displacement of the bound ligand within the NAD^+^ site [249]. Compared with olaparib, rucaparib, and niraparib (IC_50_ values 1.94, 1.98, and 3.8 nM, respectively, for the inhibition of PARP1), talazoparib is three times more potent, with an IC_50_ of 0.57 nM [250, 251]. Therefore, talazoparib exhibits superiority over olaparib or rucaparib in trapping PARP–DNA at the site of DNA damage [239]. Talazoparib monotherapy demonstrates a tolerable safety profile and preliminary clinical activity in patients with sporadic cancers harboring germline *BRCA1/2* mutations [252]. It also exhibits a significant benefit over standard chemotherapy (capecitabine, eribulin, gemcitabine, or vinorelbine) among patients with germline *BRCA1/2*-mutated breast cancer [253] (Fig. 3k).

#### PIK3CA-altered breast cancer

Phosphatidylinositol 3-kinase catalytic subunit A (*PIK3CA*) gene mutation is observed in approximately 40% of HR-positive and HER2-negative breast cancers [233, 254, 255]. *PIK3CA* mutation induces phosphatidylinositol 3-kinase (PI3K) activation, leading to cell proliferation and apoptosis evasion [233]. Alpelisib is a PI3Kα inhibitor that binds to PI3Kα and forms multiple hydrogen bonds with PI3Kα at the ATP-binding pocket, thereby inhibiting the enzymatic activity of PI3Kα and PI3Kα-mediated downstream pathways [256, 257]. Alpelisib demonstrates tolerable safety and favorable clinical efficiency in patients with *PIK3CA*-altered, HR-positive, HER2-negative breast cancer in combination with fulvestrant [255, 258–260] or letrozole [261] (Fig. 3l).

#### Triple-negative breast cancer

Triple-negative breast cancer (TNBC) is defined as breast cancer lacking expression of ER, PR, and HER2, which accounts for 10 ~ 15% of all breast cancer cases [186]. It represents the subtype with the worst prognostic outcome among breast cancers [262]. Before 2019, single-agent taxanes or anthracyclines were the first-line regimens for unresectable locally advanced or metastatic TNBC [263]. However, the median OS remains at approximately 18 months or even less [264, 265]. On March 8, 2019, the FDA-approved atezolizumab plus albumin-bound nab-paclitaxel (brand name: Abraxane, approved on January 7, 2005) as a first-line regimen for unresectable locally advanced or metastatic TNBC with PDL1 expression [265, 266]. Trophoblastic cell surface antigen-2 (Trop-2, also known as EGP-1, encoded by *TACSTD2*) is a transmembrane glycoprotein overexpressed in 83% of breast cancer cases [267] and 85% of TNBCs [268]. It is considered a key driver of human cancers, making it an attractive target for TNBC treatment [267]. Sacituzumab govitecan-hziy is a Trop-2-directed ADC composed of sacituzumab and SN-38 covalently linked with a hydrolyzable CL2A linker [269, 270]. Safituzumab is a humanized Trop-2-directed mAb developed from murine RS7-3G11 [271, 272], while SN-38 is an active metabolite of irinotecan, a TOP1 inhibitor [273]. Sacituzumab govitecan-hziy exhibits acceptable toxicity and preliminary clinical activity in previously treated patients with refractory metastatic solid tumors [274], especially with metastatic TNBC [275, 276]. Compared with standard chemotherapy, it demonstrates durable objective responses and significant superiorities in heavily treated patients with metastatic TNBC [277]. However, the clinical benefits of sacituzumab govitecan-hziy are highly dependent on Trop-2 expression; definitive conclusions are difficult to draw in the low Trop-2 expression subgroup [278]. In addition, canonical *TOP1*^E418K^ resistance mutation, *TOP1*p.-122 fs (frameshift mutation), and *TACSTD2*^T256R^ missense mutation confer resistance to sacituzumab govitecan-hziy [268]. These findings pose new challenges regarding sacituzumab govitecan-hziy application (Fig. 3m).

Breast cancer drugs are frequently at the forefront of advances in cancer treatment and diagnosis, especially in CDK4/6 inhibitors, HER2 inhibitors, and HER2-directed mAbs and ADCs [133]. Meanwhile, the progress of breast cancer drugs provides an essential basis for other malignancies in drug research and development. Cytotoxic drugs and selective ER antagonists dominated the early decades until 2010. However, these two types of medicines have been overshadowed by targeted drugs, which have accounted for the majority of the newly approved breast cancer drugs since 2010. Besides, the approved drugs mainly focused on targeting HER2 in recent years, limiting the breakthrough in drug development, especially for TNBCs.

### FDA-approved therapeutic drugs for gynecologic cancers

**Fig. 4:**
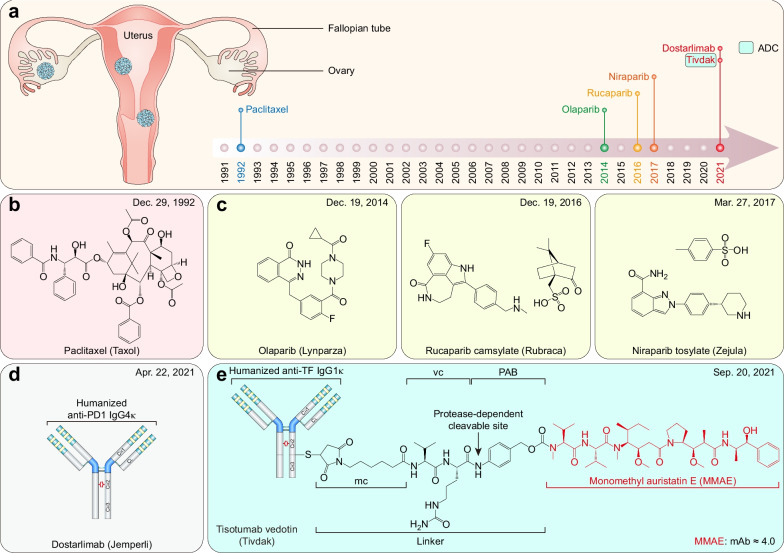
FDA-approved therapeutic drugs for gynecologic cancers. **a** Distribution of therapeutic drugs for gynecologic cancers during the past 31 years (adapted from [864]). **b** Microtubule inhibitor. **c** PARP inhibitors. **d** PD1-directed mAb. **e** TF-targeted ADC

Gynecologic cancers include cervical, ovarian, uterine, vaginal, vulvar, and fallopian tube cancers, accounting for 15.2% of all malignancies among females and 15.3% of cancer-related deaths worldwide in 2020 [2]. However, only six therapeutic drugs have been approved by the FDA for gynecologic cancers as initial indications since 1991 (Fig. 4a and Table 3).

**Table 3:** FDA-approved therapeutic drugs for gynecologic cancers

Drug (brand name)	Sponsor	Properties	Indication	Approval date	Review
Paclitaxel (Taxol)	HQ Spclt	Microtubule-stabilizing agent	Advanced ovarian cancer	12/29/1992	P, O
Olaparib (Lynparza)	AstraZeneca	PARP inhibitor	Advanced *BRCA*-mutated ovarian cancer	12/19/2014	P, O
Rucaparib camsylate (Rubraca)	Clovis Oncology	PARP inhibitor	*BRCA*-positive ovarian cancer	12/19/2016	P, O
Niraparib tosylate (Zejula)	GlaxoSmithKline	PARP inhibitor	Epithelial ovarian, fallopian tube, or primary peritoneal cancer	03/27/2017	P, O
Dostarlimab (Jemperli)	GlaxoSmithKline	PD1-directed mAb	Endometrial cancer	04/22/2021	P
Tisotumab vedotin (Tivdak)	Seagen	TF-targeted ADC	Cervical cancer	09/20/2021	P, O

*O* Orphan; *P* Priority; *PARP* Poly (ADP-ribose) polymerase; *PD1* Programmed death receptor-1; *S* Standard; *TF* Tissue factor

#### Ovarian cancer

Ovarian cancer is the third most common gynecologic cancer, accounting for 3.4% of all female malignancies and 4.7% of cancer-related deaths in females worldwide in 2020 [2]. FDA has granted four new therapeutic drug approvals for ovarian cancer. Paclitaxel is undoubtedly a milestone in the history of cancer drugs. It was isolated by Wall and Wani from the bark of *Taxus brevifolia* in 1971 [279] (Fig. 4b). Currently, paclitaxel (including nab-paclitaxel albumin-bound) and its analog docetaxel are widely used to treat various malignancies [280]. It covalently binds to β-tubulin at amino acid residues 1–31 [281], 217–233 [282], and Arg282 [283] and enhances microtubule polymerization, thereby suppressing microtubule dynamics and blocking cell mitosis [284].

Germline mutations of *BRCA1* and/or *BRCA*/*2* are present in approximately 14.1% of all ovarian cancer cases [285]. Based on the same principle described in the breast cancer section above, three PARP inhibitors (olaparib [286], rucaparib [287], and niraparib [250]) were approved as maintenance therapies for *BRCA1/2*-mutated ovarian cancer [288–290] (Fig. 4c). These PARP inhibitors are designed to competitively bind to the NAD^+^ binding site of the PARP enzyme [237, 238]. Platinum and PARP inhibitor sensitivity commonly coexist in *BRCA1/2*-mutated ovarian cancer due to homologous recombination deficiency (HRD) [291]; however, nucleotide excision repair (NER) alterations confer enhanced platinum sensitivity but not PARP inhibitor sensitivity [292]. There is no significant efficacy difference between these PARP inhibitors as maintenance therapies in patients with *BRCA*-mutated, platinum-sensitive relapsed ovarian cancer [293]. Additionally, PARP inhibitors yield similar response and survival rates in patients harboring either somatic or germline *BRCA* mutations [294]. Of note, olaparib represents the most cost-effective [295] PARP inhibitor, and the olaparib plus bevacizumab regimen achieved a dramatic improvement in PFS in ovarian cancer patients with *BRCA* mutations (37.2 months) and without *BRCA* mutations (28.1 months) compared to that with placebo plus bevacizumab (17.7 and 16.6 months, respectively) [296]. Thus, the olaparib plus bevacizumab regimen was approved for first‐line maintenance treatment of HRD-positive advanced ovarian cancer [297]. Clinical trials of PARP inhibitors (rucaparib and niraparib) combined with bevacizumab for ovarian cancer maintenance therapy are still ongoing [298, 299].

#### Endometrial cancer

Endometrial cancer is the second most common gynecologic cancer and originates in the inner epithelial lining of the uterus [300]. It accounted for 4.5% of all malignancies among females and 2.2% of cancer-related deaths in females worldwide in 2020 [2]. Mismatch repair deficiency (dMMR) is a consequence of germline mutations or epigenetic silencing in *MMR* genes, resulting in the accumulation of errors introduced during DNA replication [301]. Therefore, dMMR leads to genome-wide instability, especially in regions of simple repetitive DNA sequences (known as microsatellite instability—high (MSI-H)), resulting in tumorigenesis [302]. MSI-H/dMMR is observed in 18 ~ 28% of endometrial cancer cases [303, 304] and confers sensitivity to PD1 blockade [304]. A higher number of CD3^+^ and CD8^+^ TILs and increased PD1 expression (but not PDL1) are observed in the hypermutated subgroups (*POLE* mutations or MSI-H/dMMR) of endometrial cancer than in the hypomutated microsatellite-stable subgroup [305], explaining why MSI-H/dMMR-positive endometrial cancer is sensitive to PD1 blockade. Dostarlimab is the fourth (also the latest) FDA-approved PD1-directed mAb after pembrolizumab, nivolumab, and cemiplimab [306]. It exhibits a high affinity for both human and cynomolgus monkey PD1, preventing PDL1 and PDL2 from interacting with PD1 [307]. Dostarlimab demonstrates a manageable safety profile equivalent to that of other PD1-directed mAbs and robust clinical activity in previously treated patients with recurrent or advanced MSI-H/dMMR or MMR proficient/stable (MMRp/MSS) endometrial cancer [308, 309] (Fig. 4d). Of note, dostarlimab achieved a complete response in 100% of patients with dMMR-positive locally advanced rectal cancer [310].

#### Cervical cancer

Cervical cancer is the most common gynecologic cancer, accounting for 6.6% of all malignancies among females and 7.8% of cancer-related deaths in females worldwide in 2020 [2]. Cervical cancer is strongly linked with human-papillomavirus (HPV) infection [311], especially HPV-16 and HPV-18 subtypes [312]. Tissue factor (TF, also known as thromboplastin, factor III, or CD142) is overexpressed in various cancers [313], especially cervical cancer [314]. TF promotes tumor progression by initiating the coagulation pathway with its procoagulant activity and protease-activated receptor 2 (PAR-2)-mediated signaling, making it an attractive target [315]. Tisotumab vedotin is a TF-directed ADC composed of tisotumab and microtubule-destabilizing agent monomethyl auristatin E (MMAE), linked with protease-cleavable maleimidocaproyl valine-citrulline *p*-aminobenzyl alcohol carbamate (MC-vc-PAB) linker [316]. Tisotumab is a TF-directed mAb generated by immunization of HuMAb mice [316], while vedotin refers to MMAE plus the MC-vc-PAB linker. Tisotumab vedotin demonstrates a manageable safety profile and durable antitumor activity in previously treated (*e.g.*, bevacizumab plus doublet chemotherapy) patients with recurrent or metastatic cervical cancer [317, 318] (Fig. 4e).

### FDA-approved therapeutic drugs for gastrointestinal cancers

**Fig. 5:**
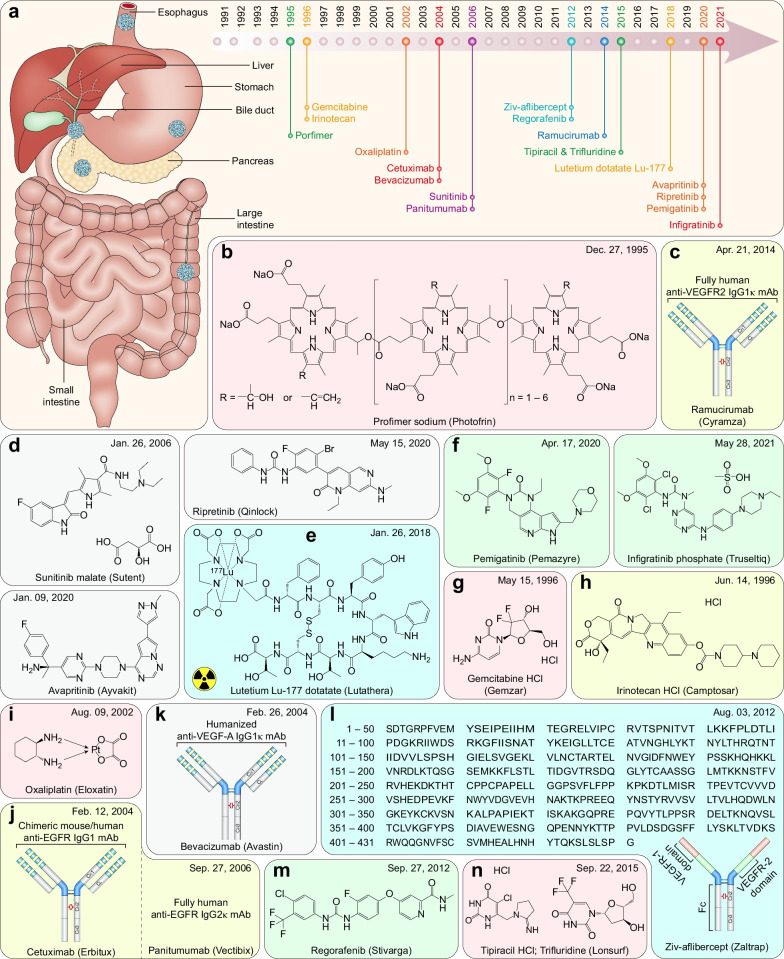
FDA-approved therapeutic drugs for gastrointestinal cancers. **a** Distribution of therapeutic drugs for gastrointestinal cancers during the past 31 years (adapted from [865, 866]). **b** Photosensitizer. **c** VEGFR2-directed mAb. **d** Multitarget TKI and PDGFR inhibitors. **e** Somatostatin receptor-targeted radiopharmaceutical. **f** FGFR inhibitors. **g** DNA synthesis inhibitor. **h** DNA topoisomerase inhibitor. **i** Organoplatinum alkylating agent. **j** EGFR‑directed mAb. **k** VEGF‑A-directed mAb. **l** Soluble receptor decoy that binds VEGF-A, VEGF-B, and PlGF. **m** Multitarget TKI. **n** Thymidine phosphorylase inhibitor plus nucleoside metabolic inhibitor

Gastrointestinal cancers include esophageal, gastric, colorectal, pancreatic, gallbladder, and liver cancer (including cholangiocarcinoma), accounting for 26.4% of cancer cases and 36.3% of cancer-related mortalities worldwide in 2020 [2]. Over the past 31 years, the FDA granted approvals for 17 new therapeutic drugs (including 12 small molecules, four mAbs, and one recombinant fusion protein) for gastrointestinal cancers (Fig. 5a and Table 4).

**Table 4:** FDA-approved therapeutic drugs for gastrointestinal cancers

Drug (brand name)	Sponsor	Properties	Indication	Approval date	Review
Porfimer sodium (Photofrin)	Pinnacle Biolgs	A photosensitizer used for photodynamic therapy	Obstructing esophageal cancer	12/27/1995	P, O
Ramucirumab (Cyramza)	Eli Lilly	VEGFR2-directed mAb	Gastric cancer	04/21/2014	P, O
Sunitinib malate (Sutent)	CPPI CV	Multitarget TKI (VEGFRs, PDGFRα/β, CSF1R, KIT, and FLT3)	Imatinib-resistant GIST and advanced RCC	01/26/2006	P
Avapritinib (Ayvakit)	Blueprint	PDGFRα, PDGFRα mutants, and KIT inhibitor	GIST with *PDGFRA* exon 18 mutations	01/09/2020	P, O
Ripretinib (Qinlock)	Deciphera	PDGFRα, PDGFRα mutants, and KIT inhibitor	Advanced GIST	05/15/2020	P, O
Lutetium Lu-177 dotatate (Lutathera)	AAA USA	Somatostatin receptor-targeted radiopharmaceutical	GEP-NETs	01/26/2018	P, O
Pemigatinib (Pemazyre)	Incyte	FGFR1-3 inhibitor	Advanced cholangiocarcinoma with *FGFR2* fusions/rearrangements	04/17/2020	P, O
Infigratinib phosphate (Truseltiq)	Helsinn Hlthcare	FGFR1-3 inhibitor	Advanced cholangiocarcinoma with *FGFR2* fusions/rearrangements	05/28/2021	P, O
Gemcitabine HCl (Gemzar)	Eli Lilly	DNA synthesis inhibitor	Locally advanced or metastatic pancreatic cancer	05/15/1996	P
Irinotecan HCl (Camptosar)	Pfizer	DNA topoisomerase I inhibitor	Metastatic colorectal cancer	06/14/1996	P
Oxaliplatin (Eloxatin)	Sanofi	Organoplatinum alkylating agent	Colorectal cancer (in combination with 5-FU and leucovorin)	08/09/2002	P
Cetuximab (Erbitux)	ImClone	EGFR‑directed mAb	Colorectal cancer	02/12/2004	N/A
Panitumumab (Vectibix)	Amgen	EGFR‑directed mAb	Colorectal cancer	09/27/2006	N/A
Bevacizumab (Avastin)	Genentech	VEGF‑A-directed mAb	Colorectal cancer	02/26/2004	O
Ziv-aflibercept (Zaltrap)	Sanofi	Soluble receptor decoy that binds VEGF-A, VEGF-B, and PlGF	Metastatic colorectal cancer	08/03/2012	N/A
Regorafenib (Stivarga)	Bayer	Multitarget TKI (RET, VEGFRs, KIT, PDGFRα/β, FGFR1/2, RAF1, BRAF, and BRAF^V600E^)	Metastatic colorectal cancer	09/27/2012	P
Tipiracil HCl; Trifluridine (Lonsurf)	Taiho	Thymidine phosphorylase inhibitor plus a nucleoside metabolic inhibitor	Colorectal cancer	09/22/2015	S

*EGFR* Epidermal growth factor receptor; *FGFR1-3* Fibroblast growth factor receptor-1–3; *GEP-NET* Gastroenteropancreatic neuroendocrine tumor; *GIST* Gastrointestinal stromal tumor; *O* Orphan; *P* Priority; *PDGFRα/β* Platelet-derived growth factor receptor *α*/*β*; *PlGF* Placenta growth factor; *RCC* Renal cell carcinoma; *RET* Rearranged during transfection; *S* Standard; *VEGF* Vascular endothelial growth factor; *VEGFR* Vascular endothelial growth factor receptor

#### Esophageal cancer

Esophageal cancer accounted for 3.1% of cancer cases and 5.5% of cancer-related mortalities worldwide in 2020 [2]. Porfimer sodium was approved by the FDA as a photosensitizer for photodynamic therapy of obstructing esophageal cancer [319] (Fig. 5b). In the presence of oxygen, this approach utilizes light to activate the porfimer sodium, which is relatively selectively concentrated in cancer cells, leading to cell death [320]. However, photodynamic therapy with porfimer sodium as an endoscopic therapy for esophageal cancer is losing popularity due to the potential for long-term complications [321]. Fluoropyrimidine plus platinum-based chemotherapies are frequently used as first-line therapy for advanced esophageal cancer [322]. Compared to chemotherapy alone, pembrolizumab plus 5-FU and cisplatin (chemotherapy) significantly improve clinical outcomes in the first-line treatment of advanced esophageal cancer [323].

#### Gastric cancer

Gastric cancer accounted for 5.6% of cancer cases and 7.7% of cancer-related mortalities worldwide in 2020 [2]. Vascular endothelial growth factor receptor 2 (VEGFR2), the principal receptor of VEGF-induced angiogenesis, is expressed in most solid tumors, including gastric cancer [324]. Ramucirumab is a VEGFR2-directed mAb [325] that binds selectively to the g-like extracellular domain III of VEGFR2, which prevents VEGF ligands from binding to VEGFR2 [326], thereby inhibiting VEGF ligand-induced cell proliferation, migration, and angiogenesis [327] (Fig. 5c). Ramucirumab monotherapy exhibits significant survival benefits in patients with advanced gastric or gastroesophageal junction adenocarcinoma who have disease progression after first-line chemotherapy compared to placebo [328]. The ramucirumab plus paclitaxel regimen also demonstrated superiority over placebo plus paclitaxel therapy in the same clinical setting; thus, ramucirumab plus paclitaxel could be regarded as a new second-line treatment for advanced gastric cancer [329].

#### Gastrointestinal stromal tumors

Gastrointestinal stromal tumors (GISTs) constitute the largest subset of mesenchymal tumors that arise from precursors of the connective tissue cells of the gastrointestinal tract [330, 331]. They occur predominantly (60%) in the stomach, with 30% of cases in the small intestine and 10% of cases in other sites of the gastrointestinal tract; 10 ~ 30% are malignant and exhibit intra-abdominal spread or liver metastases [332]. RTKs, such as VEGFR2, platelet-derived growth factor receptor α/β (PDGFRα/β), and KIT, are frequently overexpressed or mutated in GISTs, leading to constitutive activation of these kinases [333, 334]. Approximately 75 ~ 80% of GISTs harbor *KIT* mutations, and 5 ~ 8% of GISTs harbor *PDGFRA* mutations [334]. Therefore, the FDA-approved three multitarget TKIs for GIST treatment (Fig. 5d).

Imatinib (Additional file 1: Table S1, page 13; Table S2, page 44) is still the first-line treatment for advanced GISTs [335, 336]. However, approximately 50% of patients develop resistance within two years [336, 337]. Sunitinib is a potent inhibitor of multiple RTKs, including PDGFRα/β, VEGFR2, and KIT [338], and has been approved as second-line therapy for imatinib-resistant GISTs [339, 340]. The ATP-binding-pocket mutants KIT^V654A^, KIT^T670I^, and PDGFRα^D842V^ are the most common in imatinib-resistant GISTs, whereas certain mutant-induced resistance can be overcome by sunitinib, except PDGFRα^D842V^ [337, 340, 341]. Given the failures in overcoming the *PDGFRA*^D842V^-induced resistance, avapritinib was approved as a first-line regimen for GISTs harboring *PDGFRA* exon 18 (including D842V) mutation [342]. Avapritinib is a potent TKI that targets *KIT* exon 17 (including D816V) and *PDGFRA* exon 18 (including D842V) mutations. In contrast, imatinib, sunitinib, and regorafenib exhibit weak potency in blocking mutation-induced constitutive kinase activity [343, 344]. Given the heterogeneity of *KIT* and *PDGFRA* mutants in GISTs, broader spectrum drugs are needed to overcome the multiple mutations of *KIT* and *PDGFRA*, as well as other RTKs. Ripretinib was designed to overcome the drug resistance of GISTs harboring broad *KIT* and *PDGFRA* mutations [345]. As a ‘switch control’ kinase inhibitor, ripretinib forces the activation loop of KIT or PDGFRα into an inactive conformation through a switch control mechanism that prevents switches from adopting a type I active state and stabilizes switches in type II inactive state [345, 346]. Therefore, the FDA-approved ripretinib for the fourth-line treatment of patients with advanced GIST who have received prior treatment with three or more TKIs [345]. Notably, the common PDGFRα^D842V^ mutant is sensitive to avapritinib and crenolanib but resistant to ripretinib, and secondary resistance mutations after imatinib or avapritinib treatment, such as the triple mutant PDGFRα^D842V/V658A/G652E^, can be overcome by the heat shock protein 90 (HSP90) inhibitor tanespimycin [347].

#### Gastroenteropancreatic neuroendocrine tumors

Gastroenteropancreatic neuroendocrine tumors (GEP-NETs) account for more than 60% of NETs that arise from neuroendocrine cells of the digestive tract [348]. Regarding prevalence, GEP-NETs have been the second most common gastrointestinal cancer [349]. Somatostatin receptors (SSTRs) are G-protein-coupled receptors frequently expressed in GEP-NETs [350]. Somatostatin is the ligand of SSTRs that inhibits the release of pituitary and gastrointestinal hormones [351]. Octreotide (brand name: Sandostatin, approved by the FDA on Oct. 21, 1988), a synthetic octapeptide (D-Phe-c[Cys-Phe-D-Trp-Lys-Thr-Cys]-Thr-ol), is a somatostatin analog with long-acting pharmacologic properties mimicking natural somatostatin [352]. Therefore, it has been approved for metastatic carcinoid and vasoactive intestinal peptide-secreting tumors [353]. While it does not affect tumor progression, it can improve symptoms. On the other hand, Lutetium-177 (^177^Lu) is a medium-energy β- and low-energy γ-emitting radionuclide with a maximal tissue penetration of 2 mm [354] and a half-life of 160 h [355], allowing detection by scintigraphy and subsequent dosimetry. Combining the properties of ^177^Lu and octreotate (differs from octreotide only in that the C-terminal threoninol is replaced with threonine but exhibits a higher affinity for SSTR2 than octreotide [356]), [^177^Lu-DOTA^0^,Tyr^3^]-octreotate (Lutetium Lu-177 dotatate) was approved by the FDA for peptide receptor radionuclide therapy (PRRT) of SSTR-positive advanced GEP-NETs [349] (Fig. 5e).

#### Cholangiocarcinoma

Hepatocellular carcinoma (HCC, comprising 75% ~ 85% of liver cancer cases) and intrahepatic cholangiocarcinoma (ICC, comprising 10 ~ 15% of liver cancer cases) are the most frequent types of primary liver cancer, which accounted for 4.7% of cancer cases and 8.3% of cancer-related mortalities worldwide in 2020 [2]. Compared to HCC, ICC has a poorer prognosis in terms of both mOS (HCC 71.7 months vs. ICC 21.5 months) and disease-free survival (DFS) (HCC 68.2 months vs. ICC 15.5 months) [357]. Genomic alterations (including mutation, fusion, and rearrangement) that activate fibroblast growth factor receptor 2 (FGFR2) are almost exclusively found in patients with ICC, making it a promising therapeutic target [358, 359].

Pemigatinib is a potent, selective inhibitor of FGFR1-3 that binds the ATP-binding pocket of FGFR at the hinge region, thereby inhibiting FGFR-mediated cell proliferation, differentiation, and angiogenesis [360, 361]. Pemigatinib exhibits a manageable safety profile and durable antitumor activity in previously treated patients with cholangiocarcinoma harboring *FGFR2* fusions/rearrangements [362]. A phase 3 FIGHT-302 clinical trial of first-line pemigatinib vs. gemcitabine plus cisplatin for advanced cholangiocarcinoma harboring *FGFR2* fusions/rearrangements is still ongoing [363]. Similar to all other TKIs, acquired resistance mutations in *FGFR2* (N549K/H, E565A, L617V, K641R, and K659M) are observed in patients with progressive disease and may confer resistance to pemigatinib [359]. Infigratinib is another FGFR1-3 inhibitor [364] that binds to FGFR at a hinge region, similar to pemigatinib [365]. It shows manageable toxicity and meaningful clinical activity against chemotherapy-refractory cholangiocarcinoma *FGFR2* fusions/rearrangements [366, 367]. Strikingly, 5 of 6 *FGFR2* mutations observed in infigratinib-resistant patients completely overlapped with the five *FGFR2* mutations observed in pemigatinib-resistant cases, except for the *FGFR2*^V564F^ gatekeeper resistance mutation, which exclusively exists in infigratinib-resistant patients [359, 368, 369]. Thus, pemigatinib may theoretically overcome *FGFR2*^V564F^ mutation-induced resistance to infigratinib (Fig. 5f).

#### Pancreatic cancer

Pancreatic cancer has the highest mortality-to-incidence ratio (1.808) among all malignancies, accounting for 2.6% of cancer cases and 4.7% of cancer-related mortalities worldwide in 2020 [2]. Although genomic and microenvironment alterations of pancreatic cancer have been elucidated [370, 371], most alterations (*e.g.*, *KRAS* and *TP53* mutations) are not druggable. Given the lack of effective targets, systemic chemotherapy is still the first-line regimen. Gemcitabine is an analog of deoxycytidine that acts as a DNA synthesis inhibitor. It is phosphorylated by deoxycytidine kinase to form its active products (including gemcitabine diphosphate and gemcitabine triphosphate), which are incorporated into the DNA, leading to the inhibition of the DNA synthesis process [372] (Fig. 5g). Gemcitabine exhibits significant superiority over 5-FU in patients with advanced pancreatic cancer [373]. Currently, systemic chemotherapy combinations, including FOLFIRINOX (5-FU, leucovorin, irinotecan, and oxaliplatin) [374] and gemcitabine plus nab-paclitaxel [375], have become the first-line treatment for patients with advanced pancreatic cancer [376]. There was no significant difference in the treatment efficacy between the FOLFIRINOX and gemcitabine plus nab-paclitaxel regimens [377].

#### Colorectal cancer

Colorectal cancer accounted for 9.8% of cancer cases and 9.2% of cancer-related mortalities worldwide in 2020 [2]. The FDA-approved eight therapeutic drugs in the past 31 years (Fig. 5h–n). Similar to topotecan, irinotecan is also a TOP1 inhibitor (Fig. 5h). However, compared to topotecan, irinotecan is a prodrug. It is hydrolyzed by uridine diphosphate glucuronosyltransferase 1A1 (UGT1A1) to its active metabolite SN-38 by carboxylesterases, which are abundant in plasma, liver, and cancer cells [273, 378, 379]. A study indicated that SN-38 is 1000 times more potent than irinotecan in inducing DNA SSBs [378]. Thus, SN-38 was adopted as a cytotoxic agent in a Trop-2-directed ADC. Oxaliplatin is a third-generation platinum-based drug that impairs normal DNA functions by generating mono-adducts and DNA crosslinks, similar to the first- (cisplatin) and second-generation (carboplatin) platinum drugs [380] (Fig. 5i). In contrast to cisplatin and carboplatin, oxaliplatin has a unique indication for colorectal cancer, as it facilitates organic cation transporter (OCT)-mediated uptake [381]. In addition, oxaliplatin shows different drug resistance mechanisms from cisplatin and carboplatin. Specifically, dMMR and replicative bypass increases that confer cisplatin resistance do not contribute to resistance to oxaliplatin [382]. On the other hand, multidrug resistance-associated protein 2 (MRP2)-mediated drug efflux limits both cisplatin and oxaliplatin accumulation [383, 384], rendering gastrointestinal cancer cells resistant to oxaliplatin [385] but not to cisplatin [381, 386]. Given the broad-spectrum antitumor activity of irinotecan and oxaliplatin, they have become essential ingredients in some classical regimens for colorectal cancer treatment, such as FOLFOXIRI (5-FU, leucovorin, oxaliplatin, and irinotecan), FOLFIRI (5-FU, leucovorin, and irinotecan) [387], FOLFOX (5-FU, leucovorin, oxaliplatin) [388], and CAPEOX (capecitabine and oxaliplatin) [389].

Similar to NSCLC, EGFR is overexpressed in approximately 50 ~ 80% of colorectal cancers [390, 391]. However, somatic mutations of EGFR occur at a very low frequency in colorectal cancer [392]. Thus, two EGFR‑directed mAbs (cetuximab and panitumumab) were approved by the FDA for EGFR-positive metastatic colorectal cancer (Fig. 5j). Cetuximab interacts exclusively with the soluble extracellular region of EGFR and occludes the ligand-binding region on domain III of EGFR partially, which sterically prevents EGFR from adopting the extended conformation required for dimerization, thereby inhibiting the activation of EGFR [393]. Colorectal cancer harboring *EGFR*^S492R^, *EGFR*^K467T^, and *EGFR*^R451C^ mutations confer cetuximab resistance but respond to panitumumab [394, 395]. These mutations may directly block cetuximab binding to domain III of EGFR but are permissive for panitumumab binding, which is attributed to a central cavity located between the heavy and light chain of panitumumab accommodating these mutations [396]. Given the low incidence of *EGFR* mutations, cetuximab and panitumumab are considered equivalent treatments in most clinical circumstances due to a shared epitope [397].

Compelling evidence indicates that VEGFR1 and VEGFR2 are the primary mediators of tumor angiogenesis and vascular permeability [398, 399]. Accordingly, VEGFR1/2-related ligands, vascular endothelial growth factors (VEGFs), have become promising targets in malignancies. The VEGF family consists of five glycoproteins, VEGF-A, -B, -C, -D, and placenta growth factor (PlGF). Each VEGF exerts its activity by binding to the corresponding receptors. Specifically, VEGF-A binds to VEGFR1 and VEGFR2, VEGF-B and PlGF bind exclusively to VEGFR1 [400], whereas VEGF-C and VEGF-D bind to VEGFR2 and VEGFR3 [401, 402]. Based on this principle, the VEGF‑A-directed mAb bevacizumab was approved by the FDA as first-line therapy for metastatic colorectal cancer in combination with FOLFOXIRI (5-FU, leucovorin, oxaliplatin, and irinotecan) [403] (Fig. 5k). Bevacizumab binds to soluble VEGF-A and prevents VEGF-A from binding to its receptors (VEGFR1 and VEGFR2) by steric hindrance, thereby reducing blood vessel density, vascular permeability, and liver metastases of colorectal cancer mediated by VEGFR1 and VEGFR2 [404]. In contrast, ziv-aflibercept adopts a new strategy to antagonize VEGFs by utilizing the high binding affinity between VEGFRs and VEGFs (Fig. 5l). Specifically, ziv-aflibercept is constructed as a soluble receptor decoy that fuses the second immunoglobulin (Ig)-like domain of VEGFR1 and the third Ig-like domain of VEGFR2 to the Fc portion of human IgG1 [405]. Therefore, ziv-aflibercept acts as a VEGF trap that antagonizes multiple VEGFs, including VEGF-A, VEGF-B, and PlGF [406]. Similar to bevacizumab, ziv-aflibercept was also approved by the FDA as first-line therapy for metastatic colorectal cancer in combination with FOLFIRI (5-FU, leucovorin, and irinotecan) [407]. However, almost half of patients develop metastases, and most have unresectable tumors [408].

Increasing evidence indicates that the overactivation of RTKs and their downstream signaling cascades contribute to the development, progression, and acquired drug resistance of colorectal cancer [409, 410]. Regorafenib is a potent multitarget TKI that blocks angiogenic kinases (VEGFR1/2/3, PDGFRα/β, and FGFR1/2) and oncogenic kinases (KIT, RET, RAF1, BRAF^WT^, and BRAF^V600E^) [411] (Fig. 5m). CYP3A4 and UGT1A9 metabolize Regorafenib into two main circulating metabolites, M-2 (N-oxide) and M-5 (N-oxide/N-desmethyl) [412]. Both metabolites exhibit similar pharmacological activity to regorafenib. However, regorafenib primarily seems to induce stabilization of the disease rather than tumor regression in metastatic colorectal cancer because few patients achieve an objective tumor response [413]. Thus, regorafenib was approved by the FDA for patients with metastatic colorectal cancer who had received previous standard therapies [414].

Nevertheless, the OS benefit of regorafenib is 1.4 months, and over 50% of patients with colorectal cancer eventually develop resistance and progressive disease after a transient response to the standard therapy [413, 415]. Additional treatment options are needed for patients with metastatic colorectal cancer who have exhausted all standard therapies [416]. Trifluridine/tipiracil (known as TAS-102) is an antimetabolite agent that comprises a trifluridine (thymidine-based nucleoside analog) and a tipiracil (thymidine phosphorylase inhibitor) [417] (Fig. 5n). Like 5-FU, trifluridine inhibits thymidylate synthase (a central enzyme in DNA synthesis) and incorporates itself into DNA, leading to cell death [418]. Of note, trifluridine exhibits higher activity than 5-FU because it does not elicit an autophagic survival response as 5-FU [419]. Tipiracil attenuates thymidine phosphorylase-mediated catabolism of trifluridine, which increases the bioavailability and potentiates the in vivo efficacy of trifluridine [417]. Intriguingly, tipiracil/trifluridine exhibits pharmacological activity in both 5-FU-sensitive and 5-FU-resistant cancer cells [420, 421]. Thus, tipiracil/trifluridine was approved for the treatment of patients with metastatic colorectal cancer who are refractory to or are not considered candidates for current standard chemotherapy and biological therapy [422].

### FDA-approved therapeutic drugs for prostate cancers

**Fig. 6:**
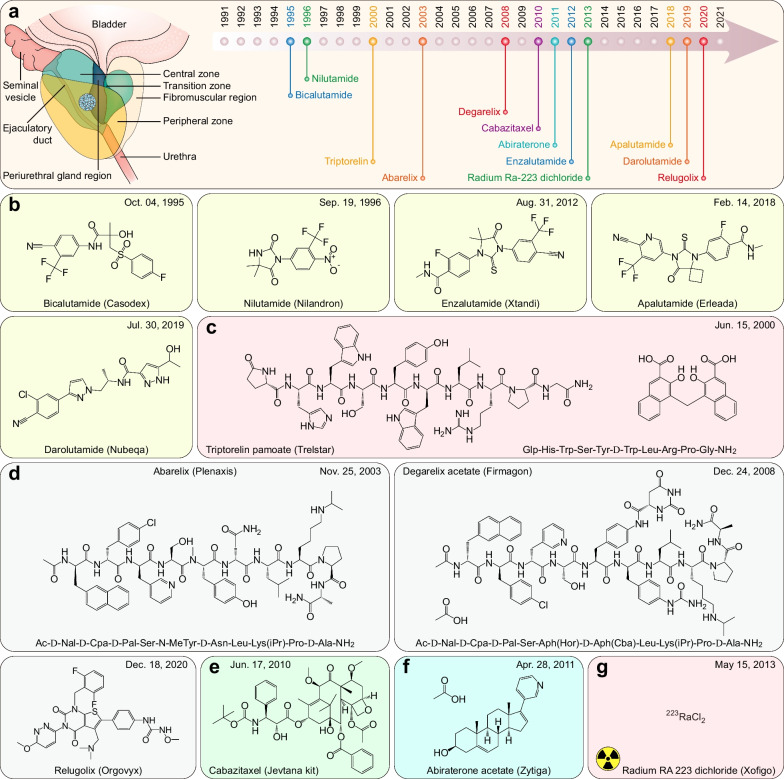
FDA-approved therapeutic drugs for prostate cancers. **a** Distribution of therapeutic drugs for prostate cancers during the past 31 years (adapted from [423]). **b** AR antagonists. **c** GnRH agonist. **d** GnRH receptor antagonists. **e** Microtubule inhibitor. **f** CYP17A1 inhibitor. **g** α-particle-emitting radiopharmaceutical

Prostate cancer accounted for 14.1% of cancer cases and 6.8% of cancer-related mortalities among males worldwide in 2020, second only to lung cancer [2]. Over the past 31 years, the FDA granted 12 new therapeutic drug approvals for prostate cancer (Fig. 6a and Table 5).

**Table 5:** FDA-approved therapeutic drugs for prostate cancers

Drug (brand name)	Sponsor	Properties	Indication	Approval date	Review
Bicalutamide (Casodex)	Ani pharms	AR antagonist	Advanced prostate cancer	10/04/1995	S
Nilutamide (Nilandron)	Concordia	AR antagonist	Metastatic prostate cancer	09/19/1996	S
Enzalutamide (Xtandi)	Astellas	AR antagonist	mCRPC	08/31/2012	P
Apalutamide (Erleada)	Janssen	AR antagonist	Prostate cancer	02/14/2018	P
Darolutamide (Nubeqa)	Bayer	AR antagonist	Prostate cancer	7/30/2019	P
Triptorelin pamoate (Trelstar)	Verity	GnRH agonist	Advanced prostate cancer	06/15/2000	S
Abarelix (Plenaxis)	Speciality	GnRH receptor antagonist	Advanced prostate cancer	11/25/2003	P
Degarelix acetate (Firmagon)	Ferring	GnRH receptor antagonist	Advanced prostate cancer	12/24/2008	S
Relugolix (Orgovyx)	Myovant	GnRH receptor antagonist	Prostate cancer	12/18/2020	P
Cabazitaxel (Jevtana kit)	Sanofi	Microtubule-stabilizing agent	Prostate cancer	06/17/2010	P
Abiraterone acetate (Zytiga)	Janssen	CYP17A1 inhibitor	mCRPC	04/28/2011	P
Radium RA 223 dichloride (Xofigo)	Bayer	α-particle-emitting radiopharmaceutical	mCRPC	05/15/2013	P

*AR* Androgen receptor; *CYP17A1* Cytochrome P450 17A1; *GnRH* Gonadotropin-releasing hormone; *mCRPC* Metastatic castration-resistant prostate cancer; *O* Orphan; *P* Priority; *S* Standard

The progression of prostate cancer is frequently accompanied by rising androgen receptor (AR) overexpression owing to the proliferation of luminal epithelial cells of the prostate caused by the accumulation of somatic mutations or *AR* amplification [423, 424]. Overexpression of AR enhances the binding activity to androgens [425], such as dihydrotestosterone (DHT), which initiates the translocation of AR from the cytoplasm to the nucleus [426], where AR binds to specific DNA sequences, namely, androgen response elements (AREs), thereby initiating the transcription of its target genes, including prostate-specific antigen (PSA) [427–429]. As a result, PSA is frequently elevated in patients with prostate cancer and has become a classic biomarker for disease diagnosis [430], whereas an AR-mediated transcription program increases cell proliferation [431] and changes central metabolism and biosynthesis [432], leading to disease progression [433].

Based on this principle, five AR antagonists have been approved by the FDA for advanced or metastatic prostate cancer; these include two first-generation antiandrogens (nilutamide and bicalutamide) (another antiandrogen, flutamide, brand name: Eulexin, approved on January 27, 1989, by the FDA) and three second-generation antiandrogens (enzalutamide, apalutamide, and darolutamide) (Fig. 6b). Mechanistically, all these AR antagonists competitively bind to the ligand-binding domain (LBD) of AR and prevent androgens from binding to AR. Compared to first-generation antiandrogens, second-generation antiandrogens improve the pharmacologic properties by which AR translocation and AR-mediated transcription are blocked [434]. However, cross-resistance widely exists throughout antiandrogens due to mutations or deletions in the LBD of AR [435, 436]. Specifically, mutations of *AR*^H874Y^, *AR*^V715M^, and *AR*^T877A/S^ confer the conversion of flutamide and nilutamide from AR antagonists to agonists [437]. Fortunately, these AR mutants are sensitive to bicalutamide [437]. However, the *AR*^W741C/L^ mutations convert bicalutamide from an AR antagonist to an agonist [438, 439] but are sensitive to nilutamide [440]. The *AR*^F876L^ mutation also switches the second-generation antiandrogens enzalutamide and apalutamide from AR antagonists to agonists [441–443] but is sensitive to the most novel antiandrogen darolutamide [444]. Moreover, darolutamide exhibits antagonistic effects on mutations of *AR*^W741L^ and *AR*^T877A^ [444]. Nevertheless, it still cannot overcome the resistance of AR LBD-deletion variants [435]. Therefore, the N-terminus of AR should be considered the target domain for the next generation of antiandrogens.

Androgen deprivation therapy (ADT) is another strategy for the treatment of prostate cancer that suppresses serum testosterone to castration levels, thereby preventing AR activation and blocking AR-mediated transcription. Both gonadotropin-releasing hormone (GnRH, also known as luteinizing hormone-releasing hormone (LH-RH)) agonists and GnRH antagonists are used for ADT, although they have different pharmacological mechanisms [445].

GnRH agonists stimulate the pituitary gland, which causes a flare phenomenon by which testosterone levels are initially increased for 5 ~ 12 days [446]. However, sustained overstimulation leads to the downregulation and desensitization of GnRH receptors located in gonadotroph cells [447], thereby reducing the luteinizing hormone (LH) and follicle-stimulating hormone (FSH) levels, which eventually decreases serum testosterone and achieves castration levels [446, 447]. In contrast, GnRH antagonists induce a rapid decrease in LH and FSH by competitively binding to the GnRH receptors [448] and decrease serum testosterone to castration levels without causing a flare phenomenon [449]. Based on this principle, the GnRH agonist triptorelin (Fig. 6c) and three GnRH antagonists (abarelix, degarelix, and relugolix) (Fig. 6d) were approved by the FDA. Another GnRH agonist, histrelin, was approved by the FDA for prostate cancer under the brand name Vantas on Oct. 12, 2004 (Additional file 1: Table S1, page 1).

Similar to the previously approved GnRH agonists goserelin (brand name: Zoladex, approved by the FDA on Dec. 29, 1989) and leuprolide (brand name: Lupron Depot, approved by the FDA on January 26, 1989), both triptorelin (decapeptide) and histrelin (nonapeptide) are synthetic, polypeptide GnRH analogs. Compared to the endogenous GnRH (Glp-His-Trp-Ser-Tyr-Gly-Leu-Arg-Pro-Gly-NH_2_), both triptorelin and histrelin preserve the N-terminal five amino acid residues (Glp-His-Trp-Ser-Tyr) and C-terminal three amino acid residues (Leu-Arg-Pro) [450–452]. Endogenous GnRH is rapidly degraded in blood by enzymatic cleavage at the Gly residue in position 6 [453]; Gly^6^ is replaced by D-Trp and D-His (Bzl) in triptorelin and histrelin, respectively, to increase resistance to degradation and thereby prolong the half-life time in vivo [452, 454]. On the other hand, the Gly^10^ of endogenous GnRH is replaced by AzaGly-NH_2_ in goserelin and Pro-NHEt in leuprolide and histrelin to increase the binding affinity between GnRH agonists and the GnRH receptor [452, 455]. Histrelin is a GnRH agonist administered once yearly that exhibits long-term efficacy and tolerability as a subcutaneous implant [456]. Although leuprolide, a GnRH agonist administered twice yearly [457], is comparable to histrelin in the drug administration schedule, 10% of patients treated with leuprolide failed to achieve medical castration [458].

Compared to triptorelin, histrelin may reduce the flare phenomenon and testosterone microsurges upon repeated administration to a certain extent. However, GnRH agonists cannot eliminate these adverse effects due to their natural pharmacological mechanism [452]. Therefore, GnRH antagonists have been developed for the treatment of advanced prostate cancer. However, first- and second-generation GnRH antagonists are unsuitable for clinical use due to solubility limitations and systemic allergic reactions caused by histamine release [452, 459, 460]. Abarelix and degarelix are third-generation GnRH antagonists derived from endogenous GnRH. Compared to endogenous GnRH, the N-terminal three amino acid residues (crucial for biological activity) Tyr^5^-Gly^6^, Arg^8^, and Gly^10^ are substituted in abarelix and degarelix to eliminate the biological activity of GnRH but increase the stability and binding affinity to the GnRH receptor [460].

As expected, abarelix induces a rapid suppression of serum testosterone and PSA levels and achieves medical castration without a testosterone surge [461–463]. However, it also causes inevitable adverse effects, such as severe allergic reactions upon long-term administration [462, 464], and exhibits more frequent and shorter time intervals in escape from castration than complete ADT [446, 465]. Consequently, abarelix was withdrawn from the market in 2005 [466].

In contrast, degarelix is generally well tolerated, with most adverse events being mild to moderate in severity [467]. Additionally, the long-term clinical efficacy in suppressing testosterone and PSA levels are comparable to that of leuprolide over a one-year treatment period [468], and PSA-PFS is significantly improved upon degarelix treatment compared to leuprolide [469]. Thus, degarelix can be an alternative to GnRH agonists. Relugolix is a nonpeptidic drug and the first orally administered GnRH antagonist [470] that exhibits significantly superior clinical efficacy and a lower incidence of major adverse cardiovascular events than leuprolide [471]. Given the easier administration, relugolix is likely to become the new standard of care, although whether it is superior to surgical or established chemical castration treatments remains to be proven [472].

Cabazitaxel is a microtubule-stabilizing agent that binds to the N-terminus of the β-tubulin subunit, which promotes the assembly of tubulin into microtubules and stabilizes the mitotic spindle [473, 474]. It is synthesized from 10-deacetyl baccatin III, a compound extracted from the needles of yew trees (*Taxus* spp.) [475]. Compared to previous taxanes, such as paclitaxel and docetaxel, cabazitaxel exhibits favorable pharmacological efficacy, including increased cytotoxic activity in multidrug- and docetaxel-resistant cancer cells, probably due to a lower affinity for P-glycoprotein than docetaxel [476]. As expected, cabazitaxel exhibited an encouraging clinical advantage for the treatment of metastatic castration-resistant prostate cancer (mCRPC) compared to docetaxel [477, 478] and was approved as a second-line chemotherapy option for mCRPC [479] (Fig. 6e).

Cytochrome P450 17A1 (CYP17A1) is critical for producing androgenic and osteogenic sex steroids with its hydroxylase and 17, 20-lyase activities [480]. CYP17A1 is significantly elevated in mCRPC, making it an essential target for the treatment of mCRPC [481]. Abiraterone is a potent, selective, irreversible CYP17A1 inhibitor that binds to haem iron and occupies the majority of the enclosed active site of CYP17A1 [482], thereby attenuating the enzymatic activity of CYP17A1 and preventing androgen biosynthesis [483]. Abiraterone exhibits favorable clinical efficacy, making it an essential first-line option for the treatment of mCRPC [484–487] (Fig. 6f).

Radium RA 223 dichloride (^223^RaCl_2_) is a radiopharmaceutical that has been approved for patients with prostate cancer-derived symptomatic bone metastases [488] (Fig. 6g). ^223^RaCl_2_ exerts its pharmacological effect through Radium-223 (^223^Ra), an α-particle-emitting radioisotope. ^223^Ra is also a calcium mimetic that binds preferentially to the newly formed bone in areas of bone metastases, with a half-life of 11.4 days and maximal tissue penetration of fewer than 100 μm [489, 490]. Each atom of ^223^Ra emits four high linear energy α-particles (composed of two protons and two neutrons), which exert pharmacological actions by inducing DNA DSBs in directly irradiated cells and adjacent cells [490] or by producing extracellular reactive oxygen species (ROS) in directly irradiated cells and then inducing DNA DSBs in adjacent cells with a bystander effect [491].

### FDA-approved therapeutic drugs for urologic cancers

**Fig. 7:**
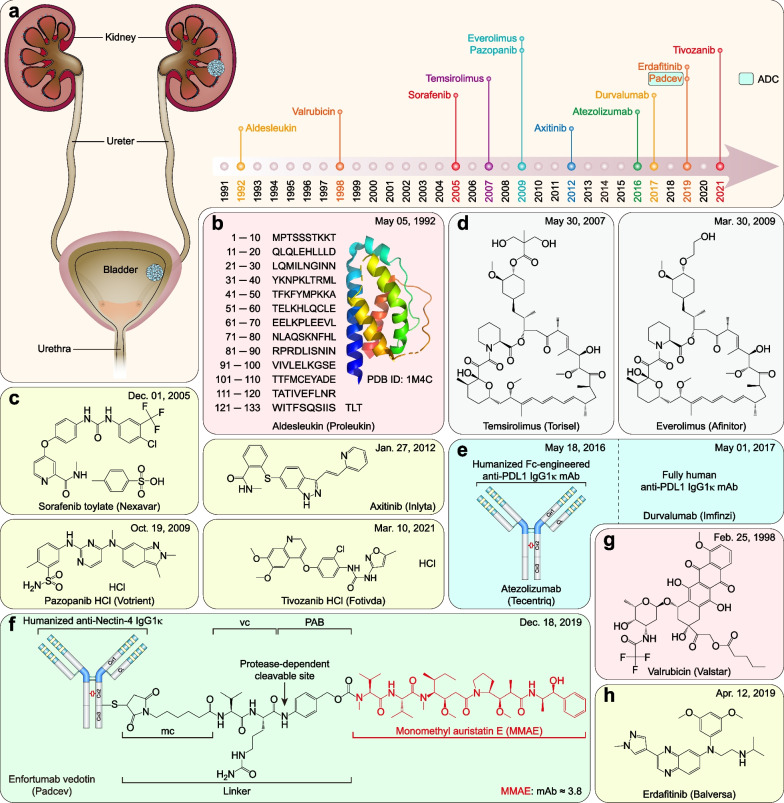
FDA-approved therapeutic drugs for urologic cancers. **a** Distribution of therapeutic drugs for urologic cancers during the past 31 years (adapted from [867]). **b** Recombinant human IL-2 (obtained from www.rcsb.org and go.drugbank.com). **c** Multitarget TKI and VEGFR inhibitors. **d** mTOR inhibitors. **e** PDL1-directed mAbs. **f** Nectin-4-directed ADC. **g** DNA topoisomerase inhibitor. **h** FGFR inhibitor

Aside from prostate cancer, kidney and bladder cancers are the most common urologic cancers [2]. Over the past 31 years, the FDA granted 12 new therapeutic drug approvals for urologic cancers (Fig. 7a and Table 6).

**Table 6:** FDA-approved therapeutic drugs for urologic cancers

Drug (brand name)	Sponsor	Properties	Indication	Approval date	Review
Aldesleukin (Proleukin)	Chiron	Recombinant human interleukin-2	Metastatic RCC	05/05/1992	O
Sorafenib toylate (Nexavar)	Bayer	Multitarget TKI (VEGFR2/3, PDGFRβ, FLT3, KIT, RAF1, and BRAF)	Advanced RCC	12/01/2005	P, O
Pazopanib HCl (Votrient)	Novartis	Multitarget TKI (VEGFRs, PDGFRα/β, FGFR1/2, KIT)	Metastatic RCC	10/19/2009	S
Axitinib (Inlyta)	Pfizer	VEGFRs inhibitor	Advanced RCC	01/27/2012	S
Tivozanib HCl (Fotivda)	Aveo Pharms	VEGFRs inhibitor	Advanced RCC	03/10/2021	S
Temsirolimus (Torisel)	Merck & Co	mTOR inhibitor	Advanced RCC	05/30/2007	P
Everolimus (Afinitor)	Novartis	mTOR inhibitor	Advanced RCC	03/30/2009	P
Atezolizumab (Tecentriq)	Genentech	PDL1-directed mAb	Urothelial carcinoma	05/18/2016	N/A
Durvalumab (Imfinzi)	AstraZeneca	PDL1-directed mAb	Urothelial carcinoma	05/01/2017	N/A
Enfortumab vedotin (Padcev)	Astellas	Nectin-4-directed ADC	Urothelial carcinoma	12/18/2019	P
Valrubicin (Valstar)	Endo Pharm	A semisynthetic analog of anthracycline doxorubicin	BCG-refractory CIS of urinary bladder cancer	09/25/1998	P, O
Erdafitinib (Balversa)	Janssen	FGFR inhibitor	Bladder cancer	04/12/2019	P

*BCG* Bacillus Calmette-Guérin; *CIS* Carcinoma in situ; *FGFR* Fibroblast growth factor receptor; *mTOR* Mammalian target of rapamycin; *O* Orphan; *P* Priority; *PDGFR* Platelet-derived growth factor receptor; *PDL1* Programmed death-ligand 1; *RCC* Renal cell carcinoma; *S* Standard; *VEGFR* Vascular endothelial growth factor receptor

#### Renal cell carcinoma

Kidney cancer accounted for 2.2% of cancer cases and 1.8% of cancer-related mortalities worldwide in 2020 [2]. Renal cell carcinoma (RCC) is the most common subtype (~ 70%) of kidney cancer [492]. One recombinant human interleukin-2 (IL-2, aldesleukin), four TKIs (sorafenib, pazopanib, axitinib, and tivozanib), and two mammalian targets of rapamycin (mTOR) inhibitors (temsirolimus and everolimus) have been approved for the treatment of RCC in the past three decades (Fig. 7b-d).

IL-2 was first discovered as a T cell growth factor in 1976 [493] and cloned in 1983 [494]. Over the ensuing years, IL-2 was proven to be a pivotal cytokine produced primarily by CD4^+^ T cells [495, 496]. As a pleiotropic mediator within the immune system, it interacts with IL-2 receptors (IL-2Rα, IL-2Rβ, and IL-2Rγ) and induces the proliferation and differentiation of immune cells, thereby regulating a range of diseases involving infection, autoimmune disease, and cancer [496]. Aldesleukin is a nonglycosylated, modified form of human endogenous IL-2 that exerts its antitumor activity by enhancing the cytotoxicity of T lymphocytes and the activity of natural killer and lymphokine-activated killer (LAK) cells [497] (Fig. 7b). Aldesleukin monotherapy achieves an ORR of 14 ~ 25% and exhibits durable antitumor activity in patients with metastatic RCC [498, 499].

Sorafenib is an oral first-generation multitarget TKI that targets several RTKs, including RAF1, BRAF^WT^, BRAF^V600E^, VEGFRs, PDGFR-β, FGFR1, FMS-like tyrosine kinase 3 (FLT3), KIT, and RET [500, 501]. It occupies the ATP adenine binding pocket of these RTKs with its distal 4-pyridyl ring, which blocks the autophosphorylation of these RTKs, thereby attenuating the mitogen-activated protein kinase (MAPK)/extracellular signal-regulated kinase (ERK) signaling pathway [500, 502]. Given its potent antitumor effects, sorafenib is applied to various malignancies in addition to RCC [501]. Sorafenib resistance inevitably occurs and mainly involves mutations in RTKs and activation of the bypass pathway [503]. Regorafenib, as a fluoro‐sorafenib, provides a nearly 3-month improvement in OS in HCC patients progressing on sorafenib treatment [504]. However, regorafenib cannot overcome sorafenib resistance because they share a similar structure [505]. Sunitinib is another first-generation multitarget TKI with a similar target profile to sorafenib. Intriguingly, sequential sorafenib-sunitinib and vice versa provide similar clinical benefits in metastatic RCC [506]. In contrast, pazopanib is an oral second-generation multitarget TKI that preferentially targets VEGFRs, PDGFRα/β, and KIT [507–509]. It competes with ATP for binding to the cytoplasmic domain of these RTKs and prevents ATP-induced activation [510]. Pazopanib retains clinical activity in patients with advanced clear-cell RCC after failure of sunitinib or bevacizumab [511]. Thus, pazopanib is non-inferior to sunitinib as first-line therapy in clinical efficacy and exhibits advantages in the safety profile [512]. Axitinib and tivozanib are selective second-generation VEGFR inhibitors that exhibit greater selectivity for VEGFRs than other TKIs (*e.g.*, sorafenib, sunitinib, pazopanib) [513, 514]. Axitinib is a substituted indazole derivative produced from a structure-based drug design [515]. It exhibits antitumor activity and a manageable safety profile in sorafenib-refractory metastatic RCC [516] but has no significant superiority over sorafenib as first-line therapy [517]. Compared with sorafenib, axitinib significantly prolongs the median PFS by two months and can be an option for second-line therapy [518, 519]. Nevertheless, axitinib plus avelumab or pembrolizumab therapies exhibit significant clinical benefits in advanced RCC as first-line treatment compared to the standard of care of sunitinib [520, 521]. Tivozanib is a quinoline urea derivative that interacts with the ATP-binding site and the allosteric-binding site consisting of the DFG motif within the activation loop of VEGFR, similar to sorafenib [522]. It inhibits VEGF-induced VEGFR phosphorylation and blocks VEGF-dependent but not VEGF-independent MAPK activation [523]. Tivozanib improves PFS and is better tolerated as third- or fourth-line therapy than sorafenib [524] (Fig. 7c).

mTOR is a serine/threonine-protein kinase that governs a diverse set of biological events by joining with other components to form two distinct complexes known as mTOR complex 1 (mTORC1) and mTOR complex 2 (mTORC2) [525]. mTORC1 is composed of three core components: mTOR, mammalian lethal with SEC13 protein 8 (mLST8, also known as GβL) [526], and its unique defining subunit, regulatory-associated protein of mTOR (RAPTOR) [527]. In lieu of RAPTOR, mTORC2 contains rapamycin-insensitive companion of mTOR (RICTOR) [528, 529]. mTORC1 governs glucose metabolism, cell cycle progression, cell survival, and the biosynthesis of proteins, lipids, and nucleotides, while mTORC2 governs cytoskeletal rearrangement and prosurvival pathways [525]. Temsirolimus and everolimus are derivatives of sirolimus (also known as rapamycin) (Additional file 1: Table S1, page 11), a compound extracted from a *Streptomyces hygroscopicus* soil bacterium [530]. Mechanistically, rapamycin, temsirolimus, and everolimus are inhibitors of mTORC1. Similar to rapamycin, both temsirolimus and everolimus bind to FK506-binding protein 12 (FKBP12) and form a gain-of-function complex, which subsequently prohibits the activation of mTOR, resulting in cell cycle arrest and suppression of hypoxia-inducible factor-1α (HIF1α) and VEGF expression [531, 532].

Temsirolimus is a water-soluble ester of rapamycin with improved pharmaceutical properties, including stability and solubility, making it suitable for intravenous administration [533]. Intriguingly, temsirolimus is hydrolyzed by CYP3A4 to its major metabolite rapamycin in vivo [534]. Compared with interferon-α (IFNα) monotherapy, temsirolimus improves OS among patients with metastatic RCC [535]. Everolimus is a hydroxyethyl ether derivative of rapamycin with superior pharmaceutical characteristics, making it suitable for oral administration. Unlike temsirolimus, everolimus is not converted to rapamycin in vivo [532]. Compared with placebo, everolimus prolongs PFS in patients with metastatic RCC previously treated with sunitinib and/or sorafenib [536]. Compared to temsirolimus, everolimus exhibits superior clinical efficacy in metastatic RCC in terms of prolonging OS and PFS and decreasing the risk of death [537, 538] (Fig. 7d).

RCC is a highly vascularized tumor prone to distant metastasis [503]. Mechanistically, clear-cell RCC accounts for approximately 80 ~ 85% of metastatic RCC cases [539], whereas 60% of clear-cell RCC harbors loss-of-function of the von Hippel–Lindau (*VHL*) tumor suppressor gene, which leads to the accumulation of HIF1α and activation of its target genes, including *VEGF* and *PDGF* [540]. It explains why all these TKIs target VEGFRs and/or PDGFRs, although they have different target profiles. In addition, two mTOR inhibitors can reduce the expression of HIF1α and VEGF. Before 2005, nonspecific immune cytokines, such as IL-2 and IFNα, were previously the mainstays of therapy for advanced RCC [540]. Currently, PD1-directed mAbs, such as pembrolizumab and nivolumab, plus TKIs, such as axitinib [521], lenvatinib [541], and cabozantinib [542], have become the first-line regimens for patients with advanced or metastatic RCC.

#### Bladder cancer

Bladder cancer accounted for 3.0% of cancer cases and 2.1% of cancer-related mortalities worldwide in 2020 [2]. Urothelial carcinoma accounts for approximately 90% of bladder cancers [543]. Over the past 31 years, the FDA has approved five therapeutic drugs for bladder cancer treatment (Fig. 7e–h).

PDL1 is an immune checkpoint expressed in 20% of tumor cells and 40% of tumor-infiltrating mononuclear cells (TIMCs) in urothelial carcinoma [544]. It binds to its receptor PD1 on the surface of T lymphocytes and negatively regulates the antitumor function of T lymphocytes [545]. PDL1-directed mAbs bind exclusively to PDL1 and block the interaction between PDL1 and PD1, which reactivates the antitumor immunity of T lymphocytes [546]. Mechanistically, atezolizumab binds to the CC′, C′C″, and FG loops of PDL1, while durvalumab binds to the CC′ loop and N-terminal region of PDL1 [547]. The Fc fragments of both mAbs are engineered to eliminate the ADCC effect and complement-dependent cytotoxicity (CDC) and prevent the depletion of activated T lymphocytes [548, 549]. Atezolizumab is the first PDL1-directed mAb [550] that exhibits durable activity, good tolerability, and superior clinical efficacy compared with chemotherapy in patients with platinum-treated locally advanced or metastatic urothelial carcinoma [551–553]. Notably, an increase in the mutation load increases the response to atezolizumab [551]. Likewise, durvalumab exhibits at least equivalent clinical efficacy to atezolizumab [554–556]. Currently, other PDL1 (avelumab) [557, 558] and PD1 (pembrolizumab [559, 560] and [561, 562])-directed mAbs are also used as first- or second-line treatments for urothelial carcinoma (Fig. 7e).

Nectin-4 is a type I transmembrane protein expressed at low levels in normal human tissues and is also known as poliovirus receptor-like 4 (PVRL4) [563, 564]. It acts as an oncoprotein [565] that promotes cancer cell proliferation and metastasis by activating Wnt/β-catenin [566] and the HER2-mediated PI3K/AKT signaling pathway [567–569]. Nectin-4 is expressed in 69% of solid tumors [563] and overexpressed in more than 60% of bladder cancers (or urothelial carcinomas) [563, 570], making it an attractive target for urothelial carcinoma treatment. Enfortumab vedotin is a nectin-4-directed ADC composed of enfortumab and MMAE with a protease-cleavable MC-vc-PAB linker [563], similar to tisotumab vedotin. Enfortumab is a nectin-4-directed mAb that binds to the extracellular domain of human nectin-4 [563] (Fig. 7f). Enfortumab vedotin significantly improves the median OS and PFS compared with that with chemotherapy (docetaxel, paclitaxel, or vinflunine) in patients with locally advanced or metastatic urothelial carcinoma who were previously treated with platinum-based treatment and PD1/PDL1 blockade, providing a new option for this patient population [571, 572].

Valrubicin is a semisynthetic analog of the anthracycline doxorubicin that binds weakly to DNA [573] (Fig. 7g). Compared to doxorubicin and epirubicin, valrubicin and its metabolites exhibit lower potency and less toxicity and exert antitumor effects by interfering with TOP2A-mediated cleavage and resealing of DNA, leading to the inhibition of DNA elongation and RNA biosynthesis [573–575]. Therefore, the S-G2 transition of the cell cycle is blocked, and chromosome stability is disrupted. Valrubicin is effective and well tolerated in patients with bacillus Calmette-Guérin (BCG)-refractory carcinoma in situ (CIS) of the bladder [576].

In addition, genetic alterations of FGFRs, including amplification, mutation, and rearrangement, occur in approximately one-third of urothelial carcinomas, making FGFRs promising therapeutic targets [577]. Erdafitinib is an oral pan-FGFR inhibitor that binds to the inactive DGF-D_in_ conformation, which prevents the FGF ligand-induced dimerization, phosphorylation, and activation of FGFRs [578–580] (Fig. 7h). Compared to other inhibitors (rogaratinib [581], pemigatinib [582], and infigratinib [583]) with an ORR of approximately 25%, erdafitinib exhibits superior clinical efficacy with an ORR of 40% [584]. Thus, erdafitinib was approved for patients with locally advanced or metastatic urothelial carcinoma harboring *FGFR2* or *FGFR3* genetic alterations [579].

### FDA-approved therapeutic drugs for melanoma and other skin cancers

#### Melanoma

**Fig. 8:**
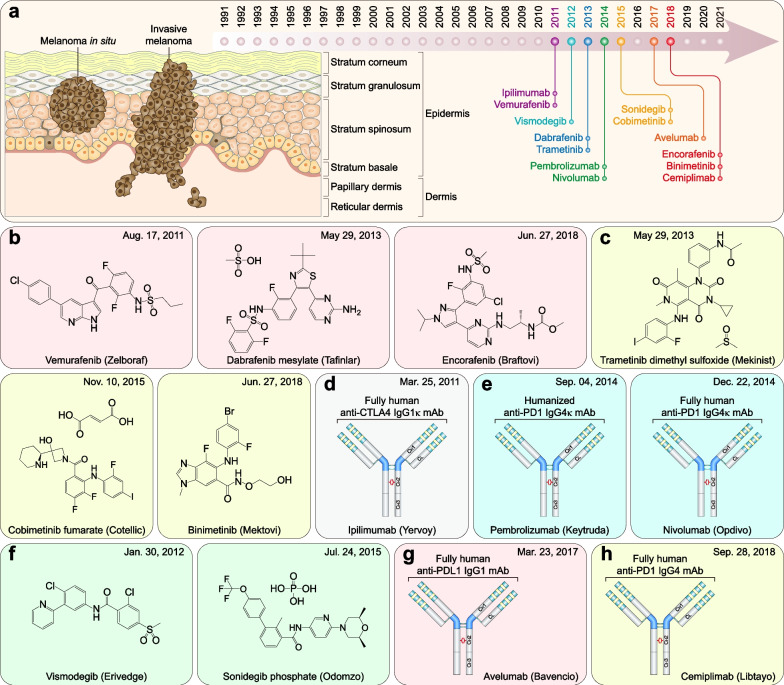
FDA-approved therapeutic drugs for melanoma and other skin cancers. **a** Distribution of therapeutic drugs for melanoma and other skin cancers during the past 31 years (adapted from [868]). **b** BRAF inhibitors. **c** MEK inhibitors. **d** CTLA4‑directed mAb. **e** PD1-directed mAbs. **f** Smoothened inhibitors. **g** PDL1-directed mAb. **h** PD1-directed mAb

Melanoma accounted for 1.7% of cancer cases and 0.6% of cancer-related mortalities worldwide in 2020 [2]. Nine therapeutic drugs (including BRAF and MAPK/ERK kinase (MEK) inhibitors and cytotoxic T lymphocyte antigen 4 (CTLA4)‑ and PD1-directed mAbs) have been approved by the FDA for melanoma in the past three decades (Fig. 8a and Table 7).

**Table 7:** FDA-approved therapeutic drugs for melanoma and other skin cancers

Drug (brand name)	Sponsor	Properties	Indication	Approval date	Review
Vemurafenib (Zelboraf)	Roche	BRAF inhibitor	BRAF-positive unresectable or metastatic melanoma	08/17/2011	P, O
Dabrafenib mesylate (Tafinlar)	Novartis	Kinase inhibitor with activity against BRAF^V600E/D/K^, wild-type BRAF and other kinases	Unresectable or metastatic melanoma with BRAF^V600E^ mutation	05/29/2013	S, O
Encorafenib (Braftovi)	Array BioPharma	BRAF inhibitor	*BRAF*-mutated melanoma	06/27/2018	S, O
Trametinib DMSO (Mekinist)	Novartis	MEK1/2 inhibitor	Unresectable or metastatic melanoma with BRAF^V600E^ mutation	05/29/2013	S, O
Cobimetinib fumarate (Cotellic)	Genentech	MEK inhibitor	Melanoma with *BRAF*^V600E/K^ mutations	11/10/2015	P, O
Binimetinib (Mektovi)	Array BioPharma	MEK inhibitor	*BRAF*-mutated melanoma	06/27/2018	S, O
Ipilimumab (Yervoy)	Bristol-Myers Squibb	CTLA4‑directed mAb	Unresectable or metastatic melanoma	03/25/2011	P, O
Pembrolizumab (Keytruda)	Merck	PD1-directed mAb	Metastatic melanoma	09/04/2014	P, O
Nivolumab (Opdivo)	Bristol-Myers Squibb	PD1-directed mAb	Unresectable or metastatic melanoma	12/22/2014	P, O
Vismodegib (Erivedge)	Genentech	Smoothened inhibitor	Advanced basal cell carcinoma	01/30/2012	P
Sonidegib phosphate (Odomzo)	Sun Pharma	Smoothened inhibitor	Basal cell carcinoma	07/24/2015	S
Avelumab (Bavencio)	Emd Serono	PDL1-directed mAb	Merkel cell carcinoma	03/23/2017	P, O
Cemiplimab (Libtayo)	Regeneron	PD1-directed mAb	Cutaneous squamous cell carcinoma	09/28/2018	P

*CTLA4* Cytotoxic T lymphocyte antigen 4; *MEK* MAPK/ERK kinase; *O* Orphan; *P* Priority; *PD1* Programmed death receptor-1; *PDL1* Programmed death-ligand 1; *S* Standard

BRAF forms a tight heterodimer with CRAF under the induction of active RAS and acts as a critical effector in the RAS–RAF–MEK–MAPK/ERK pathway [585]. However, approximately 70% of melanomas harbor *BRAF* mutations, whereas *BRAF*^V600E^ and *BRAF*^V600K^ account for 80 ~ 90% and 10 ~ 20% of all *BRAF* mutations, respectively [586, 587], making it a therapeutic target in melanoma [588]. Oncogenic BRAF constitutively activates the MAPK/ERK pathway, resulting in uncontrolled cell proliferation [589]. Therefore, three BRAF inhibitors (vemurafenib, dabrafenib, and encorafenib) (Fig. 8b) were approved for melanoma treatment.

Vemurafenib is a second-generation inhibitor with a mild selectivity for *BRAF*^V600E^ over *BRAF*^WT^. It occupies the ATP-binding pocket in the ‘αC‑OUT/DFG-in’ (active) conformation of BRAF and inhibits BRAF phosphorylation and activation, thereby attenuating downstream MEK–MAPK/ERK signaling transduction [590]. As expected, vemurafenib significantly improved OS and PFS in patients with previously untreated melanoma harboring the *BRAF*^V600E^ mutation [591]. In parallel, dabrafenib, another highly potent and specific inhibitor, exhibits a virtually identical clinical outcome to vemurafenib [592] and a BRAF binding mechanism similar to that of vemurafenib [593]. Dabrafenib plus trametinib (a MEK inhibitor) therapy adds a clear benefit over vemurafenib monotherapy in patients with unresectable or metastatic melanoma harboring *BRAF*^V600E/K^ mutations [594]. Unfortunately, most patients treated with vemurafenib or dabrafenib will develop disease progression following tumor regression within 6 ~ 8 months [595]. Encorafenib is still an αC‑OUT inhibitor of BRAF and is used in combination with binimetinib (another MEK inhibitor) in clinical practice [596]. It showed a longer residence time and lower off-rate than vemurafenib and dabrafenib in a preclinical study [597, 598]. Compared to vemurafenib or dabrafenib monotherapy, encorafenib plus binimetinib combination therapy significantly improves clinical efficacy and tolerability [599, 600].

MEK is a direct downstream target of BRAF, and *BRAF* mutations that cause overactivation of the RAS–RAF–MEK–MAPK/ERK pathway highly depend on MEK activity [601]. Thus, three MEK inhibitors (trametinib, cobimetinib, and binimetinib) have been approved and are frequently used in combination with BRAF inhibitors [602] (Fig. 8c). Trametinib stably binds to unphosphorylated MEK1/2 with high affinity and maintains MEK in an unphosphorylated state [603]. However, trametinib shows a low affinity for phosphorylated MEK1/2 [604]. In contrast, cobimetinib not only inhibits ERK1/2 phosphorylation but also retains the inhibitory effect of phosphorylated MEK1/2 [605], whereas binimetinib exhibits clinical activity in both *BRAF*-mutated and *NRAS*-mutated melanoma [606]. In clinical practice, trametinib is used in combination with dabrafenib [607, 608], cobimetinib is used with vemurafenib [609, 610], and binimetinib is used with encorafenib [599, 600]. All three combined therapies exhibit equivalent clinical outcomes, such as objective/complete response rate, median PFS, toxic effects, and two-year survival rate. Of note, the mOS is 33.6 months with encorafenib plus binimetinib, 22.3 months with vemurafenib plus cobimetinib, and 25.1 months with dabrafenib plus trametinib [602].

CTLA4 is a second counterreceptor for the B7 family of costimulatory molecules that functions as a negative regulator of T lymphocyte activation [611]. Blocking CTLA4 significantly enhances antitumor immunity [612]. Ipilimumab is the first CTLA4-directed mAb that binds to CTLA4 on the cell surface, thereby blocking the interaction between CTLA4 and B7.1/B7.2 and restoring the activation of T lymphocytes [613] (Fig. 8d). Compared with gp100 monotherapy, ipilimumab monotherapy or plus glycoprotein 100 (gp100) significantly improved the median OS of patients with advanced or metastatic melanoma [614].

PD1 is another negative regulator of T lymphocytes that confers tumor immune evasion by interacting with its ligands PDL1 and PDL2 [615, 616]. PD1 is also expressed in melanoma cells and contributes to tumor growth [617]. Pembrolizumab is a PD1-directed mAb that binds to the C’D loop of PD1 [618–620]. Pembrolizumab monotherapy is significantly superior to ipilimumab monotherapy in clinical trials [621, 622] and can be an effective treatment option for patients with ipilimumab-refractory advanced melanoma [623]. Nivolumab is another PD1-directed mAb that binds to the N-loop of PD1 [618, 620, 624] (Fig. 6e). It is frequently used in combination with ipilimumab for patients with advanced or metastatic melanoma [625, 626]. Compared with ipilimumab monotherapy, nivolumab monotherapy or ipilimumab plus ipilimumab significantly extends OS and five-year survival [627, 628] (Fig. 8e).

Nonmelanoma skin cancers (NMSCs) mainly encompass basal cell carcinoma, squamous cell carcinoma, and neuroendocrine skin carcinoma (also known as Merkel cell carcinoma) [629]. NMSCs are the most commonly diagnosed cancers, accounting for up to 30% of all human tumors [629–632] and 0.6% of cancer-related mortalities [2].

#### Basal cell carcinoma

Basal cell carcinoma constitutes approximately 80% of all NMSCs, and more than five million new cases are diagnosed each year worldwide [633, 634]. However, the absolute incidence and mortality are difficult to determine since basal cell carcinoma is usually excluded from cancer registry statistics [633]. In part, basal cell carcinoma is the most frequent human cancer subtype [632]. Loss-of-function mutations of tumor suppressor gene *patched homolog 1* (*PTCH1*) occur in 30 ~ 40% of basal cell carcinomas [635]. Dysfunctional PTCH1 causes constitutive activation of smoothened (SMO), resulting in continuous activation of hedgehog signaling and its target genes in basal cell carcinoma [174, 634], making SMO a promising target. Therefore, two SMO inhibitors (vismodegib and sonidegib) were approved for the treatment of basal cell carcinoma [636, 637] (Fig. 8f).

Vismodegib is the first SMO inhibitor that occupies the transmembrane domain core and forms hydrophobic interactions with SMO by a network of hydrogen bonds [638, 639], thereby inhibiting SMO activity and downstream signaling, regardless of PTCH1 [640]. However, approximately 21% of patients develop resistance within a year while undergoing continuous vismodegib treatment [641]. Various mutations in *SMO* are located in the drug-binding pocket of SMO and confer resistance to vismodegib by abrogating or impairing vismodegib binding to SMO, such as D473H, D473G, W281C, V321M, I408V, C469Y, and Q477E [642–646]. Sonidegib is another SMO inhibitor that binds to a drug-binding pocket of SMO similar to vismodegib. It exerts antitumor effects by inhibiting the transcriptional activity of glioma-associated oncogene (GLI) and inducing the expression of caspase-3 and the cleavage of PARP, resulting in cell cycle arrest and apoptosis [647]. However, sonidegib has an SMO binding pattern similar to that of vismodegib. As expected, sonidegib cannot overcome the vismodegib resistance induced by *SMO* mutations [648].

#### Merkel cell carcinoma

Merkel cell carcinoma is a rare but highly aggressive NMSC with neuroendocrine features [649] frequently associated with Merkel cell polyomavirus infection and accumulation of ultraviolet-induced mutations [650]. Approximately 50% of tumor cells and 55% of tumor-infiltrating immune cells (TIICs) express PDL1 in Merkel cell carcinoma [651]. Avelumab is a PDL1-directed mAb that binds to the CC’ loop of PDL1 [547, 652], thereby blocking the interaction between PDL1 and PD1, which reactivates the antitumor immunity of T lymphocytes, similar to atezolizumab and durvalumab (Fig. 8g). Avelumab is well tolerated with durable responses [653] and has become the standard-of-care treatment for metastatic and advanced Merkel cell carcinoma [654], similar to other anti-PD1 immunotherapies, including pembrolizumab [655, 656] and nivolumab [657].

#### Cutaneous squamous cell carcinoma

Cutaneous squamous cell carcinoma (CSCC) accounts for approximately 20% of NMSCs, second only to basal cell carcinoma in NMSCs [658]. In contrast to most basal cell carcinomas, CSCC is highly aggressive, prone to metastasis, and correlated with ultraviolet radiation [658, 659]. More than half of CSCC TIICs express PD1, especially CD4^+^ and CD8^+^ TILs, which show PD1 positivity rates of 73% and 80%, respectively [660]. Cemiplimab is a PD1-directed mAb that binds to the BC loop, C’D loop, and FG loop of PD1 with its heavy chain variable domain (V_H_). In contrast, the light chain variable domain (V_L_) of cemiplimab sterically inhibits the interaction between PDL1 and PD1 [661] (Fig. 8h). Given the considerable antitumor activity and acceptable safety, cemiplimab was approved for patients with metastatic or locally advanced CSCC [662–664].

### FDA-approved therapeutic drugs for thyroid cancer and other solid tumors

#### Thyroid cancer

Thyroid cancer accounted for 3.0% of cancer cases and 0.6% of cancer-related mortalities worldwide in 2020 [2]. Contrary to pancreatic cancer, thyroid cancer has the lowest mortality-to-incidence ratio (0.133) among all malignancies [2].

**Fig. 9:**
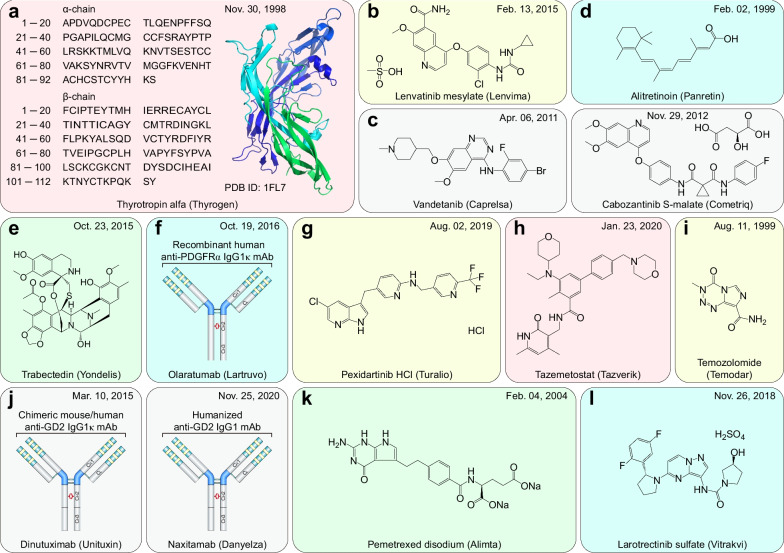
FDA-approved therapeutic drugs for thyroid cancer and other solid tumors. **a** Recombinant human thyroid-stimulating hormone (obtained from www.rcsb.org and go.drugbank.com). **b** & **c** Multitargeted TKIs. **d** 9-*cis*-retinoic acid. **e** DNA alkylating agent. **f** PDGFRα-directed mAb. **g** CSF1R, KIT, and FLT3 inhibitor. **h** EZH2 inhibitor. **i** DNA alkylating agent. **j** GD2‑directed mAbs. **k** Folate analog. **l** TRKs inhibitor

Differentiated thyroid cancer (DTC) is derived from the follicular epithelial cells of the thyroid, accounting for approximately 95% of all cases, whereas surgery followed by either radioiodine therapy or observation is the standard treatment for most patients [665]. It is crucial to stimulate iodine uptake by elevating thyroid-stimulating hormone (TSH) or depleting thyroid hormone prior to radioiodine (iodine-131) administration [666]. Thyrotropin alfa is a recombinant human TSH (rhTSH) synthesized in a genetically modified Chinese hamster ovary cell line (Fig. 9a). It stimulates the thyroid gland to produce thyroxine (T4), and its more bioactive form triiodothyronine (T3), which increases iodine uptake. Clinically, thyrotropin alfa is used for radioiodine ablation of DTC [667] and radioiodine scanning of poorly differentiated thyroid cancer [668]. However, 9% of DTCs recur after thyroid hormone plus radioiodine therapy [669], and approximately 30% of patients with advanced, metastatic DTCs have the radioiodine-refractory disease [670]. Loss or low expression of sodium–iodide symporter (NIS) is associated with radioiodine refractoriness [669, 670]. Genetic and epigenetic alterations induced activation of RTKs and their downstream RAS–RAF–MEK–MAPK/ERK and PI3K–AKT–mTOR pathways contribute to the dysfunction of NIS [671–673]. Lenvatinib is a TKI that targets VEGFRs, FGFRs, PDGFRα, RET, and KIT [674, 675] (Fig. 9b). It binds to the ATP-binding site and the neighboring region of RTKs, adopting a DFG-in conformation, compared to the DFG-out conformation of sorafenib [676]. As expected, lenvatinib significantly improves PFS with a high response rate in patients with radioiodine-refractory thyroid cancer [677].

Medullary thyroid cancer (MTC) originates in the parafollicular neuroendocrine cells of the thyroid and accounts for 1 ~ 2% of all cases [665]. Acquired somatic *RET* mutations and germline *RET* mutations are observed in 35 ~ 50% and 6.5% of sporadic MTCs, respectively [678, 679], and are considered secondary events rather than initiators that drive the tumorigenesis of MTC [680]. Of note, *RET*^M918T^ mutation is associated with a more aggressive disease and a poorer prognosis [681]. EGFR is overexpressed in 13% of MTCs, VEGFR2 expression is significantly higher in metastases than in the primary tumors of MTCs [682], and MET is overexpressed in thyroid epithelial cells [683]. These RTKs contribute to the tumorigenesis and angiogenesis of MTC [684]. Vandetanib is an inhibitor of VEGFR2, EGFR, and RET [678] that binds to the ATP-binding site of RTKs [685], leading to cell apoptosis rather than cell cycle arrest [686, 687]. However, *RET*^V804M/L^ gatekeeper mutations and *RET*-S904F mutation confer resistance to vandetanib, mainly by increasing the ATP affinity and autophosphorylation activity of RET kinase [688, 689]. Cabozantinib is an inhibitor of VEGFR2, MET, and RET [684] that also binds to the ATP-binding site of RTKs [690], thereby inhibiting autophosphorylation of RTKs, which leads to tumor hypoxia and apoptosis and suppresses metastasis, angiogenesis, and tumor growth [684, 691]. Cabozantinib significantly prolongs PFS in patients with unresectable, locally advanced, or metastatic MTC [684, 692, 693]. Intriguingly, cabozantinib potently inhibits native ROS1 and the crizotinib-resistant *ROS1*^G2032R^ mutation [694] and overcomes crizotinib resistance in *CD74*-*ROS1*^D2033N^-rearranged lung cancer [695] (Fig. 9c).

Soft tissue sarcomas (STSs) are rare tumors that account for 1% of all adult malignancies; these include Kaposi’s sarcoma, adipocytic tumors (*e.g.*, liposarcoma), smooth muscle tumors (*e.g.*, leiomyosarcoma), fibrohistiocytic tumors (*e.g.*, tenosynovial giant cell tumor), tumors of uncertain differentiation (*e.g.*, epithelioid sarcoma) [696], etc.

#### Kaposi’s sarcoma

Kaposi’s sarcoma accounted for 0.2% of cancer cases and 0.2% of cancer-related mortalities worldwide in 2020 [2]. It is a relatively rare cancer caused by Kaposi’s sarcoma-associated herpesvirus (KSHV, also known as human herpesvirus 8 (HHV8)) infection [697]. The skin and superficial mucosae are the most common sites of Kaposi’s sarcoma lesions [698]. Retinoid X receptor α (RXRα) and retinoic acid receptor γ (RARγ) control cell differentiation, proliferation, and apoptosis and are predominantly expressed in the skin [699, 700]. Alitretinoin is a 9-*cis*-retinoic acid that acts as a pan-agonist of RARs and RXRs [700]. It modulates cell differentiation and apoptosis in a variety of sarcomas by potentially inducing the formation of a homodimer of RXRs [701–703] (Fig. 9d). Alitretinoin gel demonstrates durable responses with tolerable safety in patients with acquired immunodeficiency syndrome (AIDS)-related Kaposi’s sarcoma [704, 705].

#### Liposarcoma

Liposarcoma is a rare malignant tumor of adipocytic differentiation that accounts for 15 ~ 20% of STS cases [706]. It is characterized by recurrent amplifications within chromosome 12, which leads to the overexpression of disease-driving genes [706]. Leiomyosarcoma is a malignant mesenchymal tumor that accounts for 10 ~ 20% of STS cases [707]. Leiomyosarcoma also exhibits complex genomic alterations involving DNA copy number changes and gene mutations [708]. Trabectedin is a tetrahydroisoquinoline alkaloid derived from the Caribbean marine tunicate *Ecteinascidia turbinata* [709] (Fig. 9e). It binds to the minor groove of DNA that bends DNA toward the major groove by forming trabectedin-DNA adducts, which block the G2/M phase transition of the cell cycle and inhibit cell proliferation [709–711]. The FDA-approved Trabectedin for patients with unresectable or metastatic liposarcoma or leiomyosarcoma who received a prior anthracycline-containing regimen [712].

#### Soft tissue sarcoma

PDGFRα expression in STSs is sevenfold higher than in normal tissues [713]. Olaratumab is a PDGFRα-directed mAb that selectively binds to the extracellular domain of PDGFRα, which prevents PDGF-AA, PDGF-BB, and PDGF-CC ligands from binding to PDGFRα [714, 715] (Fig. 9f). It inhibits the ligand-induced autophosphorylation of PDGFRα and downstream signaling, thereby blocking PDGFRα-mediated cell mitogenesis [716]. Olaratumab plus doxorubicin combination therapy significantly improves the PFS and OS compared to doxorubicin alone in patients with advanced STSs [717].

#### Tenosynovial giant cell tumor

Tenosynovial giant cell tumor (TGCT) is a rare, locally aggressive neoplasm mainly characterized by colony-stimulating factor-1 (*CSF1*) translocations and CSF1 receptor (CSF1R) overexpression [718, 719]. *CSF1* translocations result in local overexpression of CSF1, which attracts histiocytoid and CSF1R-expressing inflammatory cells [718–720]. Moreover, CSF1 promotes the differentiation of monocytes into tumor-associated macrophages (TAMs), which in turn facilitates tumor survival, growth, and metastases with their immunosuppressive effects [721–723]. Thus, the CSF1/CSF1R axis is critical for the tumorigenesis and progression of TGCTs. Pexidartinib is an inhibitor of CSF1R, KIT, and FLT3 that accesses the autoinhibited state of CSF1R through direct interactions with juxtamembrane residues embedded in the ATP-binding pocket, thereby blocking the CSF1/CSF1R axis [724] (Fig. 9g). Notably, pexidartinib retains activity against the quizartinib-resistant FLT3 gatekeeper F691L mutation [725]. As the first systemic therapy of TGCT, pexidartinib exhibits a robust tumor response with improved clinical outcomes [718, 723].

#### Epithelioid sarcoma

Epithelioid sarcoma is an ultrarare high-grade soft tissue sarcoma with clinicopathological complexities that predisposes patients to locoregional recurrence [726], accounting for 1.2 ~ 1.5% of STS cases [727]. INI1 (encoded by the *SMARCB1* tumor suppressor gene) is a core subunit of the switch/sucrose nonfermentable (SWI/SNF) chromatin remodeling complex frequently inactivated in epithelioid sarcomas [728]. The SWI/SNF complex is a crucial regulator of nucleosome positioning, frequently located at sites marked by acetylated histone H3 lysine 27 (H3K27ac), which establishes an open chromatin state with other transcription factors for transcriptional activation [729]. Enhancer of zeste homolog 2 (EZH2) is an enzymatic subunit of polycomb repressor complex 2 (PRC2) that negatively regulates the activity of the SWI/SNF complex by placing the repressive trimethylated histone H3 lysine 27 (H3K27me3) mark [729]. EZH2 is expressed in approximately one-third of epithelioid sarcomas [730], making EZH2 a promising target. Tazemetostat is an inhibitor of EZH2 that blocks the lysine methyltransferase activity of EZH2 by selectively binding to the S-adenosyl methionine (SAM) binding site of EZH2 [731] (Fig. 9h). Tazemetostat exhibits clinical activity with favorable safety in patients with advanced epithelioid sarcoma harboring *INI1* loss [732, 733].

Brain and other CNS tumors accounted for 1.6% of cancer cases and 2.5% of cancer-related mortalities worldwide in 2020 [2]. These tumors comprise over 100 histologically distinct subtypes with varying clinical characteristics, treatments, and outcomes, mainly including tumors of neuroepithelial tissue, cranial and spinal nerves, meninges, etc. [734].

#### Glioblastoma

Glioblastoma multiforme (GBM) is the most frequent and lethal subtype of brain cancer that originates in the CNS [735]. Compared with surrounding healthy tissue, brain cancers possess a more alkaline pH [736]. Temozolomide is a DNA alkylating prodrug stable at acidic pH values but labile at alkaline pH values [737] (Fig. 9i). The alkaline microenvironment within brain cancer preferentially facilitates the activation of temozolomide [736, 737]. It adds a methyl group to the O^6^ position of guanine (G), resulting in a methyl-guanine (meG)-to-thymine (T) mismatch during DNA replication instead of a G-to-cytosine (C) match [738], which leads to DNA damage and ultimately cell apoptosis [739, 740]. Temozolomide exhibits an acceptable safety profile and improves PFS compared with procarbazine in patients with GBM at first relapse [741]. Currently, temozolomide-containing regimens are still the first-line therapy for GBM [742, 743]. However, O^6^-meG methyltransferase (MGMT) removes alkyl groups from the O^6^ position of G, conferring resistance to temozolomide [744, 745].

#### Neuroblastoma

Neuroblastoma is a malignant embryonal tumor derived from primitive cells of the sympathetic nervous system [746, 747]. It is the most frequent and lethal solid tumor in children and is commonly associated with a poor overall prognosis [747]. Disialoganglioside GD2 is expressed almost uniformly on the surface of neuroblastoma cells and induces cell proliferation, invasion, and motility by activating RTK-mediated signal transduction [746, 748, 749], making it an effective and tractable target of neuroblastoma [750, 751]. Dinutuximab is a human/mouse chimeric GD2‑directed mAb that recognizes and binds to the sugar moiety of GD2 exposed to the extracellular milieu (similar to 14G2a antibody [752]), thereby inducing cell lysis through ADCC and CDC [751–753]. Compared with standard therapy (six cycles of 13-*cis*-retinoic acid), dinutuximab significantly improved clinical outcomes in combination with alternating granulocyte-macrophage colony-stimulating factor (GM-CSF) and IL-2 after standard therapy in patients with high-risk neuroblastoma [754]. However, anti-drug antibodies, including human anti-mouse or -chimeric antibodies, may cause treatment delays, terminations, or even abrogate the antitumor effects [746]. Naxitamab is designed to reduce the effects of anti-drug antibodies but enhance ADCC through humanized IgG1-Fc and retain complement-mediated cytotoxicity potency through its high affinity for GD2 [746, 755]. As expected, naxitamab exhibits modest toxic effects, low immunogenicity, and substantial anti-neuroblastoma activity in combination with GM-CSF in patients with relapsed or refractory high-risk neuroblastoma [756–758] (Fig. 9j).

#### Malignant pleural mesothelioma

Malignant pleural mesothelioma (MPM) is a rare but highly aggressive and lethal cancer that originates in the serosal outer linings of the lungs (pleurae), heart, abdomen, and testes, with a 5-year OS rate of ~ 5% [759, 760]. Folate receptors (FRα, FRβ, and FRγ) are cysteine-rich cell surface glycoproteins that mediate the cellular uptake of folate, commonly expressed at low levels in most normal tissues [761]. Folate-dependent one-carbon metabolism is required for the de novo synthesis of purines, thymidylate, and S-adenosyl methionine and is thus critical to DNA synthesis [762]. Nevertheless, FRα is highly activated and overexpressed in MPM tissues compared with normal adjacent tissues, making the folate–FRα delivery and metabolism system an attractive target for MPM treatment [763]. Pemetrexed is a multitargeted anti-folate agent that inhibits at least three enzymes (thymidylate synthase, dihydrofolate reductase, and glycinamide ribonucleotide formyltransferase) involved in folate metabolism and DNA synthesis [764] (Fig. 9k). Compared to cisplatin monotherapy, pemetrexed plus cisplatin therapy improved the mOS (12.1 months vs. 9.3 months) and was thus approved by the FDA for unresectable MPM treatment [765]. Most recently, compared with cisplatin-pemetrexed chemotherapy, durvalumab plus platinum-pemetrexed chemotherapy therapy significantly improved the mOS (20.4 months vs. 12.1 months) in patients with unresectable MPM [766].

#### NTRK-positive solid tumors

TRK proteins (including TRKA, TRKB, and TRKC) are encoded by the *NTRK* gene family (*NTRK1-3*), which are frequently fusion-positive in a broad range of solid tumors, including glioblastoma, NSCLC, and STSs. [767]. *NTRK* fusion leads to the constitutive activation of TRK protein, which acts as an oncogenic driver, making it a potential therapeutic target [768]. Larotrectinib is an oral, highly selective inhibitor of TRKs that binds to and competitively inhibits the ATP-binding site of TRKs [769] (Fig. 9l). Larotrectinib induces both cell apoptosis and inhibition of cell growth in TRK-overexpressed tumors [770] and exhibits encouraging antitumor activity with good tolerance in patients with tumors harboring *NTRK* gene fusions [771–773]. However, NTRK1^G595R^ and NTRK1^G667C^ mutations located in the catalytic domain confer resistance to both entrectinib and larotrectinib [117, 774]. Ponatinib and nintedanib (a PDGFR, FGFR, and VEGFR inhibitor used for idiopathic pulmonary fibrosis treatment) potentially overcome NTRK1^G667C^ mutation-induced resistance but not NTRK1^G595R^ mutation-induced resistance [774]. Moreover, the next-generation TRK inhibitors repotrectinib and LOXO-195 exhibit encouraging activity to overcome *TRK* mutation-induced resistance [775] (Table 8).

**Table 8:** FDA-approved therapeutic drugs for thyroid cancer and other solid tumors

Drug (brand name)	Sponsor	Properties	Indication	Approval date	Review
Thyrotropin alfa (Thyrogen)	Genzyme	Recombinant human thyroid-stimulating hormone	Thyroid cancer	11/30/1998	O
Lenvatinib mesylate (Lenvima)	Eisai	Multitarget TKI (VEGFRs, FGFRs, PDGFRα, RET, and KIT)	Thyroid cancer	02/13/2015	P, O
Vandetanib (Caprelsa)	Genzyme	Multitarget TKI (VEGFR2/3, EGFR, and RET)	Unresectable or metastatic medullary thyroid cancer	04/06/2011	P, O
Cabozantinib S-malate (Cometriq)	Exelixis	Multitarget TKI (VEGFRs, MET, RET, FLT3, KIT, TIE2, and AXL)	Progressive, metastatic medullary thyroid cancer	11/29/2012	P, O
Alitretinoin (Panretin)	Concordia	9-cis-retinoic acid, a form of vitamin A	AIDS-related Kaposi’s sarcoma	02/02/1999	P, O
Trabectedin (Yondelis)	Janssen	Alkylating drug	Liposarcoma or leiomyosarcoma	10/23/2015	P, O
Olaratumab (Lartruvo)	Eli Lilly	PDGFRα-directed mAb	Soft tissue sarcoma	10/19/2016	P, O
Pexidartinib HCl (Turalio)	Daiichi Sankyo	CSF1R, KIT, and FLT3 inhibitor	Tenosynovial giant cell tumor	08/02/2019	P, O
Tazemetostat (Tazverik)	Epizyme	EZH2 inhibitor	Epithelioid sarcoma	01/23/2020	P, O
Temozolomide (Temodar)	Merck	DNA alkylating agent	Glioblastoma	08/11/1999	P, O
Dinutuximab (Unituxin)	United Therap	GD2‑directed mAb	High-risk neuroblastoma	03/10/2015	P, O
Naxitamab (Danyelza)	Y-mAbs	GD2-directed mAb	High-risk neuroblastoma	11/25/2020	P, O
Pemetrexed disodium (Alimta)	Eli Lilly	Folate analog	Malignant pleural mesothelioma	02/04/2004	P, O
Larotrectinib sulfate (Vitrakvi)	Bayer	TRKs inhibitor	NTRK-positive solid tumors	11/26/2018	P, O

*AIDS* Acquired immunodeficiency syndrome; *CSF1R* Colony-stimulating factor-1 receptor; *EGFR* Epidermal growth factor receptor; *EZH2* Enhancer of zeste homolog 2; *FLT3* FMS-like tyrosine kinase 3; *MEK1/2* MAPK/ERK kinase 1/2; *MET* Mesenchymal–epithelial transition gene; *NTRK* Neurotrophic receptor tyrosine kinase; *O* Orphan; *P* Priority; *PDGFRα* Platelet-derived growth factor receptor *α*; *RET*: rearranged during transfection gene; *S* Standard; *TRKs* Tropomyosin receptor kinases; *VEGFR* Vascular endothelial growth factor receptor

### The success and dilemma of current antitumor strategies

**Fig. 10:**
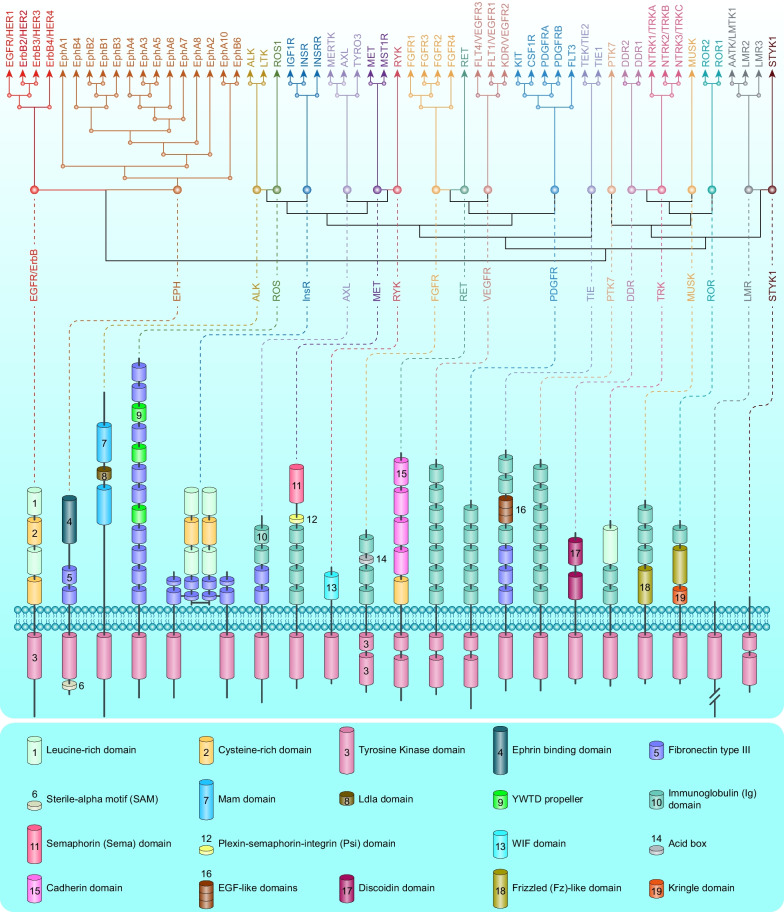
RTK families (adapted from [776])

RTK inhibitors and immune checkpoint blockades (ICBs) have undoubtedly been the most successful antitumor drugs in the past 31 years. The human RTK family comprises 58 RTK proteins, which fall into 20 subfamilies [776]. These RTKs share a similar structure, mainly with ligand-binding domains in the extracellular region, a single transmembrane helix, and a tyrosine kinase domain in the cytoplasmic region [776] (Fig. 10). Aberrant overexpression and oncogenic gain-of-function mutation-induced ligand-independent activation of RTKs frequently leads to the activation of downstream pathways, resulting in various diseases involving cancers, diabetes, inflammation, etc. RTK-targeted therapies can occur at three levels: blocking the ligand–RTK interaction in the extracellular region, inhibiting the tyrosine kinase domain in the intracellular region, and inhibiting the constitutive components of RTK-mediated downstream pathways.

**Fig. 11:**
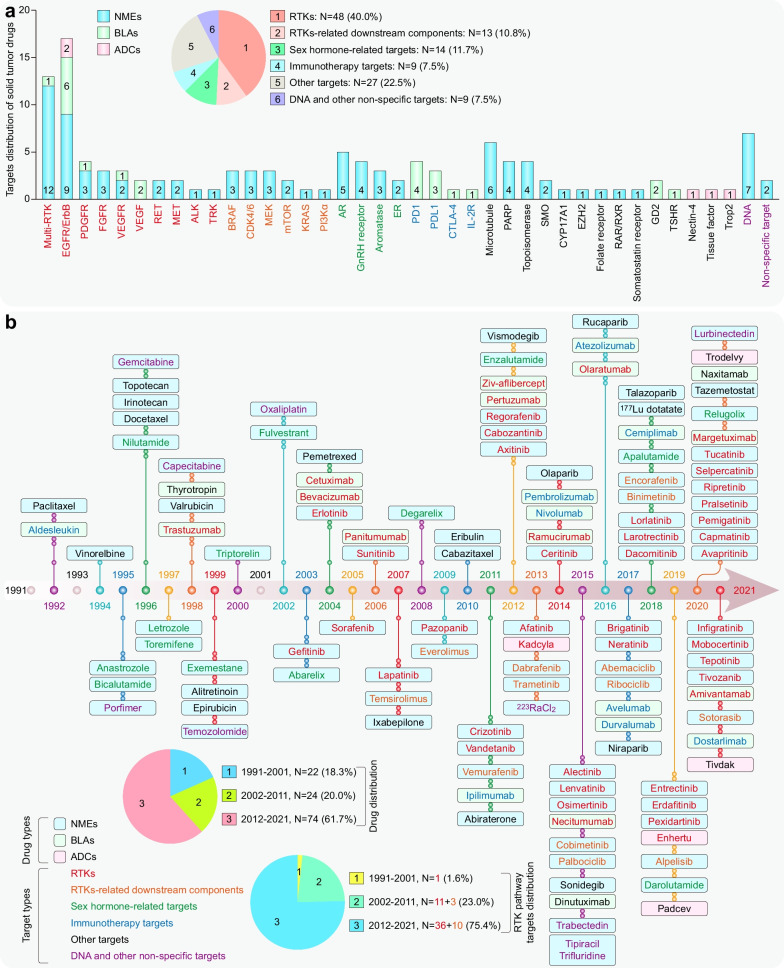
Distribution of therapeutic drugs according to targets and approval years. **a** Distribution of therapeutic drugs according to targets. **b** Distribution of therapeutic drugs according to approval years

The advent of trastuzumab is undoubtedly a milestone. It inhibited the RTK pathway from the first level and was the first RTK-targeted therapy. However, obstacles to trastuzumab–HER2 interaction [201] and reactivation of HER2 downstream pathways, whether induced by bypass pathway switching or mutations of downstream components (e.g., *PIK3CA* mutation [777]), confer resistance to trastuzumab. Regarding other RTKs, such as MET, oncogenic mutations lead to MET self-activation in a ligand-independent manner [778]. These biological mechanisms inevitably lead to the failure of the first-level RTK-targeted strategy. Gefitinib is a small-molecule inhibitor that targets the intracellular tyrosine kinase domain of RTK at the second level because it is difficult for antibodies to target intracellular antigens [779]. This strategy addresses the ligand-dependent activation and specific mutation-induced self-activation of RTK to a certain extent. However, it cannot overcome the bypass pathway switch, secondary mutations within the tyrosine kinase domain, and downstream component mutations, even if multitarget TKIs (*e.g.*, sorafenib) are used. Inhibitors of the RAS–RAF–MEK–MAPK/ERK (*e.g.*, sotorasib) and PI3K–AKT–mTOR (*e.g.*, alpelisib) pathways block the RTK pathway at the third level. This strategy blocks the RTK pathway regardless of upstream RTK activation and may address the bypass pathway switch to a certain extent. However, the secondary mutations of targets and loss-of-function *PTEN* mutations still confer resistance [124, 780]. Nevertheless, RTK-targeted drugs have been the mainstay for the treatment of solid tumors. Over the past 31 years, 48 RTK inhibitors and 13 RTK downstream component inhibitors were approved by the FDA, and these drugs account for more than half of all therapeutic drugs for solid tumors (Fig. 11a).

ICBs adopt a novel strategy that reinvigorates a range of CD4^+^ and CD8^+^ tumor-infiltrating T lymphocytes [781], enabling the possibility of long-term survival in patients with metastatic or advanced cancers [782]. The clinical application of ICBs heralds a new era of cancer treatment, as they are the most successful strategy in the recent decade [782, 783]. The FDA has approved nine ICBs in the USA since the first approval of ipilimumab in 2011 (Additional file 1: Table S4, page 56). Despite the clinical success, only a minority of people exhibit durable responses to ICBs [784]. The mutated proteins of cancer cells produced by nonsynonymous mutations and other genetic alterations need to be processed and then presented as neoantigens by major histocompatibility complex (MHC) molecules of antigen-presenting cells (APCs) and recognized by T cells [785]. However, neoantigens do not always bind to MHC molecules with high affinity or contain mutant amino acids at the appropriate position, making it difficult for T cells to recognize them [784]. Melanoma has the highest frequency of somatic mutations among human cancers and may produce the largest available neoantigen repertoire [785]. It explains why most ICBs are approved for the treatment of melanoma. In addition, preexisting PD1/PDL1-positive CD4^+^ and CD8^+^ T cells positioned in proximity to the cancer cells inside tumors are critical to clinical responses [786, 787]. In some tumors with an ‘immune-excluded’ phenotype, the T cells locate the stroma surrounding the tumor nest instead of penetrating the parenchyma of the tumor. Tumors with the ‘immune-desert’ phenotype lack T cells in either the parenchyma or stroma of the tumor [784]. Thus, tumors with immune-excluded and immune-desert phenotypes are often associated with unfavorable responses to ICBs [783]. In addition, immune-related adverse events (irAEs) [788] and hyperprogressive disease [5, 789] are of great concern. It is clear that further work is needed to reliably regulate the immune system in the clinic.

Breast and prostate cancers are associated with sex hormones and accounted for approximately one-fifth of cancer cases and more than 10% of cancer-related mortalities worldwide in 2020 [2]. Breast cancer drugs are frequently at the forefront of advances in cancer treatment and diagnosis [133]. Therapeutic drugs for breast cancer have begun to diversify, and no new drugs targeting ER (two SERDs bazedoxifene and ospemifene, are not indicated for breast cancer, Additional file 1: Table S1, page 26) or aromatase have been approved since the approval of fulvestrant in 2002. ER-positive breast cancer accounts for 80% of all breast cancer cases and half of breast cancer-related mortalities [152, 790]. Given the superiority of fulvestrant, newer-generation ER antagonists are needed to improve the poor physicochemical properties and administration mode of fulvestrant for this large group of patients [152].

In contrast, therapeutic drugs for prostate cancer are still limited to antiandrogens, even in recent years. The progression of mCRPC is the major cause of death in patients with prostate cancer [791], although OS is significantly improved with cabazitaxel [477, 478], abiraterone [486], and enzalutamide [792]. Bipolar androgen therapy (BAT) is a new strategy that induces rapid cycling between high and low serum testosterone concentrations, resulting in tumor responses and resensitization of mCRPC to enzalutamide. This strategy is more effective than abiraterone [793, 794]. Distinct strategies have been developed for the two sex hormone-related cancers; specifically, breast cancer treatment adopts strategies referring to multiple targets and mechanisms, while prostate cancer treatment emphasizes the refinement of antiandrogen strategies.

Therapeutic drugs for solid tumors have ushered in a new period of prosperity. Seventy-four therapeutic drugs and 61 RTK or RTK pathway inhibitors were approved in the last decade, accounting for 61.7% and 75.4% of all therapeutic drugs and RTK or RTK pathway inhibitors of solid tumors approved in the past 31 years, respectively (Fig. 11b). Quite a few drugs have been exquisitely designed. For instance, ziv-aflibercept utilizes the binding affinity between VEGFRs and VEGFs to capture VEGFs. In addition, ADCs retain all the antitumor efficiency of mAbs and add cytotoxic payloads, allowing for the targeted delivery of chemotherapeutic agents. The application of ADCs has dramatically expanded the clinical application of mAbs. ADCs and the first bispecific antibody, amivantamab, have started a new era of engineered antibodies. The approval of SMO, PARP, and EZH2 inhibitors was based on research progress on hedgehog signaling, synthetic lethality, and epigenetics in cancers. It is believed that there will be more drugs based on new mechanisms in the future alongside the exploration of new targets and vulnerabilities of tumors.

### Future perspectives

**Fig. 12:**
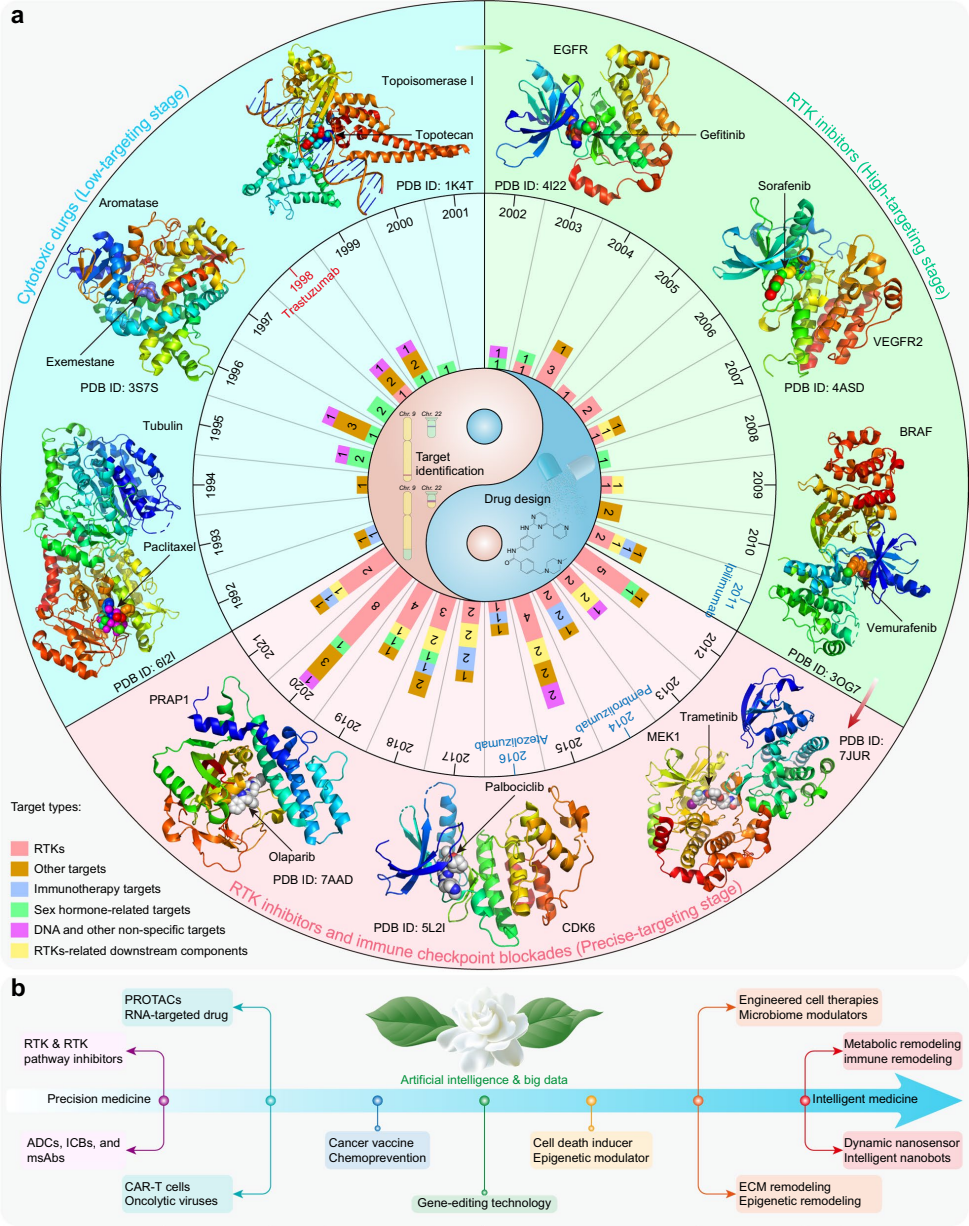
Achievements in the past 30 years and future perspectives. **a** The achievements in the past 30 years (obtained from www.rcsb.org). **b** Future perspectives of oncology drug development (compositional element was obtained from https://www.16pic.com)

Target identification and drug design have been the core drivers throughout antitumor history in recent decades, and antitumor strategies for solid tumors have profoundly changed over the past 30 years. During the first decade, pharmacologists were devoted to developing anti-endocrine agents, microtubule inhibitors, DNA alkylating agents, and DNA topoisomerase inhibitors. Overall, this stage did not focus much on targeting drugs, although the advent of trastuzumab began a new era of RTK-targeted therapy. During the second decade, pharmacologists extended RTK-targeted inhibitor studies to include RTK downstream component inhibitors, which enriched the TKI library and shifted the focus toward targeted drug development. In addition, the advent of ipilimumab, which converts immunotherapy from positive stimulation (*e.g*., IL-2 and INFα) to immune checkpoint blockade, started a true paradigm shift for metastatic or advanced solid tumors. During the third decade, RTK and RTK pathway inhibitors and ICBs were extensively developed. Drugs targeting novel targets and tumor vulnerabilities, such as PARP and SMO inhibitors, were added to the list for solid tumor treatment. KRAS^G12C^, once considered an undruggable target, was blocked successfully by sotorasib. The treatment of solid tumors ushered in the precise targeting stage (Fig. 12a).

RTK and RTK pathway inhibitors, ADCs and ICBs, are still the mainstay. A new ICB relatlimab-rmbw (lymphocyte activation gene-3 (LAG-3)-directed mAb) was approved by the FDA in combination with nivolumab for unresectable or metastatic melanoma on March 18, 2022 [795]. Next, proteolysis-targeting chimeras (PROTACs) [796] and small interfering RNA (siRNA) technologies [797] degrade targets at the protein and RNA levels, respectively [798]. Indeed, the first RNA-targeted drug, inclisiran (Additional file 1: Table S1, page 40), has been approved by the FDA [799]. Increasing clinical trials of PROTAC-based drugs are ongoing, making PROTACs the gold rush [800]. The advent of PROTAC technology makes it possible to selectively degrade proteins that are typically difficult to target (*e.g*., transcription factors). Similar technologies, such as chaperone-mediated autophagy [801], Trim-Away [802], degradation tag (dTAG) [803], and lysosome-targeting chimeras (LYTACs) [804], are also of great concern. Multispecific antibodies (msAbs) bind two or more epitopes, which greatly extends the function of mAbs. With the approval of bispecific T-cell engagers (BiTEs) blinatumomab (Additional file 1: Table S1, page 28; Table S2, page 47) and amivantamab, msAbs will become a critical antitumor strategy in the coming decades [805].

Vaccines, cell-based therapies, and gene therapy products represent another essential pillar of cancer treatment, although they are not discussed in the text. Chimeric antigen receptor (CAR)-T cells have achieved great success in patients with hematological malignancies, especially CD19-directed CAR-T cells [806]. The clinical application of CAR-T cells in solid tumors has been limited by setbacks due to substantive biological barriers and risks [807]. Efforts to seek suitable targets [808] to overcome the immunosuppressive tumor microenvironment (TME) [809] and combat cytokine release syndrome (CRS) and immune effector cell-associated neurotoxicity syndrome (ICANS) [810, 811] are still ongoing. In recent years, CAR-T-cell clinical trials against solid tumors have exhibited acceptable safety and encouraging clinical outcomes [812–815]. Oncolytic viruses are naturally or genetically engineered viruses that preferentially infect, lyse, and replicate in cancer cells relative to normal cells [816, 817]. Oncolytic viruses provide a platform for monotherapy [818] or in combination with chemotherapies [819, 820] and immunotherapies [821–823] by delivering defined factors. With the first approval of talimogene laherparepvec (T-Vec) in 2015 [824], many clinical trials are ongoing [816]. Oncolytic viruses are also attractive carriers for cancer vaccines [825] and gene editing [826].

Cancer vaccines can be simply divided into preventive and therapeutic vaccines [827]. Therapeutic vaccines directly utilize APCs (*e.g.*, dendritic cells (DCs)), viruses, liposomes, and nanoparticles as vesicles to deliver tumor-specific antigens (including neoantigens), inducing immune recognition and activation of T cells [828]. Preventive vaccines are confined to specific virus-induced cancers, such as HPV-related cancers [829] and hepatitis B virus (HBV)-related HCC [830]. Like preventive vaccines, chemoprevention is also a preventive strategy to reverse, suppress, or prevent carcinogenic progression to invasive cancer using chemical agents [831]. For instance, familial adenomatous polyposis (FAP) is a precancerous state of colorectal cancer [832] caused by germline mutations in the adenomatous polyposis coli (*APC*) gene [833, 834]. Almost all of the mutations of *APC*, both germline and somatic, produce a truncated APC protein, leading to APC dysfunction [835–837]. Dysfunctional APC fails to form a destruction complex, resulting in β-Catenin stabilization and canonical Wnt/β-Catenin signaling activation [838]. Cyclooxygenase-2 (COX-2) is a crucial enzyme of prostaglandin E_2_ (PGE_2_) biosynthesis that plays an essential role in colorectal tumorigenesis [839]. PGE_2_ is a potent proinflammatory factor that serves as a ligand for the G protein-coupled receptor (GPCR) EP2. It promotes colon cancer cell growth through the Gα_s_-Axin-β-Catenin axis [840]. Celecoxib is a potent COX-2 inhibitor approved by the FDA in 1998 for treating FAP (Additional file 1: Table S1, page 10); thus, it is also an agent for the chemoprevention of colorectal cancer [841, 842]. From the cost-effectiveness perspective, preventive vaccines and chemoprevention have absolute superiorities, both economically and physiologically.

Cell death inducers have always been an important research field in cancer treatment strategies [843]. Mechanically, the available antitumor drugs induce cell cycle arrest or cell death unexceptionally. For instance, the mTOR inhibitors (*e.g.*, temsirolimus and everolimus) can be classified as autophagy-related death inducers. In recent years, novel cell death inducers, such as the tumor necrosis factor-related apoptosis-inducing ligand (TRAIL) agonist eftozanermin alfa (ABBV-621) [844] and the mitochondrial caseinolytic protease P (ClpP) activator ONC201 [845] have entered clinical trials for the treatment of solid tumors (NCT03082209 and NCT05476939), which may bring new hope for cancer treatment. In contrast, significant success has been achieved in the field of epigenetic drugs (epi-drugs), such as EZH2 inhibitor tazemetostat and isocitrate dehydrogenase 1 (IDH1) inhibitor ivosidenib (Additional file 1: Table S1, page 34). The first- and second-generation epi-drugs that use a ‘one size fits all’ strategy, such as DNA methyltransferase (DNMT) and histone deacetylase (HDAC) inhibitors, were proven to have disappointing efficacy in patients with solid tumors [846]. The third-generation epi-drugs use more precise targets, such as IDH1, EZH2, and certain bromodomain and extra-terminal domain (BET)-containing proteins (BRDs), which are showing promising efficacy [847].

Artificial intelligence (AI) improves the ability to deal with the massive amount of tumor genome information and promotes the ability to decipher protein structures. The AI technology represented by AlphaFold may significantly shorten the process of drug development [848]. In addition, gene-editing technologies, such as clustered regularly interspaced short palindromic repeats (CRISPR)-associated (Cas) systems [849], provide a potent tool to modify primary patient-derived cells in vitro. Quite a few clinical trials of CRISPR-based immune cells for cancer treatment are ongoing, especially CAR-T cells [850]. However, multiple hurdles need to be overcome before CRISPR directly targets tumor cells in vivo, including appropriate delivery carriers, off-target cutting, and chromosomal rearrangements [850, 851]. Modifying specific mutations by gene-editing technologies is undoubtedly one of the peaks of precision medicine. Engineered cell therapies should not be limited to the currently used T cell or DC cell models; many other cell types can also be incorporated into this system, such as stem cells [852, 853], natural killer (NK) cells [854], fibroblasts [855], and even engineered cancer cells [856]. Expanding the variety of cell types available for therapy can make full use of the characteristics of different cells to meet complex clinical needs [857]. Human microbiome communities have been implicated in cancer initiation, progression, metastasis, and therapy resistance [858]. With the advent of next-generation sequencing and a deeper understanding of host–microbiome interactions, microbiome analyses are being developed as a promising approach for cancer diagnosis [859], while microbiome modulation may be a practicable adjunct to existing antitumor strategies [860].

High tumor heterogeneity and tumor mutation burden are frequently associated with treatment resistance and cancer recurrence [861], failing to predict the response to available treatment [862]. For these clinical settings, systematic manipulation and domestication of cancer cells by extracellular matrix (ECM) and epigenetic remodeling or by more complicated metabolic and immune remodeling to control the progression and metastasis of tumors instead of killing tumors may be realistic strategies in the post-precision medicine era. With the development of AI and nanotechnology, the existing approaches to diagnosis and treatment will be replaced by dynamic nanosensors and intelligent nanobots, thereby promoting the transition from precision medicine to intelligent medicine (Fig. 12b).

## Conclusion

The research and development pace of antitumor drugs is accelerating with the in-depth study of the tumorigenesis mechanism. Nevertheless, these 120 therapeutic drugs are still the mainstay for advanced, unresectable, or metastatic solid tumors. Although several drugs have been discontinued or withdrawn from the market due to severe adverse effects, commercial reasons, or the emergence of substituted new-generation drugs, the findings and lessons in the exploration of cancer treatment strategies will always be the milestones in antitumor history.

## Supplementary Information

**Additional file 1**. **Table S1.** FDA-approved drugs. **Table S2.** FDA-approved cancer drugs. **Table S3.** FDA-approved therapeutic drugs for solid tumors. **Table S4.** ICBs: first approval and primary indications in the USA and China.

## Data Availability

Not applicable.

## Abbreviations

^177^Lu: Lutetium-177
^223^Ra: Radium-223
5′DFUR: 5′-Deoxy-5-fluorouridine
5-FU: 5-Fluorouracil
ADCC: Antibody-dependent cellular cytotoxicity
ADCs: Antibody-drug conjugates
ADT: Androgen deprivation therapy
AI: Artificial intelligence
AIDS: Acquired immunodeficiency syndrome
ALK: Anaplastic lymphoma kinase
APC: Antigen-presenting cell or adenomatous polyposis coli
AR: Androgen receptor
AREs: Androgen response elements
BAT: Bipolar androgen therapy
BCG: Bacillus Calmette-Guérin
BER: Base excision repair
BET: Bromodomain and extra-terminal domain
BiTEs: Bispecific T cell engagers
BLAs: Biologics license applications
BLBC: Basal-like breast cancer
BRDs: BET-containing proteins
CAR: Chimeric antigen receptor
CDC: Complement-dependent cytotoxicity
CDK4/6: Cyclin-dependent kinases 4/6
CIS: Carcinoma in situ
ClpP: Caseinolytic protease P
CNS: Central nervous system
COX-2: Cyclooxygenase-2
CRISPR: Clustered regularly interspaced short palindromic repeats
CRS: Cytokine release syndrome
CSCC: Cutaneous squamous cell carcinoma
CSF1: Colony-stimulating factor-1
CSF1R: CSF1 receptor
CTLA4: Cytotoxic T lymphocyte antigen 4
CYP17A1: Cytochrome P450 17A1
DCs: Dendritic cells
DFS: Disease-free survival
DHT: Dihydrotestosterone
dMMR: Mismatch repair deficiency
DNMT: DNA methyltransferase
DSBs: Double-stranded breaks
dTAG: Degradation tag
DTC: Differentiated thyroid cancer
ECM: Extracellular matrix
EGFR: Epidermal growth factor receptor
EML4: EMAP-like protein 4
epi-drugs: Epigenetic drugs
ER: Estrogen receptor
ERK: Extracellular signal-regulated kinase
EZH2: Zeste homolog 2
FAP: Familial adenomatous polyposis
FDA: Food and Drug Administration
FdUMP: Fluorodeoxyuridine monophosphate
FdUTP: Fluorodeoxyuridine triphosphate
FGFR2: Fibroblast growth factor receptor 2
FKBP12: FK506-binding protein 12
FR: Folate receptor
FSH: Follicle-stimulating hormone
FUTP: Fluorouridine triphosphate
GATA3: GATA binding protein 3
GBM: Glioblastoma multiforme
GEP-NETs: Gastroenteropancreatic neuroendocrine tumors
GISTs: Gastrointestinal stromal tumors
GLI: Glioma-associated oncogene
GM-CSF: Granulocyte-macrophage colony-stimulating factor
GnRH: Gonadotropin-releasing hormone
gp100: Glycoprotein 100
GPCR: G protein-coupled receptor
H3K27ac: Acetylated histone H3 lysine27
H3K27me3: Trimethylated histone H3 lysine27
HBV: Hepatitis B virus
HCC: Hepatocellular carcinoma
HDAC: Histone deacetylase
HER2: Epidermal growth factor receptor 2
HGF: Hepatocyte growth factor
HIF1α: Hypoxia-inducible factor-1α
HPV: Human papillomavirus
HR: Hormone receptor
HRD: Homologous recombination deficiency
HSP90: Heat shock protein 90
ICANS: Immune effector cell-associated neurotoxicity syndrome
ICBs: Immune checkpoint blockades
ICC: Intrahepatic cholangiocarcinoma
IDH1: Isocitrate dehydrogenase 1
IFNα: Interferon-α
IFNγ: Interferon-γ
Ig: Immunoglobulin
IGF1R: Insulin-like growth factor-1 receptor
IL-2: Interleukin-2
IL-2R: IL-2 receptor
irAEs: Immune-related adverse events
KSHV: Kaposi’s sarcoma-associated herpesvirus
LAG-3: Lymphocyte activation gene-3
LAK: Lymphokine-activated killer
LBD: Ligand-binding domain
LCC: Large-cell carcinoma
LH: Luteinizing hormone
LH-RH: Luteinizing hormone-releasing hormone
LYTACs: Lysosome-targeting chimaeras
mAbs: Monoclonal antibodies
MAPK: Mitogen-activated protein kinase
MASC: Mammary analog secretory carcinoma
mCRPC: Metastatic castration-resistant prostate cancer
MC-vc-PAB: Maleimidocaproyl valine-citrulline p-aminobenzyl alcohol carbamate
MDR: ABCG2-mediated multidrug resistance
MEK: MAPK/ERK kinase
MET: Mesenchymal–epithelial transition
MGMT: O^6^-meG methyltransferase
MHC: Major histocompatibility complex
mLST8: Mammalian lethal with SEC13 protein 8
MMAE: Monomethyl auristatin E
MPM: Malignant pleural mesothelioma
MRP2: Multidrug resistance-associated protein 2
msAbs: Multispecific antibodies
MSI-H: Microsatellite instability—high
MTC: Medullary thyroid cancer
mTOR: Mammalian target of rapamycin
mTORC: MTOR complex
NAD^+^: Nicotinamide adenine dinucleotide
NER: Nucleotide excision repair
NIS: Sodium–iodide symporter
NK: Natural killer
NMEs: New molecular entities
NMPA: National Medical Products Administration
NMSCs: Non-melanoma skin cancers
NSCLC: Non-small-cell lung cancer
NTRK: Neurotrophic tyrosine receptor kinase
OCT: Organic cation transporter
ORR: Objective response rate
OS: Overall survival
PARPs: Poly (ADP-ribose) polymerases
PAR: Poly (ADP-ribose)
PAR-2: Protease-activated receptor 2
PD1: Programmed death receptor-1
PDGFRα/β: Platelet-derived growth factor receptor α/β
PDL1: Programmed death-ligand 1
PFS: Progression-free survival
PGE_2_: Prostaglandin E_2_
PI3K: Phosphatidylinositol 3-kinase
PIK3CA: Phosphatidylinositol 3-kinase catalytic subunit A
PlGF: Placenta growth factor
PR: Progesterone receptor
PRC2: Polycomb repressor complex 2
PROTACs: Proteolysis-targeting chimeras
PRRT: Peptide receptor radionuclide therapy
PSA: Prostate-specific antigen
PTCH1: Patched homolog 1
RAPTOR: Regulatory-associated protein of mTOR
RARγ: Retinoic acid receptor γ
RB: Retinoblastoma
RB3-SLD: RB3 protein stathmin-like domain
RCC: Renal cell carcinoma
RET: Rearranged during transfection
rhTSH: Recombinant human TSH
RICTOR: Rapamycin-insensitive companion of mTOR
ROS: Reactive oxygen species
ROS1: ROS proto-oncogene 1
RTKs: Receptor tyrosine kinases
RXRα: Retinoid X receptor α
SAM: S-adenosyl methionine
SERD: Selective ER degrader/down-regulator
SERMs: Selective ER modulators
SCC: Squamous cell carcinoma
SCLC: Small-cell lung cancer
siRNA: Small interfering RNA
SMCC: N-succinimidyl-4-(N-maleimidomethyl) cyclohexane-1-carboxylate
SMO: Smoothened
SSBs: DNA single-stranded breaks
SSTRs: Somatostatin receptors
SWI/SNF: Switch/sucrose non-fermentable
T-Vec: Talimogene laherparepvec
TF: Tissue factor
TIIs: Tumor-infiltrating immune cells
TIMCs: Tumor-infiltrating mononuclear cells
TKIs: Tyrosine kinase inhibitors
TME: Tumor microenvironment
TNBC: Triple-negative breast cancer
TOP1: Topoisomerase I
TOP1CCs: TOP1 cleavage complexes
TOP2A: Topoisomerase Iiα
TP: Thymidine phosphorylase
TRAIL: Tumor necrosis factor-related apoptosis inducing ligand
TRKs: Tropomyosin receptor kinases
Trop-2: Trophoblastic cell surface antigen-2
TSH: Thyroid-stimulating hormone
UGT1A1: Uridine diphosphate glucuronosyltransferase 1A1
UP: Uridine phosphorylase
VEGFR2: Vascular endothelial growth factor receptor 2
VEGFs: Vascular endothelial growth factors
VHL: Von Hippel–Lindau; WT: Wild type
