# Supplementary material for: Small-molecule inhibitors, immune checkpoint inhibitors, and more: FDA-approved novel therapeutic drugs for solid tumors from 1991 to 2021
Source: J Hematol Oncol. 2022 Oct 8;15:143. doi: 10.1186/s13045-022-01362-9 (PMC9548212; doi:10.1186/s13045-022-01362-9)
Supplement: Supplementary file 1 — Additional file 1. Table S1. FDA-approved drugs. Table S2. FDA-approved cancer drugs. Table S3. FDA-approved therapeutic drugs for solid tumors. Table S4. ICBs: first approval and primary indications in the USA and China. [file 13045_2022_1362_MOESM1_ESM.pdf]

Supplementary data: Table 1 – FDA-approved drugs

	Drug (brand name)	Sponsor	Properties	Indication	Approval date	Review
	**1991**	**New molecular entities = 28**				
1	Ondansetron HCl (Zofran)	Novartis	5-HT_3 receptor antagonist	Nausea and vomiting caused by radiotherapy, anesthesia, surgery or cytotoxic chemotherapy drugs	01/04/1991	P
2	Gallium nitrate (Ganite)	Chapter 7 Trustee	Bone-resorption inhibitor	Hypercalcemia of malignancy	01/17/1991	P, O
3	Ramipril (Altace)	King Pharms	Angiotensin-converting enzyme (ACE) inhibitor	Hypertension	01/28/1991	S
4	Succimer (Chemet)	Recordati Rare	Analog of dimercaprol, binds to heavy metals and forms a water-soluble complex	Poisoning by lead and other heavy metals	01/30/1991	P, O
5	Etodolac (Lodine)	Wyeth Pharms	Cycooxygenase-2 (COX-2) inhibitor	Osteoarthritis, rheumatoid arthritis and control acute pain	01/31/1991	S
6	Doxacurium chloride (Nuromax)	AbbVie	Neuromuscular-blocking drug or skeletal muscle relaxant	Adjunctively in anesthesia for endotracheal intubation or to facilitate mechanical ventilation	03/07/1991	S
7	Fludarabine phosphate (Fludara)	Genzyme Corp	DNA synthesis inhibitor	B-cell chronic lymphocytic leukemia	04/18/1991	P
8	Fosinopril sodium (Monopril)	Bristol-Myers Squibb	Angiotensin-converting enzyme (ACE) inhibitor	Hypertension	05/16/1991	S
9	Benazepril HCl (Lotensin)	Validus Pharms	Angiotensin-converting enzyme (ACE) inhibitor	Hypertension	06/25/1991	S
10	Felodipine (Plendil)	AstraZeneca	Voltage-gated L-type calcium channel stabilizer	Hypertension	07/25/1991	S
11	Foscarnet sodium (Foscavir)	Clinigen Hlthcare	Virus-specific DNA polymerase inhibitor	Cytomegalovirus retinitis in patients with AIDS	09/27/1991	P
12	Didanosine (Videx)	Bristol-Myers Squibb	HIV-1 reverse transcriptase inhibitor	HIV-1 infection	10/09/1991	P
13	Pentostatin (Nipent)	Hospira	Purine analog, irreversible inhibitor of adenosine deaminase	INFα-refractory hairy cell leukemia	10/11/1991	P, O
14	Pravastatin sodium (Pravachol)	Bristol-Myers Squibb	HMG CoA reductase inhibitor	Hypercholesterolemia	10/31/1991	S
15	Ticlopidine HCl (Ticlid)	Roche Palo	Adenosine diphosphate receptor inhibitor	Prevention of strokes	10/31/1991	P
16	Pamidronate disodium (Aredia)	Novartis	Bone-resorption inhibitor	Hypercalcemia of malignancy	10/31/1991	P
17	Clarithromycin (Biaxin)	AbbVie	Binds to 23s rRNA (with nucleotides in domains II and V) and inhibits the protein synthesis in bacteria	Broad range of infections	10/31/1991	P
18	Azithromycin (Zithromax)	Pfizer	Inhibits bacterial protein synthesis by binding to the 50S ribosomal subunit of the bacterial 70S ribosome	Bacterial infections	11/01/1991	P
19	Quinapril HCl (Accupril)	Pfizer Pharms	Angiotensin-converting enzyme (ACE) inhibitor	Hypertension	11/19/1991	S
20	Flumazenil (Romazicon)	Roche	Imidazobenzodiazepine derivative and benzodiazepine receptor antagonist	Reversal of the sedative effects of benzodiazepines	12/20/1991	P
21	Simvastatin (Zocor)	Organon	HMG CoA reductase inhibitor	Hyperlipidemia	12/23/1991	S
22	Cefprozil (Cefzil)	Corden Pharma	β-lactam antibiotic, inhibits bacterial cell wall synthesis by binding to specific PBPs	Mild to moderate infections of upper/lower respiratory tract and uncomplicated skin/skin-structure infections	12/23/1991	S
23	Nabumetone (Relafen)	Smithkline Beecham	Cycooxygenase-1/2 (COX-1/2) inhibitor	Osteoarthritis and rheumatoid arthritis	12/24/1991	S
24	Histrelin acetate (Supprelin)	Shire	Gonadotropin-releasing hormone (GnRH) agonist	Children with central precocious puberty (CPP)	12/24/1991	P
25	Isosorbide mononitrate (Ismo)	Promius Pharma	Soluble guanylate cyclase stimulator	Acute and chronic angina pectoris, hypertension, and myocardial infarction	12/30/1991	S
26	Sertraline HCl (Zoloft)	Upjohn	Serotonin reuptake inhibitor	Major depressive disorder, obsessive-compulsive disorder, panic disorder, and social anxiety disorder	12/30/1991	S
27	Enoxacin (Penetrex)	Sanofi	Quinolone/fluoroquinolone antibiotic, inhibits bacterial DNA gyrase and topoisomerase IV	Urinary tract infections and gonorrhea	12/31/1991	S
28	Loracarbef (Lorabid)	King Pharms	β-lactam antibiotic, inhibits bacterial cell wall synthesis by binding to specific PBPs	Mild to moderate infections caused by susceptible strains of the designated microorganisms	12/31/1991	S
	**1991**	**Biologics license applications = 4**				
1	Filgrastim (Neupogen)	Amgen	Recombinant human granulocyte colony-stimulating factor (G-CSF)	Severe neutropenia in patients with non-myeloid malignancies	02/20/1991	N/A
2	Sargramostim (Leukine)	Berlex Labs	Recombinant human granulocyte macrophage colony-stimulating factor (GM-CSF)	Myeloid reconstitution after PBPC/bone marrow transplantation, or with NHL, ALL, HL	03/05/1991	O
3	Aiglucerase (Ceredase)	Genzyme	Modified human β-glucocerebrosidase enzyme	Type 1 Gaucher’s disease	04/05/1991	O
4	Beractant (Survanta)	AbbVie	Natural bovine pulmonary surfactant	Respiratory distress syndrome in premature infants	07/01/1991	O

Supplementary data: Table 1 – FDA-approved drugs (Continued)

	Drug (brand name)	Sponsor	Properties	Indication	Approval date	Review
	**1992**	**New molecular entities = 26**				
1	Mivacurium chloride (Mivacron)	AbbVie	Cholinergic receptor inhibitor	Adjunctively in anesthesia for endotracheal intubation or to facilitate mechanical ventilation	01/22/1992	P
2	Temafloxacin HCl (Omniflox)	Abbott	Fluoroquinolone antibiotic	Systemic bacterial infections	01/30/1992	N/A
3	Lomefloxacin HCl (Maxaquin)	Pharmacia	Fluoroquinolone antibiotic	Respiratory tract, genital, urinary, and skin infections	02/21/1992	S
4	Finasteride (Proscar)	Merck & Co	Steroid type II 5α reductase inhibitor	Benign prostatic hyperplasia	06/19/1992	P
5	Zalcitabine (Hivid)	Roche	Nucleoside analog, HIV-reverse transcriptase inhibitor	HIV infection	06/19/1992	P, O
6	Teniposide (Vumon)	HQ spclt Pharma	Topoisomerase II inhibitor	Refractory childhood acute lymphoblastic leukemia	07/14/1992	P, O
7	Halofantrine HCl (Halfan)	GlaxoSmithKline	Blood schizonticidal antimalarial agent, binds to haemoglobin degrading enzyme unique to the malarial parasites	Malaria	07/24/1992	P, O
8	Bisoprolol fumarate (Zebeta)	Teva	Cardioselective β1-adrenergic blocking agent	Cardiovascular diseases such as hypertension, coronary heart disease, arrhythmias, ischemic heart diseases, and myocardial infarction after the acute event	07/31/1992	S
9	Cefpodoxime proxetil (Vantin)	Pharmacia & Upjohn	Inhibits bacterial cell wall synthesis by binding PBPs	Mild to moderate infections caused by susceptible strains of the designated microorganisms	08/07/1992	S
10	Masoprocol (Actinex)	Univ AZ Cancer Ctr	5-lipoxygenase inhibitor	Actinic keratoses	09/04/1992	S
11	Itraconazole (Sporanox)	Janssen Pharms	Inhibits the cytochrome P450-dependent synthesis of ergosterol	Fungal infections	09/11/1992	P
12	Desflurane (Suprane)	Baxter Hlthcare	May act on the lipid matrix of the neuronal membrane	Induction and/or maintenance of anesthesia	09/18/1992	S
13	Oxaprozin (Daypro)	Pfizer	Cycooxygenase-1 (COX-1) inhibitor	Rheumatoid arthritis, osteoarthritis, dysmenorrhea, and to alleviate moderate pain	10/29/1992	S
14	Sotalol HCl (Betapace)	Covis	β1- and β2-adrenergic receptor inhibitor	Ventricular arrhythmias, symptomatic atrial fibtillation, and symptomatic atriall flutter	10/30/1992	P, O
15	Gadoteridol (Prohance)	Bracco	Gadolinium-based MRI contrast agent	Imaging of central nervous system	11/16/1992	S
16	Atovaquone (Mepron)	GlaxoSmithKline	Cytochrome bc1 complex (Complex III) inhibitor	*Pneumocystis carinii* pneumonia	11/25/1992	P, O
17	Desogestrel; Ethinyl estradiol (Desogen)	Organon USA	Progesterone receptor activator; derivative of the natural estrogen estradiol	Contraception	12/10/1992	S
18	Zolpidem tartrate (Ambien)	Sanofi	γ-Aminobutyric-acid type A (GABA-A receptor) stimulator	Insomnia	12/16/1992	S
19	Rifabutin (Mycobutin)	Pfizer	Bacterial RNA polymerase inhibitor	Prophylaxis of disseminated MAC disease in patients with advanced HIV infection	12/23/1992	P, O
20	Sumatriptan succinate (Imitrex)	GlaxoSmithKline	5-$HT_{1B/D}$ receptor agonist	Reduction of vascular inflammation associated with migraines	12/28/1992	P
21	Indium In-111 chloride (Indiclor)	GE Healthcare	Radioactive diagnostic agent	Radiolabeling Satumomab pendetide (OncoScint), Capromab pendetide (ProstaScint) and Ibritumomab tiuxetan (Zevalin)	12/29/1992	S
22	Paroxetine HCl (Paxil)	Apotex Technologies	Serotonin reuptake inhibitor	Major depressive disorder, obsessive-compulsive disorder, social anxiety disorder, panic disorder, posttraumatic stress disorder, generalized anxiety disorder, and premenstrual dysphoric disorder	12/29/1992	S
23	Paclitaxel (Taxol)	HQ Spclt Pharma	Microtubule-stabilizing agent	Advanced ovarian cancer	12/29/1992	P, O
24	Flosequinan (Manoplax)	Boots	Potential Phosphodiesterase 3 (PDE3) inhibitor	Heart failure	12/30/1992	N/A
25	Nedocromil sodium (Tilade)	King Pharms	Mast cell stabilizer	Itching associated with allergic conjunctivitis	12/30/1992	S
26	Terbinafine HCl (Lamisil)	Novartis	Inhibits squalene epoxidase-dependent ergosterol synthesis	Ringworm and fungal nail infections	12/30/1992	S
	**1992**	**Biologics license applications = 2**				
1	Aldesleukin (Proleukin)	Chiron	Recombinant human interleukin-2	Metastatic renal cell carcinoma	05/05/1992	O
2	Indium In-111 satumomab pendetide (OncoScint)	Cytogen	TAG-72-specific radiolabeled mAb	Imaging of colorectal or ovarian cancer	12/29/1992	O

Supplementary data: Table 1 – FDA-approved drugs (Continued)

	Drug (brand name)	Sponsor	Properties	Indication	Approval date	Review
**1993**	**New molecular entities = 24**					
1	Gadodiamide (Omniscan)	GE Healthcare	Nonionic, low-osmolar gadolinium-based contrast agent	Contrast-enhanced magnetic resonance imaging	01/08/1993	S
2	Cladribine (Leustatin)	Janssen Pharms	Adenosine deaminase inhibitor	Hairy cell leukemia and multiple sclerosis	02/26/1993	S
3	Enoxaparin sodium (Lovenox)	Sanofi	Antithrombin III activator	Deep vein thrombosis (DVT) and pulmonary embolism (PE)	03/29/1993	P
4	Loratadine (Claritin)	Bayer	Histamine H1 receptor antagonist	Allergic rhinitis and urticaria	04/12/1993	S
5	Strontium chloride Sr-89 (Metastron)	Q Biomed	A radiopharmaceutical that concentrates in the bone of invaded tumor	Bone metastasis of tumor induced pain	06/18/1993	P
6	Levomethadyl acetate HCl (Orlaam)	Roxane	μ-opioid receptor agonist	Opioid addiction	07/09/1993	P, O
7	Felbamate (Felbatol)	Mylan Speciality	Dicarbamate derivative, inhibition of voltage-sensitive sodium and calcium channels, modulation of NMDA receptors, and potentiation of GABA transmission	Epilepsy	07/29/1993	P, O
8	Cisapride monohydrate (Propulsid)	Janssen Pharms	5-HT$_4$ receptor agonist	Nocturnal heartburn due to gastroesophageal reflux disease	07/29/1993	S
9	Perflubron (Imagent)	Alliance Pharm	Bromine-based particulate contrast agent	Magnetic resonance imaging, computed tomography, and sonography	08/13/1993	P
10	Torsemide (Demadex)	Mylan Speciality	Pyridine-sulfonylurea type loop diuretic	Edema associated with congestive heart failure, renal disease, or hepatic disease	08/23/1993	S
11	Tacrine HCl (Cognex)	Shionogi	Parasympathomimetic, reversible cholinesterase inhibitor	Mild to moderate dementia of the Alzheimer's type	09/09/1993	P
12	Rimantadine HCl (Flumadine)	Sun Pharm	M2 ion channel inhibitor	Influenza virus A infection	09/17/1993	P
13	Lodoxamide tromethamine (Alomide)	Novartis	Mast cell stabilizer	Vernal keratoconjunctivitis, vernal conjunctivitis, and vernal keratitis	09/23/1993	S, O
14	Piperacillin sodium; Tazobactam sodium (Zosyn)	Wyeth Pharms	Broad-spectrum ampicillin derived ureidopenicillin antibiotic; β-lactamase inhibitor	Bacterial infections	10/22/1993	S
15	Levocabastine HCl (Livostin)	Novartis	Histamine H1-receptor antagonist	Allergic conjunctivitis	11/10/1993	P
16	Trimetrexate glucuronate (Neutrexin)	Medimmune Oncology	Folate antagonist, dihydrofolate reductase inhibitor	*Pneumocystis carinii* pneumonia	12/17/1993	P, O
17	Venlafaxine HCl (Effexor)	Wyeth Pharms	Arylalkanolamine serotonin-norepinephrine reuptake inhibitor	Major depressive disorder	12/28/1993	S
18	Granisetron HCl (Kytril)	Roche	5-HT$_3$ receptor agonist	Chemotherapy-induced nausea	12/29/1993	S
19	Risperidone (Risperdal)	Janssen Pharms	5-HT$_2$ receptor and dopamine D2 receptor antagonist	Schizophrenia, bipolar mania, irritability associated with autistic disorder	12/29/1993	P
20	Calcipotriene (Dovonex)	Leo Pharma	Synthetic derivative of calcitriol, a form of vitamin D	Psoriasis	12/29/1993	S
21	Perindopril erbumine (Aceon)	Symplmed Pharms	Angiotensin-converting enzyme (ACE) inhibitor	Hypertension	12/30/1993	S
22	Gabapentin (Neurontin)	Upjohn	γ-Aminobutyric-acid (GABA) analog	Seizures	12/30/1993	P
23	Fenofibrate (Lipidil)	AbbVie	Peroxisome proliferator activated receptor α (PPARα) activator	Hyperlipidemia	12/31/1993	S
24	Fluvastatin sodium (Lescol)	Novartis	HMG CoA reductase inhibitor	Hypercholesterolemia and mixed dyslipidemia	12/31/1993	S
**1993**	**Biologics license applications = 4**					
1	Antihemophilic factor Recombinant (Kogenate FS)	Bayer	Recombinant coagulation factor VIII	Hemophilia A, von Willebrand disease, and factor XIII deficiency	02/25/1993	O
2	Interferon beta-1b (Betaseron)	Bayer	Recombinant human interferon β-1b	Relapsing multiple sclerosis	07/23/1993	O
3	Aprotinin (Trasylol)	Bayer	Bovine pancreatic trypsin inhibitor	Prophylactic use to reduce perioperative blood loss and the need for blood transfusion	12/29/1993	P, O
4	Dornase alfa (Pulmozyme)	Genentech	Recombinant human DNase I	Cystic fibrosis	12/30/1993	O

Supplementary data: Table 1 – FDA-approved drugs (Continued)

	Drug (brand name)	Sponsor	Properties	Indication	Approval date	Review
	**1994** **New molecular entities = 20**					
1	Salmeterol xinafoate (Serevent)	GlaxoSmithKline	β2-adrenergic agonist	Asthma, prevention of exercise-induced bronchospasm, maintenance treatment of chronic obstructive pulmonary disease	02/04/1994	P
2	Budesonide (Rhinocort)	Astrazeneca	Glucocorticoid	Asthma, the treatment of various skin disorders, allergic rhinitis and ulcerative colitis	02/14/1994	S
3	Rocuronium bromide (Zemuron)	Organon USA	Neuromuscular blocker or muscle relaxant	Adjunctively in anesthesia for endotracheal intubation or to facilitate mechanical ventilation	03/17/1994	S
4	Acrivastine; Pseudoephedrine HCl (Semprex-D)	Auxilium Pharms	Histamine H1 receptor antagonist; α/β-adrenergic receptors agonist	Seasonal allergic rhinitis	03/25/1994	S
5	Iobenguane sulfate I-131	Pharmalucence	Radiopharmaceutical	Location of phaeochromocytomas and neuroblastomas	03/25/1994	P
6	Tacrolimus (Prograf)	Astellas	Macrolide immunosuppressant produced by *Streptomyces tsukubaensis*	Prophylaxis of organ rejection in patients receiving allogeneic liver transplants, kidney transplants, heart transplants	04/08/1994	P, O
7	Indium In-111 pentetreotide kit (Octreoscan)	Curium	Radioactive diagnostic agent	Location of metastatic neuroblastomas	06/02/1994	P
8	Stavudine (Zerit)	Bristol-Myers Squibb	HIV reverse transcriptase inhibitor	HIV-1 infection	06/24/1994	P
9	Famciclovir (Famvir)	Novartis	Viral DNA polymerase inhibitor	Recurrent episodes of orolabial or genital herpes in HIV-infected adults	06/29/1994	S
10	Cysteamine bitartrate (Cystagon)	Mylan	Cystine-depleting agent	Corneal cystine crystal accumulation in patients with cystinosis	08/15/1994	P, O
11	Fludeoxyglucose F-18	Downstate Clincl	Positron emitting radiopharmaceutical	Positron emission tomography (PET) imaging	08/19/1994	P
12	Technetium Tc-99m bicisate kit (Neurolite)	Lantheus Medcl	Radioactive diagnostic agent	Localization of neuroendocrine tumors bearing somatostatin receptors	11/23/1994	S
13	Fluvoxamine maleate (Luvox)	Solvay	Serotonin reuptake inhibitor	Obsessive-compulsive disorder	12/05/1994	S
14	Dorzolamide HCl (Trusopt)	Merck & Co	Carbonic anhydrase II (CA-II) inhibitor	Elevated intraocular pressure in patients with ocular hypertension or open-angle glaucoma	12/09/1994	P
15	Nefazodone HCl (Serzone)	Bristol-Myers Squibb	5-HT_2 receptor agonist	Depression	12/22/1994	S
16	Dalteparin sodium (Fragmin)	Pfizer	Increasing the activity of antithrombin III (AT-III)	Thromboprophylaxis in general/abdominal surgery	12/22/1994	S
17	Vinorelbine tartrate (Navelbine)	Pierre Fabre	Microtubule-destabilizing agent	Non-small-cell lung cancer (NSCLC)	12/23/1994	P
18	Lamotrigine (Lamictal)	GlaxoSmithKline	Presynaptic neuronal membranes stabilizer and glutamate release inhibitor	Epilepsy and bipolar disorder	12/27/1994	P
19	Spirapril HCl (Renormax)	Schering	Angiotensin-converting enzyme (ACE) inhibitor	Hypertension	12/29/1994	S
20	Rimexolone (Vexol)	Eyevance	Glucocorticoid receptor agonist	Eye inflammation caused by infections, injury, surgery, or other conditions	12/30/1994	P
	**1994** **Biologics license applications = 3**					
1	Pegaspargase (Oncaspar)	Sigma Tau	*Escherichia coli*-derived L-asparaginase	Pediatric acute lymphoblastic leukaemia	02/01/1994	O
2	Imiglucerase (Cerezyme)	Genzyme	Recombinant human β-glucocerebrosidase enzyme	Type 1 Gaucher’s disease	05/23/1994	O
3	Abciximab (Reopro)	Centocor	anti-glycoprotein IIb/IIIa receptor mAb	Prevention of cardiac ischemic complications in patients undergoing percutaneous coronary intervention	12/22/1994	N/A

Supplementary data: Table 1 – FDA-approved drugs (Continued)

	Drug (brand name)	Sponsor	Properties	Indication	Approval date	Review
	**1995** **New molecular entities = 29**					
1	Nisoldipine (Sular)	Covis	Calcium channel antagonist	Hypertension	02/02/1995	S
2	Tramadol HCl (Ultram)	Janssen Pharms	μ-opioid receptor agonist	Moderate to severe pain	03/03/1995	S
3	Metformin HCl (Glucophage)	Emd Serono	Decreasing glucose production by the liver	Type 2 diabetes	03/03/1995	P
4	Losartan potassium (Cozaar)	Organon	Angiotensin II receptor type 1 (AT1) antagonist	Hypertension	04/14/1995	S
5	Nalmefene HCl (Revex)	Eurohlth Intl Sarl	μ- and δ-opioid antagonist	Reversal of opioid drug effects	04/17/1995	S
6	Moexipril HCl (Univasc)	UCB Inc	Angiotensin-converting enzyme (ACE) inhibitor	Hypertension	04/19/1995	S
7	Mycophenolate mofetil (Cellcept)	Roche Palo	Inosine-5'-monophosphate dehydrogenase inhibitor	Prophylaxis of organ rejection in recipients of allogeneic kidney, heart, or liver transplants	05/03/1995	P
8	Iopromide (Ultravist 150)	Bayer	Non-ionic X-ray contrast agent	Angiography and computed tomography imaging	05/10/1995	S
9	Lansoprazole (Prevacid)	Takeda	Proton pump inhibitor	Acid-reflux disorders (GERD), peptic ulcer disease, *H. pylori* eradication, and prevention of gastrointestinal bleeds with NSAID use	05/10/1995	S
10	Dexrazoxane HCl (Zinecard)	Pfizer	DNA topoisomerase II inhibitor and iron ions chelator	Doxorubicin-caused cardiomyopathy in patients with breast cancer	05/26/1995	P, O
11	Sevoflurane (Ultane)	AbbVie	Reduction in junctional conductance by decreasing gap junction channel opening times and increasing gap junction channel closing times	Induction and maintenance of general anesthesia	06/07/1995	S
12	Dirithromycin (Dynabac)	Lilly Res Labs	Inhibits bacterial protein synthesis by binding to the 50S ribosomal subunit of the bacterial 70S ribosome	Bacterial infections in respiratory system and skin	06/19/1995	S
13	Acarbose (Precose)	Bayer	Pancreatic α-amylase and membrane-bound intestinal α-glucoside hydrolase enzymes inhibitor	Type 2 diabetes	09/06/1995	S
14	Azelaic acid (Azelex)	Almirall	May inhibit the cellular protein synthesis in bacteria	Mild to moderate inflammatory acne vulgaris	09/13/1995	S
15	Carvedilol (Coreg)	Woodward	α1- and β-adrenergic receptor inhibitor	Mild to severe heart failure	09/14/1995	S
16	Epoprostenol sodium (Flolan)	GlaxoSmithKline	Prostaglandin	Pulmonary arterial hypertension	09/20/1995	P, O
17	Alendronate sodium (Fosamax)	Merck & Co	Osteoclast-mediated bone-resorption inhibitor	Postmenopausal osteoporosis	09/29/1995	P
18	Bicalutamide (Casodex)	Ani Pharms	Androgen receptor antagonist	Advanced prostate cancer	10/04/1995	S
19	Lamivudine (Epivir)	Viiv Hlthcare	HIV reverse transcriptase inhibitor	HIV-1 infection	11/17/1995	P
20	Glimepiride (Amaryl)	Sanofi	Insulin secretagogue	Type 2 diabetes	11/30/1995	S
21	Saquinavir mesylate (Invirase)	Roche	HIV protease inhibitor	HIV-1 infection	12/06/1995	P
22	Cetirizine HCl (Zyrtec allergy)	J&J	Histamine H1 receptor antagonist	Allergy symptoms	12/08/1995	S
23	Amifostine (Ethyol)	Clinigen	Binds to and detoxifies cytotoxic platinum-containing metabolites of cisplatin	Cisplatin caused cumulative renal toxicity in patients with ovarian cancer	12/08/1995	P, O
24	Riluzole (Rilutek)	Covis	Glutamate antagonist	Amyotrophic lateral sclerosis	12/12/1995	P, O
25	Ceftibuten dihydrate (Cedax)	Sl Pharms	Cephalosporin antibiotic, inhibits bacterial cell wall synthesis by binding to specific PBPs	Chronic bronchitis, acute bacterial otitis media, pharyngitis, and tonsillitis	12/20/1995	S
26	Ioxilan (Oxilan-300)	Guerbet	Non-ionic X-ray contrast agent	Angiography, urography, and computed tomography imaging	12/21/1995	S
27	Porfimer sodium (Photofrin)	Pinnacle Biolgs	A photosensitizer used for photodynamic therapy	Advanced esophageal cancer	12/27/1995	P, O
28	Anastrozole (Arimidex)	Ani Pharms	Aromatase inhibitor	Advanced breast cancer in post-menopausal women	12/27/1995	S
29	Ibutilide fumarate (Corvert)	Pfizer	Delays repolarization by activation of a slow, inward sodium current	The rapid conversion of atrial fibrillation or atrial flutter of recent onset to sinus rhythm	12/28/1995	S

Supplementary data: Table 1 – FDA-approved drugs (Continued)

	Drug (brand name)	Sponsor	Properties	Indication	Approval date	Review
	**1996** **New molecular entities = 51**					
1	Cefepime HCl (Maxipime)	Hospira	Bacterial cell wall synthesis inhibitor	Pneumonia caused by *Streptococcus pneumoniae*	01/18/1996	S
2	Technetium Tc-99m tetrofosmin kit (Myoview)	Janssen Pharms	Radioactive diagnostic agent	Myocardial perfusion imaging for heart disease diagnosis	03/03/1995	S
3	Ritonavir (Norvir)	AbbVie	HIV protease inhibitor	HIV-1 infection	03/01/1996	P
4	Indinavir sulfate (Crixivan)	Merck & Co	HIV protease inhibitor	HIV-1 infection	03/13/1996	P
5	Iodixanol (Visipaque 270)	GE Healthcare	Non-ionic X-ray contrast agent	Angiocardiography	03/22/1996	S
6	Trandolapril (Mavik)	AbbVie	Angiotensin-converting enzyme (ACE) inhibitor	Hypertension and left ventricular dysfunction	04/26/1996	S
7	Sodium phenylbutyrate (Buphenyl)	Horizon Therap	Pro-drug of phenylacetate, which conjugates with glutamine to form phenylacetylglutamine	Urea cycle disorders	04/30/1996	P, O
8	Docetaxel (Taxotere)	Sanofi	Microtubule-stabilizing agent	Locally advanced or metastatic breast cancer	05/14/1996	P
9	Gemcitabine HCl (Gemzar)	Eli Lilly	DNA synthesis inhibitor	Locally advanced or metastatic pancreatic cancer	05/15/1996	P
10	Topotecan HCl (Hycamtin)	Novartis	DNA topoisomerases inhibitor	Relapsed small cell lung cancer	05/28/1996	P
11	Adapalene (Differin)	Galderma Labs	Retinoid	Acne	05/31/1996	S
12	Latanoprost (Xalatan)	Upjohn	Prostaglandin F2α receptor agonist	Open-angle glaucoma or ocular hypertension	06/05/1996	P
13	Albendazole (Albenza)	Impax Labs	Microtubule-destabilizing agent, binds to the colchicine-sensitive site of β-tubulin	Neurocysticercosis (*Taenia solium tapeworm*), Hydatid (*Echinococcus tapeworm*)	06/11/1996	P, O
14	Mirtazapine (Remeron)	Organon USA	5-$HT_{2/3}$ receptors antagonist; α2 adrenergic auto- and hetero-receptors antagonist	Major depression	06/14/1996	S
15	Irinotecan HCl (Camptosar)	Pfizer	Topoisomerase I inhibitor	Metastatic colorectal cancer	06/14/1996	P
16	Nevirapine (Viramune)	Boehringer Ingelheim	HIV reverse transcriptase inhibitor	HIV-1 infection	06/21/1996	P
17	Meropenem (Merrem)	Pfizer	Inhibits cell-wall synthesis by binding to PBPs	Bacterial meningitis caused by *Haemophilus influenzae*, *Neisseria meningitidis* and penicillin-susceptible isolates of *Streptococcus pneumoniae*	06/21/1996	S
18	Cidofovir (Vistide)	Gilead Sciences	Viral DNA polymerase inhibitor	Cytomegalovirus retinitis in patients with AIDS	06/26/1996	S
19	Remifentanil HCl (Ultiva)	Mylan Institutional	μ-opioid agonist	Adjunct to an anesthetic	07/12/1996	S
20	Fexofenadine HCl (Allegra)	Chattem Sanofi	Histamine H1 receptor antagonist	Seasonal allergic rhinitis	07/25/1996	S
21	Fosphenytoin sodium (Cerebyx)	Parke Davis	Voltage-dependent neuronal sodium channels inhibitor	Epilepsy	08/05/1996	S
22	Bentoquatam (Ivy block)	Stand Homeopath	Physical shield against exposure to the irritating substance	Protects skin from the plant oils	08/26/1996	P
23	Ferumoxides (Feridex I.V.)	Amag Pharms	Superparamagnetic iron oxide, MRI contrast agent	Enhances the T2 weighted imaging for liver lesions diagnosis	08/30/1996	S
24	Midodrine HCl (Proamatine)	Takeda	α1-adrenergic receptor agonist	Orthostatic hypotension	09/06/1996	P, O
25	Brimonidine tartrate (Alphagan)	Allergan	α2-adrenergic receptor agonist	Open-angle glaucoma or ocular hypertension	09/06/1996	S
26	Urea C-13 (Breathtek UBT for H-pylori)	Otsuka America	^{13}C labeled urea	Initial diagnosis or post-treatment monitoring of Helicobacter pylori infection	09/17/1996	S
27	Nilutamide (Nilandron)	Concordia	Androgen receptor antagonist	Metastatic prostate cancer	09/19/1996	S
28	Ropivacaine HCl (Naropin)	Fresenius Kabi	Blocks the sodium-channel and decreases chances of depolarization and consequent action potentials	Local or regional anesthesia for surgery and acute pain management	09/24/1996	S
29	Penciclovir (Denavir)	Mylan	HSV DNA polymerase inhibitor	Recurrent herpes labialis	09/24/1996	S
30	Pentosan polysulfate sodium (Elmiron)	Janssen Pharms	FGF-1/2/4 antagonist	Bladder pain or discomfort caused by interstitial cystitis	09/26/1996	S, O
31	Zafirlukast (Accolate)	Strides Pharma	Leukotriene D4 and E4 receptor antagonist	Prophylaxis and chronic treatment of asthma	09/26/1996	S
32	Olanzapine (Zyprexa)	Lilly	Dopamine D2 receptor antagonist	Schizophrenia and bipolar disorder	09/30/1996	S
33	Butenafin HCl (Mentax)	Mylan	Squalene epoxidation inhibitor	Pityriasis versicolor due to *M. furfur*; Interdigital tinea pedis, tinea corporis and tinea cruris due to *E. floccosum*	10/18/1996	S
34	Betaine (Cystadane)	Recordati Rare	Methyl group donor	Hypercystinemia	10/25/1996	P, O
35	Azelastine HCl (Astelin)	Mylan Spclt Viatris	Histamine H1-receptor antagonis	Seasonal allergic rhinitis	11/01/1996	S
36	Ivermectin (Stromectol)	Merck & Co	Glutamate-gated chloride ion channels activator	Head lice, onchocerciasis, and other worms	11/22/1996	P
37	Donepezil HCl (Aricept)	Eisai	Acetylcholinesterase inhibitor	Alzheimer's disease	11/25/1996	P
38	Tizanidine HCl (Zanaflex)	Covis	α2-adrenergic receptor agonist	Relieves the spasms and increased muscle tone caused by multiple sclerosis	11/27/1996	S, O
39	Ferumoxsil (Gastromark)	Amag Pharms	MRI contrast agent	Enhanced MRI of bowel delineation	12/06/1996	S
40	Zileuton (Zyflo)	Chiesi	5-lipoxygenase inhibitor	Prophylaxis and chronic treatment of asthma	12/09/1996	S

Supplementary data: Table 1 – FDA-approved drugs (Continued)

	Drug (brand name)	Sponsor	Properties	Indication	Approval date	Review
	**1996** **New molecular entities = 51**					
41	Amlexanox (Aphthasol)	Uluru	Inhibits the slow-reacting substance of anaphylaxis release, IL-3 antagonist	Allergic asthma and rhinitis	12/17/1996	P
42	Atorvastatin calcium (Lipitor)	Upjohn	HMG CoA reductase inhibitor	Hyperlipidemia	12/17/1996	P
43	Miglitol (Glyset)	Pfizer	Membrane-bound intestinal α-glucoside hydrolase enzymes inhibitor	Type 2 diabetes	12/18/1996	S
44	Olopatadine HCl (Pataday twice daily relief)	Alcon Labs	Histamine H1 antagonist	Itching associated with allergic conjunctivitis	12/18/1996	S
45	Sparfloxacin (Zagam)	Mylan	Bacterial DNA gyrase inhibitor	Bacterial infections of the respiratory tract	12/19/1996	S
46	Fosfomycin tromethamine (Monurol)	Zambon SPA	Phosphoenolpyruvate synthetase inhibitor	Urinary tract infections (acute cystitis) caused by Escherichia coli and Enterococcus faecalis	12/19/1996	S
47	Glatiramer acetate (Copaxone)	Teva	Binds to MHC and inhibits the T cell response to several myelin antigens	Relapsing forms of multiple sclerosis	12/20/1996	S, O
48	Cabergoline (Dostinex)	Pfizer	Dopamine D2 receptor agonist	Hyperprolactinemia	12/23/1996	S
49	Valsartan (Diovan)	Novartis	Angiotensin II receptor blocker	Hypertension	12/23/1996	S
50	Danaparoid sodium (Orgaran)	Aspen Global	Factor IIa (Thrombin) and factor Xa inhibitor	Prophylaxis of post-operative deep vein thrombosis	12/24/1996	S
51	Topiramate (Topamax)	Janssen Pharms	Voltage-dependent neuronal sodium channels inhibitor, GABA-A receptor stimulator, and APMA glutamate receptors blocker	Epilepsy	12/24/1996	S
	**1996** **Biologics license applications = 9**					
1	Respiratory syncytial virus immune globulin [human] (RespiGAM)	MedImmune	Respiratory syncytial virus (RSV) immune globulin	Prevention of respiratory syncytial virus (RSV) in high risk infants	01/18/1996	N/A
2	Interferon beta-1a (Avonex)	Biogen	Recombinant human interferon β-1a	Relapsing forms of multiple sclerosis	05/17/1996	O
3	Corticorelin ovine triflutate (Acthrel)	Ferring	Synthetic human corticotropin-releasing hormone (hCRH)	Pituitary and ectopic production of ACTH in patients with ACTH-dependent Cushing's syndrome	05/23/1996	O
4	Insulin lispro recombinant (Humalog)	Eli Lilly	Recombinant human insulin	Type 1 & 2 diabetes	06/14/1996	N/A
5	Arcitumomab (CEA-Scan)	Immunomedics	^{99m}Tc labelled anti-CEA mouse IgG1 (Fab)	Recurrent colorectal cancer imaging	06/28/1996	O
6	Imciromab pentetate (Myoscint)	Centocor	^{111}In labelled anti-myosin mouse IgG2a (Fab)	Cardiac imaging	07/03/1996	N/A
7	Nofetumomab merpentan (Verluma)	Boehringer Ingelheim	^{99m}Tc labelled anti-EpCAM (and/or CD20/MS4A1) mouse IgG2b (Fab)	Small cell lung cancer imaging	8/20/1996	N/A
8	Capromab pendetide (Prostascint)	Cytogen	^{111}In labelled anti-PSMA mouse mAb	Diagnosis of metastatic prostate cancer	10/28/1996	N/A
9	Reteplase (Retavase)	EKR Therap	Recombinant plasminogen activator which catalyzes the cleavage of endogenous plasminogen to generate plasmin	Myocardial infarction, ischemic stroke, and pulmonary emboli	10/30/1996	N/A

Supplementary data: Table 1 – FDA-approved drugs (Continued)

	Drug (brand name)	Sponsor	Properties	Indication	Approval date	Review
	**1997 New molecular entities = 36**					
1	Troglitazone (Rezulin)	Pfizer	PPARγ ligand	Type 2 diabetes	01/29/1997	P
2	Imiquimod (Aldara)	Bausch	Toll-like receptor 7 agonist	External genital and perianal warts	02/27/1997	S
3	Tiludronate disodium (Skelid)	Sanofi	Protein-tyrosine-phosphatase inhibitor and osteoclastic proton pump inhibitor	Paget's disease of the bone	03/07/1997	S
4	Anagrelide HCl (Agrylin)	Takeda	Phosphodiesterase 3 (PDE3) and phospholipase A2 (PLA2) inhibitor	Thrombocythemia, secondary to myeloproliferative neoplasms	12/06/1996	S
5	Nelfinavir mesylate (Viracept)	Agouron Pharms	HIV protease inhibitor	HIV-1 infection	03/14/1997	P
6	Samarium Sm-153 lexidronam pentasodium (Quadramet)	Lantheus Medical	^{153}Sm labelled Radiopharmaceutical, chelator moiety associates with hydroxyapatite crystals concentrated in areas of bone turnover	Bone metastasis of tumor induced pain	03/28/1997	S
7	Delavirdine mesylate (Rescriptor)	Viiv Hlthcare	HIV reverse transcriptase inhibitor	HIV-1 infection	04/04/1997	P
8	Tamsulosin HCl (Flomax)	Sanofi	α-adrenoceptor blocker	Benign prostatic hyperplasia	04/15/1997	S
9	Urea, C-14 (Pytest)	Avent	^{14}C labelled urea	Breath test for *Helicobacter pylori*	05/09/1997	P
10	Ardeparin sodium (Normiflo)	Pharmacia & Upjohn	Low-molecular weight heparin	Prevention of deep venous thrombosis	05/23/1997	S
11	Toremifene citrate (Fareston)	Kyowa kirin	Estrogen receptor (ER) inhibitor	ER-positive metastatic breast cancer	05/29/1997	S, O
12	Tazarotene (Tazorac)	Allergan	Prodrug of retinoid	Plaque psoriasis	06/13/1997	S
13	Mibefradil 2HCl (Posicor)	Roche	Calcium channel blocker	Hypertension and chronic angina pectoris	06/20/1997	S
14	Cerivastatin sodium (Baycol)	Bayer	HMG CoA reductase inhibitor	Hyperlipidemia	06/26/1997	S
15	Pramipexole 2HCl (Mirapex)	Boehringer Ingelheim	Non-ergot dopamine agonist	Idiopathic Parkinson's disease	07/01/1997	S
16	Bromfenac sodium (Duract)	Wyeth Pharms	Cycooxygenase-2 (COX-2) inhibitor	Acute pain following surgery	07/15/1997	S
17	Letrozole (Femara)	Novartis	Aromatase inhibitor	Advanced breast cancer	07/25/1997	S
18	Dolasetron mesylate (Anzemet)	Validus Pharms	5-HT_3 receptor antagonist	Chemotherapy-induced nausea and vomiting	09/11/1997	S
19	Arbutamine HCl (Genesa)	Gensia automedics	α1-adrenergic receptor agonist	Adjunctive diagnosis of coronary artery disease	09/12/1997	S
20	Ropinirole HCl (Requip)	GlaxoSmithKline	Non-ergot dopamine agonist	Parkinson's disease	09/19/1997	S
21	Fenoldopam mesylate (Corlopam)	Hospira	Dopamine D1 receptor agonist	Hypertension	09/23/1997	S
22	Quetiapine fumarate (Seroquel)	AstraZeneca	Dopamine D2 and 5-HT_{2A} receptors antagonist	Bipolar disorder, schizophrenia, and major depressive disorder	09/26/1997	S
23	Tiagabine HCl (Gabitril)	Cephalon	γ-Aminobutyric-acid (GABA) reuptake inhibitor	Epilepsy	09/30/1997	S
24	Irbesartan (Avapro)	Sanofi	Angiotensin II receptor blocker	Hypertension	09/30/1997	S
25	Grepafloxacin HCl (Raxar)	Otsuka	Bacterial topoisomerase II (DNA gyrase) and topoisomerase IV inhibitor	Mild to moderate infections caused by *Haemophilus influenzae*, *Streptococcus pneumoniae*, or *Moraxella catarrhalis*	11/06/1997	S
26	Clopidogrel bisulfate (Plavix)	Sanofi	Adenosine diphosphate inhibitor	Prevents blood clots in peripheral vascular disease, coronary artery disease, and cerebrovascular disease	11/17/1997	S
27	Sibutramine HCl (Meridia)	Abbott	Norepinephrine, serotonin and dopamine reuptake inhibitor	Obesity	11/22/1997	S
28	Zolmitriptan (Zomig)	IPR	5-$HT_{1B/D/F}$ receptor agonist	Migraine	11/25/1997	S
29	Mangafodipir trisodium (Teslascan)	IC Targets	MRI contrast agent	Imaging of liver lesions with suspected metastatic disease or hepatocellular carcinomas	11/26/1997	S
30	Fomepizole (Antizol)	PAR Pharm	Alcohol dehydrogenase inhibitor	Antidote for ethylene glycol or methanol poisoning	12/04/1997	S, O
31	Cefdinir (Omnicef)	AbbVie	Cell wall synthesis inhibitor	Bacterial infections，including pneumonia, acute exacerbations of chronic bronchitis, acute maxillary sinusitis, pharyngitis/tonsillitis and skin infections	12/04/1997	S
32	Raloxifene HCl (Evista)	Eli Lilly	Estrogen receptor modulator	Osteoporosis in postmenopausal women	12/09/1997	P
33	Trovafloxacin mesylate (Trovan)	Pfizer	Bacterial topoisomerase II (DNA gyrase) and topoisomerase IV inhibitor	Gonorrhea and chlamydia	12/18/1997	S
34	Eprosartan mesylate (Teveten)	AbbVie	Angiotensin II receptor antagonist	Hypertension	12/22/1997	S
35	Repaglinide (Prandin)	Gemini Labs	Insulin secretagogue	Type 2 diabetes	12/22/1997	P
36	Emedastine difumarate (Emadine)	Novartis	Histamine H1-receptor antagonist	Allergic conjunctivitis	12/29/1997	S
	**1997 Biologics license applications = 6**					
1	Autologous cultured chondrocytes (Carticel)	Genzyme Tissue Repair	Autologous cultured chondrocytes (Cell therapy)	Knee cartilage damage	08/22/1997	N/A
2	Interferon alfacon-1 (Infergen)	Amgen	Recombinant human interferon	Hepatitis C virus infection	10/06/1997	N/A
3	Oprelvckin (Neumega)	Genetics institute	Recombinant human interleukin-11 (IL-11)	Chemotherapy-induced thrombocytopenia	11/25/1997	N/A

Supplementary data: Table 1 – FDA-approved drugs (Continued)

	Drug (brand name)	Sponsor	Properties	Indication	Approval date	Review
	**1997** **Biologics license applications = 6**					
4	Rituximab (Rituxan)	Genentech	CD20-directed mAb	B-cell non-Hodgkin's lymphoma	11/26/1997	O
5	Daclizumab (Zenapax)	Roche	CD25 (IL-2 receptor)-directed mAb	Prophylaxis of acute organ rejection in patients receiving renal transplants	12/10/1997	N/A
6	Becaplermin (Regranex)	Smith & Nephew	Recombinant human platelet-derived growth factor (PDGF)	Ulcers caused by diabetic neuropathy	12/16/1997	N/A

Supplementary data: Table 1 – FDA-approved drugs (Continued)

	Drug (brand name)	Sponsor	Properties	Indication	Approval date	Review
	**1998 New molecular entities = 26**					
1	Tolcapone (Tasmar)	Bausch	Catechol-O-methyltransferase inhibitor	Parkinson's disease	01/29/1998	S
2	Naratriptan HCl (Amerge)	GlaxoSmithKline	5-$HT_{1B/D}$ receptors agonist	Migraine headache	02/10/1998	S
3	Loteprednol etabonate (Lotemax)	Bausch & Lomb	Corticosteroid	Steroid responsive inflammatory condition of the eye	03/09/1998	S
4	Tolterodine tartrate (Detrol)	Upjohn	Muscarinic receptor antagonist	Overactive bladder with urinary incontinence, urgency, and frequency	03/25/1998	S
5	Risedronate sodium (Actonel)	APIL	Farnesyl pyrophosphate synthase inhibitor	Osteoporosis and Paget's disease of the bone	03/27/1998	S
6	Sildenafil citrate (Viagra)	Viatris	Phosphodiesterase 5 (PDE5) inhibitor	Erectile dysfunction	03/27/1998	P
7	Brinzolamide (Azopt)	Novartis	Carbonic anhydrase II inhibitor	Elevated intraocular pressure in patients with ocular hypertension or open-angle glaucoma	04/01/1998	S
8	Paricalcitol (Zemplar)	AbbVie	Synthetic vitamin D2 analog	Secondary hyperparathyroidism associated with chronic renal failure	04/17/1998	S
9	Capecitabine (Xeloda)	Roche	Prodrug of 5-FU	Metastatic breast cancer	04/30/1998	P
10	Tirofiban HCl (Aggrastat)	Medicure	Platelet glycoprotein IIb/IIIa receptor antagonist	Acute coronary syndrome	05/14/1998	P
11	Eptifibatide (Integrilin)	Schering	Platelet glycoprotein IIb/IIIa receptor antagonist	Acute coronary syndrome	05/18/1998	P
12	Candesartan cilexetil (Atacand)	Ani Pharms	Angiotensin II receptor type 1 antagonist	Hypertension	06/04/1998	S
13	Rifapentine (Priftin)	Sanofi	DNA-dependent RNA polymerase inhibitor	Active pulmonary tuberculosis caused by *Mycobacterium tuberculosis*	06/22/1998	P, O
14	Rizatriptan benzoate (Maxalt)	Merck & Co	5-$HT_{1B/D}$ receptor agonist	Migraine headache	06/29/1998	S
15	Thalidomide (Thalomid)	Celgene	Inhibition of TNF-α release from monocytes	Erythema nodosum leprosum	07/16/1998	P, O
16	Citalopram HBr (Celexa)	Allergan	Serotonin reuptake inhibitor	Major depression	07/17/1998	S
17	Fomivirsen sodium (Vitravene preservative free)	Novartis	Human cytomegalovirus replication inhibitor through an antisense mechanism	Cytomegalovirus retinitis in patients with AIDS	08/26/1998	P
18	Leflunomide (Arava)	Sanofi	Pyrimidine synthesis inhibitor	Rheumatoid arthritis	09/10/1998	P
19	Technetium Tc-99m apcitide (Acutect)	Cis Bio Intl	^{99m}Tc labelled radioactive diagnostic agent with platelet glycoprotein IIb/IIIa receptor binding	Imaging of acute venous thrombosis in the lower extremities in patients with signs and symptoms of acute blood clots	09/14/1998	P
20	Efavirenz (Sustiva)	Bristol-Myers Squibb	HIV reverse transcriptase inhibitor	HIV-1 infection	09/17/1998	P
21	Valrubicin (Valstar)	Endo Pharm	A semisynthetic analog of anthracycline doxorubicin	BCG-refractory CIS of urinary bladder cancer	09/25/1998	P, O
22	Sevelamer HCl (Renagel)	Genzyme	Phosphate binder	Hyperphosphatemia with chronic kidney disease	10/30/1998	S
23	Telmisartan (Micardis)	Boehringer Ingelheim	Angiotensin II receptor antagonist	Hypertension	11/10/1998	S
24	Abacavir sulfate (Ziagen)	Viiv Hlthcare	HIV reverse transcriptase inhibitor	HIV infection	12/17/1998	P
25	Modafinil (Provigil)	Cephalon	Indirect dopamine receptor agonist	Sleep apnea, narcolepsy, or shift work disorde	12/24/1998	S, O
26	Celecoxib (Celebrex)	Upjohn	Cyclooxygenase-2 (COX-2) inhibitor	Osteoarthritis and rheumatoid arthritis	12/31/1998	P
	**1998 Biologics license applications = 10**					
1	Lepirudin (Refludan)	Hoechst Marion Roussel	Protein-based direct thrombin inhibitor	Heparin-associated thrombocytopenia (type 2) and thromboembolic disease	03/06/1998	P, O
2	Sacrosidase (Sucraid)	QOL Medcl	Naturally-occurring gastrointestinal enzyme derived from baker's yeast (*Saccharomyces cerevisiae*)	Congenital sucrase-isomaltase deficiency	04/09/1998	O
3	Basiliximab (Simulect)	Novartis	CD25 (IL-2Rα)-directed mAb	Acute rejection of renal transplant	05/12/1998	P, O
4	Palivizumab (Synagis)	Medimmune	Anti-F protein (Respiratory syncytial virus) mAb	Respiratory syncytial virus infection	06/19/1998	P
5	Calfactant (Infasurf preservative free)	ONY	Modified natural bovine pulmonary surfactant	Respiratory distress syndrome in premature infants	07/01/1998	O
6	Infliximab (Remicade)	Centocor	Anti-TNF-α mAb	Rheumatoid arthritis, Crohn's disease, and ankylosing spondylitis	08/24/1998	O
7	Trastuzumab (Herceptin)	Genentech	HER2-directed mAb	HER2-positive breast cancer	09/25/1998	P
8	Etanercept (Enbrel)	Immunex	Dimeric fusion protein consisting of extracellular ligand-binding portion of TNFR inked to the Fc portion of human IgG1	Rheumatoid arthritis	11/02/1998	P
9	Thyrotropin alfa (Thyrogen)	Genzyme	Recombinant human thyroid stimulating hormone	Diagnostic testing for thyroid tissue remnants following radioiodine ablation, and alongside radioactive agents to destroy thyroid tissue	11/30/1998	O
10	Anti-thymocyte globulin [Rabbit] (Thymoglobulin)	Genzyme	Purified rabbit anti-thymocyte antibody	Acute rejection of renal transplant	12/30/1998	S

Supplementary data: Table 1 – FDA-approved drugs (Continued)

	Drug (brand name)	Sponsor	Properties	Indication	Approval date	Review
	**1999 New molecular entities = 32**					
1	Cilostazol (Pletal)	Otsuka	Phosphodiesterase 3 (PDE3) inhibitor	Intermittent claudication	01/15/1999	S
2	Alitretinoin (Panretin)	Concordia	9-cis-retinoic acid, a form of vitamin A	AIDS-related Kaposi's sarcoma	02/02/1999	P, O
3	Amprenavir (Agenerase)	GlaxoSmithKline	HIV-1 protease inhibitor	HIV infections	04/15/1999	N/A
4	Orlistat (Xenical)	Cheplapharm	Gastrointestinal lipase inhibitor	Obesity	04/23/1999	P
5	Rofecoxib (Vioxx)	Merck & Co	Cyclooxygenase-2 (COX-2) inhibitor	Osteoarthritis, rheumatoid arthritis, and acute pain	05/20/1999	P
6	Rosiglitazone maleate (Avandia)	Woodward	PPARs agonist	Type 2 diabetes	05/25/1999	P
7	Doxercalciferol (Hectorol)	Sanofi	Synthetic vitamin D2 analog	Secondary hyperparathyroidism in patients with chronic kidney disease	06/09/1999	S
8	Pioglitazone HCl (Actos)	Takeda	PPARγ agonist	Type 2 diabetes	07/15/1999	P
9	Zanamivir (Relenza)	GlaxoSmithKline	Neuraminidase inhibitor	Influenza A and B virus infections	07/26/1999	P
10	Ganirelix acetate (Antagon)	Organon	GnRH antagonist	Inhibition of premature luteinizing hormone (LH) surges in women undergoing controlled ovarian hyperstimulation	05/18/1998	P
11	Technetium Tc-99m depreotide (Neo tect kit)	Cis Bio Intl	^{99m}Tc labelled radioactive diagnostic agent	Imaging of malignant lung tumors in patients with highly suspect for malignancy	08/03/1999	P
12	Temozolomide (Temodar)	Merck & Co	DNA alkylating agent	Glioblastoma	08/11/1999	P, O
13	Zaleplon (Sonata)	Pfizer	Benzodiazepine receptor agonist	Insomnia	08/13/1999	S
14	Rapacuronium bromide (Raplon)	Organon	Muscarinic acetylcholine receptor M2 antagonist	Adjunctively in anesthesia for endotracheal intubation or to facilitate mechanical ventilation	08/18/1999	S
15	Rabeprazole sodium (Aciphex)	Woodward	Proton pump inhibitor	Gastroesophageal reflux disease	08/19/1999	S
16	Sirolimus (Rapamune)	Merck & Co	Mammalian target of rapamycin (mTOR) inhibitor	Prophylaxis of organ rejection	09/15/1999	P, O
17	Epirubicin HCl (Ellence)	Pfizer	DNA topoisomerase II inhibitor	Primary breast cancer with axillary node tumor involvement	09/15/1999	P, O
18	Quinupristin; Dalfopristin (Synercid)	King Pharms	Inhibitor of early and late phase of protein synthesis in the bacterial ribosome	Vancomycin-resistant *Enterococcus faecium* (VREF) bacteremia	09/21/1999	P
19	Pemirolast potassium (Alamast)	Santen	Mast cell stabilizer, Histamine H1-receptor antagonist	Itching of the eye due to allergic conjunctivitis	09/24/1999	P
20	Dofetilide (Tikosyn)	Pfizer	Blockade of the cardiac ion channel carrying the rapid component of the delayed rectifier potassium current	Maintenance of normal sinus rhythm	10/01/1999	S
21	Entacapone (Comtan)	Orion Pharma	Catechol-O-methyl transferase inhibitor	Parkinson's disease	10/19/1999	S
22	Exemestane (Aromasin)	Pfizer	Aromatase inhibitor	ER-positive early breast cancer	10/21/1999	S, O
23	Oseltamivir Phosphate (Tamiflu)	Roche	Influenza virus neuraminidase inhibitor	Influenza A and B infections	10/27/1999	P
24	Levetiracetam (Keppra)	UCB Inc	Synaptic vesicle protein 2A (SV2A) stimulator	Epilepsy	11/30/1999	S
25	Aminolevulinic acid HCl (Levulan)	DUSA	Porphyrin precursor	Actinic keratoses	12/03/1999	S
26	Gadoversetamide (Optimark)	Liebel-Flarsheim	Gadolinium-based MRI contrast agent	MRI tests of the brain, spine, and liver	12/08/1999	S
27	Mequinol; Tretinoin (Solage)	Almirall	Substrate of tyrosinase and acts as a competitive inhibitor of the formation of melanin precursors; All-trans retinoic acid	Solar lentigines	12/10/1999	S
28	Moxifloxacin HCl (Avelox)	Bayer	Topoisomerase II (DNA gyrase) and IV inhibitor	Sinusitis, pneumonia, and secondary infections in chronic bronchitis	12/10/1999	S
29	Dexmedetomidine HCl (Precedex)	Hospira	α2-adrenergic agonist	Sedation	12/17/1999	S, O
30	Gatifloxocin (Tequin)	Bristol-Myers Squibb	Topoisomerase II (DNA gyrase) and IV inhibitor	Bronchitis, sinusitis, pneumonia, and skin infections caused by *S. pneumoniae*, *H. influenzae*, *S. aureus*, *M. pneumoniae*, *C. pneumoniae*, *L. pneumophila*, and *S. pyogenes*	12/17/1999	N/A
31	Nitric oxide (Inomax)	Mallinckrodt Hosp	Guanylate cyclase activator	Hypoxic respiratory failure in premature neonates	12/23/1999	P, O
32	Bexarotene (Targretin)	Valeant Luxembourg	Retinoid, ligand of retinoid X receptors (RXRs)	Cutaneous T-cell lymphoma	12/29/1999	P, O
	**1999 Biologics license applications = 2**					
1	Denileukin diftitox (Ontak)	Eisai	Fusion protein consisting of diphtheria toxin fragments and interleukin-2	Cutaneous T-cell lymphoma	02/05/1999	O
2	Poractant alfa (Curosurf)	Chiesi USA	Natural porcine lung surfactant	Respiratory distress syndrome in premature infants	11/18/1999	O

Supplementary data: Table 1 – FDA-approved drugs (Continued)

	Drug (brand name)	Sponsor	Properties	Indication	Approval date	Review
	**2000** **New molecular entities = 24**					
1	Cevimeline HCl (Evoxac)	Daiichi Sankyo	Cholinergic agonist	Sjogren's syndrome	01/11/2000	S
2	Oxcarbazepine (Trileptal)	Novartis	Voltage-gated sodium channels inhibitor	Epilepsy	01/14/2000	S
3	Pantoprazole sodium (Protonix)	Wyeth Pharms	Proton pump inhibitor	Erosive esophagitis associated with gastroesophageal reflux disease	02/02/2000	S
4	Alosetron HCl (Lotronex)	Sebela Ireland	5-HT3 antagonist	Severe diarrhea-predominant irritable bowel syndrome (IBS) in women	02/09/2000	P
5	Perfluoropolymethylisopropyl ether; Polytetrafluoroethylene (Skin exposure reduction paste against chemical warfare agents)	US Army	Skin exposure reduction paste against chemical warfare agents	Contact dermatitis, friction-induced blisters, calluses and foot ulceration	02/17/2000	P
6	Zonisamide (Zonegran)	Concordia	Sodium and voltage sensitive calcium channels inhibitor	Epilepsy	03/27/2000	S
7	Articaine HCl; Epinephrine bitartrate (Septocaine)	Deproco	Articaine is a sodium channels blocker; Epinephrine is vasoconstrictor to slow absorption into the general circulation and prolong maintenance of an active tissue concentration	Local, infiltrative, or conductive anesthesia	04/03/2000	S
8	Verteporfin (Visudyne)	Valeant Luxembourg	Generation of highly reactive, short-lived singlet oxygen and reactive oxygen radicals by light in the presence of oxygen	Pathological myopia, ocular histoplasmosis, and choroidal neovascularization in macular degeneration	04/12/2000	P
9	Meloxicam (Mobic)	Boehringer Ingelheim	COX-2 inhibitor	Osteoarthritis and rheumatoid arthritis	04/13/2000	S, O
10	Linezolid (Zyvox)	Pfizer	Binds to bacterial 23S ribosomal RNA of the 50S subunit and prevents the formation of 70S initiation complex	Skin infections caused by *Staphylococcus aureus*, *Streptococcus pyogenes*, or *Streptococcus agalactiae*	04/18/2000	P
11	Rivastigmine tartrate (Exelon)	Novartis	Cholinesterase inhibitor	Mild-to-moderate Alzheimer's disease	04/21/2000	S
12	Colesevelam HCl (Welchol)	Daiichi Sankyo	Bile acid sequestrant	Hypercholesterolemia	05/26/2000	S
13	Triptorelin pamoate (Trelstar)	Verity	GnRH agonist	Advanced prostate cancer	06/15/2000	S
14	Argatroban	Novartis	Synthetic direct thrombin inhibitor derived from L-arginine	Thrombosis caused by heparin-induced thrombocytopenia	06/30/2000	S
15	Tinzaparin sodium (Innohep)	Leo Pharma	Low molecular weight heparin	Acute symptomatic deep vein thrombosis with or without pulmonary embolism	08/19/1999	S
16	Balsalazide disodium (Colazal)	Valeant Pharms	Mechanism of action of is unknown	Mild-to-moderate ulcerative colitis	07/18/2000	S
17	Docosanol (Abreva)	GlaxoSmithKline	Inhibiting fusion between the human cell plasma membrane and the herpes simplex virus (HSV) envelope	Oral-facial herpes simplex episodes	07/25/2000	S
18	Unoprostone isopropyl (Rescula)	Sucampo Pharma	Prostaglandin analog	Open-angle glaucoma or ocular hypertension	08/03/2000	P
19	Cetrorelix (Cetrotide)	Emd Serono	GnRH antagonist	Inhibition of premature LH surges in women undergoing controlled ovarian stimulation	08/11/2000	S
20	Lopinavir; Ritonavir (Kaletra)	Abbvie	HIV protease inhibitors	HIV-1 infection	09/15/2000	P
21	Arsenic trioxide (Trisenox)	Cephalon	Degradation of PML-RARα fusion protein	Acute promyelocytic leukemia characterized by the presence of the t (15; 17) translocation or PML/RARα gene expression	09/25/2000	P, O
22	Mifepristone (Mifeprex)	Danco Labs	Synthetic steroid, progestational and glucocorticoid hormone antagonist	Cushing's syndrome	09/28/2000	P
23	Bivalirudin (Angiomax)	Sandoz	Synthetic direct thrombin inhibitor	Heparin-induced thrombocytopenia and for the prevention of thrombosis	12/15/2000	S
24	Nateglinide (Starlix)	Novartis	Insulin secretagogue	Insulin independent diabetes	12/22/2000	S
	**2000** **Biologics license applications = 6**					
1	Insulin glargine recombinant (Lantus)	Sanofi	Recombinant human insulin [Gly (A21), Arg (B31, B32)]	Type 1 & 2 diabetes	04/20/2000	N/A
2	Gemtuzumab ozogamicin (Mylotarg)	Wyeth Pharms	CD33-directed antibody-drug conjugate (ADC)	Relapsed or refractory CD33-positive acute myeloid leukemia (AML)	05/17/2000	O
3	Tenecteplase (Tnkase)	Genentech	Recombinant human tissue plasminogen activator	Emergency treatment of myocardial infarction and pulmonary emboli	06/02/2000	N/A
4	Insulin aspart recombinant (Novolog)	Novo Nordisk	Recombinant human insulin [Asp (B28)]	Type 1 & 2 diabetes	06/07/2000	N/A
5	Choriogonadotropin alfa (Ovidrel)	Emd Serono	Recombinant human chorionic gonadotropin	Female infertility	09/20/2000	N/A
6	Botulinum toxin type B (Myobloc)	Elan Pharm	Purified botulinum toxin type B	Cervical dystonia	12/08/2000	O

Supplementary data: Table 1 – FDA-approved drugs (Continued)

	Drug (brand name)	Sponsor	Properties	Indication	Approval date	Review
	**2001**	**New molecular entities = 23**				
1	Caspofungin acetate (Cancidas)	Merck & Co	1,3-β glucan synthase inhibitor	*Aspergillus* and *Candida* infection	01/26/2001	P
2	Ziprasidone HCl (Geodon)	Viatris	5-HT$_{1D/2A}$ receptor and dopamine D2 receptor antagonist, 5-HT$_{1A}$ receptor agonist	Schizophrenia	02/05/2001	S
3	Formoterol fumarate (Foradil)	Novartis	β2-adrenergic receptor agonist	Asthma and chronic obstructive pulmonary disease (COPD)	02/16/2001	S
4	Galantamine HBr (Razadyne)	Janssen Pharms	Acetylcholinesterase inhibitor	Alzheimer's disease	02/28/2001	S
5	Travoprost (Travatan)	Alcon Pharms	Synthetic prostaglandin F2α analogt	Open angle glaucoma or ocular hypertension	03/16/2001	P
6	Bimatoprost (Lumigan)	Allergan	Synthetic prostaglandin analog	Open angle glaucoma or ocular hypertension	03/16/2001	P
7	Almotriptan malate (Axert)	Janssen Pharms	5-HT$_{1B/D}$ receptor agonist	Acute migraine headaches	05/07/2001	S
8	Imatinib mesylate (Gleevec)	Novartis	Tyrosine kinase inhibitor	Chronic myeloid leukemia	05/10/2001	P
9	Drospirenone; Ethinyl estradiol (Yasmin)	Bayer	Synthetic progestin and estrogen	Contraception	05/11/2001	S
10	Perflutren (Definity)	Lantheus Medcl	Ultrasound contrast agent	Ultrasound contrast imaging in cardiology and radiology	07/31/2001	S
11	Zoledronic acid (Zometa)	Novartis	Bone-resorption inhibitor	Hypercalcemia of malignancy	08/20/2001	P, O
12	Cefditoren pivoxil (Spectracef)	Vansen Pharma	Inhibition of cell wall synthesis	Chronic bronchitis, pneumonia, pharyngitis/tonsillitis, and skin infections	08/29/2001	S
13	Ethinyl estradiol; Etonogestrel (Nuvaring)	Organon	Synthetic estrogen; Synthetic derived progestin	Contraception	10/03/2001	S
14	Tenofovir disoproxil fumarate (Viread)	Gilead Sciences	HIV-1 and HBV reverse transcriptase inhibitor	HIV-1 and HBV infection	10/26/2001	P
15	Frovatriptan succinate (Frova)	Endo Pharms	5-HT$_{1B/1D}$ receptor agonist	Migraine headaches	11/08/2001	S
16	Valdecoxib (Bextra)	GD Searle	Cycooxygenase-2 (COX-2) inhibitor	Osteoarthritis and dysmenorrhea	11/16/2001	S
17	Ethinyl estradiol; Norelgestromin (Ortho evra)	Janssen Pharms	Synthetic estrogen and progestin	Contraception	11/20/2001	S
18	Bosentan (Tracleer)	Actelion	Endothelin receptor antagonist	Pulmonary arterial hypertension	11/20/2001	S, O
19	Dutasteride (Avodart)	Woodward	5α-reductase inhibitor	Benign prostatic hyperplasia	11/20/2001	S
20	Ertapenem sodium (Invanz)	Merck & Co	Inhibition of cell wall synthesis	Urinary tract infection	11/21/2001	S
21	Fondaparinux sodium (Arixtra)	Mylan Ireland	Antithrombin III activator	Deep vein thrombosis (DVT)	12/07/2001	P
22	Pimecrolimus (Elidel)	Bausch	Calcineurin inhibitor	Mild to moderate atopic dermatitis	12/13/2001	S
23	Desloratadine (Clarinex)	Organon	Histamine H1 receptor antagonist	Allergic rhinitis and chronic idiopathic urticaria	12/21/2001	S
	**2001**	**Biologics license applications = 5**				
1	Peginterferon alfa-2B (Pegintron)	Schering	Purified human interferon	HCV infection	01/19/2001	O
2	Alemtuzumab (Campath)	Genzyme	CD52-directed mAb	B-cell chronic lymphocytic leukemia	05/07/2001	O
3	Nesiritide recombinant (Natrecor)	Scios	Recombinant human B-type natriuretic peptide	Acutely decompensated congestive heart failure	08/10/2001	S
4	Darbepoetin alfa (Aranesp)	Amgen	Recombinant human erythropoietin	Anemia	09/17/2001	N/A
5	Anakinra (Kineret)	Biovitrum	Recombinant human interleukin-1 receptor antagonist	Rheumatoid arthritis and neonatal-onset multisystem inflammatory disease	11/14/2001	O

Supplementary data: Table 1 – FDA-approved drugs (Continued)

	Drug (brand name)	Sponsor	Properties	Indication	Approval date	Review
	**2002** **New molecular entities = 17**					
1	Nitisinone (Orfadin)	Swedish Orphan	4-hydroxyphenyl-pyruvate dioxygenase inhibitor, involved in tyrosine catabolism	Hereditary tyrosinaemia type 1	01/18/2002	P, O
2	Olmesartan medoxomil (Benicar)	Daiichi Sankyo	Angiotensin II type 1 receptor antagonist	Hypertension	04/25/2002	S
3	Fulvestrant (Faslodex)	AstraZeneca	Estrogen receptor antagonist	HR-positive metastatic breast cancer	04/25/2002	S
4	Treprostinil sodium (Remodulin)	United Therap	Prostacyclin analog, vasodilator and platelet-aggregation inhibitor	Pulmonary arterial hypertension	05/21/2002	P, O
5	Voriconazole (Vfend)	Merck & Co	Triazole, inhibitor of cytochrome P450-mediated 14 α-lanosterol demethylation	Invasive aspergillosis and serious fungal infections caused by *Scedosporium apiospermum* and *Fusarium spp*	05/24/2002	S
6	Dimyristoyl lecithin; Perflexane (Imagent)	Vesselon SPV	*n*-perfluorohexane (perflexane) and non-animal semi-synthetic phospholipid (DMPC), increases ultrasound reflectivity of blood	Patients with suboptimal echocardio-grams to opacify the left ventricular chamber and to improve the delineation of the left ventricular endocardial border	05/31/2002	S
7	Sodium oxybate (Xyrem)	Jazz	γ-hydroxybutyrate, central nervous system depressant	Narcolepsy with episodes of cataplexy	07/17/2002	P, O
8	Tegaserod maleate (Zelnorm)	Alfasigma	5-HT_4 receptor agonist	Irritable bowel syndrome	07/24/2002	P
9	Oxaliplatin (Eloxatin)	Sanofi	Organoplatinum alkylating agent, increases tumor response rate to chemotherapy	Colorectal cancer (in combination with 5-fluorouracil and leucovorin)	08/09/2002	P
10	Adefovir dipivoxil (Hepsera)	Gilead Sciences	Acyclic nucleocide phosphonate, inhibitor of hepatitis B virus DNA polymerase	Chronic hepatitis B	09/20/2002	P
11	Eplerenone (Inspra)	Upjohn	Selective aldosterone receptor antagonist	Hypertension	09/27/2002	N/A
12	Ezetimibe (Zetia)	Organon	Inhibits intestinal cholesterol absorption, NPC1L1 blocker	Primary hyperlipidemia and familial cholesterolemia	10/25/2002	S
13	Aripiprazole (Abilify)	Otsuka	Serotonin and dopamine receptors agonist	Schizophrenia	11/15/2002	S, O
14	Nitazoxanide (Alinia)	Romark	Antiprotozoal agent	Paediatric diarrhoea caused by *Cryptosporidium spp*. and *Giardia spp*.	11/22/2002	P, O
15	Atomoxetine HCl (Strattera)	Eli Lilly	Selective noradrenaline-reuptake inhibitor	Attention-deficit hyperactivity disorder	11/26/2002	S
16	Icodextrin (Extraneal)	Baxter Hlthcare	Isosmotic peritoneal dialysis solution containing glucose polymers	Continuous ambulatory peritoneal dialysis of diabetic patients, automated peritoneal dialysis for the management of end-stage renal disease	12/20/2002	S, O
17	Eletriptan HCl (Relpax)	Upjohn	Selective $5\text{-HT}_{1B/D}$ receptor agonist	Migraine	12/26/2002	S
	**2002** **Biologics license applications = 4**					
1	Pegfilgrastim (Neulasta)	Amgen	PEGylated form of recombinant human granulocyte colony-stimulating factor (G-CSF)	Febrile neutropenia or myelosuppressive chemotherapy-induced neutropenia	01/31/2002	N/A
2	Ibritumomab tiuxetan (Zevalin)	IDEC Pharmas	^{111}In and ^{90}Y labelled CD20-specific radiotherapeutic mAb	B-cell non-Hodgkin's lymphoma	02/19/2002	O
3	Peginterferon alfa-2A (Pegasys)	Roche	Recombinant human interferon	Hepatitis B and C virus infection	10/16/2002	N/A
4	Adalimumab (Humira)	AbbVie	TNFα-directed mAb	Rheumatoid arthritis, Crohn's disease, and ankylosing spondylitis	12/31/2002	O

Supplementary data: Table 1 – FDA-approved drugs (Continued)

	Drug (brand name)	Sponsor	Properties	Indication	Approval date	Review
**2003**	**New molecular entities = 20**					
1	Enfuvirtide (Fuzeon)	Roche	HIV-1 gp41 fusion inhibitor	HIV-1 infection	03/13/2003	P
2	Aprepitant (Emend)	Merck & Co	NK1 receptor antagonist	Chemotherapy-induced nausea	03/26/2003	P
3	Gemifloxacin mesylate (Factive)	LG Chem	Bacterial topoisomerase II (DNA gyrase) and topoisomerase IV inhibitor	Bacterial infections, including *S. pneumoniae*, *H. influenzae*, *H. parainfluenzae*, or *M. catarrhalis*, *S. pneumoniae*, et al.	04/04/2003	S
4	Gefitinib (Iressa)	AstraZeneca	EGFR kinase inhibitor	Non-small-cell lung cancer	05/05/2003	P
5	Bortezomib (Velcade)	Takeda Pharms	Proteasome inhibitor	Multiple myeloma	05/13/2003	P, O
6	Ibandronate sodium (Boniva)	Roche	Bisphosphonate	Osteoporosis	05/16/2003	S
7	Alfuzosin HCl (Uroxatral)	Concordia	α1-adrenoceptor antagonist	Benign prostate hyperplasia	06/12/2003	S
8	Atazanavir sulfate (Reyataz)	Bristol-Myers Squibb	HIV protease inhibitor	HIV-1 infection	06/20/2003	P
9	Emtricitabine (Emtriva)	Gilead Sciences	HIV reverse transcriptase inhibitor	HIV-1 infection	07/02/2003	S
10	Palonosetron HCl (Aloxi)	Helsinn	5-HT_3 receptor antagonist	Chemotherapy-induced nausea	07/25/2003	S
11	Miglustat (Zavesca)	Actelion	Glucosylceramide synthase inhibitor	Gaucher's disease	07/31/2003	S, O
12	Rosuvastatin calcium (Crestor)	IPR	HMG CoA reductase inhibitor	Hypercholesterolemia	08/12/2003	S
13	Vardenafil HCl (Levitra)	Bayer	Phosphodiesterase 5 (PDE5) inhibitor	Erectile dysfunction	08/19/2003	S
14	Daptomycin (Cubicin)	Cubist Pharms	Cyclic lipopeptide, cell membrane/wall constituent biosynthesis inhibitor	Skin infections, or bloodstream infections caused by *Staphylococcus aureus*	09/12/2003	P
15	Ferric hexacyanoferrate II (Radiogardase [Prussian blue])	Heyl Chemisch	Metal-binding complex	Systemic contamination with radioactive cesium and/or radioactive or non-radioactive thallium	10/02/2003	P, O
16	Memantine HCl (Namenda)	Allergan	NMDA receptor antagonist	Alzheimer's disease	10/16/2003	S
17	Epinastine HCl (Elestat)	Allergan	Histamine H1-receptor antagonist	Hay fever	10/16/2003	S
18	Tadalafil (Cialis)	Lilly	Phosphodiesterase 5 (PDE5) inhibitor	Erectile dysfunction	11/21/2003	S
19	Abarelix (Plenaxis)	Speciality European	GnRH receptor antagonist	Advanced prostate cancer	11/25/2003	P
20	Sertaconazole nitrate (Ertaczo)	Bausch	Imidazole-derivative antifungal	Tinea pedis	12/10/2003	S
**2003**	**Biologics license applications = 8**					
1	Alefacept (Amevive)	Biogen	CD2-directed mAb	Psoriasis	01/31/2003	N/A
2	Pegvisomant (Somavert)	Pharmacia	Growth hormone receptor antagonist	Acromegaly	03/25/2003	O
3	Desirudin recombinant (Iprivask)	Bausch	Direct human thrombin inhibitor	Prophylaxis of deep vein thrombosis for patients undergoing hip replacement surgery	04/04/2003	N/A
4	Agalsidase beta (Fabrazyme)	Genzyme	Recombinant human α-galactosidase A	Fabry disease	04/24/2003	O
5	Laronidase (Aldurazyme)	Biomarin	Recombinant α-L-iduronidase	Mucopolysaccharidosis type 1	04/30/2003	O
6	Omalizumab (Xolair)	Genentech	IgE-directed mAb	Asthma	06/20/2003	N/A
7	Tositumomab and iodine I-131 tositumomab (Bexxar)	Corixa	CD20-directed radiotherapeutic mAb	Non-Hodgkin's lymphoma	06/30/2003	P, O
8	Efalizumab (Raptiva)	Genentech	CD11a-directed mAb	Psoriasis	10/27/2003	N/A

Supplementary data: Table 1 – FDA-approved drugs (Continued)

	Drug (brand name)	Sponsor	Properties	Indication	Approval date	Review
	**2004** New molecular entities = 28					
1	Tiotropium bromide (Spiriva)	Boehringer Ingelheim	Muscarinic M1/5 receptors antagonist	Chronic obstructive pulmonary disease (COPD)	01/30/2004	S
2	Pemetrexed disodium (Alimta)	Eli Lilly	Folate analog	Malignant pleural mesothelioma	02/04/2004	P, O
3	Cinacalcet HCl (Sensipar)	Amgen	Calcimimetic, calcium sensing receptor agonist	Secondary hyper-parathyroidism, hypercalcaemia	03/08/2004	P, O
4	Telithromycin (Ketek)	Sanofi	Ketolide antibiotic	Respiratory infections	04/01/2004	S
5	Secretin synthetic human (Chirhostim)	ChiRhoClin	Pancreatic and gastrin secretagogue	Exocrine pancreas dysfunction, gastrinoma (diagnostic)	04/09/2004	P
6	Apomorphine HCl (Apokyn)	MDD US	Non-ergoline dopamine D2 agonist	Parkinson's disease	04/20/2004	P, O
7	Tinidazole (Tindamax)	Mission Pharma	Antiprotozoal agent	Giardiasis, amoebiasis and trichomoniasis	05/17/2004	S, O
8	Azacitidine (Vidaza)	Celgene	Antineoplastic pyrimidine nucleoside analogue, DNA methyltransferase inhibitor	Myelodysplastic syndrome	05/19/2004	P, O
9	Rifaximin (Xifaxan)	Salix Pharmas	Rifamycin-based non-systemic antibiotict	Traveler's diarrhea	05/25/2004	S, O
10	Trospium chloride (Sanctura)	Allergan	Antimuscarinic antispasmodic	Overactive bladder	05/28/2004	S
11	L-Glutamine (NutreStore)	Emmaus Medcl	Amino acid supplement	Short bowel syndrome	06/10/2004	S, O
12	Acamprosate calcium (Campral)	Forest Labs	Neurotransmitter γ-aminobutyric acid (GABA) analog	Alcohol dependence	07/29/2004	P
13	Duloxetine HCl (Cymbalta)	Lilly	Serotonin- and norepinephrine-reuptake inhibitor	Diabetic peripheral neuropathy	08/03/2004	S
14	Pentetate calcium trisodium	Hameln	Chelating agent	Internal radioactive contamination	08/11/2004	P, O
15	Pentetate zinc trisodium	Hameln	Chelating agent	Internal radioactive contamination	08/11/2004	P, O
16	Pegaptanib sodium (Macugen)	Valeant Pharms	Selective VEGF antagonist	Neovascular age-related macular degeneration	09/17/2004	P
17	Lanthanum carbonate (Fosrenol)	Takeda	Phosphate binder	Hyperphosphatemia in end-stage renal failure	10/26/2004	S
18	ω3-acid ethyl esters (Lovaza)	Woodward	Lipid-regulating agent	Hypertriglyceridemia	11/10/2004	S
19	Erlotinib HCl (Tarceva)	OSI Pharmas	EGFR inhibitor	Non-small-cell lung cancer (NSCLC)	11/18/2004	P
20	Solifenacin succinate (Vesicare)	Astellas	Muscarinic M2/3 receptors antagonist	Overactive bladder	11/19/2004	S
21	Gadobenate dimeglumine (Multihance)	Bracco	Gadolinium-based MRI contrast agent	MRI imaging of liver, heart and central nervous system (CNS)	11/23/2004	S
22	Eszopiclone (Lunesta)	Sunovion Pharms	Non-benzodiazepine hypnotic, GABA-A receptor stimulator	Insomnia	12/15/2004	S
23	Trypan blue (Visionblue)	DORC	Selective staining of the anterior lens capsule	Cataract surgery	12/16/2004	P
24	Darifenacin HBr (Enablex)	APIL	Selective muscarinic M3 receptor antagonist	Overactive bladder	12/22/2004	S
25	Ziconotide acetate (Prialt)	Tersera	N-type calcium channel blocker	Severe chronic pain	12/28/2004	P
26	Clofarabine (Clolar)	Genzyme	Nucleoside antimetabolite, DNA synthesis inhibitor	Relapsed or refractory acute lymphoblastic leukemia	12/28/2004	P, O
27	Iloprost (Ventavis)	Actelion	Prostacyclin analog	Pulmonary arterial hypertension	12/29/2004	P, O
28	Pregabalin (Lyrica)	Upjohn	Neurotransmitter γ-aminobutyric acid (GABA) analog	Diabetic peripheral neuropathy and post-herpetic neuralgia pain	12/30/2004	P
	**2004** Biologics license applications = 8					
1	Cetuximab (Erbitux)	ImClone	EGFR-directed mAb	Colorectal cancer	02/12/2004	N/A
2	Bevacizumab (Avastin)	Genentech	VEGF-A-directed mAb	Colorectal cancer	02/26/2004	O
3	Insulin glulisine recombinant (Apidra)	Sanofi	Recombinant human insulin [Lys (B3), Glu (B29)]	Type 1 and type 2 diabetes	04/16/2004	N/A
4	Hyaluronidase (Vitrase)	Bausch & Lomb	Connective-tissue-permeability modifier (Ovine)	Adjunct in subcutaneous urography for improving resorption of radiopaque agents	05/05/2004	N/A
5	Hyaluronidase (Amphadase)	Amphastar Pharm	Connective-tissue-permeability modifier (Bovine)		10/26/2004	N/A
6	Technetium (99m Tc) fanolesomab (NeutroSpec)	Palatin Technologies	^{99m}Tc labelled CD15-directed mAb	Imaging and detection of appendicitis	07/02/2004	N/A
7	Natalizumab (Tysabri)	Biogen Idec	α4-integrin-directed mAb	Crohn's disease or multiple sclerosis	11/23/2004	N/A
8	Palifermin (Kepivance)	Biovitrum	Recombinant human keratinocyte growth factor	Oral mucositis following radiation or chemotherapy	12/15/2004	N/A

Supplementary data: Table 1 – FDA-approved drugs (Continued)

	Drug (brand name)	Sponsor	Properties	Indication	Approval date	Review
	**2005** **New molecular entities = 13**					
1	Pramlintide acetate (Symlin)	AstraZeneca	Amylin analog, glucoregulatory hormone	Type 1 and 2 diabetes	03/16/2005	S
2	Micafungin sodium (Mycamine)	Astellas	Inhibits fungal cell wall by inhibiting β-1,3-D-glucan synthesis	Candida infections in patients undergoing hematopoietic stem cell transplantation	03/16/2005	S
3	Entecavir (Baraclude)	Bristol-Myers Squibb	Nucleoside analog	Chronic hepatitis B virus infection	03/29/2005	P
4	Exenatide synthetic (Byetta)	AstraZeneca	Incretin mimetics, human glucacon-like peptide-1(GLP-1) receptor agonist	Type 2 diabetes	04/28/2005	S
5	Tigecycline (Tygacil)	Merck & Co	Tetracycline antibiotic, inhibits protein translation in bacteria by binding to the 30S ribosomal subunit	Complicated skin and skin structure infections and complicated intra-abdominal infections	06/15/2005	P
6	Tipranavir (Aptivus)	Boehringer Ingelheim	HIV-1 protease inhibitor	HIV-1 infection	06/22/2005	P
7	Ramelteon (Rozerem)	Takeda	Melatonin receptor agonist	Insomnia	07/22/2005	S
8	Nepafenac (Nevanac)	Novartis	Nonsteroidal anti-inflammatory prodrug	Pain and inflammation associated with cataract surgery	08/19/2005	P
9	Nelarabine (Arranon)	Novartis	Purine nucleoside analog, inhibits the DNA elongation	T-cell acute lymphoblastic leukaemia and T-cell lymphoblastic lymphoma	10/28/2005	P, O
10	Deferasirox (Exjade)	Novartis	Iron chelator	Chronic iron overload due to blood transfusions	11/02/2005	P, O
11	Sorafenib tosylate (Nexavar)	Bayer	Multi-target TKI (VEGFR2/3, PDGFRβ, FLT3, KIT, RAF1, and BRAF)	Advanced renal cell carcinoma	12/01/2005	P, O
12	Lenalidomide (Revlimid)	Celgene	Thalidomide derivative, immunomodulatory and antiangiogenic agent	Transfusion-dependent anemia due to myelodysplastic syndromes associated with a deletion 5q abnormality with or without additional cytogenetic abnormalities	12/27/2005	P, O
13	Conivaptan HCl (Vaprisol)	Cumberland Pharms	Antidiuretic hormone (Vasopressin) inhibitor	Euvolemic hyponatremia in hospitalized patients	12/29/2005	S
	**2005** **Biologics license applications = 7**					
1	Galsulfase (Naglazyme)	Biomarin	Recombinant human N-acetylgalactosamine 4-sulfatase	Mucopolysaccharidosis VI	05/31/2005	P, O
2	Insulin detemir recombinant (Levemir)	Novo Nordisk	Recombinant human insulin [ΔThr (B30)]	Type 1 and 2 diabetes	06/16/2005	N/A
3	Mecasermin recombinant (Increlex)	Ipsen	Recombinant DNA-origin human insulin-like growth factor 1 (IGF-1)	Growth failure in pediatric patients with primary IGF-1 deficiency or growth hormone gene deletion	08/30/2005	P, O
4	Hyaluronidase (Hydase)	Akorn	Connective-tissue-permeability modifier (Bovine)	Hypodermoclysis, and in subcutaneous urography for improving resorption of radiopaque agents	10/25/2005	P
5	Hyaluronidase recombinant human (Hylenex recombinant)	Halozyme Therap	Connective-tissue-permeability modifier (Human)	Subcutaneous urography for improving resorption of radiopaque agents	12/02/2005	P
6	Mecasermin rinfabate recombinant (iPlex)	Insmed	Recombinant DNA-origin IGF-1	Growth failure in children with severe primary IGF-1 deficiency	12/12/2005	O
7	Abatacept (Orencia)	Bristol-Myers Squibb	Selective T-cell co-stimulation modulator	Rheumatoid arthritis	12/23/2005	P

Supplementary data: Table 1 – FDA-approved drugs (Continued)

	Drug (brand name)	Sponsor	Properties	Indication	Approval date	Review
	**2006** **New molecular entities = 18**					
1	Sunitinib malate (Sutent)	CPPI CV	Multi-target TKI (VEGFRs, PDGFRα/β, CSF1R, KIT, and FLT3)	Imatinib-resistant gastrointestinal stromal tumor and advanced renal cell carcinoma	01/26/2006	P
2	Ranolazine (Ranexa)	Gilead Sciences	Sodium and potassium ion channel inhibitor	Chronic angina	01/27/2006	S
3	Lubiprostone (Amitiza)	Sucampo Pharma	Chloride channel inhibitor	Constipation	01/31/2006	S
4	Anidulafungin (Eraxis)	Vicuron Holdings	Fungal cell-wall synthesis inhibitor	Esophageal candidiasis	02/17/2006	S
5	Decitabine (Dacogen)	Otsuka	Demethylating agent	Myelodysplastic syndrome	05/02/2006	S, O
6	Varenicline tartrate (Chantix)	Merck & Co	Nicotinic acetylcholine receptor (nACh-R) agonist	Smoking cessation	05/10/2006	P
7	Rasagiline mesylate (Azilect)	Teva	Monoamine oxidase B inhibitor	Parkinson’s disease	05/16/2006	S
8	Darunavir (Prezista)	Janssen Prods	HIV-1 protease inhibitor	HIV-1 infection	06/23/2006	P
9	Dasatinib (Sprycel)	Bristol-Myers Squibb	Tyrosine kinase inhibitor	Chronic myeloid leukemia	06/28/2006	P, O
10	Avobenzone; Ecamsule; Octocrylene (Anthelios SX)	Loreal USA	UVA/UVB filter	Prevention of sunburn	07/21/2006	S
11	Posaconazole (Noxafil)	Schering	Fungal cell-wall synthesis inhibitor	*Aspergillus* and *Candida* infection	09/15/2006	P
12	Bismuth subcitrate potassium; Metronidazole; Tetracycline HCl (Pylera)	Allergan	Bismuth subcitrate potassium inhibits peptic activity; Metronidazole inhibits bacterial and protozoal nucleic acid synthesis; Tetracycline HCl interferes with protein synthesis by preventing binding of tRNA to the mRNA-ribosome complex	Gastric and duodenal ulcers caused by *H. pylori* infection	09/28/2006	S
13	Vorinostat (Zolinza)	Merck & Co	Histone deacetylase inhibitor	Cutaneous T-cell lymphoma	10/06/2006	P, O
14	Sitagliptin phosphate (Januvia)	Merck & Co	DPP4 inhibitor	Type 2 diabetes	10/16/2006	S
15	Ciclesonide (Omnaris)	Covis	Corticosteroid	Allergic rhinitis	10/20/2006	S
16	Telbivudine (Tyzeka)	Novartis	Reverse transcriptase inhibitor	Chronic hepatitis B	10/25/2006	S
17	Sinecatechins (Veregen)	Ani Pharms	Antioxidant	External genital and perianal warts	10/31/2006	S
18	Paliperidone (Invega)	Janssen Pharms	Dopamine D2 and 5-HT_{2A} receptor antagonist	Schizophrenia	12/19/2006	S
	**2006** **Biologics license applications = 4**					
1	Alglucosidase alfa (Myozyme)	Genzyme	Recombinant human α-glucosidase	α-glucosidase deficiency (Pompe disease)	04/28/2006	O
2	Ranibizumab (Lucentis)	Genentech	VEGF-A-directed mAb fragment	Neovascular (wet) age-related macular degeneration	06/30/2006	N/A
3	Idursulfase (Elaprase)	Shire	Recombinant human iduronate-2-sulfatase	Hunter syndrome (Mucopolysaccharidosis II)	07/24/2006	O
4	Panitumumab (Vectibix)	Amgen	EGFR-directed mAb	EGFR-expressing metastatic colorectal carcinoma	09/27/2006	N/A

Supplementary data: Table 1 – FDA-approved drugs (Continued)

	Drug (brand name)	Sponsor	Properties	Indication	Approval date	Review
	**2007** **New molecular entities = 17**					
1	Lisdexamfetamine dimesylate (Vyvanse)	Takeda	Prodrug of dextroamphetamine with CNS stimulant activity	Attention-deficit/hyperactivity disorder	02/23/2007	S
2	Aliskiren Hemifumarate (Tekturna)	Noden Pharma	Direct renin inhibitor	Hypertension	03/05/2007	S
3	Lapatinib Ditosylate (Tykerb)	Novartis	HER2 and EGFR inhibitor	Advanced or metastatic breast cancer (HER2-positive)	03/13/2007	P
4	Retapamulin (Altabax)	Almirall	Bacterial protein-synthesis inhibitor	Impetigo	04/12/2007	S
5	Rotigotine (Neupro)	UCB Inc	Dopamine receptor agonist	Early-stage idiopathic Parkinson’s disease	05/09/2007	S
6	Temsirolimus (Torisel)	Merck & Co	Mammalian target of rapamycin (mTOR) inhibitor	Advanced renal cell carcinoma	05/30/2007	P, O
7	Ambrisentan (Letairis)	Gilead Sciences	Endothelin receptor antagonist	Pulmonary arterial hypertension	06/15/2007	P, O
8	Maraviroc (Selzentry)	Viiv Hlthcare	CCR5 co-receptor antagonist	CCR5-tropic HIV-1	08/06/2007	P
9	Ammonia N-13 (Ammonia N 13)	Feinstein	^{13}N labelled labelled radioactive diagnostic agent	Radioactive agent for positron emission tomography diagnosis of coronary artery disease	08/23/2007	S
10	Lanreotide (Somatuline depot)	Ipsen Pharma	Somatostatin analog	Acromegaly	08/30/2007	S, O
11	Doripenem (Doribax)	Shionogi	Synthetic broad-spectrum carbapenem antibiotic	Urinary tract infections	10/12/2007	S
12	Raltegravir potassium (Isentress)	Merck & Co	HIV integrase strand transfer inhibitor	HIV-1 infection	10/12/2007	P
13	Ixabepilone (Ixempra)	R-Pharm US	Microtubule-stabilizing agent	Advanced or metastatic breast cancer	10/16/2007	P
14	Nilotinib HCl (Tasigna)	Novartis	BCR-ABL kinase inhibitor	Chronic myelogenous leukemia	10/29/2007	S, O
15	Sapropterin 2HCl (Kuvan)	Biomarin Pharm	Synthetic tetrahydrobiopterin, co-factor for the enzyme phenylalanine hydroxylase	Hyperphenylalaninemia	12/13/2007	P, O
16	Nebivolol HCl (Bystolic)	Allergan	β-adrenoceptor antagonist	Hypertension	12/17/2007	S
17	Hydroxyethyl starch (Voluven)	Fresenius Kabi	Blood volume expander	Serious blood volume loss	12/27/2007	N/A
	**2007** **Biologics license applications = 2**					
1	Eculizumab (Soliris)	Alexion Pharm	Complement protein C5-directed mAb	Paroxysmal nocturnal hemoglobinuria	03/16/2007	P, O
2	Methoxy polyethylene glycol-epoetin beta (Mircera)	Roche	Erythropoietin receptor activator	Anemia associated with chronic renal failure	11/14/2007	P

Supplementary data: Table 1 – FDA-approved drugs (Continued)

	Drug (brand name)	Sponsor	Properties	Indication	Approval date	Review
	**2008** **New molecular entities = 22**					
1	Etravirine (Intelence)	Janssen R&D	Non-nucleoside HIV-1 reverse transcriptase inhibitor	HIV-1 infection	01/18/2008	P
2	Fosaprepitant dimeglumine (Emend)	Merck & Co	Prodrug of aprepitant, NK1 receptor antagonist	Nausea and vomiting caused by chemotherapy	01/25/2008	S
3	Desvenlafaxine Succinate (Pristiq)	Pfizer	Selective serotonin and noradrenaline reuptake inhibitor	Major depressive disorder	02/29/2008	S
4	Bendamustine HCl (Treanda)	Cephalon	Mechlorethamine derivative with DNA-alkylating activity	Chronic lymphocytic leukemia	03/20/2008	P, O
5	Regadenoson (lexiscan)	Astellas	A_{2A} adenosine receptor agonist	Pharmacological stress agent for radionuclide imaging	04/10/2008	S
6	Methylnaltrexone bromide (Relistor)	Salix Pharms	Peripherally acting μ opioid receptor antagonist	Opioid-induced constipation	04/24/2008	S
7	Alvimopan (Entereg)	Cubist Pharms	Peripherally acting μ opioid receptor antagonist	To accelerate gastrointestinal recovery following bowel resection surgery	05/20/2008	S
8	Difluprednate (Durezol)	Novartis	Ocular corticosteroid thought to act by the induction of phospholipase A2 inhibitory proteins	Inflammation and pain associated with ocular surgery	06/23/2008	P, O
9	Gadoxetate disodium (Eovist)	Bayer	Gadolinium-based contrast agent	MRI diagnosis of liver lesions	07/03/2008	S
10	Clevidipine (Cleviprex)	Chiesi	Short-acting dihydropyridine calcium channel antagonist	Peri-operative hypertension when oral therapy is not feasible or not desirable	08/01/2008	S
11	Tetrabenazine (Xenazine)	Bausch	Monoamine-depleting agent	Chorea associated with Huntington's disease	08/15/2008	P, O
12	Iobenguane sulfate I-123 (AdreView)	GE Healthcare	Taken up by the noradrenaline transporter in adrenergic nerve terminals	Radiopharmaceutical agent, detection of primary or metastatic phaeochromocytoma or neuroblastoma	09/19/2008	P, O
13	Silodosin (Rapaflo)	Allergan	α1-adrenoceptor antagonist	Benign prostatic hyperplasia	10/08/2008	S
14	Lacosamide (Vimpat)	UCB Inc	Selectively enhances slow inactivation of voltage-gated sodium channels and binds to collapsin response mediator protein 2	Partial-onset seizures in epilepsy	10/28/2008	S
15	Fesoterodine fumarate (Toviaz)	Pfizer	Competitive muscarinic receptor antagonist	Overactive bladder disorder	10/31/2008	S
16	Rufinamide (Banzel)	Eisai	Sodium channel activity modulator	Seizures associated with Lennox-Gastaut syndrome	11/14/2008	S
17	Eltrombopag olamine (Promacta)	Novartis	Thrombopoietin receptor agonist	Thrombocytopenia in patients withchronic immune (idiopathic) thrombocytopenic purpura	11/20/2008	P, O
18	Tapentadol HCl (Nucynta)	Collegium Pharm	μ opioid receptor agonist and noradrenaline reuptake inhibitor	Moderate to severe acute pain	11/20/2008	S
19	Fospropofol disodium (lusedra)	Eisai	Prodrug of propofol	Monitored anesthesia care sedation	12/12/2008	S
20	Plerixafor (Mozobil)	Genzyme	CXCR4 antagonist	Autologous transplantation in patients with non-Hodgkin's lymphoma and multiple myeloma	12/15/2008	P, O
21	Gadofosveset Trisodium (Vasovist)	Lantheus Medcl	Gadolinium-based contrast agent	Magnetic resonance angiography (MRA) diagnosis of aortoiliac occlusive disease (AIOD)	12/22/2008	S
22	Degarelix acetate (Firmagon)	Ferring	GnRH receptor antagonist	Advanced prostate cancer	12/24/2008	S
	**2008** **Biologics license applications = 5**					
1	Somatropin (Accretropin)	Emergent	Recombinant human growth hormone	Growth failure, growth hormone deficiency	01/23/2008	N/A
2	Rilonacept (Arcalyst)	Kiniksa Pharma (UK) Ltd	IL-1 blocker, dimeric fusion protein consisting of portions of IL-1R and the IL-1R accessory protein linked to the Fc portion IgG1	Cryopyrin-associated periodic syndromes including familial cold autoinflammatory syndrome and Muckle-wells syndrome	02/27/2008	P, O
3	Certolizumab pegol (Cimzia)	UCB Inc	TNFα blocker, pegylated anti-TNFα IgG1 (Fab)	Crohn's disease	04/22/2008	S
4	Romiplostim (Nplate)	Amgen	Thrombopoietin receptor agonist, thrombopoiesis stimulating dimer Fc-peptide fusion protein (Peptibody)	Thrombocytopenia in patients with chronic immune (idiopathic) thrombocytopenic purpura	08/22/2008	P, O
5	Insulin aspart protamine recombinant; Insulin aspart recombinant (Novolog mix 50/50)	Novo Nordisk	Recombinant human insulin [Asp (B28)]	Type 1 & 2 diabetes	08/26/2008	N/A

Supplementary data: Table 1 – FDA-approved drugs (Continued)

	Drug (brand name)	Sponsor	Properties	Indication	Approval date	Review
	**2009** **New molecular entities = 20**					
1	Milnacipran HCl (Savella)	Allergan	Selective serotonin and noradrenaline reuptake inhibitor	Fibromyalgia	01/14/2009	S
2	Febuxostat (Uloric)	Takeda	Xanthine oxidase inhibitor	Hyperuricemia in patients with gout	02/13/2009	S
3	Everolimus (Afinitor)	Novartis	Mammalian target of rapamycin (mTOR) inhibitor	Advanced renal cell carcinoma	03/30/2009	P
4	Artemether; Lumefantrine (Coartem)	Novartis	Works against the erythrocytic stages of *P. falciparum* by inhibiting nucleic acid and protein synthesis	Malaria	04/07/2009	P, O
5	Benzyl alcohol (Ulesfia)	Shionogi	Closes the respiratory spiracles of lice and causes the lice to asphyxiate	Head lice	04/09/2009	S
6	Iloperidone (Fanapt)	Vanda Pharms	Atypical antipsychotic agent, 5-HT_{2A} and dopamine D2 receptors antagonist	Schizophrenia	05/06/2009	S
7	Tolvaptan (Samsca)	Otsuka	Selective vasopressin V2 receptor antagonist	Hypervolemic and euvolemic hyponatremia, including patients with heart failure, cirrhosis and syndrome of inappropriate antidiuretic hormone	05/19/2009	S
8	Besifloxacin HCl (Besivance)	Bausch & Lomb	Fluoroquinolone antimicrobial agent that inhibits bacterial topoisomerase II (DNA gyrase) and topoisomerase IV	Bacterial conjunctivitis	05/28/2009	S
9	Dronedarone HCl (Multaq)	Sanofi	Multi-channel blocker, including potassium, sodium, and calcium channels	Atrial fibrillation and atrial flutter	07/01/2009	P
10	Prasugrel HCl (Effient)	Daiichi Sankyo	Platelet $P2Y_{12}$ purinoceptor inhibitor	Reduction of thrombotic cardiovascular events in patients with acute coronary syndrome undergoing percutaneous coronary intervention	07/10/2009	P
11	Saxagliptin HCl (Onglyza)	AstraZeneca	Dipeptidyl peptidase 4 inhibitor	Type 2 diabetes	07/31/2009	S
12	Pitavastatin calcium (Livalo)	Kowa Co	HMG CoA reductase inhibitor	Hyperlipidemia and mixed dyslipidemia	08/03/2009	S
13	Asenapine Maleate (Saphris)	Allergan	Atypical antipsychotic agent, 5-HT2A and dopamine D2 receptors antagonist	Schizophrenia and bipolar disorder	08/13/2009	S
14	Vigabatrin (Sabril)	Lundbeck	Irreversible γ-aminobutyric acid transaminase inhibitor	Infantile spasms in children aged 1 month to 2 years and complex partial seizures in adults	08/21/2009	P, O, S
15	Bepotastine besilate (Bepreve)	Bausch & Lomb	Histamine H1 receptor antagonist	Allergic conjunctivitis	09/04/2009	S
16	Telavancin HCl (Vibativ)	Cumberland Pharms	Semi-synthetic derivative of vancomycin, bacterial cell wall synthesis inhibitor	Complicated skin and skin structure infections	09/11/2009	S
17	Pralatrexate (Folotyn)	Acrotech	Folate analogue metabolic inhibitor that competitively inhibits dihydrofolate reductase	Peripheral T cell lymphoma	09/24/2009	P, O
18	Pazopanib HCl (Votrient)	Novartis	Multi-target TKI (VEGFRs, PDGFRα/β, FGFR1/2, KIT)	Advanced renal cell carcinoma	10/19/2009	S
19	Romidepsin (Istodax)	Celgene	Histone deacetylase inhibitor	Cutaneous T cell lymphoma	11/05/2009	S
20	Capsaicin (Qutenza)	Averitas	Transient receptor potential vanilloid 1 (TRPV1) receptor agonist	Neuropathic pain associated with post-herpetic neuralgia	11/16/2009	S, O
	**2009** **Biologics license applications = 6**					
1	Golimumab (Simponi)	Centocor Ortho Biotech	TNFα-directed mAb	Rheumatoid arthritis, psoriatic arthritis, and ankylosing spondylitis	04/24/2009	S
2	Abobotulinum-toxin A (Dysport)	Ipsen Biopharm	Acetylcholine release inhibitor and euromuscular blocking agent	Cervical dystonia and glabellar lines	04/29/2009	S
3	Canakinumab (Ilaris)	Novartis	Interleukin-1β-directed mAb	Cryopyrin-associated periodic syndromes	06/17/2009	P, O
4	Ustekinumab (Stelara)	Centocor Ortho Biotech	Interleukin (IL)-12 and IL-23 (p40 subunit)-directed mAb	Moderate-to-severe plaque psoriasis	09/25/2009	S
5	Ofatumumab (Arzerra)	Novartis	CD20-directed mAb	Chronic lymphocytic leukemia	10/26/2009	P, O
	Ofatumumab (Kesimpta)			Relapsing forms of multiple sclerosis		
6	Ecallantide (Kalbitor)	Dyax Corp	Plasma kallikrein inhibitor	Hereditary angioedema	12/01/2009	P, O

Supplementary data: Table 1 – FDA-approved drugs (Continued)

	Drug (brand name)	Sponsor	Properties	Indication	Approval date	Review
	**2010** **New molecular entities = 14**					
1	Dalfampridine (Ampyra)	Acorda Therapeutics	Potassium channel blocker	Improving walking in patients with multiple sclerosis	01/22/2010	P, O
2	Liraglutide recombinant (Victoza)	Novo Nordisk	Glucagon-like peptide 1 (GLP1) receptor agonist	Type 2 diabetes	01/25/2010	P
3	Carglumic acid (Carbaglu)	Orphan Europe	Carbamoyl phosphate synthetase 1 (CPS1) activator	Acute hyperammonemia	03/18/2010	P, O
4	Polidocanol (Asclera)	Chemisch Fbrk Krsslr	Sclerosing agent	Uncomplicated spider veins and uncomplicated reticular veins	03/30/2010	S
5	Dienogest; Estradiol valerate (Natazia)	Bayer	Semisynthetic progestogen; Estrogen	Contraception	05/06/2010	S
6	Cabazitaxel (Jevtana kit)	Sanofi	Microtubule-stabilizing agent	Prostate cancer	06/17/2010	P
7	Alcaftadine (Lastacaft)	Allergan	Histamine H1 receptor antagonist	Allergic conjunctivitis	07/28/2010	S
8	Ulipristal acetate (Ella)	Lab HRA Pharma	Progesterone receptor modulator	Contraception	08/13/2010	S
9	Fingolimod HCl (Gilenya)	Novartis	Sphingosine 1-phosphate (S1P) receptor modulator	Multiple sclerosis	09/21/2010	P
10	Dabigatran etexilate mesylate (Pradaxa)	Boehringer Ingelheim	Direct thrombin inhibitor	Stroke prevention in atrial fibrillation	10/19/2010	P
11	Lurasidone HCl (Latuda)	Sunovion Pharms	Atypical antipsychotic agent, 5-$HT_{2A/7}$ and dopamine D2 receptors antagonist	Schizophrenia	10/28/2010	S
12	Ceftaroline fosamil (Teflaro)	Cerexa	Broad-spectrum cephalosporin antibiotic	Skin and skin-structure infections; Community acquired pneumonia	10/29/2010	S
13	Tesamorelin acetate (Egrifta)	Theratechnologies	Growth hormone-releasing factor analog	HIV lipodystrophy	11/10/2010	S
14	Eribulin mesylate (Halaven)	Eisai	Microtubule-destabilizing agent	Breast cancer	11/15/2010	P
	**2010** **Biologics license applications = 7**					
1	Tocilizumab (Actemra)	Genentech	Interleukin-6 (IL-6) receptor-directed mAb	Rheumatoid arthritis	01/08/2010	N/A
2	Collagenase clostridium histolyticum (Xiaflex)	Auxilium Pharms	Purified collagenase clostridium histolyticum	Dupuytren's contracture	02/02/2010	O
3	Velaglucerase alfa (Vpriv)	Shire	Recombinant human β-glucocerebrosidase	Gaucher's disease	02/26/2010	P
4	Alglucosidase alfa (Lumizyme)	Genzyme	Recombinant human acid α-glucosidase	Pompe's disease	05/24/2010	O
5	Denosumab (Prolia)	Amgen	RANK ligand (RANKL)-directed mAb	Postmenopausal osteoporosis	06/01/2010	O
6	IncobotulinumtoxinA (Xeomin)	Merz Pharms	Acetylcholine release inhibitor and neuromuscular blocking agent	Cervical dystonia and blepharospasm	07/30/2010	N/A
7	Pegloticase (Krystexxa)	Horizon Pharma	PEGylated uric acid specific enzyme	Gout	09/14/2010	O

Supplementary data: Table 1 – FDA-approved drugs (Continued)

	Drug (brand name)	Sponsor	Properties	Indication	Approval date	Review
	**2011** **New molecular entities = 24**					
1	Ioflupane I-123 (Datscan)	GE Healthcare	Dopamine transporter-binding imaging agent	Parkinsonian syndromes (Imaging agent)	01/14/2011	P
2	Spinosad (Natroba)	ParaPro	Causes neuronal hyperexcitation in insects	Head lice	01/18/2011	S
3	Vilazodone HCl (Viibryd)	Allergan	Selective serotonin reuptake inhibitor and 5-HT_{1A} partial agonist	Major depressive disorder	01/21/2011	S
4	Azilsartan kamedoxomil (Edarbi)	Arbor Pharms	Angiotensin II type 1 receptor antagonist	Hypertension	02/25/2011	S
5	Roflumilast (Daliresp)	Forest Laboratories	Phosphodiesterase 4 (PDE4) inhibitor	COPD exacerbations	02/28/2011	S
6	Gadobutrol (Gadavist)	Bayer	Gadolinium-based contrast agent	Blood-brain barrier imaging	03/14/2011	S
7	Gabapentin enacarbil (Horizant)	Arbor Pharms	Voltage-activated calcium channel inhibitor	Moderate-to-severe restless legs syndrome	04/06/2011	S
8	Vandetanib (Caprelsa)	Genzyme	Multi-target TKI (VEGFR2/3, EGFR, and RET)	Unresectable or metastatic medullary thyroid cancer	04/06/2011	P, O
9	Abiraterone acetate (Zytiga)	Janssen Biotech	CYP17A1 inhibitor	Metastatic castration-resistant prostate cancer	04/28/2011	P
10	Linagliptin (Tradjenta)	Boehringer Ingelheim	Dipeptidyl peptidase 4 inhibitor	Type 2 diabetes	05/02/2011	S
11	Boceprevir (Victrelis)	Merck & Co	NS3/4A protease inhibitor	HCV genotype 1	05/13/2011	P
12	Rilpivirine HCl (Edurant)	Janssen Prods	Non-nucleoside HIV reverse transcriptase inhibitor	HIV-1 infection	05/20/2011	S
13	Telaprevir (Incivek)	Vertex Pharms	NS3/4A protease inhibitor	HCV genotype 1	05/23/2011	P
14	Fidaxomicin (Dificid)	Cubist Pharms	RNA polymerase inhibitor	*Clostridium difficile*-associated diarrhea	05/27/2011	P
15	Ezogabine (Potiga)	GlaxoSmithKline	Potassium channel opener	Partial-onset seizures	06/10/2011	S
16	Indacaterol maleate (Arcapta neohaler)	Novartis	Long-acting β2-adrenergic receptor agonist	Chronic obstructive pulmonary disease (COPD)	07/01/2011	S
17	Rivaroxaban (Xarelto)	Janssen Pharms	Factor Xa inhibitor	Prophylaxis of deep vein thrombosis in hip and knee replacement surgery	07/01/2011	S
18	Ticagrelor (Brilinta)	AstraZeneca	$P2Y_{12}$ platelet inhibitor	Thrombotic cardiovascular events in patients with acute coronary syndrome	07/20/2011	S
19	Vemurafenib (Zelboraf)	Roche	BRAF inhibitor	BRAF-positive unresectable or metastatic melanoma	08/17/2011	P, O
20	Icatibant acetate (Firazyr)	Shire	Bradykinin B2 receptor antagonist	Hereditary angioedema	08/25/2011	P, O
21	Crizotinib (Xalkori)	Merck & Co	Multi-target TKI (ALK, ROS1, and MET)	ALK-positive advanced or metastatic NSCLC	08/26/2011	P, O
22	Deferiprone (Ferriprox)	Chiesi	Iron chelator	Transfusional iron overload due to thalassemia syndromes	10/14/2011	S, O
23	Clobazam (Onfi)	Lundbeck	Benzodiazepine, partial GABA-A receptors agonist	Seizures associated with Lennox-Gastaut syndrome	10/21/2011	S, O
24	Ruxolitinib phosphate (Jakafi)	Incyte Corp	JAK1/JAK2 inhibitor	Intermediate or high-risk myelofibrosis	11/16/2011	P, O
	**2011** **Biologics license applications = 6**					
1	Belimumab (Benlysta)	Human Genome Sciences	B-lymphocyte stimulator (BLyS)-directed mAb	Active, autoantibody-positive, systemic lupus erythematosus	03/09/2011	P
2	Ipilimumab (Yervoy)	Bristol-Myers Squibb	Cytotoxic T-lymphocyte antigen 4 (CTLA-4)-directed mAb	Unresectable or metastatic melanoma	03/25/2011	P, O
3	Belatacept (Nulojix)	Bristol-Myers Squibb	T cell co-stimulation blocker; soluble fusion protein consisting of extracellular domain of human CTLA-4 and modified Fc region of IgG	Organ rejection in kidney transplant	06/15/2011	S, O
4	Brentuximab vedotin (Adcetris)	Seattle Genetics	CD30-directed antibody drug conjugate (ADC)	Hodgkin's lymphoma; systemic anaplastic large cell lymphoma	08/19/2011	P, O
5	Aflibercept (Eylea)	Regeneron Pharmaceuticals	VEGF inhibitor; recombinant protein consisting of binding domains of VEGFR and Fc region of IgG	Neovascular (wet) age-related macular degeneration	11/18/2011	P
6	Asparaginase Erwinia chrysanthemi (Erwinaze)	EUSA Pharma (USA)	*Erwinia chrysanthemi*-derived L-asparaginase	Acute lymphoblastic leukemia	11/18/2011	P, O

Supplementary data: Table 1 – FDA-approved drugs (Continued)

	Drug (brand name)	Sponsor	Properties	Indication	Approval date	Review
	**2012** **New molecular entities = 32**					
1	Ingenol mebutate (Picato)	Leo Labs	MOA unknown	Actinic keratosis	01/23/2012	S
2	Axitinib (Inlyta)	Pfizer	VEGFRs inhibitor	Advanced renal cell carcinoma	01/27/2012	S
3	Vismodegib (Erivedge)	Genentech	Smoothened (Hedgehog pathway) inhibitor	Metastatic or locally advanced basal cell carcinoma	01/30/2012	P
4	Ivacaftor (Kalydeco)	Vertex Pharms	CFTR potentiator	Cystic fibrosis in patients with the G551D mutation in the *CFTR* gene	01/31/2012	P, O
5	Tafluprost (Zioptan)	Akorn	Selective prostaglandin F prostanoid receptor agonist	Elevated intraocular pressure in patients with open-angle glaucoma or ocular hypertension	02/10/2012	S
6	Lucinactant (Surfaxin)	Windtree Therap	Synthetic peptide-containing surfactant that mimics human surfactant protein-B	Prevention of respiratory distress syndrome	03/06/2012	S
7	Peginesatide acetate (Omontys)	Takeda	Synthetic, pegylated erythropoiesis stimulating agent	Anemia due to chronic kidney disease	03/27/2012	S
8	Florbetapir F-18 (Amyvid)	Avid Radiopharms	^{18}F labelled radioactive diagnostic agent	Diagnostic agent to estimate amyloid-β plaques in patients with suspected Alzheimer's disease	04/06/2012	S
9	Avanafil (Stendra)	Metuchen Pharms	Phosphodiesterase 5 (PDE5) inhibitor	Erectile dysfunction	04/27/2012	S
10	Lorcaserin (Belviq)	Eisai	5-HT$_{2C}$ receptor agonist	Obesity	06/27/2012	S
11	Mirabegron (Myrbetriq)	APGDI	β3-adrenergic receptor agonist	Overactive bladder	06/28/2012	S
12	Citric acid; Magnesium oxide; Sodium picosulfate (Prepopik)	Ferring Pharms	Osmotic and stimulant laxatives	Cleansing of the colon before colonoscopy	07/16/2012	S
13	Carfilzomib (Kyprolis)	Onyx Therap	20S proteasome inhibitor	Multiple myeloma	07/20/2012	S, O
14	Aclidinium bromide (Tudorza pressair)	AstraZeneca	Long-acting antimuscarinic agent	Bronchospasms associated with COPD	07/23/2012	S
15	Cobicistat; Elvitegravir; Emtricitabine; Tenofovir disoproxil fumarate (Stribild)	Gilead Sciences	Cytochrome P450 inhibitor; HIV-1 integrase inhibitor; NRTI; Acyclic nucleoside phosphonate analog	HIV-1 infection	08/27/2012	S
16	Linaclotide (Linzess)	Allergan	Guanylyl cyclase C agonist	Irritable bowel syndrome with constipation; Chronic idiopathic constipation	08/31/2012	S
17	Enzalutamide (Xtandi)	Astellas	Androgen receptor inhibitor	Metastatic castration-resistant prostate cancer	08/31/2012	P
18	Bosutinib monohydrate (Bosulif)	Pfizer	BCR-ABL inhibitor; SRC family kinase inhibitor	Chronic, accelerated or blast phase Ph+ chronic myelogenous leukemia	09/04/2012	S, O
19	Teriflunomide (Aubagio)	Sanofi	Dihydroorotate dehydrogenase inhibitor	Relapsing forms of multiple sclerosis	09/12/2012	S
20	Choline C-11	MCPRF	^{11}C labelled choline analog, radioactive diagnostic agent	Diagnostic agent for imaging of patients with suspected prostate cancer recurrence	09/12/2012	P
21	Regorafenib (Stivarga)	Bayer	Multi-target TKI (RET, VEGFRs, KIT, PDGFRα/β, FGFR1/2, RAF1, BRAF^{WT}, and BRAF^{V600E})	Metastatic colorectal cancer	09/27/2012	P
22	Perampanel (Fycompa)	Eisai	Non-competitive ionotropic AMPA glutamate receptor antagonist	Partial-onset seizures in patients with epilepsy	10/22/2012	S
23	Omacetaxine mepesuccinate (Synribo)	Teva	Cephalotaxine, inhibits protein chain elongation by binding the large ribosomal subunit	Chronic or accelerated-phase chronic myeloid leukemia	10/26/2012	S, O
24	Tofacitinib citrate (Xeljanz)	Pfizer	JAK inhibitor	Severely active rheumatoid arthritis	11/06/2012	S
25	Cabozantinib S-malate (Cometriq)	Exelixis	Multi-target TKI (VEGFRs, MET, RET, FLT3, KIT, TIE2, and AXL)	Progressive, metastatic medullary thyroid cancer	11/29/2012	P, O
26	Pasireotide diaspartate (Signifor)	Recordati Rare	Somatostatin analog	Cushing's disease	12/14/2012	P, O
27	Ponatinib HCl (Iclusig)	Takeda	Multi-kinase inhibitor (BCR-ABL, KIT, RET and FLT3)	Chronic, accelerated or blast-phase chronic myeloid leukemia	12/14/2012	P, O
28	Teduglutide recombinant (Gattex kit)	NPS Pharms	Glucagon-like peptide-2 (GLP-2) analog	Short bowel syndrome	12/21/2012	S, O
29	Lomitapide mesylate (Juxtapid)	Amryt	Microsomal triglyceride transfer protein inhibitor	Homozygous familial hypercholesterolemia	12/21/2012	S, O
30	Apixaban (Eliquis)	Bristol-Myers Squibb	Factor Xa inhibitor	Stroke and systemic embolism in patients with non-valvular atrial fibrillation	12/28/2012	P
31	Bedaquiline fumarate (Sirturo)	Janssen Therap	Diarylquinoline antimycobacterial	Pulmonary multidrug-resistant tuberculosis	12/28/2012	P, O
32	Crofelemer (Mytesi)	Napo Pharms	CFTR and CaCC channels inhibitor	HIV-associated diarrhea	12/31/2012	P
	**2012** **Biologics license applications = 7**					
1	Glucarpidase (Voraxaze)	BTG International	Recombinant carboxypeptidase G2	Toxic plasma methotrexate concentrations in patients with delayed methotrexate clearance	01/17/2012	P, O
2	Taliglucerase alfa (Elelyso)	Pfizer	Recombinant human lysosomal enzyme β-glucocerebrosidase, produced in carrot cells	Gaucher's disease	05/01/2012	S, O
3	Pertuzumab (Perjeta)	Genentech	HER2-directed mAb	HER2-positive metastatic breast cancer	06/08/2012	P
4	Ziv-aflibercept (Zaltrap)	Sanofi	Soluble receptor decoy that binds VEGF-A, VEGF-B, and PlGF	Metastatic colorectal cancer	08/03/2012	P

Supplementary data: Table 1 – FDA-approved drugs (Continued)

	Drug (brand name)	Sponsor	Properties	Indication	Approval date	Review
	**2012**	**Biologics license applications = 7**				
5	Tbo-Filgrastim (Neutroval)	Sicor Biotech	Methionyl human G-CSF	Severe neutropaenia in patients with non-myeloid malignancies	08/29/2012	S
6	Ocriplasmin (Jetrea)	Thrombogenics	Recombinant truncated form of human plasmin	Symptomatic vitreomacular adhesion	10/17/2012	P
7	Raxibacumab	Human Genome Sciences	Human mAb against *Bacillus anthracis* toxin	Anthrax	12/14/2012	P, O

Supplementary data: Table 1 – FDA-approved drugs (Continued)

	Drug (brand name)	Sponsor	Properties	Indication	Approval date	Review
	**2013** **New molecular entities = 25**					
1	Alogliptin benzoate (Nesina)	Takeda	DPP4 inhibitor	Type 2 diabetes	01/25/2013	S
2	Mipomersen sodium (Kynamro)	Kastle Theraps	Oligonucleotide inhibitor of apolipoprotein B100 synthesis	Homozygous familial hypercholesterolemia	01/29/2013	S, O
3	Pomalidomide (Pomalyst)	Celgene	Thalidomide analog, immunomodulatory antineoplastic agent	Multiple myeloma	02/08/2013	S, O
4	Ospemifene (Osphena)	Duchesnay	Estrogen receptor modulator	Moderate to severe dyspareunia due to menopause	02/26/2013	S
5	Technetium TC 99m tilmanocept (Lymphoseek kit)	Cardinal Health 414	^{99m}Tc labelled radioactive diagnostic agent	Lymphatic mapping in patients with breast cancer or melanoma	03/13/2013	S
6	Gadoterate meglumine (Dotarem)	Guerbet	Gadolinium-based contrast agent	Contrast agent to visualize disruption of the blood-brain barrier	03/20/2013	P
7	Dimethyl fumarate (Tecfidera)	Biogen	MOA unknown; activates NRF2 pathway	Relapsing forms of multiple sclerosis	03/27/2013	S
8	Canagliflozin (Invokana)	Janssen Pharms	Sodium-dependent glucose cotransporter 2 (SGLT2) inhibitor	Type 2 diabetes	03/29/2013	S
9	Fluticasone furoate; Vilanterol trifenatate (Breo ellipta)	GlaxoSmithKline	Corticosteroid plus long-acting β2-adrenergic agonist	Chronic obstructive pulmonary disease	05/10/2013	S
10	Radium Ra-223 dichloride (Xofigo)	Bayer	α-particle-emitting radioactive therapeutic	Castration-resistant prostate cancer	05/15/2013	P
11	Dabrafenib mesylate (Tafinlar)	Novartis	Kinase inhibitor with activity against BRAF$^{V600E/D/K}$, wild-type BRAF and other kinases	Unresectable or metastatic melanoma with *BRAF*^{V600E} mutation	05/29/2013	S, O
12	Trametinib dimethyl sulfoxide (Mekinist)	Novartis	MEK1 and MEK2 kinase inhibitor	Unresectable or metastatic melanoma with *BRAF*^{V600E} mutation	05/29/2013	S, O
13	Afatinib dimaleate (Gilotrif)	Boehringer Ingelheim	EGFR, HER2, and HER4 inhibitor	Metastatic NSCLC with *EGFR* exon 19 deletions or exon 21 (L858R) substitution mutations	07/12/2013	P, O
14	Dolutegravir sodium (Tivicay)	Viiv Hlthcare	HIV-1 integrase strand transfer inhibitor	HIV-1 infection in adults and children, in combination with other antiretroviral agents	08/12/2013	P
15	Vortioxetine HBr (Brintellix)	Takeda	Serotonin reuptake inhibitor	Major depressive disorder	09/30/2013	S
16	Bazedoxifene acetate; Estrogens, conjugated (Duavee)	Wyeth Pharms	Conjugated estrogens with an estrogen receptor modulator	Moderate to severe vasomotor symptoms associated with menopause and prevention of postmenopausal osteoporosis	10/03/2013	S
17	Riociguat (Adempas)	Bayer	Soluble guanylyl cyclase stimulator	Persistent or recurrent chronic thromboembolic pulmonary hypertension and pulmonary arterial hypertension	10/08/2013	P, O
18	Macitentan (Opsumit)	Actelion	Endothelin receptor antagonist	Pulmonary arterial hypertension	10/18/2013	S, O
19	Flutemetamol F-18 (Vizamyl)	GE Healthcare	^{18}F labelled Radioactive diagnostic agent	PET imaging of the brain to estimate β-amyloid neuritic plaque density in patients who are being evaluated for Alzheimer's disease	10/25/2013	S
20	Eslicarbazepine acetate (Aptiom)	Sunovion Pharms	MOA unknown, but thought to involve voltage-gated sodium channels	Partial-onset seizures	11/08/2013	S
21	Ibrutinib (Imbruvica)	Pharmacyclics	Bruton's tyrosine kinase inhibitor	Mantle cell lymphoma	11/13/2013	P, O
22	Luliconazole (Luzu)	Bausch	Azole antifungal, MOA unknown, but thought to inhibit the enzyme lanosterol demethylase	Interdigital tinea pedis, tinea cruris and tineacorporis caused by *Trichophyton rubrum* and *Epidermophyton floccosum*	11/14/2013	S
23	Simeprevir sodium (Olysio)	Janssen Prods	HCV NS3/4A protease inhibitor	Chronic HCV infection, as a component of a combination antiviral treatment regimen	11/22/2013	P
24	Sofosbuvir (Sovaldi)	Gilead Sciences	HCV nucleotide analogue NS5B polymerase inhibitor	Chronic HCV infection, as a component of a combination antiviral treatment regimen	12/06/2013	P
25	Umeclidinium bromide; Vilanterol trifenatate (Anoro ellipta)	GlaxoSmithKline	Anticholinergic plus long-acting β2-adrenergic agonist	Chronic obstructive pulmonary disease	12/18/2013	S
	**2013** **Biologics license applications = 2**					
1	Ado-trastuzumab emtansine (Kadcyla)	Genentech	HER2-directed ADC	HER2-positive metastatic breast cancer	02/22/2013	P
2	Obinutuzumab (Gazyva)	Genentech	CD20-directed mAb	Previously untreated chronic lymphocytic leukemia	11/01/2013	P, O

Supplementary data: Table 1 – FDA-approved drugs (Continued)

	Drug (brand name)	Sponsor	Properties	Indication	Approval date	Review
	**2014** **New molecular entities = 30**					
1	Dapagliflozin (Farxiga)	AstraZeneca	Sodium-dependent glucose cotransporter 2 (SGLT2) inhibitor	Type 2 diabetes	01/08/2014	S
2	Tasimelteon (Hetlioz)	Vanda Pharms	Melatonin-receptor agonist	Non-24-hour sleep-wake disorder	01/31/2014	P, O
3	Droxidopa (Northera)	Lundbeck	Synthetic amino-acid precursor of noradrenaline; α-adrenergic receptors agonist	Light-headedness in patients with neurogenic orthostatic hypotension	02/18/2014	P, O
4	Florbetaben F-18 (Neuraceq)	Life Molecular	^{18}F labelled radioactive diagnostic agent	Imaging of the brain to estimate density of neuritic amyloid-β plaques	03/19/2014	S
5	Miltefosine (Impavido)	Knight Theraps	Antileishmanial agent, MOA unknown	Leishmaniasis	03/19/2014	P, O
6	Apremilast (Otezla)	Amgen	Phosphodiesterase 4 (PDE4) inhibitor	Psoriatic arthritis and plaque psoriasis	03/21/2014	S
7	Ceritinib (Zykadia)	Novartis	Multi-target TKI (ALK, IGF1R, INSR, and ROS1)	ALK-positive metastatic NSCLC	04/29/2014	P, O
8	Vorapaxar sulfate (Zontivity)	Xspire Pharma	Protease-activated receptor 1 antagonist	Thrombotic cardiovascular events	05/08/2014	S
9	Dalbavancin HCl (Dalvance)	Allergan	Semi-synthetic lipoglycopeptide, bacterial cell wall biosynthesis inhibitor	Acute bacterial skin and skin structure infections (ABSSSI)	05/23/2014	P
10	Efinaconazole (Jublia)	Bausch	Azole antifungal, fungal lanosterol 14α-demethylase inhibitor	Onychomycosis of the toenails	06/06/2014	S
11	Tedizolid phosphate (Sivextro)	Cubist	Oxazolidinone-class antibacterial, bacterial protein synthesis inhibitor	Acute bacterial skin and skin structure infections (ABSSSI)	06/20/2014	P
12	Belinostat (Beleodaq)	Acrotech	Histone-deacetylase inhibitor	Peripheral T-cell lymphoma	07/03/2014	P, O
13	Tavaborole (Kerydin)	Anacor Pharms	Oxaborole antifungal, cellular protein synthesis inhibitor	Onychomycosis of the toenails	07/07/2014	S
14	Idelalisib (Zydelig)	Gilead Sciences	PI3Kδ inhibitor	Chronic lymphocytic leukaemia (CLL), B-cell non-Hodgkin's lymphoma, and small lymphocytic lymphoma (SLL)	07/23/2014	P, O
15	Olodaterol HCl (Striverdi respimat)	Boehringer Ingelheim	Long-acting β2-adrenoceptor agonist	Chronic obstructive pulmonary disease (COPD)	07/31/2014	S
16	Empagliflozin (Jardiance)	Boehringer Ingelheim	Sodium-dependent glucose cotransporter 2 (SGLT2) inhibitor	Type 2 diabetes	08/01/2014	S
17	Oritavancin diphosphate (Orbactiv)	Melinta Therap	Semi-synthetic lipoglycopeptide, bacterial cell wall biosynthesis inhibitor	Acute bacterial skin and skin structure infections (ABSSSI)	08/06/2014	P
18	Suvorexant (Belsomra)	Merck & Co	Orexin-receptor antagonist	Insomnia	08/13/2014	S
19	Eliglustat tartrate (Cerdelga)	Genzyme	Glucosylceramide-synthase inhibitor	Gaucher's disease	08/19/2014	P, O
20	Naloxegol oxalate (Movantik)	Redhill	Opioid-receptor antagonist	Opioid-induced constipation	09/16/2014	S
21	Sulfur hexafluoride lipid-type A microspheres (Lumason)	Bracco	Ultrasound contrast agent	Cardiovascular imaging	10/10/2014	S
22	Netupitant; Palonosetron HCl (Akynzeo)	Helsinn Hlthcare	NK1-receptor antagonist plus 5-HT_3 receptor antagonist	Chemotherapy-related nausea	10/10/2014	S
23	Ledipasvir; Sofosbuvir (Harvoni)	Gilead Sciences	NS5A inhibitor plus HCV nucleotide-analog NS5B-polymerase inhibitor	Genotype 1 HCV	10/10/2014	P
24	Pirfenidone (Esbriet)	Genentech	MOA unknown	Idiopathic pulmonary fibrosis (IPF)	10/15/2014	P, O
25	Nintedanib esylate (Ofev)	Boehringer Ingelheim	Multi-kinase inhibitor (PDGFRs, FGFRs, VEGFRs, and FLT3)	Idiopathic pulmonary fibrosis (IPF)	10/15/2014	P, O
26	Finafloxacin (Xtoro)	Merlion Pharms	Fluoroquinolone antimicrobial	Acute otitis externa (Swimmer's ear)	12/17/2014	P
27	Olaparib (Lynparza)	AstraZeneca	PARP inhibitor	Advanced *BRCA*-mutated ovarian cancer	12/19/2014	P, O
28	Peramivir (Rapivab)	BioCryst	Neuraminidase inhibitor	Influenza infection	12/19/2014	S
29	Dasabuvir sodium; Ombitasvir; Paritaprevir; Ritonavir (Viekira pak ([Copackaged])	AbbVie	NS5A inhibitor plus NS3A/4A-protease inhibitor plus non-nucleoside NS5B palm-polymerase inhibitor plus CYP3A inhibitor	Chronic HCV genotype 1 infection	12/19/2014	P
30	Ceftolozane sulfate; Tazobactam sodium (Zerbaxa)	Cubist Pharms	Cephalosporin antibacterial plus β-lactamase inhibitor	Complicated intra-abdominal infections and complicated urinary tract infections	12/19/2014	P
	**2014** **Biologics license applications = 11**					
1	Elosulfase alfa (Vimizim)	BioMarin Pharm	Recombinant human lysosomal glycosaminoglycan-specific enzyme	Mucopolysaccharidosis type IV A	02/14/2014	P, O
2	Metreleptin (Myalept)	Aegerion	Recombinant analog of the human hormone leptin	Leptin deficiency in patients with congenital or acquired generalized lipodystrophy	02/24/2014	P, O
3	Albiglutide (Tanzeum)	GlaxoSmithKline	Glucagon-like peptide 1 (GLP1) receptor agonist	Type 2 diabetes	04/15/2014	S
4	Ramucirumab (Cyramza)	Eli Lilly & Co	VEGFR2-directed mAb	Gastric cancer	04/21/2014	P, O
5	Siltuximab (Sylvant)	Janssen Biotech	Interleukin-6 (IL-6)-directed mAb	Multicentric Castleman's disease	04/23/2014	P, O
6	Vedolizumab (Entyvio)	Takeda	Integrin receptor antagonist	Ulcerative colitis and Crohn's disease	05/20/2014	P
7	Peginterferon β-1a (Plegridy)	Biogen Idec	Long-acting interferon β-1a	Relapsing multiple sclerosis	08/15/2014	S

Supplementary data: Table 1 – FDA-approved drugs (Continued)

	Drug (brand name)	Sponsor	Properties	Indication	Approval date	Review
	**2014** **Biologics license applications = 11**					
8	Pembrolizumab (Keytruda)	Merck & Co	PD1-directed mAb	Metastatic melanoma	09/04/2014	P, O
9	Dulaglutide (Trulicity)	Eli Lilly & Co	GLP1-receptor agonist, recombinant dimeric fusion protein consisting of GLP1 peptide and Fc portion of human IgG4	Type 2 diabetes	09/18/2014	S
10	Blinatumomab (Blincyto)	Amgen	CD19- and CD3-bispecific mAb	B-cell precursor acute lymphoblastic leukemia (ALL)	12/03/2014	P, O
11	Nivolumab (Opdivo)	Bristol-Myers Squibb	PD1-directed mAb	Unresectable or metastatic melanoma	12/22/2014	P, O

Supplementary data: Table 1 – FDA-approved drugs (Continued)

	Drug (brand name)	Sponsor	Properties	Indication	Approval date	Review
	**2015** **New molecular entities = 32**					
1	Edoxaban tosylate (Savaysa)	Daiichi Sankyo	Factor Xa inhibitor	Risk of stroke and systemic embolism in NVAF, and deep vein thrombosis	01/08/2015	S
2	Palbociclib (Ibrance)	Pfizer	Cyclin-dependent kinase 4/6 (CDK4/6) inhibitor	HER2-negative and HR-positive advanced or metastatic breast cancer	02/03/2015	P
3	Lenvatinib mesylate (Lenvima)	Eisai	Multi-target TKI (VEGFRs, FGFRs, PDGFRα, RET, and KIT)	Thyroid cancer	02/13/2015	P, O
4	Panobinostat lactate (Farydak)	Secura	Histone deacetylase inhibitor	Multiple myeloma	02/23/2015	P, O
5	Avibactam sodium; Ceftazidime (Avycaz)	Allergan	β-lactamase inhibitor plus cephalosporin	Complicated intra-abdominal infections and complicated urinary tract infections	02/25/2015	P
6	Isavuconazonium sulfate (Cresemba)	Astellas	Azole antifungal, sterol 14-α-demethylase inhibitor	Invasive aspergillosis and mucormycosis	03/06/2015	P, O
7	Cholic acid (Cholbam)	Travere	Primary bile acid	Bile acid synthesis disorders and peroxisomal disorders	03/17/2015	P, O
8	Ivabradine HCl (Corlanor)	Amgen	Hyperpolarization-activated cyclic nucleotide-gated channel blocker	Chronic heart failure	04/15/2015	P
9	Deoxycholic acid (Kybella)	Kythera Biopharms	Cytolytic drug	Fat below the chin	04/29/2015	S
10	Eluxadoline (Viberzi)	Allergan Holdings	μ-opioid receptor agonist	Irritable bowel syndrome	05/27/2015	P
11	Cangrelor (Kengreal)	Chiesi	P2Y12 platelet inhibitor	Myocardial infarction, repeat coronary revascularization, and stent thrombosis	06/22/2015	S
12	Ivacaftor; Lumacaftor (Orkambi)	Vertex Pharms	CFTR potentiator plus CFTR corrector	Cystic fibrosis in patients with homozygous ΔF508 CFTR mutation	07/02/2015	P, O
13	Sacubitril; Valsartan (Entresto)	Novartis	Neprilysin inhibitor plus angiotensin II receptor blocker	Chronic heart failure	07/07/2015	P
14	Brexpiprazole (Rexulti)	Otsuka	Atypical antipsychotic, 5-HT$_{1A}$ and dopamine D2 receptors agonist, and 5-HT$_{2A}$ receptor antagonist	Major depressive disorder (MDD) and schizophrenia	07/10/2015	S
15	Sonidegib phosphate (Odomzo)	Sun Pharma Global	Smoothened inhibitor	Basal cell carcinoma	07/24/2015	S
16	Daclatasvir 2HCl (Daklinza)	Bristol-Myers Squibb	NS5A inhibitor	HCV infection	07/24/2015	P
17	Flibanserin (Addyi)	Sprout Pharms	Serotonin receptor modulator	Hypoactive sexual desire disorder	08/18/2015	S
18	Rolapitant HCl (Varubi)	Tersera	Neurokinin 1 receptor antagonist	Nausea and vomiting associated with chemotherapy	09/01/2015	S
19	Uridine triacetate (Xuriden)	Wellstat Therap	Pyrimidine analog, synthetic uridine prodrug	Hereditary orotic aciduria	09/04/2015	P, O
20	Cariprazine HCl (Vraylar)	Allergan	Atypical antipsychotic, 5-HT$_{1A}$ and dopamine D2 receptors agonist, and 5-HT$_{2A}$ receptor antagonist	Schizophrenia and bipolar 1 disorder	09/17/2015	S
21	Tipiracil HCl; Trifluridine (Lonsurf)	Taiho Oncology	Thymidine phosphorylase inhibitor plus a nucleoside metabolic inhibitor	Colorectal cancer	09/22/2015	S
22	Aripiprazole lauroxil (Aristada)	Alkermes	Atypical antipsychotic, 5-HT$_{1A}$ and dopamine D2 receptors agonist, and 5-HT$_{2A}$ receptor antagonist	Schizophrenia	10/05/2015	S
23	Patiromer sorbitex calcium (Veltassa)	Vifor Pharma	Potassium binder	Hyperkalemia	10/21/2015	S
24	Trabectedin (Yondelis)	Janssen Prods	Alkylating drug, interacts with the minor groove of DNA	Liposarcoma or leiomyosarcoma	10/23/2015	P, O
25	Cobicistat; Elvitegravir; Emtricitabine; Tenofovir alafenamide fumarate (Genvoya)	Gilead Sciences	INSTI plus CYP3A inhibitor plus two nucleoside analog reverse transcriptase inhibitors	HIV-1 infection	11/05/2015	S
26	Cobimetinib fumarate (Cotellic)	Genentech	MEK inhibitor	Melanoma with *BRAF*$^{V600E/K}$ mutations	11/10/2015	P, O
27	Osimertinib mesylate (Tagrisso)	AstraZeneca	EGFR inhibitor	NSCLC with *EGFR*^{T790M} mutations	11/13/2015	P, O
28	Ixazomib citrate (Ninlaro)	Takeda	Oral proteasome inhibitor	Multiple myeloma	11/20/2015	P, O
29	Alectinib HCl (Alecensa)	Roche	ALK inhibitor	Non-small-cell lung cancer (NSCLC)	12/11/2015	P, O
30	Sugammadex sodium (Bridion)	Merck & Co	Modified γ-cyclodextrin	Reversal of neuromuscular blockade during surgery	12/15/2015	P
31	Selexipag (Uptravi)	Actelion	Prostacyclin receptor agonist	Pulmonary arterial hypertension	12/21/2015	S, O
32	Lesinurad (Zurampic)	Ironwood Pharms	Uric acid transporter 1 (URAT1) inhibitor	Gout	12/22/2015	S
	**2015** **Biologics license applications = 13**					
1	Secukinumab (Cosentyx)	Novartis	Interleukin-17A (IL-17A)-directed mAb	Plaque psoriasis	01/21/2015	S
2	Parathyroid hormone (Natpara)	NPS Pharma	Human parathyroid hormone (PTH) analog	Hypocalcemia in patients with hypoparathyroidism	01/23/2015	S, O
3	Dinutuximab (Unituxin)	United Therap	GD2-directed mAb	High-risk neuroblastoma	03/10/2015	P, O
4	Alirocumab (Praluent)	Sanofi	Proprotein convertase subtilisin kexin type 9 (PCSK9) inhibitor	Heterozygous familial hypercholesterolemia	07/24/2015	P
5	Evolocumab (Repatha)	Amgen	PCSK9 inhibitor	Primary hyperlipidemia	08/27/2015	S, O
6	Insulin degludec (Tresiba)	Novo Nordisk	Recombinant human insulin [Lys (B28), Pro (B29)]	Type 1 & 2 diabetes	09/25/2015	S

Supplementary data: Table 1 – FDA-approved drugs (Continued)

	Drug (brand name)	Sponsor	Properties	Indication	Approval date	Review
	**2015** **Biologics license applications = 13**					
7	Idarucizumab (Praxbind)	Boehringer Ingelheim	Humanized anti-dabigatran IgG1κ Fab	Reversal of anticoagulant effects of dabigatran	10/16/2015	P, O
8	Asfotase alfa (Strensiq)	Strensiq	Human recombinant tissue-nonspecific alkaline phosphatase-Fc-deca-aspartate fusion protein	Hypophosphatasia	10/23/2015	P, O
9	Mepolizumab (Nucala)	GlaxoSmithKline	Interleukin-5 (IL-5)-directed mAb	Severe asthma	11/04/2015	S
10	Daratumumab (Darzalex)	Janssen Biotech	CD38-directed mAb	Multiple myeloma	11/16/2015	P, O
11	Necitumumab (Portrazza)	Eli Lilly & Co	EGFR-directed mAb	Non-small-cell lung cancer (NSCLC)	11/24/2015	S, O
12	Elotuzumab (Empliciti)	Bristol-Myers Squibb	SLAMF7-directed mAb	Multiple myeloma	11/30/2015	P, O
13	Sebelipase alfa (Kanuma)	Alexion Pharm	Recombinant human lysosomal acid lipase	Lysosomal acid lipase deficiency	12/08/2015	P, O

Supplementary data: Table 1 – FDA-approved drugs (Continued)

	Drug (brand name)	Sponsor	Properties	Indication	Approval date	Review
	**2016** **New molecular entities = 15**					
1	Elbasvir; Grazoprevir (Zepatier)	Merck & Co	NS5A inhibitor plus NS3/4A protease inhibitor	HCV genotypes 1 and 4	01/28/2016	P
2	Brivaracetam (Briviact)	UCB	Anticonvulsant with unknown MOA, binds to SV2A	Partial-onset seizures	02/18/2016	S
3	Defibrotide sodium (Defitelio)	Jazz	Profibrinolytic	Hepatic veno-occlusive disease	03/30/2016	P, O
4	Venetoclax (Venclexta)	AbbVie	BCL-2 inhibitor	Chronic lymphocytic leukemia	04/11/2016	P, O
5	Pimavanserin tartrate (Nuplazid)	Acadia Pharmas	Atypical antipsychotic, $5\text{-}HT_{2A}$ receptor antagonist	Hallucinations and delusions associated with Parkinson's disease psychosis	04/29/2016	P
6	Obeticholic acid (Ocaliva)	Intercept Pharms	Bile acid analog and farnesoid X receptor agonist	Primary biliary cholangitis	05/27/2016	P, O
7	Fluciclovine F-18 (Axumin)	Blue Earth	^{18}F labelled radioactive diagnostic agent	Imaging diagnosis of suspected prostate cancer recurrence	05/27/2016	P
8	Gallium dotatate Ga-68 (Netspot)	AAA USA Inc	^{68}Ga labelled radioactive diagnostic	Imaging diagnosis of neuroendocrine tumors	06/01/2016	P, O
9	Sofosbuvir; Velpatasvir (Epclusa)	Gilead Sciences	Nucleotide analog NS5B polymerase inhibitor plus HCV NS5A inhibitor	HCV genotypes 1-6	06/28/2016	P
10	Lifitegrast (Xiidra)	Novartis	Tetrahydroisoquinoline derivative and lymphocyte function-associated antigen-1 (LFA1) antagonist	Dry eye disease	07/11/2016	P
11	Lixisenatide (Adlyxin)	Sanofi	Glucagon-like peptide-1 (GLP-1) receptor agonist	Type 2 diabetes	07/27/2016	S
12	Eteplirsen (Exondys 51)	Sarepta Theraps	Antisense oligonucleotide designed to target dystrophin pre-mRNA	Duchenne muscular dystrophy	09/19/2016	P, O
13	Crisaborole (Eucrisa)	Anacor Pharms	PDE4 inhibitor	Atopic dermatitis	12/14/2016	S
14	Rucaparib camsylate (Rubraca)	Clovis Oncology	PARP inhibitor	BRCA-positive ovarian cancer	12/19/2016	P, O
15	Nusinersen sodium (Spinraza)	Biogen Idec	Antisense oligonucleotide designed to target SMN2 pre-mRNA	Spinal muscular atrophy	12/23/2016	P, O
	**2016** **Biologics license applications = 7**					
1	Obiltoxaximab (Anthim)	Elusys Therapeutics	Anthrax toxin-directed mAb	Anthrax	03/18/2016	S, O
2	Ixekizumab (Taltz)	Eli Lilly & Co	Interleukin-17A (IL-17A)-directed mAb	Plaque psoriasis	03/22/2016	S
3	Reslizumab (Cinqair)	Teva	Interleukin-5 (IL-5)-directed mAb	Severe asthma	03/23/2016	S
4	Atezolizumab (Tecentriq)	Genentech	PDL1-directed mAb	Advanced or metastatic urothelial carcinoma	05/18/2016	P
5	Daclizumab (Zinbryta)	Biogen	Interleukin-2 (IL-2)-directed mAb	Multiple sclerosis	05/27/2016	S
6	Olaratumab (Lartruvo)	Eli Lilly & Co	PDGFRα-directed mAb	Soft tissue sarcoma	10/19/2016	P, O
7	Bezlotoxumab (Zinplava)	Merck & Co	*Clostridium difficile* toxin-directed mAb	*C. difficile* recurrence	10/21/2016	P

Supplementary data: Table 1 – FDA-approved drugs (Continued)

	Drug (brand name)	Sponsor	Properties	Indication	Approval date	Review
**2017**	**New molecular entities = 34**					
1	Plecanatide (Trulance)	Salix	Guanylate cyclase C agonist	Chronic idiopathic constipation	01/19/2017	S
2	Etelcalcetide (Parsabiv)	Kai Pharms Inc	Calcium-sensing receptor agonist	Secondary hyperparathyroidism in patients with chronic kidney disease on hemodialysis	02/07/2017	S
3	Deflazacort (Emflaza)	PTC Therap	Corticosteroid	Duchenne muscular dystrophy	02/09/2017	P, O
4	Telotristat etiprate (Xermelo)	Tersera	Tryptophan hydroxylase inhibitor	Carcinoid syndrome diarrhea	02/28/2017	P, O
5	Ribociclib succinate (Kisqali)	Novartis	CDK4/6 inhibitor	HR-positive, HER2-negative breast cancer	03/13/2017	P
6	Safinamide mesylate (Xadago)	MDD US	MAO-B inhibitor	Parkinson's disease	03/21/2017	S
7	Naldemedine tosylate (Symproic)	BDSI	Opioid antagonist	Opioid-induced constipation	03/23/2017	S
8	Niraparib tosylate (Zejula)	GlaxoSmithKline	Poly-ADP ribose polymerase (PARP) inhibitor	Epithelial ovarian, fallopian tube or primary peritoneal cancer	03/27/2017	P, O
9	Deutetrabenazine (Austedo)	Teva	VMAT2 inhibitor	Chorea associated with Huntington disease	04/03/2017	S, O
10	Valbenazine tosylate (Ingrezza)	Neurocrine	VMAT2 inhibitor	Tardive dyskinesia	04/11/2017	P
11	Abaloparatide (Tymlos)	Radius Health	Parathyroid hormone-related protein	Osteoporosis	04/28/2017	S
12	Brigatinib (Alunbrig)	Takeda	Multi-target TKI (ALK, EGFR, IGF1R, FLT3, and ROS1)	ALK-positive NSCLC	04/28/2017	P, O
13	Midostaurin (Rydapt)	Novartis	Fms-like tyrosine kinase 3 (FLT3) inhibitor	FLT3-positive acute myeloid leukemia (AML)	04/28/2017	P, O
14	Edaravone (Radicava)	Mitsubishi Tanabe	MOA unknown (Free radical scavenger)	Amyotrophic lateral scleorosis (ALS)	05/05/2017	S, O
15	Delafloxacin meglumine (Baxdela)	Melinta	Fluoroquinolone antibacterial, bacterial DNA gyrase (topoisomerase II) and DNA topoisomerase IV inhibitor	Acute bacterial skin and skin structure infections	06/19/2017	P
16	Betrixaban (Bevyxxa)	Portola Pharms	FXa inhibitor	Prophylaxis of venous thromboembolism	06/23/2017	P
17	Neratinib maleate (Nerlynx)	Puma Biotech	EGFR, HER2, and HER4 inhibitor	HER2-overexpressed breast cancer	07/17/2017	S
18	Sofosbuvir; Velpatasvir; Voxilaprevir (Vosevi)	Gilead Sciences	Nucleotide analog NS5B polymerase inhibitor plus NS5A inhibitor plus NS3/4A protease inhibitor	HCV	07/18/2017	P
19	Enasidenib mesylate (Idhifa)	Celgene Corp	IDH2 inhibitor	*IDH2*-mutated acute myeloid leukemia (AML)	08/01/2017	P, O
20	Glecaprevir; Pibrentasvir (Mavyret)	AbbVie	NS3/4A protease inhibitor plus NS5A inhibitor	HCV	08/03/2017	P
21	Benznidazole	Chemo Research	Nitroimidazole antimicrobial	Chagas disease	08/29/2017	P, O
22	Meropenem; Vaborbactam (Vabomere)	Rempex	Carbapenem antimicrobial plus a β-lactamase inhibitor	Complicated urinary tract infections	08/29/2017	P
23	Copanlisib 2HCl (Aliqopa)	Bayer	PI3Kα/δ inhibitor	Follicular lymphoma (FL)	09/14/2017	P, O
24	Secnidazole (Solosec)	Lupin	Nitroimidazole antimicrobial	Bacterial vaginosis	09/15/2017	P
25	Abemaciclib (Verzenio)	Eli Lilly & Co	CDK4/6 inhibitor	HR-positive, HER2-negative breast cancer	09/28/2017	P
26	Acalabrutinib (Calquence)	AstraZeneca	BTK inhibitor	Mantle cell lymphoma	10/31/2017	P, O
27	Latanoprostene bunod (Vyzulta)	Bausch & Lomb	Prostaglandin analog	Intraocular pressure	11/02/2017	S
28	Letermovir (Prevymis)	Merck & Co	CMV DNA terminase complex inhibitor	Prophylaxis of CMV	11/08/2017	P, O
29	Semaglutide (Ozempic)	Novo Nordisk	GLP1 receptor agonist	Type 2 diabetes	12/05/2017	S
30	Ozenoxacin (Xepi)	Ferrer Internacional	Quinolone antimicrobial	Impetigo due to *Staphylococcus aureus* or *Streptococcus pyogenes*	12/11/2017	S
31	Netarsudil mesylate (Rhopressa)	Aerie Pharms	RHO kinase inhibitor	Open-angle glaucoma or ocular hypertension	12/18/2017	P
32	Ertugliflozin (Steglatro)	Merck & Co	Sodium-dependent glucose cotransporter 2 (SGLT2) inhibitor	Type 2 diabetes	12/19/2017	S
33	Macimorelin acetate (Macrilen)	Novo Nordisk	Growth hormone secretagogue receptor agonist	Diagnosis of adult growth hormone deficiency	12/20/2017	S, O
34	Angiotensin II acetate (Giapreza)	La Jolla Pharma	Synthetic human angiotensin	Blood pressure in adults with septic or other distributive shock	12/21/2017	P
**2017**	**Biologics license applications = 12**					
1	Brodalumab (Siliq)	Valeant Luxembourg	IL-17RA-directed mAb	Plaque psoriasis	02/15/2017	S
2	Avelumab (Bavencio)	Emd Serono	PDL1-directed mAb	Merkel cell carcinoma	03/23/2017	P, O
3	Ocrelizumab (Ocrevus)	Genentech	CD20-directed mAb	Relapsing or primary progressive forms of multiple sclerosis	03/23/2017	P
4	Dupilumab (Dupixent)	Regeneron	Interleukin-4 (IL-4) receptor alpha (IL-4Rα)-directed mAb	Atopic dermatitis	03/28/2017	P
5	Cerliponase alfa (Brineura)	BioMarin Pharm	Tripeptidyl peptidase	Tripeptidyl peptidase 1 deficiency	04/27/2017	P, O
6	Durvalumab (Imfinzi)	AstraZeneca	PDL1-directed mAb	Urothelial carcinoma	05/01/2017	P
7	Sarilumab (Kevzara)	Sanofi	Interleukin 6 (IL-6) receptor-directed mAb	Rheumatoid arthritis	05/22/2017	S

Supplementary data: Table 1 – FDA-approved drugs (Continued)

	Drug (brand name)	Sponsor	Properties	Indication	Approval date	Review
	**2017** **Biologics license applications = 12**					
8	Guselkumab (Tremfya)	Janssen Biotech	Interleukin 23 (IL-23)-directed mAb	Plaque psoriasis	07/13/2017	P, O
9	Inotuzumab ozogamicin (Besponsa)	Wyeth Pharms	CD22-directed ADC	B cell precursor acute lymphoblastic leukemia (ALL)	08/17/2017	P, O
10	Benralizumab (Fasenra)	AstraZeneca	Interleukin-5 (IL-5) receptor alpha (IL-5Rα)-directed mAb	Severe asthma	11/14/2017	S
11	Vestronidase alfa (Mepsevii)	Ultragenyx Pharm Inc	Recombinant human lysosomal β-glucuronidase	Mucopolysaccharidosis VII	11/15/2017	P, O
12	Emicizumab (Hemlibra)	Genentech	Bispecific FIX and FX-directed antibody	Hemophilia A	11/16/2017	P, O

Supplementary data: Table 1 – FDA-approved drugs (Continued)

	Drug (brand name)	Sponsor	Properties	Indication	Approval date	Review
**2018**	**New molecular entities = 42**					
1	Lutetium dotatate Lu-177 (Lutathera)	AAA USA Inc	Somatostatin receptor-targeted radiopharmaceutical	Gastroenteropancreatic neuroendocrine tumors (GEP-NETs)	01/26/2018	P, O
2	Bictegravir sodium; Emtricitabine; Tenofovir alafenamide fumarate (Biktarvy)	Gilead Sciences	HIV-1 integrase inhibitor plus HIV-1 nucleoside/nucleotide reverse transcriptase inhibitors	HIV-1 infection	02/07/2018	P
3	Tezacaftor; Ivacaftor (Symdeko)	Vertex Pharmas	Cystic fibrosis transmembrane conductance regulator (CFTR) corrector and CFTR potentiator	Cystic fibrosis	02/12/2018	P, O
4	Apalutamide (Erleada)	Janssen Biotech	Androgen receptor inhibitor	Prostate cancer	02/14/2018	P
5	Fostamatinib disodium (Tavalisse)	Rigel Pharmas	Spleen tyrosine kinase (SYK) inhibitor	Immune thrombocytopenic purpura	04/17/2018	S, O
6	Fosnetupitant chloride HCl; Palonosetron HCl (Akynzeo IV)	Helsinn Group	Neurokinin 1 (NK1) receptor antagonist plus 5-HT_3 receptor antagonist	Chemotherapy-induced nausea	04/19/2018	S
7	Lofexidine HCl (Lucemyra)	US WorldMeds	α2-adrenoceptor agonist	Opioid withdrawal	05/16/2018	P
8	Sodium zirconium cyclosilicate (Lokelma)	AstraZeneca	Potassium binder	Hyperkaliemia	05/18/2018	S
9	Avatrombopag maleate (Doptelet)	Akarx	Thrombopoietin receptor agonist	Thrombocytopenia	05/21/2018	P
10	Baricitinib (Olumiant)	Eli Lilly & Co	Reversible Janus kinase (JAK) inhibitor	Rheumatoid arthritis	05/31/2018	S
11	Moxidectin	MDGH	Antihelmintic GABA receptor and glutamate channel modulator	River blindness	06/13/2018	P, O
12	Plazomicin sulfate (Zemdri)	Cipla USA	Aminoglycoside antibacterial	Urinary tract infections	06/25/2018	P
13	Cannabidiol (Epidiolex)	GW Res Ltd	Cannabinoid	Dravet syndrome and Lennox-Gastaut syndrome	06/25/2018	P, O
14	Encorafenib (Braftovi)	Array BioPharma	BRAF inhibitor	*BRAF*-mutated melanoma	06/27/2018	S, O
15	Binimetinib (Mektovi)	Array BioPharma	MEK inhibitor	*BRAF*-mutated melanoma	06/27/2018	S, O
16	Tecovirimat (Tpoxx)	SIGA Technologies	Viral p37 protein inhibitor	Smallpox	07/13/2018	P, O
17	Tafenoquine succinate (Krintafel)	GlaxoSmithKline	8-Aminoquinoline antimalarial	Plasmodium vivax malaria	07/20/2018	P, O
18	Ivosidenib (Tibsovo)	Servier	Isocitrate dehydrogenase-1 (IDH1) inhibitor	*IDH1*-mutated acute myeloid leukemia (AML)	07/20/2018	P, O
19	Elagolix sodium (Orilissa)	AbbVie	GnRH receptor antagonist	Pain associated with endometriosis	07/23/2018	P
20	Fish oil triglycerides (Omegaven)	Fresenius Kabi USA	Mixture of fatty acids	Parenteral nutrition-associated cholestasis	07/27/2018	P, O
21	Lusutrombopag (Mulpleta)	Shionogi Inc	Thrombopoietin receptor agonist	Thrombocytopenia	07/31/2018	P
22	Migalastat HCl (Galafold)	Amicus Therap US	α-galactosidase regulator	Fabry disease	08/10/2018	P, O
23	Ethinyl estradiol; Segesterone acetate (Annovera)	Therapeuticsmd	Estrogen and progestin combined hormonal contraceptive	Female contraception	08/10/2018	S
24	Patisiran sodium (Onpattro)	Alnylam Pharms	Transthyretin (TTR)-directed small interfering RNA	Hereditary TTR-mediated amyloidosis	08/10/2018	P, O
25	Stiripentol (Diacomit)	Biocodex	γ-aminobutyric acid (GABA) reuptake inhibitor	Dravet syndrome	08/20/2018	P, O
26	Eravacycline 2HCl (Xerava)	Tetraphase Pharms	Tetracycline antibiotic	Complicated intra-abdominal infections	08/27/2018	P
27	Doravirine (Pifeltro)	Merck & Co	Non-nucleoside reverse transcriptase inhibitor	HIV-1 infection	08/30/2018	S
28	Duvelisib (Copiktra)	Secura	Phosphatidylinositol 3-kinase δ/γ (PI3Kδ/γ) inhibitor	Chronic lymphocytic leukaemia (CLL), follicular lymphoma (FL), and small lymphocytic lymphoma (SLL)	09/24/2018	P, O
29	Dacomitinib (Vizimpro)	Pfizer	EGFR inhibitor	*EGFR*-mutated NSCLC	09/27/2018	P, O
30	Sarecycline HCl (Seysara)	Almirall	Tetracycline antibiotic	Severe acne vulgaris	10/01/2018	S
31	Omadacycline tosylate (Nuzyra)	Paratek Pharms	Tetracycline antibiotic	Community-acquired bacterial pneumonia (CABP) and acute bacterial skin and skin structure infection (ABSSSI)	10/02/2018	P
32	Inotersen sodium (Tegsedi)	Akcea Theraps	Transthyretin (TTR)-directed antisense oligonucleotide	Hereditary TTR-mediated amyloidosis	10/05/2018	P, O
33	Talazoparib tosylate (Talzenna)	Pfizer	Poly-ADP ribose polymerase (PARP) inhibitor	*BRCA*-mutated HER2-negative breast cancer	10/16/2018	P
34	Baloxavir marboxil (Xofluza)	Genentech	Polymerase acidic endonuclease inhibitor	Acute uncomplicated influenza	10/24/2018	P
35	Lorlatinib (Lorbrena)	Pfizer	Multi-target TKI (ALK and ROS1)	ALK-positive NSCLC	11/02/2018	P, O
36	Revefenacin (Yupelri)	Mylan Ireland	Long-acting muscarinic receptor antagonist	Chronic obstructive pulmonary disease (COPD)	11/09/2018	S
37	Rifamycin sodium (Aemcolo)	Cosmo Technologies	Ansamycin antibacterial	Travelers' diarrhea	11/16/2018	P
38	Glasdegib maleate (Daurismo)	Pfizer	Smoothened (Hedgehog pathway) inhibitor	Acute myeloid leukemia (AML)	11/21/2018	P, O
39	Larotrectinib sulfate (Vitrakvi)	Bayer	TRKs inhibitor	NTRK-positive solid cancers	11/26/2018	P, O
40	Amifampridine phosphate (Firdapse)	Catalyst Pharms	Potassium channel blocker	Lambert-Eaton myasthenic syndrome	11/28/2018	P, O
41	Gilteritinib fumarate (Xospata)	Astellas	Fms-like tyrosine kinase 3 (FLT3) inhibitor	FLT3-positive AML	11/28/2018	P, O

Supplementary data: Table 1 – FDA-approved drugs (Continued)

	Drug (brand name)	Sponsor	Properties	Indication	Approval date	Review
42	Prucalopride succinate (Motegrity)	Takeda Pharms USA	5-HT4 receptor agonist	Chronic idiopathic constipation	12/14/2018	S
	**2018**	**Biologics license applications = 17**				
1	Ibalizumab (Trogarzo)	TaiMed Biologics USA	CD4-directed mAb	HIV-1 infection	03/06/2018	P, O
2	Tildrakizumab (Ilumya)	Sun Pharma Global	Interleukin-23 (IL-23)-directed mAb	Plaque psoriasis	03/20/2018	S
3	Burosumab (Crysvita)	Ultragenyx Pharm	Fibroblast growth factor 23 (FGF23)-directed mAb	X-linked hypophosphatemia	04/17/2018	P, O
4	Erenumab (Aimovig)	Amgen Inc	Calcitonin gene-related peptide receptor (CGRPR)-directed mAb	Migraine	05/17/2018	S
5	Pegvaliase (Palynziq)	BioMarin Pharm	Recombinant phenylalanine ammonia lyase (PAL) enzyme	Phenylketonuria	05/24/2018	P, O
6	Mogamulizumab (Poteligeo)	Kyowa Kirin	CC chemokine receptor 4 (CCR4)-directed mAb	Mycosis fungoides and Sézary syndrome	08/08/2018	P, O
7	Cenegermin (Oxervate)	Dompe Farmaceutici	Recombinant human nerve growth factor (NGF)	Neurotrophic keratitis	08/22/2018	P, O
8	Lanadelumab (Takhzyro)	Dyax Corp	Kallikrein-directed mAb	Hereditary angioedema	08/23/2018	P, O
9	Moxetumomab pasudotox (Lumoxiti)	Innate Pharma	CD22-directed ADC	Hairy cell leukemia	09/13/2018	P, O
10	Fremanezumab (Ajovy)	Teva Pharms USA	Calcitonin gene-related peptide (CGRP)-directed mAb	Migraine	09/14/2018	P
11	Galcanezumab (Emgality)	Eli Lilly & Co	Calcitonin gene-related peptide (CGRP)-directed mAb	Migraine	09/27/2018	S
12	Cemiplimab (Libtayo)	Regeneron Pharmaceuticals	Programmed death receptor-1 (PD1)-directed mAb	Metastatic or locally advanced cutaneous squamous cell carcinoma (CSCC)	09/28/2018	P
13	Elapegademase (Revcovi)	Leadiant Biosci	Recombinant human adenosine deaminase	Adenosine deaminase severe combined immune deficiency (ADA-SCID)	10/05/2018	P, O
14	Emapalumab (Gamifant)	Novimmune S.A.	Interferon-γ (INFγ)-directed mAb	Primary hemophagocytic lymphohistiocytosis	11/20/2018	P, O
15	Calaspargase pegol (Asparlas)	Servier Pharma	Asparagine specific enzyme	Acute lymphoblastic leukemia (ALL)	12/20/2018	S, O
16	Ravulizumab (Ultomiris)	Alexion Pharm	Complement 5 (C5)-directed mAb	Paroxysmal nocturnal hemoglobinuria	12/21/2018	S, O
17	Tagraxofusp (Elzonris)	Stemline Therapeutics	IL-3 and diphtheria toxin fusion protein	Blastic plasmacytoid dendritic cell neoplasm	12/21/2018	P, O

Supplementary data: Table 1 – FDA-approved drugs (Continued)

	Drug (brand name)	Sponsor	Properties	Indication	Approval date	Review
	**2019** **New molecular entities = 38**					
1	Triclabendazole (Egaten)	Novartis	Anthelmintic, MOA unknown	Fascioliasis	02/13/2019	P, O
2	Brexanolone (Zulresso)	Sage Therap	γ-aminobutyric acid A ($GABA_A$) receptor- positive modulator	Postpartum depression	03/19/2019	P
3	Solriamfetol HCl (Sunosi)	Jazz	Dopamine and noradrenaline reuptake inhibitor	Excessive sleepiness due to narcolepsy or obstructive sleep apnea	03/20/2019	S, O
4	Siponimod fumaric acid (Mayzent)	Novartis	Sphingosine 1-phosphate (S1P) receptor modulator	Relapsing forms of multiple sclerosis	03/26/2019	S
5	Erdafitinib (Balversa)	Janssen Biotech	Fibroblast growth factor receptor (FGFR) inhibitor	Bladder cancer	04/12/2019	P
6	Tafamidis meglumine (Vyndaqel)	Foldrx Pharms	Transthyretin stabilizer	Heart disease caused by transthyretin amyloid cardiomyopathy (ATTR-CM)	05/03/2019	P, O
7	Alpelisib (Piqray)	Novartis	Phosphatidylinositol 3-kinase α (PI3Kα) inhibitor	Breast cancer	05/24/2019	P
8	Bremelanotide acetate (Vyleesi)	Palatin Technologies	Melanocortin receptor agonist	Hypoactive sexual desire disorder	06/21/2019	S
9	Selinexor (Xpovio)	Karyopharm Theraps	Exportin 1 (XPO1) inhibitor	Multiple myeloma	07/03/2019	P, O
10	Cilastatin sodium; Imipenem; Relebactam (Recarbrio)	Msd Merck Co	Renal dehydropeptidase inhibitor; Penem antibacterial; β-lactamase inhibitor	Complicated urinary tract and complicated intra-abdominal infections	07/16/2019	P
11	Ferric maltol (Accrufer)	Shield TX	Iron replacement product	Iron deficiency anemia	07/25/2019	S
12	Darolutamide (Nubeqa)	Bayer Healthcare	Androgen receptor inhibitor	Prostate cancer	07/30/2019	P
13	Pexidartinib HCl (Turalio)	Daiichi Sankyo Inc	CSF1R, KIT, and FLT3 inhibitor	Tenosynovial giant cell tumor	08/02/2019	P, O
14	Pitolisant HCl (Wakix)	Harmony	Histamine H3 receptor antagonist/inverse agonist	Excessive sleepiness due to narcolepsy	08/14/2019	P, O
15	Pretomanid (Pretomanid)	Mylan Ireland Ltd	Antimycobacterial	Tuberculosis	08/14/2019	P, O
16	Entrectinib (Rozlytrek)	Genentech Inc	Multi-target TKI (TRKs, ROS1, and ALK)	NTRK fusion- positive solid tumors and ROS1-positive NSCLC	08/15/2019	P, O
17	Upadacitinib (Rinvoq)	AbbVie Inc	JAK inhibitor	Rheumatoid arthritis	08/16/2019	S
18	Fedratinib HCl (Inrebic)	Impact	JAK2 and FLT3 inhibitor	Myelofibrosis	08/16/2019	P, O
19	Lefamulin acetate (Xenleta)	Nabriva	Pleuromutilin antibacterial	Community-acquired bacterial pneumonia (CABP)	08/19/2019	P
20	Gallium dotatoc Ga-68	UIHC PET Imaging	^{68}Ga labelled radioactive diagnostic agent	Localization of somatostatin receptor-positive neuroendocrine tumors	08/21/2019	S, O
21	Istradefylline (Nourianz)	Kyowa Kirin	Adenosine receptor antagonist	Parkinson disease, 'off' episodes	08/27/2019	S
22	Tenapanor HCl (Ibsrela)	Ardelyx Inc	Sodium/hydrogen exchanger 3 (NHE3) inhibitor	Irritable bowel syndrome (IBS) with constipation	09/12/2019	S
23	Trifarotene (Aklief)	Galderma Labs Lp	Retinoic acid receptor agonist	Acne vulgaris	10/04/2019	S
24	Afamelanotide (Scenesse)	Clinuvel Inc	Melanocortin 1 receptor agonist	Erythropoietic protoporphyria	10/08/2019	P, O
25	Fluorodopa F-18	Feinstein	^{18}F labelled radioactive diagnostic agent	Diagnosis of parkinsonian syndromes	10/10/2019	S
26	Lasmiditan succinate (Reyvow)	Eli Lilly & Co	5-HT_{1F} receptor agonist	Migraine with or without aura	10/11/2019	S
27	Tezacaftor; Elexacaftor; Ivacaftor (Trikafta)	Vertex Pharms Inc	Two cystic fibrosis transmembrane conductance regulator (CFTR) correctors and a CFTR potentiator	Most common gene mutation that causes cystic fibrosis	10/21/2019	P, O
28	Air polymer-type A (ExEm Foam kit)	Giskit	Ultrasound contrast agent	Assess fallopian tube patency in women with known or suspected infertility	11/07/2019	S
29	Cefiderocol sulfate tosylate (Fetroja)	Shionogi Inc	Cephalosporin antibacterial, inhibits cell wall synthesis	Complicated urinary tract infections	11/14/2019	P
30	Zanubrutinib (Brukinsa)	BeiGene	Bruton's tyrosine kinase (BTK) inhibitor	Mantle cell lymphoma	11/14/2019	P, O
31	Givosiran sodium (Givlaari)	Alnylam Pharms Inc	5-aminolevulinic acid synthase (ALAS1)-directed siRNA	Acute hepatic porphyria	11/20/2019	P, O
32	Cenobamate (Xcopri)	SK Life	Voltage-gated sodium channel inhibitor and γ-aminobutyric acid A ($GABA_A$) receptor- positive modulator	Partial onset seizures	11/21/2019	S
33	Voxelotor (Oxbryta)	Global Blood Theraps	Hemoglobin S polymerization inhibitor	Sickle cell disease (SCD)	11/25/2019	P, O
34	Golodirsen (Vyondys 53)	Sarepta Theraps Inc	Exon 53 skipping antisense	Duchenne muscular dystrophy	12/12/2019	P, O
35	Lumateperone tosylate (Caplyta)	Intra-Cellular	Atypical antipsychotic	Schizophrenia	12/20/2019	S
36	Brilliant Blue G (Tissueblue)	Dutch Ophthalmic	Brilliant Blue G dye	Staining the internal limiting membrane	12/20/2019	P, O
37	Lemborexant (Dayvigo)	Eisai Inc	Orexin receptor antagonist	Insomnia	12/20/2019	S
38	Ubrogepant (Ubrelvy)	Allergan	Calcitonin gene- related peptide (CGRP) receptor antagonist	Migraine with or without aura	12/23/2019	S
	**2019** **Biologics license applications = 10**					
1	PrabotulinumtoxinA (Jeuveau)	Evolus Inc	Acetylcholine release inhibitor and a neuromuscular blocking agent	Glabellar lines associated with corrugator and/or procerus muscle activity	02/01/2019	S
2	Caplacizumab (Cablivi)	Ablynx NV	von Willebrand factor (vWF)-directed nanobody	Acquired thrombotic thrombocytopenic purpura (ATTP)	02/06/2019	P, O

Supplementary data: Table 1 – FDA-approved drugs (Continued)

	Drug (brand name)	Sponsor	Properties	Indication	Approval date	Review
	**2019** **Biologics license applications = 10**					
3	Romosozumab (Evenity)	Amgen Inc	Sclerostin-directed mAb	Osteoporosis	04/09/2019	S
4	Risankizumab (Skyrizi)	AbbVie Inc	Interleukin-23 (IL-23)-directed mAb	Plaque psoriasis	04/23/2019	S
5	Polatuzumab vedotin (Polivy)	Genentech	CD79b-directed antibody-drug conjugate (ADC)	Diffuse large B-cell lymphoma	06/10/2019	P, O
6	Brolucizumab (Beovu)	Novartis	VEGFA-directed antibody-drug conjugate (ADC)	Wet age-related macular degeneration	10/07/2019	S
7	Luspatercept (Reblozyl)	Celgene	Recombinant fusion protein comprised of modified extracellular domain of activin receptor type IIB and FC domain of human IgG1	Anemia in β-thalassemia	11/08/2019	P, O
8	Crizanlizumab (Adakveo)	Novartis	P-selectin blocker	Painful complications of sickle cell disease (SCD)	11/15/2019	P, O
9	Enfortumab vedotin (Padcev)	Astellas	Nectin-4-directed antibody-drug conjugate (ADC)	Urothelial carcinoma	12/18/2019	P
10	Trastuzumab deruxtecan (Enhertu)	Daiichi Sankyo	HER2-directed antibody-drug conjugate (ADC)	HER2-positive breast cancer	12/20/2019	P

Supplementary data: Table 1 – FDA-approved drugs (Continued)

	Drug (brand name)	Sponsor	Properties	Indication	Approval date	Review
	**2020** **New molecular entities = 40**					
1	Avapritinib (Ayvakit)	Blueprint Medicines	PDGFRα, PDGFRα mutants, and KIT inhibitor	Gastrointestinal stromal tumor (GIST) with *PDGFRA* exon 18 mutations	01/09/2020	P, O
2	Tazemetostat (Tazverik)	Epizyme Inc	Enhancer of Zeste Homolog 2 (EZH2) inhibitor	Epithelioid sarcoma	01/23/2020	P, O
3	Lactitol (Pizensy)	Braintree Labs	Osmotic laxative	Chronic idiopathic constipation	02/12/2020	S
4	Bempedoic acid (Nexletol)	Esperion Therapeutics	Adenosine triphosphate-citrate lyase (ACL) inhibitor	Heterozygous familial hypercholesterolemia (HeFH) or atherosclerotic cardiovascular disease	02/21/2020	S
5	Amisulpride (Barhemsys)	Acacia	Dopamine D2 receptor antagonist	Nausea and vomiting after surgery	02/26/2020	S
6	Rimegepant sulfate (Nurtec ODT)	Biohaven	Calcitonin gene-related peptide (CGRP) receptor antagonist	Migraine	02/27/2020	S
7	Osilodrostat phosphate (Isturisa)	Recordati Rare Diseases	Cortisol synthesis inhibitor	Cushing's disease	03/06/2020	S, O
8	Ozanimod HCl (Zeposia)	Celgene	Sphingosine 1-phosphate (S1P) receptor modulator	Multiple sclerosis	03/25/2020	S
9	Selumetinib sulfate (Koselugo)	AstraZeneca	MEK1/2 kinase inhibitor	Neurofibromatosis type 1	04/10/2020	P, O
10	Tucatinib (Tukysa)	Seagen	HER2 inhibitor	HER2-positive breast cancer	04/17/2020	P, O
11	Pemigatinib (Pemazyre)	Incyte	Fibroblast growth factor receptor 1-3 (FGFR1-3) kinase inhibitor	Advanced cholangiocarcinoma with *FGFR2* fusions/rearrangements	04/17/2020	P, O
12	Opicapone (Ongentys)	Neurocrine	Catechol-O-methyltransferase (COMT) inhibitor	Parkinson's disease	04/24/2020	S
13	Capmatinib HCl (Tabrecta)	Novartis	MET kinase inhibitor	Non-small-cell lung cancer (NSCLC)	05/06/2020	P, O
14	Selpercatinib (Retevmo)	Loxo Oncology	RET kinase inhibitor	RET fusion-positive NSCLC and thyroid cancer	05/08/2020	P, O
15	Ripretinib (Qinlock)	Deciphera	PDGFRα, PDGFRα mutants, and KIT inhibitor	Advanced gastrointestinal stromal tumor (GIST)	05/15/2020	P, O
16	Fluoroestradiol F-18 (Cerianna)	Zionexa	^{18}F labelled radioactive diagnostic agent	Imaging, breast cancer	05/20/2020	S
17	Artesunate	Amivas	Artemisinin antimalarial	Severe malaria	05/26/2020	P, O
18	Flortaucipir F-18 (Tauvid)	Avid Radiopharms	^{18}F labelled radioactive diagnostic agent	Imaging, tau in Alzheimer's disease	05/28/2020	P
19	Lurbinectedin (Zepzelca)	Jazz	DNA alkylating drug	Small-cell lung cancer	06/15/2020	P, O
20	Triheptanoin (Dojolvi)	Ultragenyx	Medium-chain triglyceride	Long chain fatty acid oxidation disorders (LC-FAODs)	06/30/2020	S, O
21	Remimazolam besylate (Byfavo)	Acacia	Benzodiazepine	Procedural sedation	07/02/2020	S
22	Fostemsavir tromethamine (Rukobia)	Viiv Healthcare	HIV-1 attachment inhibitor	HIV-1 infection	07/02/2020	P
23	Cedazuridine; Decitabine (Inqovi)	Otsuka	Cytidine deaminase inhibitor; Nucleoside metabolic inhibitor	Myelodysplastic syndromes	07/07/2020	P, O
24	Abametapir (Xeglyze)	Dr Reddy's	Metalloproteinase inhibitor	Head lice	07/24/2020	S
25	Nifurtimox (Lampit)	Bayer	Nitrofuran antiprotozoal	Chagas disease	08/06/2020	P, O
26	Oliceridine (Olinvyk)	Trevena	Opioid receptor agonist	Acute pain	08/07/2020	S
27	Risdiplam (Evrysdi)	Genentech	Survival of motor neuron 2 (SMN2) splicing modifier	Spinal muscular atrophy	08/07/2020	P, O
28	Viltolarsen (Viltepso)	Nippon Shinyaku	Dystrophin splicing modifier	Duchenne muscular dystrophy	08/12/2020	P, O
29	Clascoterone (Winlevi)	Sun Pharm	Androgen receptor inhibitor	Acne vulgaris	08/26/2020	S
30	Copper dotatate Cu-64 (Detectnet)	Radiomedix	^{64}Cu labelled somatostatin analog	Imaging, cancer	09/03/2020	P, O
31	Pralsetinib (Gavreto)	Genentech	RET kinase inhibitor	RET fusion-positive NSCLC	09/04/2020	P, O
32	Remdesivir (Veklury)	Gilead Sciences	Nucleotide analog RNA polymerase inhibitor	COVID-19 infection	10/22/2020	P
33	Lonafarnib (Zokinvy)	Eiger Biopharms	Farnesyltransferase inhibitor	Hutchinson-Gilford progeria syndrome (HGPS)	11/20/2020	P, O
34	Lumasiran sodium (Oxlumo)	Alnylam	Hydroxyacid oxidase 1 (HAO1)-directed siRNA	Hyperoxaluria type 1	11/23/2020	P, O
35	Setmelanotide acetate (Imcivree)	Rhythm	Melanocortin 4 (MC4) receptor agonist	Rare genetic diseases of obesity	11/25/2020	P, O
36	PSMA-11 Ga-68	UCLA	^{68}Ga labelled radioactive diagnostic agent	Imaging, prostate cancer	12/01/2020	S
37	Berotralstat HCl (Orladeyo)	BioCryst	Plasma kallikrein inhibitor	Hereditary angioedema	12/03/2020	S, O
38	Tirbanibulin (Klisyri)	Almirall	Microtubule inhibitor	Actinic keratosis	12/14/2020	S
39	Relugolix (Orgovyx)	Myovant Sciences	GnRH receptor antagonist	Prostate cancer	12/18/2020	P
40	Vibegron (Gemtesa)	Urovant Sciences	β3-adrenoceptor agonist	Overactive bladder	12/23/2020	S
	**2020** **Biologics license applications = 13**					
1	Teprotumumab (Tepezza)	Horizon Therapeutics	Insulin-like growth factor-1 receptor (IGF1R)-directed mAb	Thyroid eye disease	01/21/2020	P, O
2	Eptinezumab (Vyepti)	Lundbeck	Calcitonin gene-related peptide (CGRP)-directed mAb	Migraine	02/21/2020	S

Supplementary data: Table 1 – FDA-approved drugs (Continued)

	Drug (brand name)	Sponsor	Properties	Indication	Approval date	Review
	**2020** **Biologics license applications = 13**					
3	Isatuximab (Sarclisa)	Sanofi	CD38-directed mAb	Multiple myeloma	03/02/2020	S, O
4	Sacituzumab govitecan (Trodelvy)	Immunomedics Inc	Trophoblast cell surface antigen 2 (Trop2)-directed ADC	Triple-negative breast cancer	04/22/2020	P
5	Inebilizumab (Uplizna)	Viela Bio	CD19-directed mAb	Neuromyelitis optica spectrum disorder (NMOSD)	06/11/2020	S, O
6	Tafasitamab (Monjuvi)	MorphoSys	CD19-directed mAb	Diffuse large B-cell lymphoma (DLBCL)	07/31/2020	P, O
7	Belantamab mafodotin (Blenrep)	GlaxoSmithKline	B-cell maturation antigen (BCMA)-directed ADC	Multiple myeloma	08/05/2020	P, O
8	Satralizumab (Enspryng)	Genentech	Interleukin-6 receptor (IL-6R)-directed mAb	Neuromyelitis optica spectrum disorder (NMOSD)	08/14/2020	S, O
9	Somapacitan (Sogroya)	Novo Nordisk	Growth hormone analog	Growth hormone deficiency	08/28/2020	S
10	Atoltivimab; Odesivimab; Maftivimab (Inmazeb)	Jazz	Cocktail of Ebola glycoprotein-directed mAbs	Ebola virus	10/14/2020	P, O
11	Naxitamab (Danyelza)	Y-mAbs Therapeutics	GD2-directed mAb	High-risk neuroblastoma	11/25/2020	P, O
12	Margetuximab (Margenza)	MacroGenics	HER2-directed mAb	HER2-positive breast cancer	12/16/2020	S
13	Ansuvimab (Ebanga)	Ridgeback	Ebola glycoprotein-directed mAb	Ebola virus	12/21/2020	P, O

Supplementary data: Table 1 – FDA-approved drugs (Continued)

	Drug (brand name)	Sponsor	Properties	Indication	Approval date	Review
	**2021**	**New molecular entities = 36**				
1	Vericiguat (Verquvo)	Merck & Co	Soluble guanylate cyclase (sGC) stimulator	Chronic heart failure	01/19/2021	P
2	Cabotegravir; Rilpivirine (Cabenuva Kit)	Viiv Hlthcare	Integrase strand transfer inhibitor (INSTI) and non-nucleoside reverse transcriptase inhibitor (NNRTI) of HIV-1	HIV-1 infection	01/21/2021	P
3	Voclosporin (Lupkynis)	Aurinia	Calcineurin inhibitor	Lupus nephritis	01/22/2021	P
4	Tepotinib HCl (Tepmetko)	Emd Serono	MET inhibitor	Non-small-cell lung cancer (NSCLC)	02/03/2021	P, O
5	Umbralisib tosylate (Ukoniq)	TG Theraps	PI3Kδ and CK1ε inhibitor	Relapsed or refractory marginal zone lymphoma (MZL), follicular lymphoma	02/05/2021	P, O
6	Trilaciclib 2HCl (Cosela)	G1 Therap	CDK4/6 kinase inhibitor	Chemotherapy-induced myelosuppression	02/12/2021	P
7	Casimersen (Amondys 45)	Sarepta	Exon 45-skipping antisense oligonucleotide (ASO)	Duchenne muscular dystrophy (DMD)	02/25/2021	P, O
8	Fosdenopterin HCl (Nulibry)	Origin	Cyclic pyranopterin monophosphate (cPMP)	Molybdenum cofactor deficiency (MoCD) type A	02/26/2021	P, O
9	Melphalan flufenamide HCl (Pepaxto)	Oncopeptides	Peptide-conjugated alkylating drug	Multiple myeloma	02/26/2021	P, O
10	Dexmethylphenidate HCl; Serdexmethylphenidate chloride (Azstarys)	Commave Therap	Central nervous system (CNS) stimulant	Attention deficit hyperactivity disorder (ADHD)	03/02/2021	S
11	Tivozanib HCl (Fotivda)	Aveo Pharms	VEGFRs inhibitor	Advanced renal cell carcinoma	03/10/2021	S
12	Ponesimod (Ponvory)	Janssen Pharms	Sphingosine 1- phosphate (S1P) receptor modulator	Relapsing multiple sclerosis	03/18/2021	S
13	Dasiglucagon HCl (Zegalogue)	Zealand Pharma	Glucagon receptor agonist	Severe hypoglycemia	03/22/2021	S
14	Viloxazine HCl (Qelbree)	Supernus	Selective norepinephrine reuptake inhibitor (NRI)	Attention deficit hyperactivity disorder (ADHD)	04/02/2021	S
15	Drospirenone; Estetrol (Nextstellis)	Mayne Pharma	Spironolactone and estrogen analog	Contraception	04/15/2021	S
16	Pegcetacoplan (Empaveli)	Apellis	Complement protein C3 inhibitor	Paroxysmal nocturnal hemoglobinuria (PNH)	05/14/2021	P, O
17	Piflufolastat F-18 (Pylarify)	Progenics	^{18}F labelled prostate-specific membrane antigen (PSMA) imaging agent	Prostate cancer imaging	05/26/2021	P
18	Olanzapine; Samidorphan L-malate (Lybalvi)	Alkermes	Atypical antipsychotic and opioid antagonist	Schizophrenia and bipolar I disorder	05/28/2021	S
19	Infigratinib phosphate (Truseltiq)	Helsinn Hlthcare	FGFR1-3 inhibitor	Advanced cholangiocarcinoma with *FGFR2* fusions/rearrangements	05/28/2021	P, O
20	Sotorasib (Lumakras)	Amgen	$KRAS^{G12C}$ inhibitor	$\textit{KRAS}^{G12C}$-mutated NSCLC	05/28/2021	P, O
21	Ibrexafungerp citrate (Brexafemme)	Scynexis	Triterpenoid antifungal	Vulvovaginal candidiasis	06/01/2021	P
22	Finerenone (Kerendia)	Bayer	Non-steroidal mineralocorticoid receptor (MR) antagonist	Chronic kidney disease (CKD) with type 2 diabetes	07/09/2021	P
23	Fexinidazole (Fexinidazole)	Sanofi	Nitroimidazole antimicrobial	Sleeping sickness	07/16/2021	P, O
24	Belumosudil mesylate (Rezurock)	Kadmon	Rho-associated coiled-coil-containing protein kinase 2 (ROCK2) kinase inhibitor	Chronic graft-versus-host disease (GVHD)	07/16/2021	P, O
25	Odevixibat (Bylvay)	Albireo	Ileal bile acid transporter (IBAT) inhibitor	Pruritus in progressive familial intrahepatic cholestasis (PFIC)	07/20/2021	P, O
26	Belzutifan (Welireg)	Merck & Co	Hypoxia-inducible factor 2α (HIF-2α) inhibitor	von Hippel-Lindau (VHL) disease	08/13/2021	P, O
27	Difelikefalin (Korsuva)	Cara Therap	κ-Opioid receptor agonist	Pruritus associated with chronic kidney disease (CKD)	08/23/2021	P
28	Mobocertinib succinate (Exkivity)	Takeda	EGFR kinase inhibitor	*EGFR* exon 20-mutated NSCLC	09/15/2021	P, O
29	Atogepant (Qulipta)	AbbVie	Calcitonin gene-related peptide (CGRP) receptor antagonist	Episodic migraine	09/28/2021	S
30	Maralixibat chloride (Livmarli)	Mirum	Ileal bile acid transporter (IBAT) inhibitor	Pruritus in Alagille syndrome	09/29/2021	P, O
31	Avacopan (Tavneos)	ChemoCentryx	Complement 5a receptor antagonist	Anti-neutrophil cytoplasmic (auto)antibody (ANCA)-associated vasculitis	10/07/2021	S, O
32	Asciminib HCl (Scemblix)	Novartis	ABL/BCR-ABL1 kinase inhibitor	Philadelphia chromosome-positive chronic myeloid leukemia (Ph+ CML)	10/29/2021	P, O
33	Vosoritide (Voxzogo)	Biomarin	C-type naturietic peptide (CNP) analog	Achondroplasia	11/19/2021	P, O
34	Maribavir (Livtencity)	Takeda	Cytomegalovirus (CMV) pUL97 kinase inhibitor	Post-transplant CMV infection	11/23/2021	P, O
35	Pafolacianine sodium (Cytalux)	On Target Labs	Fluorescent folate receptor α (FRα) imaging agent	Ovarian cancer imaging	11/29/2021	P, O
36	Inclisiran sodium (Leqvio)	Novartis	Proprotein convertase subtilisin-kexin type 9 (PCSK9)-targeted siRNA	Heterozygous familial hypercholesterolemia (HeFH) or atherosclerotic cardiovascular disease (ASCVD)	12/22/2021	S
	**2021**	**Biologics license applications = 14**				
1	Evinacumab (Evkeeza)	Regeneron	Angiopoetin-like protein 3 (ANGPTL3)-directed mAb	Homozygous familial hypercholesterolemia (HoFH)	02/11/2021	P, O
2	Dostarlimab (Jemperli)	GlaxoSmithKline	PD1-directed mAb	Endometrial cancer	04/22/2021	P

Supplementary data: Table 1 – FDA-approved drugs (Continued)

	Drug (brand name)	Sponsor	Properties	Indication	Approval date	Review
	**2021** **Biologics license applications = 14**					
3	Loncastuximab tesirine (Zynlonta)	ADC Therapeutics	CD19-directed ADC	B-cell lymphoma	04/23/2021	P, O
4	Amivantamab (Rybrevant)	Janssen Biotech	EGFR- and MET-bispecific antibody	*EGFR* exon 20-mutated NSCLC	05/21/2021	P
5	Aducanumab (Aduhelm)	Biogen	Amyloid-β-directed mAb	Alzheimer’s disease	06/07/2021	P
6	Asparaginase erwinia chrysanthemi (Rylaze)	Jazz	Recombinant asparagine-specific enzyme	Acute lymphoblastic leukaemia (ALL), and lymphoblastic lymphoma (LBL), in patients allergic to *E. coli*-derived products	06/30/2021	S, O
7	Anifrolumab (Saphnelo)	AstraZeneca	Type 1 interferon receptor (IFNAR)-targeted mAb	Systemic lupus erythematosus (SLE)	07/30/2021	S
8	Avalglucosidase alfa (Nexviazyme)	Genzyme	Recombinant α-glucosidase	Pompe disease	08/06/2021	P, O
9	Lonapegsomatropin (Skytrofa)	Ascendis Pharma	PEGylated human growth hormone	Growth failure due to growth hormone deficiency (GHD)	08/25/2021	S, O
10	Tisotumab vedotin (Tivdak)	Seagen	Tissue-factor-targeted ADC	Cervical cancer	09/20/2021	P, O
11	Ropeginterferon alfa-2b (Besremi)	PharmaEssentia	PEGylated interferon α-2b	Polycythemia vera	11/12/2021	S, O
12	Efgartigimod alfa (Vyvgart)	Argenx	Neonatal Fc receptor (FcRn)-binding Fc fragment	Myasthenia gravis	12/17/2021	S, O
13	Tezepelumab (Tezspire)	AstraZeneca	Thymic stromal lymphopoietin (TSLP)-directed mAb	Severe asthma	12/17/2021	P
14	Tralokinumab (Adbry)	LEO Pharma	LEO Pharma Interleukin-13 (IL-13)-directed mAb	Atopic dermatitis	12/27/2021	S

Supplementary data: Table 2 – FDA-approved cancer drugs

	Drug (brand name)	Sponsor	Properties	Indication	Approval date	Review
	1991	New molecular entities = 5				
1	Ondansetron HCl (Zofran)	Novartis	5-HT3 receptor antagonist	Nausea and vomiting caused by radiotherapy, anesthesia, surgery or cytotoxic chemotherapy drugs	01/04/1991	P
2	Gallium nitrate (Ganite)	Chapter 7 Trustee	Bone-resorption inhibitor	Hypercalcemia of malignancy	01/17/1991	P, O
3	Fludarabine phosphate (Fludara)	Genzyme Corp	DNA synthesis inhibitor	B-cell chronic lymphocytic leukemia	04/18/1991	P
4	Pentostatin (Nipent)	Hospira	Purine analog, irreversible inhibitor of adenosine deaminase	INFα-refractory hairy cell leukemia	10/11/1991	P, O
5	Pamidronate disodium (Aredia)	Novartis	Bone-resorption inhibitor	Hypercalcemia of malignancy	10/31/1991	P
	1991	Biologics license applications = 2				
1	Filgrastim (Neupogen)	Amgen	Recombinant human granulocyte colony-stimulating factor (G-CSF)	Severe neutropenia in patients with non-myeloid malignancies	02/20/1991	N/A
2	Sargramostim (Leukine)	Berlex Labs	Recombinant human granulocyte macrophage colony-stimulating factor (GM-CSF)	Myeloid reconstitution after PBPC/bone marrow transplantation, or with NHL, ALL, HL	03/05/1991	O

	Drug (brand name)	Sponsor	Properties	Indication	Approval date	Review
	1992	New molecular entities = 2				
1	Teniposide (Vumon)	HQ spclt Pharma	Topoisomerase II inhibitor	Refractory childhood acute lymphoblastic leukemia	07/14/1992	P, O
2	Paclitaxel (Taxol)	HQ Spclt Pharma	Microtubule-stabilizing agent	Advanced ovarian cancer	12/29/1992	P, O
	1992	Biologics license applications = 2				
1	Aldesleukin (Proleukin)	Chiron	Recombinant human interleukin-2	Metastatic renal cell carcinoma	05/05/1992	O
2	Indium In-111 satumomab pendetide (OncoScint)	Cytogen	TAG-72-specific radiolabeled mAb	Imaging of colorectal or ovarian cancer	12/29/1992	O

	Drug (brand name)	Sponsor	Properties	Indication	Approval date	Review
	1993	New molecular entities = 3				
1	Cladribine (Leustatin)	Janssen Pharms	Adenosine deaminase inhibitor	Hairy cell leukemia and multiple sclerosis	02/26/1993	S
2	Strontium chloride Sr-89 (Metastron)	Q Biomed	A radiopharmaceutical that concentrates in the bone of invaded tumor	Bone metastasis of tumor induced pain	06/18/1993	P
3	Granisetron HCl (Kytril)	Roche	5-HT3 receptor agonist	Chemotherapy-induced nausea	12/29/1993	S

	Drug (brand name)	Sponsor	Properties	Indication	Approval date	Review
	1994	New molecular entities = 4				
1	Iobenguane sulfate I-131	Pharmalucence	Radiopharmaceutical	Location of phaeochromocytomas and neuroblastomas	03/25/1994	P
2	Indium In-111 pentetreotide kit (Octreoscan)	Curium	Radioactive diagnostic agent	Location of metastatic neuroblastomas	06/02/1994	P
3	Technetium Tc-99m bicisate kit (Neurolite)	Lantheus Medcl	Radioactive diagnostic agent	Localization of neuroendocrine tumors bearing somatostatin receptors	11/23/1994	S
4	Vinorelbine tartrate (Navelbine)	Pierre Fabre	Microtubule-destabilizing agent	Non-small-cell lung cancer (NSCLC)	12/23/1994	P
	1994	Biologics license applications = 1				
1	Pegaspargase (Oncaspar)	Sigma Tau	*Escherichia coli*-derived L-asparaginase	Pediatric acute lymphoblastic leukaemia	02/01/1994	O

	Drug (brand name)	Sponsor	Properties	Indication	Approval date	Review
	1995	New molecular entities = 5				
1	Dexrazoxane HCl (Zinecard)	Pfizer	DNA topoisomerase II inhibitor and iron ions chelator	Doxorubicin-caused cardiomyopathy in patients with breast cancer	05/26/1995	P, O
2	Bicalutamide (Casodex)	Ani Pharms	Androgen receptor antagonist	Advanced prostate cancer	10/04/1995	S
3	Amifostine (Ethyol)	Clinigen	Binds to and detoxifies cytotoxic platinum-containing metabolites of cisplatin	Cisplatin caused cumulative renal toxicity in patients with ovarian cancer	12/08/1995	P, O
4	Porfimer sodium (Photofrin)	Pinnacle Biolgs	A photosensitizer used for photodynamic therapy	Advanced esophageal cancer	12/27/1995	P, O
5	Anastrozole (Arimidex)	Ani Pharms	Aromatase inhibitor	Advanced breast cancer in post-menopausal women	12/27/1995	S

Supplementary data: Table 2 – FDA-approved cancer drugs (Continued)

	Drug (brand name)	Sponsor	Properties	Indication	Approval date	Review
	1996 New molecular entities = 5					
1	Docetaxel (Taxotere)	Sanofi	Microtubule-stabilizing agent	Locally advanced or metastatic breast cancer	05/14/1996	P
2	Gemcitabine HCl (Gemzar)	Eli Lilly	DNA synthesis inhibitor	Locally advanced or metastatic pancreatic cancer	05/15/1996	P
3	Topotecan HCl (Hycamtin)	Novartis	DNA topoisomerases inhibitor	Relapsed small cell lung cancer	05/28/1996	P
4	Irinotecan HCl (Camptosar)	Pfizer	Topoisomerase I inhibitor	Metastatic colorectal cancer	06/14/1996	P
5	Nilutamide (Nilandron)	Concordia	Androgen receptor antagonist	Metastatic prostate cancer	09/19/1996	S
	1996 Biologics license applications = 3					
1	Arcitumomab (CEA-Scan)	Immunomedics	^{99m}Tc labelled anti-CEA mouse IgG1 (Fab)	Recurrent colorectal cancer imaging	06/28/1996	O
2	Nofetumomab merpentan (Verluma)	Boehringer Ingelheim	^{99m}Tc labelled anti-EpCAM (and/or CD20/MS4A1) mouse IgG2b (Fab)	Small cell lung cancer imaging	8/20/1996	N/A
3	Capromab pendetide (Prostascint)	Cytogen	^{111}In labelled anti-PSMA mouse mAb	Diagnosis of metastatic prostate cancer	10/28/1996	N/A

	Drug (brand name)	Sponsor	Properties	Indication	Approval date	Review
	1997 New molecular entities = 6					
1	Anagrelide HCl (Agrylin)	Takeda	Phosphodiesterase 3 (PDE3) and phospholipase A2 (PLA2) inhibitor	Thrombocythemia, secondary to myeloproliferative neoplasms	12/06/1996	S
2	Samarium Sm-153 lexidronam pentasodium (Quadramet)	Lantheus Medical	^{153}Sm labelled Radiopharmaceutical, chelator moiety associates with hydroxyapatite crystals concentrated in areas of bone turnover	Bone metastasis of tumor induced pain	03/28/1997	S
3	Toremifene citrate (Fareston)	Kyowa kirin	Estrogen receptor (ER) inhibitor	ER-positive metastatic breast cancer	05/29/1997	S, O
4	Letrozole (Femara)	Novartis	Aromatase inhibitor	Advanced breast cancer	07/25/1997	S
5	Dolasetron mesylate (Anzemet)	Validus Pharms	5-HT_3 receptor antagonist	Chemotherapy-induced nausea and vomiting	09/11/1997	S
6	Mangafodipir trisodium (Teslascan)	IC Targets	MRI contrast agent	Imaging of liver lesions with suspected metastatic disease or hepatocellular carcinomas	11/26/1997	S
	1997 Biologics license applications = 2					
1	Oprelvckin (Neumega)	Genetics institute	Recombinant human interleukin-11 (IL-11)	Chemotherapy-induced thrombocytopenia	11/25/1997	N/A
2	Rituximab (Rituxan)	Genentech	CD20-directed mAb	B-cell non-Hodgkin's lymphoma	11/26/1997	O

	Drug (brand name)	Sponsor	Properties	Indication	Approval date	Review
	1998 New molecular entities = 2					
1	Capecitabine (Xeloda)	Roche	Prodrug of 5-FU	Metastatic breast cancer	04/30/1998	P
2	Valrubicin (Valstar)	Endo Pharm	A semisynthetic analog of anthracycline doxorubicin	BCG-refractory CIS of urinary bladder cancer	09/25/1998	P, O
	1998 Biologics license applications = 2					
1	Trastuzumab (Herceptin)	Genentech	HER2-directed mAb	HER2-positive breast cancer	09/25/1998	P
2	Thyrotropin alfa (Thyrogen)	Genzyme	Recombinant human thyroid stimulating hormone	Diagnostic testing for thyroid tissue remnants following radioiodine ablation, and alongside radioactive agents to destroy thyroid tissue	11/30/1998	O

	Drug (brand name)	Sponsor	Properties	Indication	Approval date	Review
	1999 New molecular entities = 6					
1	Alitretinoin (Panretin)	Concordia	9-cis-retinoic acid, a form of vitamin A	AIDS-related Kaposi's sarcoma	02/02/1999	P, O
2	Technetium Tc-99m depreotide (Neo tect kit)	Cis Bio Intl	^{99m}Tc labelled radioactive diagnostic agent	Imaging of malignant lung tumors in patients with highly suspect for malignancy	08/03/1999	P
3	Temozolomide (Temodar)	Merck & Co	DNA alkylating agent	Glioblastoma	08/11/1999	P, O
4	Epirubicin HCl (Ellence)	Pfizer	DNA topoisomerase II inhibitor	Primary breast cancer with axillary node tumor involvement	09/15/1999	P, O
5	Exemestane (Aromasin)	Pfizer	Aromatase inhibitor	ER-positive early breast cancer	10/21/1999	S, O
6	Bexarotene (Targretin)	Valeant Luxembourg	Retinoid, ligand of retinoid X receptors (RXRs)	Cutaneous T-cell lymphoma	12/29/1999	P, O
	1999 Biologics license applications = 1					
1	Denileukin diftitox (Ontak)	Eisai	Fusion protein consisting of diphtheria toxin fragments and interleukin-2	Cutaneous T-cell lymphoma	02/05/1999	O

Supplementary data: Table 2 – FDA-approved cancer drugs (Continued)

	Drug (brand name)	Sponsor	Properties	Indication	Approval date	Review
	2000 New molecular entities = 2					
1	Triptorelin pamoate (Trelstar)	Verity	GnRH agonist	Advanced prostate cancer	06/15/2000	S
2	Arsenic trioxide (Trisenox)	Cephalon	Degradation of PML-RARα fusion protein	Acute promyelocytic leukemia characterized by the presence of the t (15; 17) translocation or PML/RARα gene expression	09/25/2000	P, O
	2000 Biologics license applications = 1					
1	Gemtuzumab ozogamicin (Mylotarg)	Wyeth Pharms	CD33-directed antibody-drug conjugate (ADC)	Relapsed or refractory CD33-positive acute myeloid leukemia (AML)	05/17/2000	O

	Drug (brand name)	Sponsor	Properties	Indication	Approval date	Review
	2001 New molecular entities = 2					
1	Imatinib mesylate (Gleevec)	Novartis	Tyrosine kinase inhibitor	Chronic myeloid leukemia	05/10/2001	P
2	Zoledronic acid (Zometa)	Novartis	Bone-resorption inhibitor	Hypercalcemia of malignancy	08/20/2001	P, O
	2001 Biologics license applications = 1					
1	Alemtuzumab (Campath)	Genzyme	CD52-directed mAb	B-cell chronic lymphocytic leukemia	05/07/2001	O

	Drug (brand name)	Sponsor	Properties	Indication	Approval date	Review
	2002 New molecular entities = 2					
1	Fulvestrant (Faslodex)	AstraZeneca	Estrogen receptor antagonist	HR-positive metastatic breast cancer	04/25/2002	S
2	Oxaliplatin (Eloxatin)	Sanofi	Organoplatinum alkylating agent, increases tumor response rate to chemotherapy	Colorectal cancer (in combination with 5-fluorouracil and leucovorin)	08/09/2002	P
	2002 Biologics license applications = 2					
1	Pegfilgrastim (Neulasta)	Amgen	PEGylated form of recombinant human granulocyte colony-stimulating factor (G-CSF)	Febrile neutropenia or myelosuppressive chemotherapy-induced neutropenia	01/31/2002	N/A
2	Ibritumomab tiuxetan (Zevalin)	IDEC Pharmas	^{111}In and ^{90}Y labelled CD20-specific radiotherapeutic mAb	B-cell non-Hodgkin's lymphoma	02/19/2002	O

	Drug (brand name)	Sponsor	Properties	Indication	Approval date	Review
	2003 New molecular entities = 5					
1	Aprepitant (Emend)	Merck & Co	NK1 receptor antagonist	Chemotherapy-induced nausea	03/26/2003	P
2	Gefitinib (Iressa)	AstraZeneca	EGFR kinase inhibitor	Non-small-cell lung cancer (NSCLC)	05/05/2003	P
3	Bortezomib (Velcade)	Takeda Pharms	Proteasome inhibitor	Multiple myeloma	05/13/2003	P, O
4	Palonosetron HCl (Aloxi)	Helsinn	5-HT_3 receptor antagonist	Chemotherapy-induced nausea	07/25/2003	S
5	Abarelix (Plenaxis)	Speciality European	GnRH receptor antagonist	Advanced prostate cancer	11/25/2003	P
	2003 Biologics license applications = 1					
1	Tositumomab and iodine I-131 tositumomab (Bexxar)	Corixa	CD20-directed radiotherapeutic mAb	Non-Hodgkin's lymphoma	06/30/2003	P, O

	Drug (brand name)	Sponsor	Properties	Indication	Approval date	Review
	2004 New molecular entities = 3					
1	Pemetrexed disodium (Alimta)	Eli Lilly	Folate analog	Malignant pleural mesothelioma	02/04/2004	P, O
2	Erlotinib HCl (Tarceva)	OSI Pharmas	EGFR inhibitor	Non-small-cell lung cancer (NSCLC)	11/18/2004	P
3	Clofarabine (Clolar)	Genzyme	Nucleoside antimetabolite, DNA synthesis inhibitor	Relapsed or refractory acute lymphoblastic leukemia	12/28/2004	P, O
	2004 Biologics license applications = 3					
1	Cetuximab (Erbitux)	ImClone	EGFR-directed mAb	Colorectal cancer	02/12/2004	N/A
2	Bevacizumab (Avastin)	Genentech	VEGF-A-directed mAb	Colorectal cancer	02/26/2004	O
3	Palifermin (Kepivance)	Biovitrum	Recombinant human keratinocyte growth factor	Oral mucositis following radiation or chemotherapy	12/15/2004	N/A

Supplementary data: Table 2 – FDA-approved cancer drugs (Continued)

	Drug (brand name)	Sponsor	Properties	Indication	Approval date	Review
	2005 New molecular entities = 2					
1	Nelarabine (Arranon)	Novartis	Purine nucleoside analog, inhibits the DNA elongation	T-cell acute lymphoblastic leukaemia and T-cell lymphoblastic lymphoma	10/28/2005	P, O
2	Sorafenib tosylate (Nexavar)	Bayer	Multi-target TKI (VEGFR2/3, PDGFRβ, FLT3, KIT, RAF1, and BRAF)	Advanced renal cell carcinoma	12/01/2005	P, O

	Drug (brand name)	Sponsor	Properties	Indication	Approval date	Review
	2006 New molecular entities = 3					
1	Sunitinib malate (Sutent)	CPPI CV	Multi-target TKI (VEGFRs, PDGFRα/β, CSF1R, KIT, and FLT3)	Imatinib-resistant gastrointestinal stromal tumor and advanced renal cell carcinoma	01/26/2006	P
2	Dasatinib (Sprycel)	Bristol-Myers Squibb	Tyrosine kinase inhibitor	Chronic myeloid leukemia	06/28/2006	P, O
3	Vorinostat (Zolinza)	Merck & Co	Histone deacetylase inhibitor	Cutaneous T-cell lymphoma	10/06/2006	P, O
	2006 Biologics license applications = 1					
1	Panitumumab (Vectibix)	Amgen	EGFR-directed mAb	EGFR-expressing metastatic colorectal carcinoma	09/27/2006	N/A

	Drug (brand name)	Sponsor	Properties	Indication	Approval date	Review
	2007 New molecular entities = 4					
1	Lapatinib Ditosylate (Tykerb)	Novartis	HER2 and EGFR inhibitor	Advanced or metastatic breast cancer (HER2-positive)	03/13/2007	P
2	Temsirolimus (Torisel)	Merck & Co	Mammalian target of rapamycin (mTOR) inhibitor	Advanced renal cell carcinoma	05/30/2007	P, O
3	Ixabepilone (Ixempra)	R-Pharm US	Microtubule-stabilizing agent	Advanced or metastatic breast cancer	10/16/2007	P
4	Nilotinib HCl (Tasigna)	Novartis	BCR-ABL kinase inhibitor	Chronic myelogenous leukemia	10/29/2007	S, O

	Drug (brand name)	Sponsor	Properties	Indication	Approval date	Review
	2008 New molecular entities = 5					
1	Fosaprepitant dimeglumine (Emend)	Merck & Co	Prodrug of aprepitant, NK1 receptor antagonist	Nausea and vomiting caused by chemotherapy	01/25/2008	S
2	Bendamustine HCl (Treanda)	Cephalon	Mechlorethamine derivative with DNA-alkylating activity	Chronic lymphocytic leukemia	03/20/2008	P, O
3	Iobenguane sulfate I-123 (AdreView)	GE Healthcare	Taken up by the noradrenaline transporter in adrenergic nerve terminals	Radiopharmaceutical agent, detection of primary or metastatic phaeochromocytoma or neuroblastoma	09/19/2008	P, O
4	Plerixafor (Mozobil)	Genzyme	CXCR4 antagonist	Autologous transplantation in patients with non-Hodgkin's lymphoma and multiple myeloma	12/15/2008	P, O
5	Degarelix acetate (Firmagon)	Ferring	GnRH receptor antagonist	Advanced prostate cancer	12/24/2008	S

	Drug (brand name)	Sponsor	Properties	Indication	Approval date	Review
	2009 New molecular entities = 4					
1	Everolimus (Afinitor)	Novartis	Mammalian target of rapamycin (mTOR) inhibitor	Advanced renal cell carcinoma	03/30/2009	P
2	Pralatrexate (Folotyn)	Acrotech	Folate analogue metabolic inhibitor that competitively inhibits dihydrofolate reductase	Peripheral T cell lymphoma	09/24/2009	P, O
3	Pazopanib HCl (Votrient)	Novartis	Multi-target TKI (VEGFRs, PDGFRα/β, FGFR1/2, KIT)	Advanced renal cell carcinoma	10/19/2009	S
4	Romidepsin (Istodax)	Celgene	Histone deacetylase inhibitor	Cutaneous T-cell lymphoma	11/05/2009	S
	2009 Biologics license applications = 1					
1	Ofatumumab (Arzerra)	Novartis	CD20-directed mAb	Chronic lymphocytic leukemia	10/26/2009	P, O

	Drug (brand name)	Sponsor	Properties	Indication	Approval date	Review
	2010 New molecular entities = 2					
1	Cabazitaxel (Jevtana kit)	Sanofi	Microtubule-stabilizing agent	Prostate cancer	06/17/2010	P
2	Eribulin mesylate (Halaven)	Eisai	Microtubule-destabilizing agent	Breast cancer	11/15/2010	P

Supplementary data: Table 2 – FDA-approved cancer drugs (Continued)

	Drug (brand name)	Sponsor	Properties	Indication	Approval date	Review
	2011 New molecular entities = 4					
1	Vandetanib (Caprelsa)	Genzyme	Multi-target TKI (VEGFR2/3, EGFR, and RET)	Unresectable or metastatic medullary thyroid cancer	04/06/2011	P, O
2	Abiraterone acetate (Zytiga)	Janssen Biotech	CYP17A1 inhibitor	Metastatic castration-resistant prostate cancer	04/28/2011	P
3	Vemurafenib (Zelboraf)	Roche	BRAF inhibitor	BRAF-positive unresectable or metastatic melanoma	08/17/2011	P, O
4	Crizotinib (Xalkori)	Merck & Co	Multi-target TKI (ALK, ROS1, and MET)	ALK-positive advanced or metastatic NSCLC	08/26/2011	P, O
	2011 Biologics license applications = 3					
1	Ipilimumab (Yervoy)	Bristol-Myers Squibb	Cytotoxic T-lymphocyte antigen 4 (CTLA-4)-directed mAb	Unresectable or metastatic melanoma	03/25/2011	P, O
2	Brentuximab vedotin (Adcetris)	Seattle Genetics	CD30-directed antibody drug conjugate (ADC)	Hodgkin’s lymphoma; systemic anaplastic large cell lymphoma	08/19/2011	P, O
3	Asparaginase Erwinia chrysanthemi (Erwinaze)	EUSA Pharma (USA)	*Erwinia chrysanthemi*-derived L-asparaginase	Acute lymphoblastic leukemia	11/18/2011	P, O

	Drug (brand name)	Sponsor	Properties	Indication	Approval date	Review
	2012 New molecular entities = 9					
1	Axitinib (Inlyta)	Pfizer	VEGFRs inhibitor	Advanced renal cell carcinoma	01/27/2012	S
2	Vismodegib (Erivedge)	Genentech	Smoothened (Hedgehog pathway) inhibitor	Metastatic or locally advanced basal cell carcinoma	01/30/2012	P
3	Carfilzomib (Kyprolis)	Onyx Therap	20S proteasome inhibitor	Multiple myeloma	07/20/2012	S, O
4	Enzalutamide (Xtandi)	Astellas	Androgen receptor inhibitor	Metastatic castration-resistant prostate cancer	08/31/2012	P
5	Bosutinib monohydrate (Bosulif)	Pfizer	BCR-ABL inhibitor; SRC family kinase inhibitor	Chronic, accelerated or blast phase Ph+ chronic myelogenous leukemia	09/04/2012	S, O
6	Regorafenib (Stivarga)	Bayer	Multi-target TKI (RET, VEGFRs, KIT, PDGFRα/β, FGFR1/2, RAF1, $BRAF^{WT}$, and $BRAF^{V600E}$)	Metastatic colorectal cancer	09/27/2012	P
7	Omacetaxine mepesuccinate (Synribo)	Teva	Cephalotaxine, inhibits protein chain elongation by binding the large ribosomal subunit	Chronic or accelerated-phase chronic myeloid leukemia	10/26/2012	S, O
8	Cabozantinib S-malate (Cometriq)	Exelixis	Multi-target TKI (VEGFRs, MET, RET, FLT3, KIT, TIE2, and AXL)	Progressive, metastatic medullary thyroid cancer	11/29/2012	P, O
9	Ponatinib HCl (Iclusig)	Takeda	Multi-kinase inhibitor (BCR-ABL, KIT, RET and FLT3)	Chronic, accelerated or blast-phase chronic myeloid leukemia	12/14/2012	P, O
	2012 Biologics license applications = 3					
1	Pertuzumab (Perjeta)	Genentech	HER2-directed mAb	HER2-positive metastatic breast cancer	06/08/2012	P
2	Ziv-aflibercept (Zaltrap)	Sanofi	Soluble receptor decoy that binds VEGF-A, VEGF-B, and PlGF	Metastatic colorectal cancer	08/03/2012	P
3	Tbo-Filgrastim (Neutroval)	Sicor Biotech	Methionyl human G-CSF	Severe neutropaenia in patients with non-myeloid malignancies	08/29/2012	S

	Drug (brand name)	Sponsor	Properties	Indication	Approval date	Review
	2013 New molecular entities = 7					
1	Pomalidomide (Pomalyst)	Celgene	Thalidomide analog, immunomodulatory antineoplastic agent	Multiple myeloma	02/08/2013	S, O
2	Technetium TC 99m tilmanocept (Lymphoseek kit)	Cardinal Health 414	^{99m}Tc labelled radioactive diagnostic agent	Lymphatic mapping in patients with breast cancer or melanoma	03/13/2013	S
3	Radium Ra-223 dichloride (Xofigo)	Bayer	α-particle-emitting radioactive therapeutic	Castration-resistant prostate cancer	05/15/2013	P
4	Dabrafenib mesylate (Tafinlar)	Novartis	Kinase inhibitor with activity against $BRAF^{V600E/D/K}$, wild-type BRAF and other kinases	Unresectable or metastatic melanoma with *BRAF*^{V600E} mutation	05/29/2013	S, O
5	Trametinib dimethyl sulfoxide (Mekinist)	Novartis	MEK1 and MEK2 kinase inhibitor	Unresectable or metastatic melanoma with *BRAF*^{V600E} mutation	05/29/2013	S, O
6	Afatinib dimaleate (Gilotrif)	Boehringer Ingelheim	EGFR, HER2, and HER4 inhibitor	Metastatic NSCLC with *EGFR* exon 19 deletions or exon 21 (L858R) substitution mutations	07/12/2013	P, O
7	Ibrutinib (Imbruvica)	Pharmacyclics	Bruton’s tyrosine kinase inhibitor	Mantle cell lymphoma	11/13/2013	P, O
	2013 Biologics license applications = 2					
1	Ado-trastuzumab emtansine (Kadcyla)	Genentech	HER2-directed ADC	HER2-positive metastatic breast cancer	02/22/2013	P
2	Obinutuzumab (Gazyva)	Genentech	CD20-directed mAb	Previously untreated chronic lymphocytic leukemia	11/01/2013	P, O

Supplementary data: Table 2 – FDA-approved cancer drugs (Continued)

	Drug (brand name)	Sponsor	Properties	Indication	Approval date	Review
	2014 New molecular entities = 5					
1	Ceritinib (Zykadia)	Novartis	Multi-target TKI (ALK, IGF1R, INSR, and ROS1)	ALK-positive metastatic NSCLC	04/29/2014	P, O
2	Belinostat (Beleodaq)	Acrotech	Histone-deacetylase inhibitor	Peripheral T-cell lymphoma	07/03/2014	P, O
3	Idelalisib (Zydelig)	Gilead Sciences	PI3Kδ inhibitor	Chronic lymphocytic leukaemia (CLL), B-cell non-Hodgkin's lymphoma, and small lymphocytic lymphoma (SLL)	07/23/2014	P, O
4	Netupitant; Palonosetron HCl (Akynzeo)	Helsinn Hlthcare	NK1-receptor antagonist plus 5-HT3 receptor antagonist	Chemotherapy-related nausea	10/10/2014	S
5	Olaparib (Lynparza)	AstraZeneca	PARP inhibitor	Advanced *BRCA*-mutated ovarian cancer	12/19/2014	P, O
	2014 Biologics license applications = 4					
1	Ramucirumab (Cyramza)	Eli Lilly & Co	VEGFR2-directed mAb	Gastric cancer	04/21/2014	P, O
2	Pembrolizumab (Keytruda)	Merck & Co	PD1-directed mAb	Metastatic melanoma	09/04/2014	P, O
3	Blinatumomab (Blincyto)	Amgen	CD19- and CD3-bispecific mAb	B-cell precursor acute lymphoblastic leukemia (ALL)	12/03/2014	P, O
4	Nivolumab (Opdivo)	Bristol-Myers Squibb	PD1-directed mAb	Unresectable or metastatic melanoma	12/22/2014	P, O

	Drug (brand name)	Sponsor	Properties	Indication	Approval date	Review
	2015 New molecular entities = 11					
1	Palbociclib (Ibrance)	Pfizer	Cyclin-dependent kinase 4/6 (CDK4/6) inhibitor	HER2-negative and HR-positive advanced or metastatic breast cancer	02/03/2015	P
2	Lenvatinib mesylate (Lenvima)	Eisai	Multi-target TKI (VEGFRs, FGFRs, PDGFRα, RET, and KIT)	Thyroid cancer	02/13/2015	P, O
3	Panobinostat lactate (Farydak)	Secura	Histone deacetylase inhibitor	Multiple myeloma	02/23/2015	P, O
4	Sonidegib phosphate (Odomzo)	Sun Pharma Global	Smoothened inhibitor	Basal cell carcinoma	07/24/2015	S
5	Rolapitant HCl (Varubi)	Tersera	Neurokinin 1 receptor antagonist	Nausea and vomiting associated with chemotherapy	09/01/2015	S
6	Tipiracil HCl; Trifluridine (Lonsurf)	Taiho Oncology	Thymidine phosphorylase inhibitor plus a nucleoside metabolic inhibitor	Colorectal cancer	09/22/2015	S
7	Trabectedin (Yondelis)	Janssen Prods	Alkylating drug, interacts with the minor groove of DNA	Liposarcoma or leiomyosarcoma	10/23/2015	P, O
8	Cobimetinib fumarate (Cotellic)	Genentech	MEK inhibitor	Melanoma with *BRAF*$^{V600E/K}$ mutations	11/10/2015	P, O
9	Osimertinib mesylate (Tagrisso)	AstraZeneca	EGFR inhibitor	NSCLC with *EGFR*^{T790M} mutations	11/13/2015	P, O
10	Ixazomib citrate (Ninlaro)	Takeda	Oral proteasome inhibitor	Multiple myeloma	11/20/2015	P, O
11	Alectinib HCl (Alecensa)	Roche	ALK inhibitor	Non-small-cell lung cancer (NSCLC)	12/11/2015	P, O
	2015 Biologics license applications = 4					
1	Dinutuximab (Unituxin)	United Therap	GD2-directed mAb	High-risk neuroblastoma	03/10/2015	P, O
2	Daratumumab (Darzalex)	Janssen Biotech	CD38-directed mAb	Multiple myeloma	11/16/2015	P, O
3	Necitumumab (Portrazza)	Eli Lilly & Co	EGFR-directed mAb	Non-small-cell lung cancer (NSCLC)	11/24/2015	S, O
4	Elotuzumab (Empliciti)	Bristol-Myers Squibb	SLAMF7-directed mAb	Multiple myeloma	11/30/2015	P, O

	Drug (brand name)	Sponsor	Properties	Indication	Approval date	Review
	2016 New molecular entities = 4					
1	Venetoclax (Venclexta)	AbbVie	BCL-2 inhibitor	Chronic lymphocytic leukemia	04/11/2016	P, O
2	Fluciclovine F-18 (Axumin)	Blue Earth	^{18}F labelled radioactive diagnostic agent	Imaging diagnosis of suspected prostate cancer recurrence	05/27/2016	P
3	Gallium dotatate Ga-68 (Netspot)	AAA USA Inc	^{68}Ga labelled radioactive diagnostic	Imaging diagnosis of neuroendocrine tumors	06/01/2016	P, O
4	Rucaparib camsylate (Rubraca)	Clovis Oncology	PARP inhibitor	BRCA-positive ovarian cancer	12/19/2016	P, O
	2016 Biologics license applications = 2					
1	Atezolizumab (Tecentriq)	Genentech	PDL1-directed mAb	Advanced or metastatic urothelial carcinoma	05/18/2016	P
2	Olaratumab (Lartruvo)	Eli Lilly & Co	PDGFRα-directed mAb	Soft tissue sarcoma	10/19/2016	P, O

Supplementary data: Table 2 – FDA-approved cancer drugs (Continued)

	Drug (brand name)	Sponsor	Properties	Indication	Approval date	Review
	2017 New molecular entities = 9					
1	Ribociclib succinate (Kisqali)	Novartis	CDK4/6 inhibitor	HR-positive, HER2-negative breast cancer	03/13/2017	P
2	Niraparib tosylate (Zejula)	GlaxoSmithKline	Poly-ADP ribose polymerase (PARP) inhibitor	Epithelial ovarian, fallopian tube or primary peritoneal cancer	03/27/2017	P, O
3	Brigatinib (Alunbrig)	Takeda	Multi-target TKI (ALK, EGFR, IGF1R, FLT3, and ROS1)	ALK-positive NSCLC	04/28/2017	P, O
4	Midostaurin (Rydapt)	Novartis	Fms-like tyrosine kinase 3 (FLT3) inhibitor	FLT3-positive acute myeloid leukemia (AML)	04/28/2017	P, O
5	Neratinib maleate (Nerlynx)	Puma Biotech	EGFR, HER2, and HER4 inhibitor	HER2-overexpressed breast cancer	07/17/2017	S
6	Enasidenib mesylate (Idhifa)	Celgene Corp	IDH2 inhibitor	*IDH2*-mutated acute myeloid leukemia (AML)	08/01/2017	P, O
7	Copanlisib 2HCl (Aliqopa)	Bayer	PI3Kα/δ inhibitor	Follicular lymphoma (FL)	09/14/2017	P, O
8	Abemaciclib (Verzenio)	Eli Lilly & Co	CDK4/6 inhibitor	HR-positive, HER2-negative breast cancer	09/28/2017	P
9	Acalabrutinib (Calquence)	AstraZeneca	BTK inhibitor	Mantle cell lymphoma	10/31/2017	P, O
	2017 Biologics license applications = 3					
1	Avelumab (Bavencio)	Emd Serono	PDL1-directed mAb	Merkel cell carcinoma	03/23/2017	P, O
2	Durvalumab (Imfinzi)	AstraZeneca	PDL1-directed mAb	Urothelial carcinoma	05/01/2017	P
3	Inotuzumab ozogamicin (Besponsa)	Wyeth Pharms	CD22-directed ADC	B cell precursor acute lymphoblastic leukemia (ALL)	08/17/2017	P, O

	Drug (brand name)	Sponsor	Properties	Indication	Approval date	Review
	2018 New molecular entities = 13					
1	Lutetium dotatate Lu-177 (Lutathera)	AAA USA Inc	Somatostatin receptor-targeted radiopharmaceutical	Gastroenteropancreatic neuroendocrine tumors (GEP-NETs)	01/26/2018	P, O
2	Apalutamide (Erleada)	Janssen Biotech	Androgen receptor inhibitor	Prostate cancer	02/14/2018	P
3	Fosnetupitant chloride HCl; Palonosetron HCl (Akynzeo IV)	Helsinn Group	Neurokinin 1 (NK1) receptor antagonist plus 5-HT_3 receptor antagonist	Chemotherapy-induced nausea	04/19/2018	S
4	Encorafenib (Braftovi)	Array BioPharma	BRAF inhibitor	*BRAF*-mutated melanoma	06/27/2018	S, O
5	Binimetinib (Mektovi)	Array BioPharma	MEK inhibitor	*BRAF*-mutated melanoma	06/27/2018	S, O
6	Ivosidenib (Tibsovo)	Servier	Isocitrate dehydrogenase-1 (IDH1) inhibitor	*IDH1*-mutated acute myeloid leukemia (AML)	07/20/2018	P, O
7	Duvelisib (Copiktra)	Secura	Phosphatidylinositol 3-kinase δ/γ (PI3Kδ/γ) inhibitor	Chronic lymphocytic leukaemia (CLL), follicular lymphoma (FL), and small lymphocytic lymphoma (SLL)	09/24/2018	P, O
8	Dacomitinib (Vizimpro)	Pfizer	EGFR inhibitor	*EGFR*-mutated NSCLC	09/27/2018	P, O
9	Talazoparib tosylate (Talzenna)	Pfizer	Poly-ADP ribose polymerase (PARP) inhibitor	*BRCA*-mutated HER2-negative breast cancer	10/16/2018	P
10	Lorlatinib (Lorbrena)	Pfizer	Multi-target TKI (ALK and ROS1)	ALK-positive NSCLC	11/02/2018	P, O
11	Glasdegib maleate (Daurismo)	Pfizer	Smoothened (Hedgehog pathway) inhibitor	Acute myeloid leukemia (AML)	11/21/2018	P, O
12	Larotrectinib sulfate (Vitrakvi)	Bayer	TRKs inhibitor	NTRK-positive solid cancers	11/26/2018	P, O
13	Gilteritinib fumarate (Xospata)	Astellas	Fms-like tyrosine kinase 3 (FLT3) inhibitor	FLT3-positive AML	11/28/2018	P, O
	2018 Biologics license applications = 4					
1	Moxetumomab pasudotox (Lumoxiti)	Innate Pharma	CD22-directed ADC	Hairy cell leukemia	09/13/2018	P, O
2	Cemiplimab (Libtayo)	Regeneron Pharmaceuticals	Programmed death receptor-1 (PD1)-directed mAb	Metastatic or locally advanced cutaneous squamous cell carcinoma (CSCC)	09/28/2018	P
3	Calaspargase pegol (Asparlas)	Servier Pharma	Asparagine specific enzyme	Acute lymphoblastic leukemia (ALL)	12/20/2018	S, O
4	Tagraxofusp (Elzonris)	Stemline Therapeutics	IL-3 and diphtheria toxin fusion protein	Blastic plasmacytoid dendritic cell neoplasm	12/21/2018	P, O

Supplementary data: Table 2 – FDA-approved cancer drugs (Continued)

	Drug (brand name)	Sponsor	Properties	Indication	Approval date	Review
	2019 New molecular entities = 8					
1	Erdafitinib (Balversa)	Janssen Biotech	Fibroblast growth factor receptor (FGFR) inhibitor	Bladder cancer	04/12/2019	P
2	Alpelisib (Piqray)	Novartis	Phosphatidylinositol 3-kinase α (PI3Kα) inhibitor	Breast cancer	05/24/2019	P
3	Selinexor (Xpovio)	Karyopharm Theraps	Exportin 1 (XPO1) inhibitor	Multiple myeloma	07/03/2019	P, O
4	Darolutamide (Nubeqa)	Bayer	Androgen receptor inhibitor	Prostate cancer	07/30/2019	P
5	Pexidartinib HCl (Turalio)	Daiichi Sankyo	CSF1R, KIT, and FLT3 inhibitor	Tenosynovial giant cell tumor	08/02/2019	P, O
6	Entrectinib (Rozlytrek)	Genentech	Multi-target TKI (TRKs, ROS1, and ALK)	NTRK fusion- positive solid tumors and ROS1-positive NSCLC	08/15/2019	P, O
7	Gallium dotatoc Ga-68	UIHC PET Imaging	^{68}Ga labelled radioactive diagnostic agent	Localization of somatostatin receptor-positive neuroendocrine tumors	08/21/2019	S, O
8	Zanubrutinib (Brukinsa)	BeiGene	Bruton's tyrosine kinase (BTK) inhibitor	Mantle cell lymphoma	11/14/2019	P, O
	2019 Biologics license applications = 3					
1	Polatuzumab vedotin (Polivy)	Genentech	CD79b-directed antibody-drug conjugate (ADC)	Diffuse large B-cell lymphoma	06/10/2019	P, O
2	Enfortumab vedotin (Padcev)	Astellas	Nectin-4-directed antibody-drug conjugate (ADC)	Urothelial carcinoma	12/18/2019	P
3	Trastuzumab deruxtecan (Enhertu)	Daiichi Sankyo	HER2-directed antibody-drug conjugate (ADC)	HER2-positive breast cancer	12/20/2019	P

	Drug (brand name)	Sponsor	Properties	Indication	Approval date	Review
	2020 New molecular entities = 13					
1	Avapritinib (Ayvakit)	Blueprint Medicines	PDGFRα, PDGFRα mutants, and KIT inhibitor	Gastrointestinal stromal tumor (GIST) with *PDGFRA* exon 18 mutations	01/09/2020	P, O
2	Tazemetostat (Tazverik)	Epizyme Inc	Enhancer of Zeste Homolog 2 (EZH2) inhibitor	Epithelioid sarcoma	01/23/2020	P, O
3	Tucatinib (Tukysa)	Seagen	HER2 inhibitor	HER2-positive breast cancer	04/17/2020	P, O
4	Pemigatinib (Pemazyre)	Incyte	Fibroblast growth factor receptor 1-3 (FGFR1-3) kinase inhibitor	Advanced cholangiocarcinoma with *FGFR2* fusions/rearrangements	04/17/2020	P, O
5	Capmatinib HCl (Tabrecta)	Novartis	MET kinase inhibitor	Non-small-cell lung cancer (NSCLC)	05/06/2020	P, O
6	Selpercatinib (Retevmo)	Loxo Oncology	RET kinase inhibitor	RET fusion-positive NSCLC and thyroid cancer	05/08/2020	P, O
7	Ripretinib (Qinlock)	Deciphera	PDGFRα, PDGFRα mutants, and KIT inhibitor	Advanced gastrointestinal stromal tumor (GIST)	05/15/2020	P, O
8	Fluoroestradiol F-18 (Cerianna)	Zionexa	^{18}F labelled radioactive diagnostic agent	Imaging, breast cancer	05/20/2020	S
9	Lurbinectedin (Zepzelca)	Jazz	DNA alkylating drug	Small-cell lung cancer	06/15/2020	P, O
10	Copper dotatate Cu-64 (Detectnet)	Radiomedix	^{64}Cu labelled somatostatin analog	Imaging, cancer	09/03/2020	P, O
11	Pralsetinib (Gavreto)	Genentech	RET kinase inhibitor	RET fusion-positive NSCLC	09/04/2020	P, O
12	PSMA-11 Ga-68	UCLA	^{68}Ga labelled radioactive diagnostic agent	Imaging, prostate cancer	12/01/2020	S
13	Relugolix (Orgovyx)	Myovant Sciences	GnRH receptor antagonist	Prostate cancer	12/18/2020	P
	2020 Biologics license applications = 6					
1	Isatuximab (Sarclisa)	Sanofi	CD38-directed mAb	Multiple myeloma	03/02/2020	S, O
2	Sacituzumab govitecan (Trodelvy)	Immunomedics Inc	Trophoblast cell surface antigen 2 (Trop2)-directed ADC	Triple-negative breast cancer	04/22/2020	P
3	Tafasitamab (Monjuvi)	MorphoSys	CD19-directed mAb	Diffuse large B-cell lymphoma (DLBCL)	07/31/2020	P, O
4	Belantamab mafodotin (Blenrep)	GlaxoSmithKline	B-cell maturation antigen (BCMA)-directed ADC	Multiple myeloma	08/05/2020	P, O
5	Naxitamab (Danyelza)	Y-mAbs Therapeutics	GD2-directed mAb	High-risk neuroblastoma	11/25/2020	P, O
6	Margetuximab (Margenza)	MacroGenics	HER2-directed mAb	HER2-positive breast cancer	12/16/2020	S

Supplementary data: Table 2 – FDA-approved cancer drugs (Continued)

	Drug (brand name)	Sponsor	Properties	Indication	Approval date	Review
	2021 New molecular entities = 11					
1	Tepotinib HCl (Tepmetko)	Emd Serono	MET kinase inhibitor	Non-small-cell lung cancer (NSCLC)	02/03/2021	P, O
2	Umbralisib tosylate (Ukoniq)	TG Theraps	PI3Kδ and CK1ε inhibitor	Relapsed or refractory marginal zone lymphoma (MZL), follicular lymphoma	02/05/2021	P, O
3	Trilaciclib 2HCl (Cosela)	G1 Therap	CDK4/6 kinase inhibitor	Chemotherapy-induced myelosuppression	02/12/2021	P
4	Melphalan flufenamide HCl (Pepaxto)	Oncopeptides	Peptide-conjugated alkylating drug	Multiple myeloma	02/26/2021	P, O
5	Tivozanib HCl (Fotivda)	Aveo Pharms	VEGFRs inhibitor	Advanced renal cell carcinoma	03/10/2021	S
6	Piflufolastat F-18 (Pylarify)	Progenics	^{18}F labelled prostate-specific membrane antigen (PSMA) imaging agent	Prostate cancer imaging	05/26/2021	P
7	Infigratinib phosphate (Truseltiq)	Helsinn Hlthcare	FGFR1-3 inhibitor	Advanced cholangiocarcinoma with *FGFR2* fusions/rearrangements	05/28/2021	P, O
8	Sotorasib (Lumakras)	Amgen	$KRAS^{G12C}$ inhibitor	*KRAS*^{G12C}-mutated NSCLC	05/28/2021	P, O
9	Mobocertinib succinate (Exkivity)	Takeda	EGFR kinase inhibitor	*EGFR* exon 20-mutated NSCLC	09/15/2021	P, O
10	Asciminib HCl (Scemblix)	Novartis	ABL/BCR-ABL1 kinase inhibitor	Philadelphia chromosome-positive chronic myeloid leukemia (Ph+ CML)	10/29/2021	P, O
11	Pafolacianine sodium (Cytalux)	On Target Labs	Fluorescent folate receptor α (FRα) imaging agent	Ovarian cancer imaging	11/29/2021	P, O
	2021 Biologics license applications = 5					
1	Dostarlimab (Jemperli)	GlaxoSmithKline	PD1-directed mAb	Endometrial cancer	04/22/2021	P
2	Loncastuximab tesirine (Zynlonta)	ADC Therapeutics	CD19-directed ADC	B-cell lymphoma	04/23/2021	P, O
3	Amivantamab (Rybrevant)	Janssen Biotech	EGFR- and MET-bispecific antibody	*EGFR* exon 20-mutated NSCLC	05/21/2021	P
4	Asparaginase erwinia chrysanthemi (Rylaze)	Jazz	Recombinant asparagine-specific enzyme	Acute lymphoblastic leukaemia (ALL), and lymphoblastic lymphoma (LBL), in patients allergic to *E. coli*-derived products	06/30/2021	S, O
5	Tisotumab vedotin (Tivdak)	Seagen	Tissue-factor-targeted ADC	Cervical cancer	09/20/2021	P, O

Supplementary data: Table 3 – FDA-approved therapeutic drugs for solid tumors

	Drug (brand name)	Sponsor	Properties	Indication	Approval date	Review
	Therapeutic drugs for lung cancers					
1	Vinorelbine tartrate (Navelbine)	Pierre Fabre	Microtubule-destabilizing agent	Non-small-cell lung cancer (NSCLC)	12/23/1994	P
2	Gefitinib (Iressa)	AstraZeneca	EGFR kinase inhibitor	Non-small-cell lung cancer (NSCLC)	05/05/2003	P
3	Erlotinib HCl (Tarceva)	OSI Pharmas	EGFR inhibitor	Non-small-cell lung cancer (NSCLC)	11/18/2004	P
4	Crizotinib (Xalkori)	Merck & Co	Multi-target TKI (ALK, ROS1, and MET)	ALK-positive advanced or metastatic NSCLC	08/26/2011	P, O
5	Afatinib dimaleate (Gilotrif)	Boehringer Ingelheim	EGFR, HER2, and HER4 inhibitor	Metastatic NSCLC with *EGFR* exon 19 deletions or exon 21 (L858R) substitution mutations	07/12/2013	P, O
6	Ceritinib (Zykadia)	Novartis	Multi-target TKI (ALK, IGF1R, INSR, and ROS1)	ALK-positive metastatic NSCLC	04/29/2014	P, O
7	Osimertinib mesylate (Tagrisso)	AstraZeneca	EGFR inhibitor	NSCLC with *EGFR*^{T790M} mutations	11/13/2015	P, O
8	Necitumumab (Portrazza)	Eli Lilly & Co	EGFR-directed mAb	Non-small-cell lung cancer (NSCLC)	11/24/2015	S, O
9	Alectinib HCl (Alecensa)	Roche	ALK inhibitor	Non-small-cell lung cancer (NSCLC)	12/11/2015	P, O
10	Brigatinib (Alunbrig)	Takeda	Multi-target TKI (ALK, EGFR, IGF1R, FLT3, and ROS1)	ALK-positive NSCLC	04/28/2017	P, O
11	Dacomitinib (Vizimpro)	Pfizer	EGFR inhibitor	*EGFR*-mutated NSCLC	09/27/2018	P, O
12	Lorlatinib (Lorbrena)	Pfizer	Multi-target TKI (ALK and ROS1)	ALK-positive NSCLC	11/02/2018	P, O
13	Entrectinib (Rozlytrek)	Genentech	Multi-target TKI (TRKs, ROS1, and ALK)	NTRK fusion- positive solid tumors and ROS1-positive NSCLC	08/15/2019	P, O
14	Capmatinib HCl (Tabrecta)	Novartis	MET kinase inhibitor	Non-small-cell lung cancer (NSCLC)	05/06/2020	P, O
15	Selpercatinib (Retevmo)	Loxo Oncology	RET kinase inhibitor	RET fusion-positive NSCLC and thyroid cancer	05/08/2020	P, O
16	Pralsetinib (Gavreto)	Genentech	RET kinase inhibitor	RET fusion-positive NSCLC	09/04/2020	P, O
17	Tepotinib HCl (Tepmetko)	Emd Serono	MET kinase inhibitor	Non-small-cell lung cancer (NSCLC)	02/03/2021	P, O
18	Amivantamab (Rybrevant)	Janssen Biotech	EGFR- and MET-bispecific antibody	*EGFR* exon 20-mutated NSCLC	05/21/2021	P
19	Sotorasib (Lumakras)	Amgen	KRAS^{G12C} inhibitor	*KRAS*^{G12C}-mutated NSCLC	05/28/2021	P, O
20	Mobocertinib succinate (Exkivity)	Takeda	EGFR kinase inhibitor	*EGFR* exon 20-mutated NSCLC	09/15/2021	P, O
21	Topotecan HCl (Hycamtin)	Novartis	DNA topoisomerases inhibitor	Relapsed small cell lung cancer	05/28/1996	P
22	Lurbinectedin (Zepzelca)	Jazz	DNA alkylating drug	Small-cell lung cancer	06/15/2020	P, O

Supplementary data: Table 3 – FDA-approved therapeutic drugs for solid tumors (Continued)

	Drug (brand name)	Sponsor	Properties	Indication	Approval date	Review
	Therapeutic drugs for breast cancers					
1	Anastrozole (Arimidex)	Ani Pharms	Aromatase inhibitor	Advanced breast cancer in post-menopausal women	12/27/1995	S
2	Docetaxel (Taxotere)	Sanofi	Microtubule-stabilizing agent	Locally advanced or metastatic breast cancer	05/14/1996	P
3	Toremifene citrate (Fareston)	Kyowa kirin	Estrogen receptor (ER) inhibitor	ER-positive metastatic breast cancer	05/29/1997	S, O
4	Letrozole (Femara)	Novartis	Aromatase inhibitor	Advanced breast cancer	07/25/1997	S
5	Capecitabine (Xeloda)	Roche	Prodrug of 5-FU	Metastatic breast cancer	04/30/1998	P
6	Trastuzumab (Herceptin)	Genentech	HER2-directed mAb	HER2-positive breast cancer	09/25/1998	P
7	Epirubicin HCl (Ellence)	Pfizer	DNA topoisomerase II inhibitor	Primary breast cancer with axillary node tumor involvement	09/15/1999	P, O
8	Exemestane (Aromasin)	Pfizer	Aromatase inhibitor	ER-positive early breast cancer	10/21/1999	S, O
9	Fulvestrant (Faslodex)	AstraZeneca	Estrogen receptor antagonist	HR-positive metastatic breast cancer	04/25/2002	S
10	Lapatinib Ditosylate (Tykerb)	Novartis	HER2 and EGFR inhibitor	Advanced or metastatic breast cancer (HER2-positive)	03/13/2007	P
11	Ixabepilone (Ixempra)	R-Pharm US	Microtubule-stabilizing agent	Advanced or metastatic breast cancer	10/16/2007	P
12	Eribulin mesylate (Halaven)	Eisai	Microtubule-destabilizing agent	Breast cancer	11/15/2010	P
13	Pertuzumab (Perjeta)	Genentech	HER2-directed mAb	HER2-positive metastatic breast cancer	06/08/2012	P
14	Ado-trastuzumab emtansine (Kadcyla)	Genentech	HER2-directed ADC	HER2-positive metastatic breast cancer	02/22/2013	P
15	Palbociclib (Ibrance)	Pfizer	Cyclin-dependent kinase 4/6 (CDK4/6) inhibitor	HER2-negative and HR-positive advanced or metastatic breast cancer	02/03/2015	P
16	Ribociclib succinate (Kisqali)	Novartis	CDK4/6 inhibitor	HR-positive, HER2-negative breast cancer	03/13/2017	P
17	Neratinib maleate (Nerlynx)	Puma Biotech	EGFR, HER2, and HER4 inhibitor	HER2-overexpressed breast cancer	07/17/2017	S
18	Abemaciclib (Verzenio)	Eli Lilly & Co	CDK4/6 inhibitor	HR-positive, HER2-negative breast cancer	09/28/2017	P
19	Talazoparib tosylate (Talzenna)	Pfizer	Poly-ADP ribose polymerase (PARP) inhibitor	*BRCA*-mutated HER2-negative breast cancer	10/16/2018	P
20	Alpelisib (Piqray)	Novartis	Phosphatidylinositol 3-kinase α (PI3Kα) inhibitor	Breast cancer	05/24/2019	P
21	Trastuzumab deruxtecan (Enhertu)	Daiichi Sankyo	HER2-directed antibody-drug conjugate (ADC)	HER2-positive breast cancer	12/20/2019	P
22	Tucatinib (Tukysa)	Seagen	HER2 inhibitor	HER2-positive breast cancer	04/17/2020	P, O
23	Sacituzumab govitecan (Trodelvy)	Immunomedics Inc	Trophoblast cell surface antigen 2 (Trop2)-directed ADC	Triple-negative breast cancer	04/22/2020	P
24	Margetuximab (Margenza)	MacroGenics	HER2-directed mAb	HER2-positive breast cancer	12/16/2020	S

	Drug (brand name)	Sponsor	Properties	Indication	Approval date	Review
	Therapeutic drugs for gynecologic cancers					
1	Paclitaxel (Taxol)	HQ Spclt Pharma	Microtubule-stabilizing agent	Advanced ovarian cancer	12/29/1992	P, O
2	Olaparib (Lynparza)	AstraZeneca	PARP inhibitor	Advanced *BRCA*-mutated ovarian cancer	12/19/2014	P, O
3	Rucaparib camsylate (Rubraca)	Clovis Oncology	PARP inhibitor	BRCA-positive ovarian cancer	12/19/2016	P, O
4	Niraparib tosylate (Zejula)	GlaxoSmithKline	Poly-ADP ribose polymerase (PARP) inhibitor	Epithelial ovarian, fallopian tube or primary peritoneal cancer	03/27/2017	P, O
5	Dostarlimab (Jemperli)	GlaxoSmithKline	PD1-directed mAb	Endometrial cancer	04/22/2021	P
6	Tisotumab vedotin (Tivdak)	Seagen	Tissue-factor-targeted ADC	Cervical cancer	09/20/2021	P, O

Supplementary data: Table 3 – FDA-approved therapeutic drugs for solid tumors (Continued)

	Drug (brand name)	Sponsor	Properties	Indication	Approval date	Review
	Therapeutic drugs for gastrointestinal cancers					
1	Porfimer sodium (Photofrin)	Pinnacle Biolgs	A photosensitizer used for photodynamic therapy	Advanced esophageal cancer	12/27/1995	P, O
2	Ramucirumab (Cyramza)	Eli Lilly & Co	VEGFR2-targeted mAb	Gastric cancer	04/21/2014	P, O
3	Sunitinib malate (Sutent)	CPPI CV	Multi-target TKI (VEGFRs, PDGFRα/β, CSF1R, KIT, and FLT3)	Imatinib-resistant gastrointestinal stromal tumor and advanced renal cell carcinoma	01/26/2006	P
4	Avapritinib (Ayvakit)	Blueprint Medicines	PDGFRα, PDGFRα mutants, and KIT inhibitor	Gastrointestinal stromal tumor (GIST) with *PDGFRA* exon 18 mutations	01/09/2020	P, O
5	Ripretinib (Qinlock)	Deciphera	PDGFRα, PDGFRα mutants, and KIT inhibitor	Advanced gastrointestinal stromal tumor (GIST)	05/15/2020	P, O
6	Lutetium dotatate Lu-177 (Lutathera)	AAA USA Inc	Somatostatin receptor-targeted radiopharmaceutical	Gastroenteropancreatic neuroendocrine tumors (GEP-NETs)	01/26/2018	P, O
7	Pemigatinib (Pemazyre)	Incyte	Fibroblast growth factor receptor 1-3 (FGFR1-3) kinase inhibitor	Advanced cholangiocarcinoma with *FGFR2* fusions/rearrangements	04/17/2020	P, O
8	Infigratinib phosphate (Truseltiq)	Helsinn Hlthcare	FGFR1-3 inhibitor	Advanced cholangiocarcinoma with *FGFR2* fusions/rearrangements	05/28/2021	P, O
9	Gemcitabine HCl (Gemzar)	Eli Lilly	DNA synthesis inhibitor	Locally advanced or metastatic pancreatic cancer	05/15/1996	P
10	Irinotecan HCl (Camptosar)	Pfizer	Topoisomerase I inhibitor	Metastatic colorectal cancer	06/14/1996	P
11	Oxaliplatin (Eloxatin)	Sanofi	Organoplatinum alkylating agent, increases tumor response rate to chemotherapy	Colorectal cancer (in combination with 5-FU and leucovorin)	08/09/2002	P
12	Cetuximab (Erbitux)	ImClone	EGFR-directed mAb	Colorectal cancer	02/12/2004	N/A
13	Bevacizumab (Avastin)	Genentech	VEGF-A-directed mAb	Colorectal cancer	02/26/2004	O
14	Panitumumab (Vectibix)	Amgen	EGFR-directed mAb	EGFR-expressing metastatic colorectal carcinoma	09/27/2006	N/A
15	Ziv-aflibercept (Zaltrap)	Sanofi	Soluble receptor decoy that binds VEGF-A, VEGF-B, and PlGF	Metastatic colorectal cancer	08/03/2012	P
16	Regorafenib (Stivarga)	Bayer	Multi-target TKI (RET, VEGFRs, KIT, PDGFRα/β, FGFR1/2, RAF1, $BRAF^{WT}$, and $BRAF^{V600E}$)	Metastatic colorectal cancer	09/27/2012	P
17	Tipiracil HCl; Trifluridine (Lonsurf)	Taiho Oncology	Thymidine phosphorylase inhibitor plus a nucleoside metabolic inhibitor	Colorectal cancer	09/22/2015	S

Supplementary data: Table 3 – FDA-approved therapeutic drugs for solid tumors (Continued)

	Drug (brand name)	Sponsor	Properties	Indication	Approval date	Review
	Therapeutic drugs for prostate cancers					
1	Bicalutamide (Casodex)	Ani Pharms	Androgen receptor antagonist	Advanced prostate cancer	10/04/1995	S
2	Nilutamide (Nilandron)	Concordia	Androgen receptor antagonist	Metastatic prostate cancer	09/19/1996	S
3	Triptorelin pamoate (Trelstar)	Verity	GnRH agonist	Advanced prostate cancer	06/15/2000	S
4	Abarelix (Plenaxis)	Speciality European	GnRH receptor antagonist	Advanced prostate cancer	11/25/2003	P
5	Degarelix acetate (Firmagon)	Ferring	GnRH receptor antagonist	Advanced prostate cancer	12/24/2008	S
6	Cabazitaxel (Jevtana kit)	Sanofi	Microtubule-stabilizing agent	Prostate cancer	06/17/2010	P
7	Abiraterone acetate (Zytiga)	Janssen Biotech	CYP17A1 inhibitor	Metastatic castration-resistant prostate cancer	04/28/2011	P
8	Enzalutamide (Xtandi)	Astellas	Androgen receptor inhibitor	Metastatic castration-resistant prostate cancer	08/31/2012	P
9	Radium Ra-223 dichloride (Xofigo)	Bayer	α-particle-emitting radioactive therapeutic	Castration-resistant prostate cancer	05/15/2013	P
10	Apalutamide (Erleada)	Janssen Biotech	Androgen receptor inhibitor	Prostate cancer	02/14/2018	P
11	Darolutamide (Nubeqa)	Bayer	Androgen receptor inhibitor	Prostate cancer	07/30/2019	P
12	Relugolix (Orgovyx)	Myovant Sciences	GnRH receptor antagonist	Prostate cancer	12/18/2020	P

	Drug (brand name)	Sponsor				
	Therapeutic drugs for urologic cancers					
1	Aldesleukin (Proleukin)	Chiron	Recombinant human interleukin-2	Metastatic renal cell carcinoma	05/05/1992	O
2	Sorafenib tosylate (Nexavar)	Bayer	Multi-target TKI (VEGFR2/3, PDGFRβ, FLT3, KIT, RAF1, and BRAF)	Advanced renal cell carcinoma	12/01/2005	P, O
3	Temsirolimus (Torisel)	Merck & Co	Mammalian target of rapamycin (mTOR) inhibitor	Advanced renal cell carcinoma	05/30/2007	P, O
4	Everolimus (Afinitor)	Novartis	Mammalian target of rapamycin (mTOR) inhibitor	Advanced renal cell carcinoma	03/30/2009	P
5	Pazopanib HCl (Votrient)	Novartis	Multi-target TKI (VEGFRs, PDGFRα/β, FGFR1/2, KIT)	Advanced renal cell carcinoma	10/19/2009	S
6	Axitinib (Inlyta)	Pfizer	VEGFRs inhibitor	Advanced renal cell carcinoma	01/27/2012	S
7	Tivozanib HCl (Fotivda)	Aveo Pharms	VEGFRs inhibitor	Advanced renal cell carcinoma	03/10/2021	S
8	Atezolizumab (Tecentriq)	Genentech	PDL1-directed mAb	Advanced or metastatic urothelial carcinoma	05/18/2016	P
9	Durvalumab (Imfinzi)	AstraZeneca	PDL1-directed mAb	Urothelial carcinoma	05/01/2017	P
10	Enfortumab vedotin (Padcev)	Astellas	Nectin-4-directed antibody-drug conjugate (ADC)	Urothelial carcinoma	12/18/2019	P
11	Valrubicin (Valstar)	Endo Pharm	A semisynthetic analog of anthracycline doxorubicin	BCG-refractory CIS of urinary bladder cancer	09/25/1998	P, O
12	Erdafitinib (Balversa)	Janssen Biotech	Fibroblast growth factor receptor (FGFR) inhibitor	Bladder cancer	04/12/2019	P

Supplementary data: Table 3 – FDA-approved therapeutic drugs for solid tumors (Continued)

	Drug (brand name)	Sponsor	Properties	Indication	Approval date	Review
	Therapeutic drugs for melanoma and other skin cancers					
1	Ipilimumab (Yervoy)	Bristol-Myers Squibb	Cytotoxic T-lymphocyte antigen 4 (CTLA-4)-directed mAb	Unresectable or metastatic melanoma	03/25/2011	P, O
2	Vemurafenib (Zelboraf)	Roche	BRAF inhibitor	BRAF-positive unresectable or metastatic melanoma	08/17/2011	P, O
3	Dabrafenib mesylate (Tafinlar)	Novartis	Kinase inhibitor with activity against $BRAF^{V600E/D/K}$, wild-type BRAF and other kinases	Unresectable or metastatic melanoma with *BRAF*^{V600E} mutation	05/29/2013	S, O
4	Trametinib dimethyl sulfoxide (Mekinist)	Novartis	MEK1 and MEK2 kinase inhibitor	Unresectable or metastatic melanoma with *BRAF*^{V600E} mutation	05/29/2013	S, O
5	Pembrolizumab (Keytruda)	Merck & Co	PD1-directed mAb	Metastatic melanoma	09/04/2014	P, O
6	Nivolumab (Opdivo)	Bristol-Myers Squibb	PD1-directed mAb	Unresectable or metastatic melanoma	12/22/2014	P, O
7	Cobimetinib fumarate (Cotellic)	Genentech	MEK inhibitor	Melanoma with *BRAF*$^{V600E/K}$ mutations	11/10/2015	P, O
8	Encorafenib (Braftovi)	Array BioPharma	BRAF inhibitor	*BRAF*-mutated melanoma	06/27/2018	S, O
9	Binimetinib (Mektovi)	Array BioPharma	MEK inhibitor	*BRAF*-mutated melanoma	06/27/2018	S, O
10	Vismodegib (Erivedge)	Genentech	Smoothened (Hedgehog pathway) inhibitor	Metastatic or locally advanced basal cell carcinoma	01/30/2012	P
11	Sonidegib phosphate (Odomzo)	Sun Pharma Global	Smoothened inhibitor	Basal cell carcinoma	07/24/2015	S
12	Avelumab (Bavencio)	Emd Serono	PDL1-directed mAb	Merkel cell carcinoma	03/23/2017	P, O
13	Cemiplimab (Libtayo)	Regeneron Pharmaceuticals	Programmed death receptor-1 (PD1)-directed mAb	Metastatic or locally advanced cutaneous squamous cell carcinoma (CSCC)	09/28/2018	P

	Drug (brand name)	Sponsor	Properties	Indication	Approval date	Review
	Therapeutic drugs for thyroid cancers and other solid tumors					
1	Thyrotropin alfa (Thyrogen)	Genzyme	Recombinant human thyroid stimulating hormone	Diagnostic testing for thyroid tissue remnants following radioiodine ablation, and alongside radioactive agents to destroy thyroid tissue	11/30/1998	O
2	Vandetanib (Caprelsa)	Genzyme	Multi-target TKI (VEGFR2/3, EGFR, and RET)	Unresectable or metastatic medullary thyroid cancer	04/06/2011	P, O
3	Cabozantinib S-malate (Cometriq)	Exelixis	Multi-target TKI (VEGFRs, MET, RET, FLT3, KIT, TIE2, and AXL)	Progressive, metastatic medullary thyroid cancer	11/29/2012	P, O
4	Lenvatinib mesylate (Lenvima)	Eisai	Multi-target TKI (VEGFRs, FGFRs, PDGFRα, RET, and KIT)	Thyroid cancer	02/13/2015	P, O
5	Alitretinoin (Panretin)	Concordia	9-cis-retinoic acid, a form of vitamin A	AIDS-related Kaposi's sarcoma	02/02/1999	P, O
6	Temozolomide (Temodar)	Merck & Co	DNA alkylating agent	Glioblastoma	08/11/1999	P, O
7	Dinutuximab (Unituxin)	United Therap	GD2-directed mAb	High-risk neuroblastoma	03/10/2015	P, O
8	Naxitamab (Danyelza)	Y-mAbs Therapeutics	GD2-directed mAb	High-risk neuroblastoma	11/25/2020	P, O
9	Pemetrexed disodium (Alimta)	Eli Lilly	Folate analog	Malignant pleural mesothelioma	02/04/2004	P, O
10	Trabectedin (Yondelis)	Janssen Prods	Alkylating drug, interacts with the minor groove of DNA	Liposarcoma or leiomyosarcoma	10/23/2015	P, O
11	Olaratumab (Lartruvo)	Eli Lilly & Co	PDGFRα-directed mAb	Soft tissue sarcoma	10/19/2016	P, O
12	Larotrectinib sulfate (Vitrakvi)	Bayer	TRKs inhibitor	NTRK-positive solid cancers	11/26/2018	P, O
13	Pexidartinib HCl (Turalio)	Daiichi Sankyo	CSF1R, KIT, and FLT3 inhibitor	Tenosynovial giant cell tumor	08/02/2019	P, O
14	Tazemetostat (Tazverik)	Epizyme Inc	Enhancer of Zeste Homolog 2 (EZH2) inhibitor	Epithelioid sarcoma	01/23/2020	P, O

Supplementary data: Table 4 – ICBs: first approval and primary indications in the USA and China

	Drug (brand name)	Sponsor	Target	IgG type	Primary indication	Approval date
	Food and Drug Administration (USA), N = 9					
1	Ipilimumab (Yervoy)	Bristol-Myers Squibb	CTLA-4	IgG1κ	Malignant melanoma	03/25/2011
2	Pembrolizumab (Keytruda)	Merck	PD-1	IgG4κ	Advanced, unresectable or metastatic melanoma	09/04/2014
3	Nivolumab (Opdivo)	Bristol-Myers Squibb	PD-1	IgG4κ	Unresectable or metastatic melanoma	12/22/2014
4	Atezolizumab (Tecentriq)	Genentech	PD-L1	IgG1κ	Urothelial carcinoma	05/18/2016
5	Avelumab (Bavencio)	Emd Serono	PD-L1	IgG1λ	Metastatic Merkel cell carcinoma	03/23/2017
6	Durvalumab (Imfinzi)	AstraZeneca	PD-L1	IgG1κ	Locally advanced or metastatic urothelial carcinoma	05/01/2017
7	Cemiplimab (Libtayo)	Regeneron	PD-1	IgG4κ	Locally advanced or metastatic Cutaneous squamous cell carcinoma	09/28/2018
8	Dostarlimab (Jemperli)	GlaxoSmithKline	PD-1	IgG4κ	Mismatch repair deficient, recurrent or advanced endometrial cancer	04/22/2021
9	Nivolumab/Relatlimab (Opdualag)	Bristol-Myers Squibb	PD-1/LAG-3	IgG4κ	Unresectable or metastatic melanoma	03/18/2022
	National Medical Products Administration (China), N = 11					
10	Toripalimab (Tuoyi)	Junshi Bio	PD-1	IgG4	Unresectable or metastatic melanoma	12/17/2018
11	Sintilimab (Tyvyt)	Innovent Bio, Eli Lilly	PD-1	IgG4	Relapsed or refractory classical Hodgkin's lymphoma	12/27/2018
12	Camrelizumab (AiRuiKa)	Hengrui Med	PD-1	IgG4κ	Relapsed or refractory classical Hodgkin's lymphoma	05/31/2019
13	Tislelizumab (百泽安)	BeiGene	PD-1	IgG4	Relapsed or refractory classical Hodgkin's lymphoma	12/26/2019
14	Penpulimab (安尼可)	Akeso Bio, Chia Tai Tianqing	PD-1	IgG1	Relapsed or refractory classical Hodgkin's lymphoma	08/05/2021
15	Zimberelimab (誉妥)	Gloria Bio, Arcus Bio, Taiho Pharma	PD-1	IgG4	Relapsed or refractory classical Hodgkin's lymphoma	08/25/2021
16	Envafolimab (恩维达)	Alphamab Oncology	PD-L1	Nanobody	Advanced mismatch repair deficient solid tumors	11/24/2021
17	Sugemalimab (Cejemly)	CStone Pharma	PD-L1	IgG4	*EGFR*^{Mut} and *ALK*^{Neg} metastatic non-small cell lung cancer	12/21/2021
18	Serplulimab (汉斯状)	Henlius Bio	PD-1	IgG4	Advanced, unresectable or metastatic microsatellite instability-high solid tumors	03/25/2022
19	Cadonilimab (开坦尼)	Akeso Bio	PD-1/CTLA-4	IgG1	Relapsed or metastatic cervical cancer	06/29/2022
20	Pucotenlimab (普佑恒)	Lepu Bio	PD-1	IgG4	Microsatellite instability-high solid tumors	07/22/2022

Reference

1. Kaitin KI, Manocchia M, Seibring M, Lasagna L: **The new drug approvals of 1990, 1991, and 1992: trends in drug development**. *J Clin Pharmacol* 1994, **34**(2):120-127.
2. Beary JF, Robert Eaton C, Copmann TL, Wierenga DE: **U.S. drug and biologic product approvals during 1993**. *Drug Development Research* 1994, **32**(3):127-133.
3. Beary JF, Duchaine CM, Rhein RW: **U.S. drug and biologic approvals in 1994-1995**. *Drug Development Research* 1996, **37**(4):197-207.
4. Beary JF, Robillard LE, Woollett GR, Siegfried JD, White TX, Shriver DA: **U.S. drug and biologic approvals in 1996**. *Drug Development Research* 1997, **40**(4):275-291.
5. Beary JF, Siegfried JD, Tavares R: **US drug and biologic approvals in 1997**. *Drug Development Research* 1998, **44**(2-3):114-129.
6. Spilker B, FitzSimmons S, Horan M: **US drug and biologic approvals in 1998**. *Drug Development Research* 1999, **48**(4):139-153.
7. Kaitin KI, Cairns C: **The New Drug Approvals of 1999, 2000, and 2001: Drug Development Trends a Decade after Passage of the Prescription Drug User Fee Act of 1992**. *Drug Information Journal* 2003, **37**(4):357-371.
8. Frantz S, Smith A: **New drug approvals for 2002**. *Nat Rev Drug Discov* 2003, **2**(2):95-96.
9. Frantz S: **2003 approvals: a year of innovation and upward trends**. *Nat Rev Drug Discov* 2004, **3**(2):103-105.
10. Frantz S: **2004 approvals: the demise of the blockbuster?** *Nat Rev Drug Discov* 2005, **4**(2):93-94.
11. Frantz S: **2005 approvals: Safety first**. *Nature Reviews Drug Discovery* 2006, **5**(2):92-93.
12. Owens J: **2006 drug approvals: finding the niche**. *Nat Rev Drug Discov* 2007, **6**(2):99-101.
13. Hughes B: **2007 FDA drug approvals: a year of flux**. *Nat Rev Drug Discov* 2008, **7**(2):107-109.
14. Hughes B: **2008 FDA drug approvals**. *Nat Rev Drug Discov* 2009, **8**(2):93-96.
15. Hughes B: **2009 FDA drug approvals**. *Nat Rev Drug Discov* 2010, **9**(2):89-92.
16. Mullard A: **2010 FDA drug approvals**. *Nat Rev Drug Discov* 2011, **10**(2):82-85.
17. Mullard A: **2011 FDA drug approvals**. *Nat Rev Drug Discov* 2012, **11**(2):91-94.
18. Mullard A: **2012 FDA drug approvals**. *Nat Rev Drug Discov* 2013, **12**(2):87-90.
19. Mullard A: **2013 FDA drug approvals**. *Nat Rev Drug Discov* 2014, **13**(2):85-89.
20. Mullard A: **2014 FDA drug approvals**. *Nat Rev Drug Discov* 2015, **14**(2):77-81.
21. Mullard A: **2015 FDA drug approvals**. *Nat Rev Drug Discov* 2016, **15**(2):73-76.
22. Mullard A: **2016 FDA drug approvals**. *Nat Rev Drug Discov* 2017, **16**(2):73-76.
23. Mullard A: **2017 FDA drug approvals**. *Nat Rev Drug Discov* 2018, **17**(2):81-85.
24. Mullard A: **2018 FDA drug approvals**. *Nat Rev Drug Discov* 2019, **18**(2):85-89.
25. Mullard A: **2019 FDA drug approvals**. *Nat Rev Drug Discov* 2020, **19**(2):79-84.
26. Mullard A: **2020 FDA drug approvals**. *Nat Rev Drug Discov* 2021, **20**(2):85-90.
27. Mullard A: **2021 FDA approvals**. *Nat Rev Drug Discov* 2022, **21**(2):83-88.
28. Cameron F, Whiteside G, Perry C: **Ipilimumab: first global approval**. *Drugs* 2011, **71**(8):1093-1104.

29. Poole RM: **Pembrolizumab: first global approval**. *Drugs* 2014, **74**(16):1973-1981.
30. Scott LJ: **Nivolumab: A Review in Advanced Melanoma**. *Drugs* 2015, **75**(12):1413-1424.
31. Markham A: **Atezolizumab: First Global Approval**. *Drugs* 2016, **76**(12):1227-1232.
32. Kim ES: **Avelumab: First Global Approval**. *Drugs* 2017, **77**(8):929-937.
33. Syed YY: **Durvalumab: First Global Approval**. *Drugs* 2017, **77**(12):1369-1376.
34. Markham A, Duggan S: **Cemiplimab: First Global Approval**. *Drugs* 2018, **78**(17):1841-1846.
35. Markham A: **Dostarlimab: First Approval**. *Drugs* 2021, **81**(10):1213-1219.
36. Paik J: **Nivolumab Plus Relatlimab: First Approval**. *Drugs* 2022, **82**(8):925-931.
37. Keam SJ: **Toripalimab: First Global Approval**. *Drugs* 2019, **79**(5):573-578.
38. Hoy SM: **Sintilimab: First Global Approval**. *Drugs* 2019, **79**(3):341-346.
39. Markham A, Keam SJ: **Camrelizumab: First Global Approval**. *Drugs* 2019, **79**(12):1355-1361.
40. Lee A, Keam SJ: **Tislelizumab: First Approval**. *Drugs* 2020, **80**(6):617-624.
41. Dhillon S: **Penpulimab: First Approval**. *Drugs* 2021, **81**(18):2159-2166.
42. Markham A: **Zimberelimab: First Approval**. *Drugs* 2021, **81**(17):2063-2068.
43. Markham A: **Envafolimab: First Approval**. *Drugs* 2022, **82**(2):235-240.
44. Dhillon S, Duggan S: **Sugemalimab: First Approval**. *Drugs* 2022, **82**(5):593-599.
45. Lee A: **Serplulimab: First Approval**. *Drugs* 2022, **82**(10):1137-1141.
46. Keam SJ: **Cadonilimab: First Approval**. *Drugs* 2022.
